# Changes in the accreditation standards of medical schools by the Korean Institute of Medical Education and Evaluation from 2000 to 2019

**DOI:** 10.3352/jeehp.2020.17.2

**Published:** 2020-04-07

**Authors:** Hyo Hyun Yoo, Mi Kyung Kim, Yoo Sang Yoon, Keun Mi Lee, Jong Hun Lee, Seung-Jae Hong, Jung –Sik Huh, Won Kyun Park

**Affiliations:** 1 Department of Medical Education, Jeonbuk National University Medical School, Jeonju, Korea; 2 Department of Pathology, Chung-Ang University College of Medicine, Seoul, Korea; 3 Department of Emergency Medicine, The Institute of Medical Humanities, Inje University College of Medicine, Busan, Korea; 4 Department of Family Medicine, Yeungnam University College of Medicine, Daegu, Korea; 5 Department of Plastic Surgery, Eulji University School of Medicine, Daejeon, Korea; 6 Department of Rheumatology, Kyung Hee University School of Medicine, Seoul, Korea; 7 Department of Urology, Institute of Medical Science, Jeju National University School of Medicine, Jeju, Korea; 8 Department of Medical Education, Keimyung University School of Medicine, Daegu, Korea; Hallym University, Korea

**Keywords:** Accreditation, Curriculum, Longitudinal studies, Medical schools, Republic of Korea

## Abstract

This review presents information on changes in the accreditation standards of medical schools in Korea by the Korean Institute of Medical Education and Evaluation (KIMEE) from 2000 to 2019. Specifically, the following aspects are explained: the development process, setting principles and directions, evaluation items, characteristics of the standards, and validity testing over the course of 4 cycles. The first cycle of accreditation (2000–2005) focused on ensuring the minimum requirements for the educational environment. The evaluation criteria emphasized the core elements of medical education, including facilities and human resources. The second cycle of accreditation (2007–2010) emphasized universities’ commitment to social accountability and the pursuit of excellence in medical education. It raised the importance of qualitative standards for judging the content and quality of education. In the post-second accreditation cycle (2012–2018) which means third accreditation cycle, accreditation criteria were developed to standardize the educational environment and programs and to be used for curriculum development in order to continually improve the quality of basic medical education. Most recently, the ASK 2019 (Accreditation Standards of KIMEE 2019) accreditation cycle focused on qualitative evaluations in accordance with the World Federation of Medical Education’s accreditation criteria to reach the international level of basic medical education, which emphasizes the need for a student-centered curriculum, communication with society, and evaluation through a comprehensive basic medical education course. The KIMEE has developed a basic medical education evaluation and accreditation system in a step-by-step manner, as outlined above. Understanding previous processes will be helpful for the future development of accreditation criteria for medical schools in Korea.

## Introduction

### Background/rationale

The Korean Institute of Medical Education and Evaluation (KIMEE) operates the accreditation system of medical schools in Korea, with the goal of supporting continuous improvements in the educational environment and programs. The accreditation standards have changed over the past 20 years (from 2000 to 2019) across the first cycle (2000–2005), the second cycle (2007–2011), the post-second cycle (2012–2018), and the Accreditation Standards of KIMEE 2019 (ASK 2019) cycle. A report was issued on the changes in accreditation standards and development directions of the KIMEE (Supplement 1), which is summarized in this review article.

### Objectives

This study aimed to explain the following aspects of the accreditation process: the development process, setting principles and directions, evaluation items, characteristics of the standards, and validity testing in each cycle. It may be worthwhile to create a record of the background and features of each accreditation cycle for the sake of future generations who are involved with the accreditation of medical schools, both in the KIMEE and at various medical schools throughout Korea.

## The first cycle (2000–2005)

### Development process

To prepare for the first cycle of medical school accreditation, the Accreditation Board for Medical Education in Korea (ABMEK), the predecessor of the KIMEE, formed an Evaluation Criteria Working Committee to develop evaluation accreditation standards in 1998 [1]. The Evaluation Criteria Working Committee of the ABMEK developed draft versions of accreditation criteria for medical universities through meetings, workshops, and opinion-gathering, and these draft criteria were applied to preliminary evaluations of newly established medical schools in 1999. By supplementing the deficiencies identified in the preliminary evaluation, the criteria for the first cycle of medical school accreditation were established, and through this process, 41 medical schools were evaluated over the 5-year period from 2000 to 2004 [2].

### Setting principles and directions

The criteria for the first cycle of medical school accreditation were based on the CIPP evaluation model [3], which provides a systematic approach by setting the following 5 principles for evaluation criteria: usefulness, adequacy, clarity, feasibility, and future orientation. These accreditation criteria aimed to present future-oriented considerations in light of the real-world circumstances of Korea, to improve the quality of medical schools and to foster greater social accountability, and to ensure the minimum necessary educational conditions and curriculum. The evaluation areas were divided into mandatory standards and recommended standards. Mandatory criteria were those that each medical school was required to meet for the purposes of basic accountability in medical education. Recommended criteria were future-oriented standards that encouraged medical schools to develop themselves to achieve the recommended levels. The KIMEE took into account the “Rules for establishing a medical school” and “Medical school evaluation standards” proposed by the Korean Council for University Education in 1996 [2]. In particular, mandatory standards related to facilities and working personnel were aligned with the “Rules for establishing a medical school.” The balance between quantitative and qualitative criteria was also considered.

### Evaluation items

The evaluation items for the first accreditation were based on the 93 items proposed by the Korean Council for University Education in 1996, excluding graduate schools. Fifty evaluation items were selected in the following 5 areas: educational goals and curriculum; students; professors; facilities and equipment; and administration and finance. Of the 50 items, 18 items were mandatory, and 32 items were recommended (Table 1).

### Characteristics of the standards

The educational environment and the entire curriculum of medical schools were evaluated, including the following components: the circumstances under which the curriculum was implemented; the degree to which available resources were invested to achieve the objectives; the interactions among the resources allocated to achieve the objectives; and the achievements of education, research, and community service. The items that were used reflected core elements of medical education. Specific aspects of medical education were also analyzed, including medicine-related interpersonal skills, student research activities, the distinction between the basic and clinical curriculum, and the distinction between basic and clinical teaching. This level of specificity is infrequently addressed in other educational fields. Universities were recommended to provide a plan for improving the quality of medical education based on a long-term vision.

### Validity testing of the measurement tool

A study was conducted to examine the classification of validity and predictive validity of the 50 items used as accreditation standards for medical schools in 2000 (Table 1). The validity of the standards for medical school accreditation varied depending on whether schools were classified as leading, average, or unconcerned; therefore, applying uniform standards to all types of schools was concluded not to be a reasonable approach to enhancing the quality of medical education. The standards of accreditation predicted 84.4% of students’ level of satisfaction [4]. Another study surveyed 309 professors to test the validity of the measurement tool, which contained 50 items. In that study, 70.2% of the faculty members who responded stated that the accreditation system achieved its goals, and 80.7% of them pointed out the necessity of a continuing accreditation system. As a result of the 5-year accreditation process, 32 medical schools were accredited and 9 medical schools were accredited on a probationary basis. The satisfaction level for items regarding students and facilities was lower than that for items regarding curriculum and administration/finance [5].

## The second cycle (2007–2011)

### Development process

With the end of the first cycle of the medical school accreditation project in 2005, opinions were solicited on problems with the standards of the first cycle in order to provide directions for standards to be applied in the second cycle of accreditation. The committee held several meetings and workshops to develop the accreditation criteria for the second cycle, which were revised and supplemented through 2 public hearings in 2005 and opinions from 41 medical schools. A special committee was formed to discuss and address these issues and to increase the reliability of the application and interpretation of standards by linking the accreditation evaluation criteria with the Medical Education Database (Korea Medical School Information System). Through this process, plans for the second cycle of the accreditation criteria were completed in 2007.

### Setting principles and direction

The principles of the standards focused on social responsibility and educational excellence. According to the degree of accountability of each university, the criteria were divided into essential and recommended, as well as those focusing on excellence. Essential criteria were related to the performance of universities regarding basic accountability, while recommended criteria were related to improvements in the quality of education. Criteria focusing on excellence were related to the pursuit of excellence in education. Furthermore, to improve the quality of medical education to the international level, international accreditation criteria were satisfied, including the standards of the World Medical Education Association (WFME), the International Medical Education Organization, the American Medical Education Joint Commission, and the British Medical Association.

### Evaluation items

In total, 109 evaluation criteria were assessed in the second cycle, including 41 essential criteria, 34 recommended criteria, and 34 criteria related to excellence in the areas of operation of the university system, educational goals and curriculum, students, faculty, facilities and equipment, and graduate education [5,6] (Table 2).

### Characteristics of the standards

The evaluation criteria were divided into essential and recommended criteria, as well as criteria regarding excellence. While the importance of quantitative standards was reduced, the importance of qualitative evaluation criteria for judging the actual content and quality of medical education increased. Competence after graduation was introduced as a criterion to be evaluated, as were the structural standardization of university hospitals and graduate school programs [6].

### Validity test of the measurement tool

A meta-analysis of items used in the criteria for the second cycle of accreditation found that most of them were important and appropriate, except for a few items. The less critical items were alumni and community standards in the field of university operation, complementary and integrated medicine as educational goals, and areas related to the curriculum. For adequacy, low scores were found for the following items: complementary and integrative medicine in the curriculum area; the proportion of graduates who entered fields other than clinical medicine in the student area; the ratio of basic and clinical medicine faculty numbers in the faculty area; and ensuring places in university-affiliated hospitals, information system operation, and faculty spaces in the facilities area. The validity of the criteria used in the second cycle of accreditation was high. It was suggested that ongoing efforts should be made to improve deficiencies in qualitative aspects that cannot be measured by quantitative standards and to develop accreditation standards that balance the need of respecting diversity and pursuing specialized skills and excellence at each medical school [6].

The goodness of fit was tested for the 109 items used in the second cycle of accreditation. Dichotomous data on 109 items from 40 medical schools were analyzed according to the Rasch model. All items were in the acceptable range in terms of the infit mean square, while 107 items were in the acceptable range for the outfit mean square. These findings meant that 2 items were outliers: one was “the college must have education, research, and patient care policies regarding social accountability and such policies must be practiced” and the other was “the ratio of faculty members who graduated from the same college was 70% or less among the total faculty of the medical school.” Therefore, the items used in the second cycle of accreditation from 2007 to 2011 by the KIMEE were favorable [7].

## Post-second cycle (2012–2018)

### Development process

In order to eliminate the concept of the cycle, the KIMEE decided to ask medical schools to submit mandatory self-assessment reports every 2 years in the next round of the accreditation process. Furthermore, the KIMEE was recognized as the medical education accreditation agency of South Korea by the Ministry of Education for the 5-year period starting on May 12, 2014, based on the Higher Education Act [8]. Some of the accreditation criteria were revised before and after recognition by the Ministry of Education.

#### Development of criteria before recognition by the Ministry of Education

Starting in 2010, the KIMEE Standards Committee conducted several meetings and workshops to prepare the post-second cycle accreditation criteria based on the results of a meta-analysis of the previous 2 cycles and opinion-gathering through public hearings. The accreditation criteria were approved in January 2011 and applied starting in 2012.

#### Development of criteria after recognition by the Ministry of Education

In November 2013, 36 quantitative items were reviewed out of 97 basic standard items according to the request of the Ministry of Education to revise items to be qualitative. Of the 36 quantitative items, 27 items were revised to be qualitative, including 2 sub-areas of the operational system of the university, 5 sub-areas of basic medical education, 2 sub-areas of students, 4 sub-areas of faculty members, 3 sub-areas of facilities and equipment, and 1 sub-area of graduate education. The remaining 9 quantitative items were kept; these items were related to curriculum operation, evaluation, and clinical practice (duration, methodology, etc.) .

### Setting principles and directions

To improve the accreditation standards, the following principles were introduced after a review of the results of the meta-analysis of the second accreditation cycle [6]: first, the need to establish the minimum level of the educational environment and educational programs; second, international-level standards; third, standards respecting the specialization and excellence of each medical school as part of pursuing the diversity of medical schools; fourth, long-term, future-oriented standards; fifth, standards to fulfill society’s demands; and sixth, standards for performance-based education.

### Evaluation items

There were 140 post-second-cycle evaluation items, including 97 basic items and 43 supplemental items in 6 areas with 20 sub-areas. The number of items in each area remained the same after the KIMEE was recognized by the Ministry of Education, but 27 quantitative items were revised to qualitative items and 1 item in the area of basic medical education was added (Table 3).

### Characteristics of the standards

The curriculum based on learning outcomes focused on the qualities that graduates could achieve above a certain level of competence as doctors, reflecting the international trend of emphasizing competence-based education for the development of students’ performance. The qualitative criteria were strengthened with the goal of improving the environment and programs of basic medical education and to protect the right of students to receive a high-quality education and to guarantee the health of the public. The essential items and recommended items of the second accreditation cycle were combined into the basic items, which reflected the steps necessary to ensure the minimum requirements of medical education. In the area of students, the aspects relating to student protection and rights were strengthened [6,8].

### Validity test of the measurement tool

A meta-analysis of the post-second cycle accreditation was conducted to determine whether the evaluation items helped universities to develop. In a self-evaluation, 77.8% of faculty members stated that the basic items were helpful, while 52.6% of them reported that the supplemental items contributed to the development of the university [9].

## ASK 2019 (2019–present)

### Development process

To be recognized by the WFME as a medical school accreditation agency, the KIMEE conducted a self-assessment through expert meetings and workshops starting in 2013. The KIMEE received WFME certification as an accreditation agency in 2015. Furthermore, the KIMEE developed the ASK standards suitable for the circumstances of medical education in Korea based on the WFME evaluation standards. To do so, the KIMEE collected opinions through 3 sets of public hearings in 2015 and 2016. The ASK 2018 criteria were developed through meetings with related institutions such as the Korea Association of Medical Colleges, the Korean Association for Basic Medical Scientists, and the Korean Society of Medical Education. ASK 2019 was announced in May 2017 as a tool to be fully implemented starting in 2019. The KIMEE also developed guidelines for the application of ASK 2019 and announced them in February 2018.

### Setting principles and directions

The following directions were set in consideration of the circumstances of medical schools in Korea based on the WFME global standards for quality improvement in basic medical education (2015 revision) [10]. The overall structure and composition were also revised according to the WFME global standards. The basic items were those that universities were required to meet, corresponding to the purpose of accreditation. Supplemental items were desirable, future-oriented standards for universities and basic medical education, aiming to reform medical education to follow international-level best practices. The following principles were followed: First, it was necessary to maintain a balance between the WFME evaluation items and the post-second accreditation items. Second, WFME evaluation items that did not fit the circumstances of medical education in Korea would not be used. Third, some of the WFME evaluation items would be added or revised in accordance with the post-second cycle items according to the circumstances of medical education in Korea. Fourth, any evaluation items not corresponding to the basic medical curriculum would not be included. Fifth, legal requirements specific to Korea would not be included in the evaluation items.

### Evaluation items

The ASK 2019 consisted of a total of 143 items, consisting of 92 basic items and 51 supplemental items in 9 areas and 34 sub-areas (Table 4).

### Characteristics of the standards

#### Mission and outcomes

Based on its founding philosophy, each university should establish its mission by actively gathering opinions from stakeholders such as students, faculty members, university staff, headquarters staff, health care workers, and community medical associations. If graduates’ performance can be assessed before the institution’s mission is established, a careful review of the graduates’ performance and the intended educational outcomes is necessary.

#### Curriculum

The basic medical curriculum area of the post-second cycle accreditation was divided into 3 components: curriculum overview, curriculum development and support, and curriculum composition and operation. The curriculum must be organized based on the institution’s mission and graduates’ achievements. As such, the curriculum must be continuously revised and supplemented to reflect changes in the medical environment. The basic medical curriculum must be linked to a curriculum that incorporates post-graduate education and lifelong learning, and the curriculum should be operated in a way that reflects community opinions and needs. This means that faculty, students, staff, alumni, parents, and health care stakeholders should all participate in training programs for physicians who are able to carry out primary care.

#### Student assessment

It is proposed that student assessments should be operated systematically. In addition to paper and pencil-based assessments, students’ knowledge, skills, and attitudes are required to be evaluated. An emphasis is placed on student assessments to ensure that they achieve the intended educational outcomes through a framework grounded in performance-based education.

#### Students

The evaluation items did not change dramatically compared to the post-second cycle accreditation. However, academic guidance for students who do not succeed in passing to a higher grade and students’ participation in medical school committees were changed from supplemental standards to basic standards.

#### Faculty

Assessment of faculty members’ research performance, including the number of journal articles or amount of research funding, was excluded from the post-second cycle accreditation. Instead, the ASK 2019 criteria evaluate the degree of educational and development support for faculty by medical schools, with an emphasis on the balance among education, research, and social service in faculty members’ performance. These criteria assess whether the composition of faculty members in each curriculum is balanced, whether there is a policy to support all faculty members in understanding the curriculum, and whether the medical school supports faculty members in developing their competencies in integrated education.

#### Educational resources

This area is characterized by the transition of educational resources to student-centered education. From the student-centered perspective, evaluation items include clinical practice resources, information technology, medical research, educational expertise, and exchange programs. One of the new additions is educational expertise, which refers to physicians, pedagogues, sociologists, and institutions that deal with the processes, practices, and problems of medical education. In recent years, the flow of medical education has required extensive changes throughout the educational process. The demand for development, improvement, and evolution of new curricula is inevitable; therefore, a strategy is needed to solve these problems. This area aims to secure educational expertise and to reflect natural choices for the times.

#### Education evaluation

An emphasis is placed on expanding the curriculum beyond the elements that are traditionally considered as part of the curriculum in order to facilitate the continuous improvement of education-related activities. In other words, it is necessary to systematically evaluate all courses related to education, including the curriculum as a whole, from the time of enrollment to graduation, through planning, implementation, data collection and analysis, and feedback. Therefore, while the post-second cycle accreditation criteria focused on curriculum evaluation, the ASK 2019 criteria regularly monitor and evaluate all activities related to education, including the curriculum, educational resources, faculty, staff, students, university culture, and learning environment. A systematic implementation is required to ensure a comprehensive evaluation capable of contributing to continuous improvements.

#### Operational system and administration

Considering the similarities of the ASK 2019 criteria to the post-second cycle accreditation criteria, it is necessary for medical schools to have an administrative system capable of executing the school affairs, budgeting systematically, and maintaining operational standards even if the dean changes.

#### Continuous improvement

To improve competitiveness, medical schools should establish mid- and long-term development plans for constant quality improvement.

#### Validity test

Validity testing will be done after finishing the accreditation process.

## Conclusion

The KIMEE was certified by the Ministry of Education of the Korean Government as an official accreditor for basic medical education in 2014 in response to ongoing requests by medical educators [11]. Meanwhile, a national, large-scale objective structured clinical examination (OSCE) was introduced as part of the licensing examination to improve practical clinical education in the last 2 years of the medical curriculum in 2010. This was enabled by the accreditation standards that prescribed the installation of skill labs and OSCEs at medical schools [12]. At the WFME Recognition Committee meeting held in Melbourne, Australia, on September 19, 2016, the KIMEE was recognized as the accreditation agency in Korea for 10 years from the time of recognition.

The WFME-centered international standardization of medical education has been developed to ensure the minimum quality of medical practice through a common accreditation system of medical schools. Accordingly, to raise medical education to the international level, evaluation standards corresponding to the international level must be developed. The KIMEE has developed an accreditation system of medical schools in South Korea in a step-by-step manner for the past 20 years. The outcomes were remarkable, as highlighted herein.

Most notably, the ASK 2019 system was established, which is comparable to the top-tier systems throughout the world. It is no exaggeration to state that the history of the development of medical education in Korea during the past 20 years has proceeded hand in hand with the history of the development of the accreditation of medical schools by the KIMEE. Understanding previous processes will be helpful for developing items for the accreditation of medical schools in Korea in the future. Furthermore, the following 5 assessment elements are suggested for the further development of the accreditation process: institution- and culture-based assessments; future-oriented assessments; excellence- and diversity-oriented assessments; qualitative assessments with higher validity and reliability; and authentic assessments.

## Figures and Tables

**Table 1.** Evaluation areas and the number of items for the first cycle of medical school accreditation in Korea by the Korean Institute of Medical Education and Evaluation

Area	Sub-area	No. of items		Total
		Mandatory standards	Recommended standards	
1. Educational goals and curriculum	1-1. Composition of the goal and effort to achieve it	1	2	3
	1-2. Basic medicine curriculum	2	-	2
	1-3. Clinical medicine curriculum	2	2	4
	1-4. Class guidance and evaluation of lectures	2	2	4
	1-5. Assessment of students’ accomplishments	-	4	4
	1-6. Efforts for curricular improvements	1	1	2
	1-7. Medicine-related courses on interpersonal skills	1	-	1
	Subtotal	9	11	20
2. Students	2-1. Student guidance system	1	1	2
	2-2. Financial support and facilities for students	1	2	3
	2-3. Students’ academic research activities and learning outcomes	-	2	2
	Subtotal	2	5	7
3. Faculty	3-1. Ensuring sufficient staffing of basic and clinical faculty members	1	2	3
	3-2. Research and academic activities of faculty members	1	2	3
	3-3. Faculty development	1	2	3
	Subtotal	3	6	9
4. Facilities and equipment	4-1. Basic supporting facilities for education	3	1	4
	4-2. Facilities for faculty members	-	2	2
	Subtotal	3	3	6
5. Administration and finance	5-1. Administration and operational system	-	3	3
	5-2. Finance	-	2	2
	5-3. Development plan	1	2	3
	Subtotal	1	7	8
Total		18	32	50

**Table 2.** Evaluation areas and the number of items for the second cycle of accreditation of medical schools in Korea by the Korean Institute of Medical Education and Evaluation

Area	Sub-area	No. of items			Total
		Essential standards	Recommended standards	Standards related to excellence	
1. Operation of the system	1-1. Establishment	3	-	-	3
	1-2. Administrative structure and operation of the system	4	-	1	5
	1-3. Administration	1	2	-	3
	1-4. Development plan	1	2	-	3
	1-5. Efforts for improvement	2	1	1	4
	Subtotal	11	5	2	18
2. Goals and curriculum	2-1. Goals and basic system of the curriculum	2	3	2	7
	2-2. Curriculum of basic medical science	3	-	-	3
	2-3. Curriculum of clinical medicine	6	2	2	10
	2-4. Curriculum of humanities and social medicine	1	2	1	4
	2-5. Teaching methods and course evaluation	3	-	2	5
	2-6. Assessment of academic achievement	2	1	2	5
	Subtotal	15	8	9	34
3. Students	3-1. Students’ admission policy and selection of students	-	2	-	2
	3-2. Student guidance system	1	2	2	5
	3-3. Financial support and facilities for students	2	3	3	8
	3-4. Career and learning outcomes after graduation	-	2	1	3
	Subtotal	3	9	6	18
4. Faculty	4-1. Full-time basic and clinical faculty members	2	3	4	9
	4-2. Research and academic activities of faculty members	1	4	4	9
	4-3. Faculty development	3	-	3	6
	Subtotal	6	7	11	24
5. Facilities and equipment	5-1. Facilities and equipment for education	3	4	4	8
	5-2. Facilities and equipment for research	1	4	1	3
	Subtotal	4	2	5	11
6. Graduate education	6-1. Graduate education	-	3	1	4
	Subtotal	-	3	1	4
Total		41	34	34	109

**Table 3.** Evaluation areas and the number of items for the post-second cycle accreditation of medical schools in Korea by the Korean Institute of Medical Education and Evaluation

Area	Sub-area	No. of items	
		Basic	Supplemental
1. Operational system of the university	1-1. Establishment	3	-
	1-2. Administration structure and operation of the system	6	2
	1-3. Finances	3	-
	1-4. Development plan	3	-
	1-5. Efforts for improvement	3	2
	Subtotal	18	4
2. Basic medical education	2-1. Overview of curriculum	3	-
	2-2. Curriculum development and support	5	3
	2-3. Composition and operation of the curriculum	16	3 (4)^a)^
	2-4. Assessment of academic achievement	3	3
	2-5. Evaluation of curriculum and improvement	3	-
	Subtotal	30	9
3. Students	3-1. Admission policies and selection of students	4	1
	3-2. Student guidance system	6	5
	3-3. Well-being and safety of students	7	6
	3-4. Careers after graduation	2	1
	Subtotal	19	13
4. Faculty	4-1. Full-time faculty members	6	6
	4-2. Faculty members’ activities	5	3
	4-3. Faculty development	7	3
	Subtotal	18	12
5. Facilities and equipment	5-1. Facilities and equipment for education	7	3
	5-2. Facilities and equipment for research	2	1
	Subtotal	9	4
6. Graduate education	6-1. Graduate education	3	1
	Subtotal	3	1
Total		97^b)^	43 (44)^a)^

a) Item number increased after recognition of the Korean Institute of Medical Education and Evaluation by the Ministry of Education.

b) Items may be included among both basic and supplemental items.

**Table 4.** Evaluation area, category, and items for the Accreditation Standards of KIMEE 2019 (ASK 2019) for medical schools in Korea by the KIMEE

Evaluation area	Sub-area	No. of items		
		Basic	Supplemental	Total
1. Mission and outcomes	1-1. Mission	3	1	4
	1-2. Institutional autonomy and academic freedom	1	-	1
	1-3. Educational outcomes	3	1	4
	1-4. Participation in the formulation of mission and outcomes	1	1	2
	Subtotal	8	3	11
2. Curriculum	2-1. Curriculum	3	1	4
	2-2. Scientific method	3	-	3
	2-3. Basic medical sciences	2	1	3
	2-4. Medical humanities	1	1	2
	2-5. Clinical sciences and skills	4	3	7
	2-6. Program structure, composition, and duration	2	2	4
	2-7. Curriculum management	2	-	2
	2-8. Linkage with medical practice and the health sector	1	1	2
	Subtotal	18	9	27
3. Student assessments	3-1. Assessment methods	4	1	5
	3-2. Relationship between assessments and learning	4	2	6
	Subtotal	8	3	11
4. Students	4-1. Admission policy and selection	1	3	4
	4-2. Student intake	1	-	1
	4-3. Student counseling and support	6	3	9
	4-4. Student representation	2	-	2
	Subtotal	10	6	16
5. Faculty	5-1. Recruitment and selection policy	6	1	7
	5-2. Faculty activity and development	6	1	7
	Subtotal	12	2	14
6. Educational resources	6-1 Physical facilities	8	1	9
	6-2. Clinical training resources	3	1	4
	6-3. Information technology	1	2	3
	6-4. Medical research and fostering medical scientists	3	1	4
	6-5. Educational expertise	2	3	5
	6-6. Educational exchanges	1	1	2
	Subtotal	18	9	27
7. Education evaluation	7-1. Mechanisms for education monitoring and evaluation	3	1	4
	7-2. Teacher and student feedback	1	1	2
	7-3. Performance of students and graduates	1	1	2
	7-4. Involvement of stakeholders	1	-	1
	Subtotal	6	3	9
8. Operational system and administration	8-1. Operational system	4	2	6
	8-2. Academic leadership	1	1	2
	8-3. Educational budget and resource allocation	2	-	2
	8-4. Administrative staff and management	1	1	2
	8-5. Interaction with the health sector	1	1	2
	Subtotal	9	5	14
9. Continuous improvement	9-0. Continuous improvement	3	11	14
	Subtotal	3	11	14
Total	36 areas	92	51	143

KIMEE, Korean Institute of Medical Education and Evaluation.

## Supplementary materials

Data files are available from Harvard Dataverse https://doi.org/10.7910/DVN/

Supplement 1. Past, present, and future of the accreditation of medical schools in Korea by the Korean Institute of Medical Education and Evaluation, 2019.

Supplement 2. Audio recording of the abstract.
