# Supplementary material for: Changes in the accreditation standards of medical schools by the Korean Institute of Medical Education and Evaluation from 2000 to 2019
Source: J Educ Eval Health Prof. 2020 Apr 7;17:2. doi: 10.3352/jeehp.2020.17.2 (PMC7214195; doi:10.3352/jeehp.2020.17.2)
Supplement: Supplementary file 1 — Supplement 1. Past, present, and future of the accreditation of medical schools in Korea by the Korean Institute of Medical Education and Evaluation, 2019. [file jeehp-17-02-suppl.pdf]

| 의평원간행물등록번호 |
| --- |
| 201812-RE004-030 |

# 한국의학교육평가원 평가인증기준의 변천과 발전방향

2018

한국의학교육평가원

Korean Institute of Medical Education and Evaluation

이 보고서는 한국의학교육평가원에서 주관하는 연구사업에 의해 수행된 것이며, 이 보고서에 수록된 내용은 연구자 개인적인 의견이며 한국의학교육평가원의 공식 견해가 아님을 밝혀둡니다.

# 제 출 문

**한국의학교육평가원장 귀 하**

이 보고서를 “한국의학교육평가원 평가인증기준의 변천과 발전방향” 과제의 최종보고서로 제출합니다.

2018. 12. 17.


연구책임자 : 박 원 균

연 구 원 : 김 미 경

연 구 원 : 유 효 현

연 구 원 : 윤 유 상

연 구 원 : 이 근 미

연 구 원 : 이 종 훈

연 구 원 : 허 정 식

연 구 원 : 홍 승 재

# 목 차

서문 ········································ iii

제1장 서 론 ········································ 1

1. 필요성 ········································ 1
2. 목적 및 목표 ········································ 1

제2장 내용과 방법 ········································ 3

1. 내용과 방법 ········································ 3
2. 추진 일정 ········································ 3

제3장 결과 ········································ 4

1. 제1주기 의과대학 인정평가기준 ········································ 4
2. 제2주기 의과대학 인정평가기준 ········································ 11
3. Post-2주기 의과대학 평가인증기준 ········································ 18
4. ASK2019 의학교육 평가인증기준 ········································ 31

제4장 제언 및 결론 ········································ 38

참고자료 ········································ 42

부록

1. 역대 인증기준위원장 회고사 ········································ 43
2. 시기별 평가인증기준 ········································ 83

# 요 약 문

## Ⅰ. 제목

한국의학교육평가원 평가인증기준의 변천과 발전방향

## Ⅱ. 연구의 필요성 및 목적

한국의학교육평가원 20주년을 맞아 우리나라에서 실시해온 의학교육 평가인증기준의 변천사를 되돌아보고, 앞으로 우리나라 의학교육 평가인증의 발전을 위한 기틀이 되는 백서를 발간하고자 하였다.

## Ⅲ. 연구내용과 방법

1. 제1주기 인정평가기준부터 제2주기와 Post-2주기를 거쳐 새로 시행할 ASK2019 평가인증까지 평가인증 제도의 시기별 평가인증기준 설정배경과 평가인증기준 항목, 각 평가인증기준으로 평가를 실시한 결과 등이 포함된 각종 자료와 문헌을 분석하여 정리하였다.
2. 연구결과를 토대로 앞으로의 평가인증기준의 개발방향을 영역별로 제안한다.
3. 역대 인증기준위원장의 회고사를 통해 역사적 사료를 남긴다.

## Ⅳ. 연구결과

한국의학교육평가원은 많은 어려운 환경에서도 양질의 평가인증을 위한 기준을 마련하기 위해 노력하여 정량적인 평가인증기준에서 대학의 특성에 알맞은 정성적인 평가인증기준으로 전환함으로써 장래에는 보다 발전적인 의학교육 평가인증이 시행될 것으로 기대한다.

## V. 결론 및 활용방안

지금까지 의학교육 평가인증은 수많은 연구자와 교수자의 헌신을 통해 괄목할만한 발전을 이루어 냈다. 장래 평가인증의 발전을 위해서는 ‘제도적, 문화적 특성을 고려한 평가(institution and culture based assessment)’, ‘미래를 지향하는 평가(future oriented assessment)’, ‘교육적 수월성을 지향하는 평가(excellence and diversity oriented assessment)’, ‘타당도와 신뢰도가 높은 정성적 평가(qualitative assessment)’, ‘소모적이지 않은 실제적 평가(authentic assessment)’가 실현되도록 노력해야 할 것이다.

연구진은 이 연구결과가 우리나라 의학교육의 질적 도약을 위한 기초자료를 제공함으로써 국제적 수준의 평가인증기준의 개발과 의학교육의 수월성을 추구하는데 적합한 교육프로그램 개발에 활용되기를 기대한다.

# 서 문

2019년은 우리나라에서 의학교육 평가인증을 시작한지 20년을 맞이하는 해로, 평가인증체제가 확립되기까지 의학교육에 관계하는 많은 의과대학 교수와 교육전문가들의 끊임없는 노력이 밑받침되었다. 한국의학교육평가원(이하 '의평원')에서 평가인증 10주년을 맞이하여 2009년 발간한 '의학교육 평가인증 10년사'를 제외하고는 우리나라 평가인증을 위한 노력을 알릴 기회가 없었다. 이에 의평원 의학교육인증단 인증기준위원회는 평가인증 20주년을 맞이하여 우리나라에서 실시한 평가인증의 변천사를 돌아보고 앞으로 평가인증에 참여할 교육자에게 길잡이를 제공하여 우리나라 의학교육의 발전을 위한 기틀로 삼고자 이 연구를 시행하였다.

그동안 의평원의 평가인증제도는 우리나라에서 기본의학교육과정을 운영하는 모든 의과대학과 의학전문대학원을 대상으로 실시하였다. 이를 위하여 의평원은 예비평가(1999) 및 제1주기(2000~2005), 제2주기(2007~2011), Post-2주기(2012~2018)를 거쳐 ASK2019(Accreditation Standards of KIMEE 2019) 평가인증(2019~)이라는 평가인증체제의 발전을 이루었다. 이러한 변화는 평가인증에서 가장 중요한 평가인증기준의 발전과 병행되었다. 평가인증기준은 예비평가 및 제1주기 인정평가기준, 제2주기 인정평가기준, Post-2주기 평가인증기준을 각각 개발하여 적용하였고, 이제 WFME(World Federation of Medical Education, 세계의학교육연합회)의 평가인증기준과 동등한 수준의 ASK2019 평가인증기준을 개발하였다.

평가인증 용어는 초기에 '인정평가'로 2008년까지 사용되었고, 법령 개정에 따라 2009년부터 '평가인증'으로 수정하여 현재까지 사용되고 있다.

이 연구는 우리나라 평가인증의 시기별 변천과 진행과정을 파악하는데 필요한 자료들을 모으고 역대 인증기준위원장의 회고와 평가인증기준의 20년에 걸친 자취를 정

리함으로써 앞으로의 평가인증체제 발전과 국제적 수준의 의학교육 평가인증 제도를 추구하는 것을 목적으로 하였다. 연구진은 이 연구결과가 국내 평가인증체제 발전, 국제적 수준의 의학교육을 추구하기 위한 평가인증기준 개발, 의학교육의 수월성을 달성할 교육프로그램 발굴 등을 위한 기초자료로 활용될 것을 기대한다.

2018. 12.

한국의학교육평가원 의학교육인증단 인증기준위원회 일동

# 제1장 서 론

## 1. 필요성

40개 의과대학/의학전문대학(이하 ‘의과대학’, 2018년 현재)이 우리나라 의사의 배출을 책임지고 있다. 따라서 의과대학의 교육프로그램과 교육환경에 대한 평가는 무엇보다 중요하다. 대학마다 특색은 있겠지만 의학교육의 기본적인 틀인 나아갈 방향을 표준화하는 과정은 필요하다. 고등교육법에는 의예과 2년, 의학과 4년(의학전문대학원 4년)으로 명시되어 있고, 의학과와 의학전문대학원의 교육은 일차의료를 담당할 수 있는 의사의 전문역량개발과 강화라는 기본적인 틀에서 매우 중요하다. 또한 우리나라에서 의과대학 졸업생은 독립적으로 진료를 수행할 수 있는 최소한의 기본적인 역량을 갖추어야 한다. 의과대학이 스스로 교육프로그램과 환경에 대하여 평가를 하고 질 관리를 할 수 있도록 의평원이 설립되었다. 의평원은 지금까지 제1주기, 제2주기, Post-2주기, ASK2019 등 시대가 요구하는 의학교육의 평가인증기준을 마련하여 각 의과대학이 급격히 변화하는 의료 환경에 적합한 의료인을 육성할 수 있도록 지원하기 위해 환경변화에 최대한 부응하려는 노력을 경주하였다. 향후 급변하는 의료 환경 속에서 의평원과 각 의과대학의 구성원 및 이해관계자들이 평가인증기준의 변화를 이해할 수 있도록 평가인증 주기마다 평가인증기준을 개발한 배경과 기준 항목에 대한 고찰이 필요하다.

## 2. 목적 및 목표

빠른 의료 환경의 변화로 인해 의학지식의 양이 기하급수적으로 증가하고 있다. 각 의과대학은 학생들이 방대한 양의 의학지식을 효과적으로 학습하도록 자체적인 교육 목표와 성과를 정하고, 다양한 교육방법을 개발하여 시행하고 있다.

대학의 이러한 노력을 뒷받침하기 위하여 졸업생이 적절한 역량을 갖출 수 있도록 기본적인 의학교육시스템에 대해 지속적으로 점검할 필요가 있어, 이를 위해 의평원

은 의학교육시스템을 평가할 수 있는 평가인증기준을 개발하였고 각 시기에 필요로 하는 의학교육의 요구에 따라 개선해 왔다.

평가인증 20주년을 맞이하는 시점에서 의평원은 평가인증기준의 변천과정을 되돌아보고, 앞으로 우리나라 평가인증의 발전을 위한 백서를 발간하고자 하였다.

# 제2장 내용과 방법

## 1. 내용과 방법

제1주기, 제2주기, Post-2주기, ASK2019 평가인증에서 평가인증기준이 개발된 배경과 기준 항목의 변화 등을 의평원에 보관된 각종 자료집과 회의자료를 정리하고 분석하였다. 또한 시기별 평가인증기준의 개발과 실행을 실질적으로 주도했던 역대 인증기준위원회 위원장들의 회고와 자문을 바탕으로 자료를 해석하였다. 이와 함께 연구팀은 Post-2주기 평가인증기준의 관리와 ASK2019 개발의 경험을 바탕으로 앞으로의 발전방향을 도출하였다.

## 2. 추진 일정

| 연구내용 | 추 진 일 정 (개월) | | | | | | 비 고 |
|---|---|---|---|---|---|---|---|
| | 1 | 2 | 3 | 4 | 5 | 6 | |
| 자료수집:<br>시기별 평가인증기준 | ====== | =====〉 | | | | | |
| 자료수집:<br>평가인증기준개발 경험 | | ====== | =====〉 | | | | |
| 자료 분석 및 정렬 | | | ====== | =====〉 | | | |
| 발전방향 도출 | | | | ====== | =====〉 | | |
| 보고서 작성 | | | | | | =====〉 | |

# 제3장 결과

## 1. 제1주기 의과대학 인정평가기준

### 가. 평가인증기준 개발 과정

1998년 9월 한국의과대학인정평가위원회(Accreditation Board for Medical Education in Korea, ABMEK)에서 인정평가를 위한 평가항목개발의 중요성을 인식하고, 이를 위하여 평가기준실무위원회를 별도로 구성하였다. 평가기준실무위원회는 평가항목 및 평가기준개발, 개발을 위한 계획과 전략 수립, 평가결과에 따라 평가항목과 기준을 보완하고 관리함으로써 의학교육의 이념과 목표에 따라 의학교육을 발전시키는 역할을 담당하였다.

평가기준실무위원회는 자체회의, 워크숍, 의과대학 및 의료계의 의견수렴 과정을 거쳐 의과대학 인정평가를 위한 '의과대학 인정평가기준(안)'을 개발하였으며, 한국의과대학인정평가위원회의 최종 인준을 받아 1999년 의과대학 예비평가에 적용하였다. 예비평가 결과의 미비점들을 보완하여 '2000년 의과대학 인정평가기준'이 최종 확정되었다. 구체적인 개발 과정은 다음 〈표 3-1〉과 같다.

〈표 3-1〉 제1주기 인정평가기준 개발

| 연도 | 일자 | 내 용 |
|---|---|---|
| 1998 | 9.24. | 제1차 회의<br>• 평가기준실무위원회의 구성과 한국 의과대학 인정평가제도의 목적과 평가기준실무위원회의 역할 등에 관한 의견교환 |
| | 10.10. | 제2차 회의<br>• 인정평가 항목 및 기준(안) 검토<br>• 정성적 평가지향, 필수항목과 권장항목 구분, 필수항목에 대한 기준과 척도개발 논의 |
| | 10.22. | 제3차 회의<br>• 개발된 각 영역별 평가항목 및 기준의 수정, 보완 |

| 연도 | 일자 | 내 용 |
|---|---|---|
| 1999 | 1.6. | 제4차 회의<br>• 평가항목, 평가기준 및 자체평가보고서 작성지침에 대한 논의 |
| | 4.8. | 제6차 회의 |
| | 5.4. | 제7차 회의 및 워크숍 |
| | 5.26. | • 평가기준 초안에 대한 의과대학 의견수렴 마감 |
| | 6.14. | 제8차 회의 |
| | 7.29. | 제9차 회의<br>• 서면평가 서식 및 서면평가지침(안) 개발 |
| | 8.26. | 제10차 회의<br>• 서면평가를 위한 워크숍 준비를 위한 최종 점검 |
| | 9.3. | 제11차 회의 및 워크숍<br>• 서면평가를 위한 지침개발 워크숍 |
| | 10.13. | 제12차 회의 |
| | 11.17. | 제13차 회의<br>• 방문평가위원 및 대학자체평가위원 대상으로 한 설문지 개발 |
| 2000 | 1.19. | 제14차 회의 및 워크숍<br>• 1999년 인정평가 결과보고서 검토 및 보완을 위한 워크숍 |
| | 3.24. | 제15차 회의 |
| | 10.18. | 회의개최 |
| 2001 | 3.4. | 워크숍<br>• 2000년도 평가를 위해 평가기준과 평가지침의 문제점 개선방안 논의 |

## 나. 평가인증기준 설정 원칙과 방향

### 1) 평가인증기준 설정원칙

의과대학 인정평가를 위한 평가기준을 설정하는데 있어서 대학의 교육·연구·봉사 기능에 대한 체계적 접근을 가능하게 하는 '투입-과정-산출의 환류모형'을 기본으로 하였다. 해당모형에 근거하여 다음의 5개 세부원칙을 평가기준 설정원칙으로 하였다 〈표 3-2〉.

〈표 3-2〉 투입-과정-산출의 환류모형에 근거한 평가기준 설정원칙

| 원 칙 | 주 요 내 용 |
|---|---|
| 유용성의 원칙 | 평가기준은 의학교육의 제공자나 소비자 모두에게 적절한 도움을 줄 수 있는 유용성의 원칙을 바탕으로 설정되어야 한다. 아무리 좋은 평가기준이라 할지라도 평가결과의 효용가치가 없다면 평가기준으로 인정받을 수 없을 것이다. |
| 적절성의 원칙 | 평가기준은 적절성에 입각해서 설정되어야 한다. 평가기준의 내용에 따라서 윤리적으로, 법적으로 그리고 시기에 따라 다양한 견해가 있을 수 있기 때문에 이를 미연에 방지할 수 있도록 기준의 적절성이 마련되어야 한다. 따라서 같은 성과를 기대하는 기준이라면 가능한 효율적으로 평가할 수 있는 기준을 마련하는 것이 바람직할 것이다. |
| 명료성의 원칙 | 설정된 모든 평가기준은 대학과 사회 모두가 이해할 수 있고 인정할 수 있도록 명료하게 표현되어 여러 가지로 해석할 수 있는 가능성을 배제해야 한다. 또한 기준을 증명하는 자료들이 기술적으로 동일할 수 있도록 하여야 한다. |
| 실현 가능성의 원칙 | 평가기준은 허황된 꿈과는 구별이 되어야 한다. 현실적으로 달성 불가능한 기준을 마련하여 평가하는 것은 설득력이 떨어질 수 있다. 따라서 평가기준은 실현가능성에 입각하여 사회의 변화와 각 대학의 여건을 고려할 수 있는 것으로 설정되어야 한다. |
| 미래지향성의 원칙 | 의과대학 인정평가가 교육의 질적 향상과 의과대학의 사회적 책무성 향상에 있다면, 이는 반드시 미래지향적인 기준으로 설정되어야 한다. 평가를 통하여 모든 대학이 어떠한 노력도 없이 달성할 수 있는 기준이라면 인정평가의 목적과는 부합되지 않을 것이다. |

### 2) 평가인증기준의 설정방향

의과대학 인정평가의 평가기준은 우리나라의 현실을 고려한 미래 지향적인 수준을 제시하고, 의학교육의 질적인 향상과 의과대학의 사회적 책무성 수행을 높일 수 있으며, 기초의학 및 임상의학 교육에서 최소한의 교육여건과 교육과정 등을 필수로 확보하도록 하는 방향으로 설정하였다. 구체적인 평가기준의 설정은 다음과 같다.

- 평가영역은 크게 필수영역과 권장영역으로 구분한다.

  필수영역은 모든 의과대학이 의학교육의 책무성을 확보하기 위하여 반드시 갖추

어야 할 기본적인 항목들로, 원칙적으로는 합격/불합격으로 판정한다. 필수영역에서 불합격 판정을 받은 대학에 대하여는 대학의 현실을 고려하여 일정 기간 안에 보완하도록 한다. 권장영역은 미래지향적인 항목들로 구성되며, 가능한 대학 스스로 평가준거에 도달하여 발전할 수 있도록 장려하는 사항이다.

- 한국대학교육협의회의 의학과평가기준과 의학대학설립준칙을 고려한다.
  1997년 범 의료계의 합의로 제정한 의과대학 설립준칙 기준과 한국대학교육협의회에 의해 실시된 1996년 의학과 평가기준을 고려한다. 특히, 필수영역의 시설 및 인력 관련 평가항목은 최소한 의과대학 설립준칙에 부합하여야 한다는 점에서 이를 참고로 한다.

- 정량적 평가기준과 정성적 평가기준의 균형을 고려한다.
  1997년 평가항목은 신뢰성과 객관성을 유지하도록 하여 정량적 평가와 정성적 평가가 고르게 분포되도록 하고, 평가항목에 따라 단기 및 중·장기적 달성 기준을 정한다.

## 다. 의과대학 인정평가기준

한국의과대학인정평가위원회는 평가기준 설정원칙과 설정방향에 근거하여 타당하고 신뢰성을 갖춘 평가기준을 개발하였다. 한국의과대학인정평가위원회의 의과대학 인정평가기준은 기본적으로 1996년 한국대학교육협의회의 93개 의학과 평가기준(대학원 제외)을 바탕으로 개발하였다. 평가영역은 1) 교육목표 및 교육과정, 2) 학생, 3) 교수, 4) 시설·설비, 5) 행정·재정의 5개 영역, 영역별 평가부문 18개, 부문별 필수기준과 권장기준 총 50개 평가기준으로 개발하였다〈표 3-3〉.

〈표 3-3〉 제1주기 의과대학 인정평가 평가영역, 평가부문, 평가문항 수

| 평가영역 | 평가부문 | 평가문항 수 | |
|---|---|---|---|
| | | 필수기준 | 권장기준 |
| 교육목표 및 교육과정 | 목표구성 및 반영노력 | 1 | 2 |
| | 기초의학 교육과정 | 2 | |
| | 임상의학 교육과정 | 2 | 2 |
| | 수업지도와 강의평가 | 2 | 2 |
| | 학생 학습평가 | | 4 |
| | 교육과정 개선 노력 | 1 | 1 |
| | 의학 관련 인성교육과정 과목 개설 | 1 | |
| | 소 계 | 9 | 11 |
| 학생 | 학생지도 체제 | 1 | 1 |
| | 학생복지제도와 시설 | 1 | 2 |
| | 학생 학술연구활동 및 학습성과 | | 2 |
| | 소 계 | 2 | 5 |
| 교수 | 기초 및 임상교수 확보 | 1 | 2 |
| | 교수연구 및 학술활동 | 1 | 2 |
| | 교수개발 | 1 | 2 |
| | 소 계 | 3 | 6 |
| 시설·설비 | 기본교육 및 지원시설 | 3 | 1 |
| | 교수시설 | | 2 |
| | 소 계 | 3 | 3 |
| 행정·재정 | 대학행정 및 운영 체계 | | 3 |
| | 대학재정 | | 2 |
| | 대학발전계획 | 1 | 2 |
| | 소 계 | 1 | 7 |
| | 합 계 | 18 | 32 |

## 라. 평가인증기준의 특징

• 의과대학의 교육여건과 교육과정전체를 평가대상으로 한다.
평가영역별 평가항목의 내용은 의학교육과정이 운영되는 상황과 여건, 바람직한 목표 성취를 위한 가용 자원의 투입정도, 대학의 목적을 구현하기 위해 투입된 요소들 간의 상호작용 과정과 교육·연구·사회봉사 성취 정도를 포함한다.

• 의학교육의 핵심적 요소를 중심으로 평가하였다.
1996년 (사)한국대학교육협의회의 93개 의학과 평가기준에서 50개로 대폭 축소된 것은 평가대상 대학의 부담을 최대한 완화하고, 의학교육의 핵심적 요소만을 평가하며, 평가대상 대학의 특성을 최대한 반영하기 위한 것이다.

• 평가기준을 필수기준과 권장기준으로 구분하였다.
평가기준을 필수기준과 권장기준으로 구분한 것은 평가기준을 적용하는 과정에서 대학을 획일화할 수 있는 단점을 보완하고, 나아가 대학의 특성화를 촉진하는 효과적 장치로 활용하기 위함이다. 더불어 평가기준별 가중치는 부여하지 않았다.

• 의학이라는 특수성을 반영하였다.
의학 관련 인성과목, 학생 연구 활동, 기초와 임상 교육과정의 구분, 기초와 임상 교수의 구분 등은 의학이라는 특수성을 반영하였다.

• 대학발전계획 평가부문을 도입하였다.
의과대학의 발전계획을 평가항목으로 도입함으로써 평가대상 대학들이 장기적인 비전을 가지고 의학교육의 질을 지속적으로 높일 수 있는 계기를 제공하고자 하였다.

## 마. 평가인증기준에 대한 평가

한국의학교육평가원 인증평가사업단은 제1주기 의과대학 인정평가사업에 대한 의과대학 교수들의 인식과 제2주기 의과대학 인증평가사업에 대한 의견을 수렴하기 위하여 2005년 9월 15일부터 9월 30일까지 대학자체평가위원, 현지방문평가위원, 의과대학 인증평가사업단 관계자 등을 대상으로 설문조사를 실시하였고, 총 314명이 응답하였다.

평가기준의 타당성과 관련하여 제1주기 인정평가기준이 의학교육의 질적인 수준을 평가할 수 있는 '타당한 기준으로 구성되었다'고 생각하는 의견은 51.7%, '보통이다'는 42.2%, '타당하지 않다'는 6.3%로 평가기준의 타당성에 대한 인식은 낮은 것으로 나타났다. 인정평가기준이 타당하지 않다고 생각하는 이유는 대학의 여건을 고려하지 않은 획일적인 평가기준, 교육의 질적인 기준을 나타내는 평가지표 부족, 평가기준의 객관성 부족 등이었다.

제1주기 인정평가의 평가결과를 바탕으로 제2주기 인정평가기준의 개발에서는 타당하고 신뢰할 수 있는 평가기준의 개발, 평가항목의 내용과 평가기준 수준향상이 요구되었다. 세계화, 국제화 시대에 우리나라 의과대학들이 경쟁력을 갖기 위해서는 다양한 내용의 교육환경과 프로그램 항목을 평가해야 하며, 최종 인증을 위한 이들 교육환경과 프로그램을 국제적 수준으로 상향조정해 나가야 한다는 발전방향을 제언하였다.

## 2. 제2주기 의과대학 인정평가기준

### 가. 평가인증기준 개발 과정

의학교육인증단 산하 전문위원회의 하나로 평가기준전문위원회를 구성하고, 의과대학 인정평가를 위한 평가항목과 타당하고 신뢰할 수 있는 인정평가기준을 개발하는 역할을 담당하였다.

평가기준전문위원회는 자체 회의, 내·외부 워크숍을 통하여 '제2주기 의과대학 인정평가기준(안)'을 개발하였다. 개발된 인정평가기준(안)은 2005년 2월과 10월에 개최된 공청회를 통해 수정·보완되었고, 해당 기준에 대한 전국 의과대학의 의견수렴을 위하여 한국의과대학장협의회 및 41개 의과대학에 인정평가기준(안)을 발송하였다. 한국의과대학장협의회 전문위원 및 평가기준전문위원회 위원으로 특별위원회를 구성하여 의과대학 인정평가기준(안)에서 쟁점이 되는 사항에 대해 협의·조정하였고, 인정평가기준(안)과 '의학교육데이터베이스(KOMSIS)'를 상호 연계하여 평가기준의 적용과 해석에 대한 신뢰성을 높일 수 있도록 하였다.

또한 정부에서는 고등교육의 국제경쟁력 향상을 위한 대학의 교육·연구수준의 질 보장을 위해 고등교육기관에 대한 평가를 법제화하는 「고등교육법」을 개정(일부개정 2008.3.28, 법률 제8638호)하였고, 「고등교육기관의 평가·인증 등에 관한 규정」(제정 2008.12.17, 대통령령 제21163호)을 마련하였다. 이에 따라 2008년까지 사용되던 '인정평가'라는 용어는 2009년부터 '평가인증' 으로 수정되어 현재까지 사용되고 있다. 이상과 같은 절차를 통해 2006년도에 개발된 '제2주기 의과대학 인정평가기준(안)'은 2007년 3월 최종 확정되었다. 구체적인 평가기준 개발 과정은 다음 〈표 3-4〉와 같다.

〈표 3-4〉 제2주기 인정평가기준 개발

| 연도 | 일자 | 내 용 |
|---|---|---|
| 2004 | 11.30. | 제1차 회의<br>• 제1주기 인정평가 현황과 제2주기 인정평가기준 마련 방향 논의 |
| | 12.9. | 제2차 회의<br>• 제2주기 인정평가기준 개발 계획사업 논의 |
| | 12.29. | 제3차 회의<br>• 제2주기 인정평가기준 개발을 위한 내부워크숍 준비 |
| 2005 | 1.19. | 제4차 회의<br>• 제2주기 인정평가기준 개발 방향 점검<br>• 의과대학 인정평가 교수워크숍 계획 논의 |
| | 1.26. | 제5차 회의<br>• 교육목표 및 교육과정 영역 기준 검토 |
| | 2.16. | 제6차 회의<br>• 의과대학 인정평가 교수워크숍 발표 자료 점검<br>• 제2주기 인정평가기준 위원별 담당부분 기준초안 작성 후 회의하는 진행 방법 변경 |
| | 3.15. | 제7차 회의<br>• 제2주기 인정평가기준 평가기준 1영역 발표 및 토론 |
| | 3.29. | 제8차 회의<br>• 제2주기 인정평가기준 평가기준 2, 4영역 발표 및 토론 |
| | 4.14. | 제9차 회의<br>• 제2주기 인정평가기준 평가기준 3, 5영역 발표 및 토론<br>• 제2차 의과대학 인정평가기준 개발 교수워크숍 관련 사항 논의 |
| | 4.22.-24. | 평가기준전문위원회 내부워크숍<br>• 필수와 권장 기준에 대한 문항과 영역구성 검토 작업 |
| | 5.11. | 제10차 회의<br>• 제2주기 인정평가기준 지침서 관련 사항 논의<br>• 제2주기 인정평가기준 평가부문별 기준 추가 논의 |
| | 5.17. | 제11차 회의 및 내부워크숍<br>• 제2차 의과대학 인정평가기준 개발 교수워크숍 최종 확인<br>• 평가부문별 담당위원 발표 및 확인 |

| 연도 | 일자 | 내 용 |
|---|---|---|
| | 5.20. | 제12차 회의 및 내부워크숍<br>• 필수와 권장 기준에 대한 문항과 영역 검토 작업 |
| | 6.21. | 제13차 회의<br>• 제2차 의과대학 인정평가기준 개발 교수워크숍 분임토의 결과 논의 |
| | 8.16. | 제14차 회의<br>• 제2주기 인정평가기준별 데이터베이스 출력 양식(안) 검토<br>• 대학원영역(GM)에 대한 평가기준 개발 |
| | 8.23. | 제15차 회의<br>• 제2주기 인정평가기준별 데이터베이스 출력 양식(안)'영역별 검토 |
| | 9.23. | 제16차 회의<br>• 제2주기 인정평가기준별 데이터베이스 출력 양식(안)' 수정보완<br>• 제2주기 인정평가기준에 대한 최종 수정안 REVIEWER 선정 |
| | 10.22. | 제17차 회의<br>• 제2주기 인정평가기준 최종 수정안 REVIEW결과 논의<br>• 일부 평가영역 기준 추가 검토 |
| | 10.31. | 제18차 회의<br>• 제2주기 인정평가기준 최종 수정안 논의 |
| | 11.3. | 제2주기 인정평가기준 공청회 |
| | 11.29. | 제19차 회의<br>• 공청회에서 제시된 의견 논의 |
| | 12.14. | 제20차 회의<br>• 제2주기 인정평가기준(안) 검토 |
| 2006 | 1.4. | 제21차 회의<br>• 제2주기 인정평가기준(안) 최종 검토 |
| | 1.18. | 제22차 회의<br>• 제2주기 인정평가기준(안)과 WFME 평가기준 비교<br>• 의과대학 인정평가 출력폼(안) 확인 작업 |
| | 2.1. | 제23차 회의<br>• 제2주기 인정평가기준(안) 수정 |
| | 2.8. | 제24차 회의<br>• 제2주기 인정평가기준(안) 신설항목 논의 및 수정<br>• 5.영역: 의과대학 대학부속병원 관련 법규, 겸무교원에 관한 법규 확인 |

| 연도 | 일자 | 내 용 |
| --- | --- | --- |
| | 3.13. | 제25차 회의<br>• 제2주기 인정평가기준 관련 문의사항 점검<br>• 제2주기 인정평가기준 평가대학 지침서<br>• 제2주기 인정평가기준 평가자 지침서 |
| | 3.30. | 제26차 회의<br>• 학장협 제2주기 인정평가기준 의견검토 확인 |
| | 4.20. | 제27차 회의<br>• 제2주기 의과대학 인정평가 진행 관련 논의<br>• 학장협 제2주기 인정평가기준 의견검토 관련 논의 |
| | 6.1. | 제28차 회의<br>• 학장협 제2주기 인정평가기준 의견검토 관련 논의 및 수정 |
| | 9.26. | 제29차 회의<br>• 제2주기 의과대학 인정평가기준(부록 포함) 최종본 검토 |
| | 10.20. | 제30차 회의<br>• 제2주기 의과대학 인정평가기준 최종본 평가부문별 재검토 |
| | 11.29. | 제31차 회의<br>• 학장협 제2주기 인정평가기준 수정요청 사항 검토 |
| | 12.27. | 제32차 회의<br>• 제2주기 인정평가기준 및 규정집 최종본 평가부문별 재검토 |
| 2007 | 1.18. | 제33차 회의<br>• 제2주기 의과대학 인정평가기준 영역별 평가지침 작성검토 |
| | 2.28. | 제34차 회의<br>• 제2주기 의과대학 인정평가기준 영역별 평가지침 작성검토 |
| | 3.16.-17. | 제35차 회의<br>• 제2주기 의과대학 인정평가기준 영역별 평가지침 검토 작업 |

## 나. 평가인증기준 설정 방향 및 원칙

의평원은 제1주기 의과대학 인정평가사업에 대한 평가결과에서 지적된 인정평가기준의 문제점과 제2주기 인정평가기준이 지향해야 할 방향에 대한 의견을 수렴하였고, 2005년 제2주기 의과대학 인정평가기준 개발 초기부터 타당성 확보를 위하여 다양한 노력을 하였다.

제2주기 의과대학 인정평가기준은 평가대상 의과대학의 사회적 책무성 수행을 강화하고, 각 대학들이 교육의 수월성을 추구하기 위해 노력해야 한다는 관점에서 개발되었다. 또한 국내 평가 대상 의과대학의 의학교육이 국제적 수준에 도달할 수 있고, 의학교육의 질적, 양적 특성을 균형 있게 평가할 수 있도록 하였다. 따라서 제2주기 의과대학 인정평가기준 설정방향은 다음과 같다.

- 대학의 책무성 정도에 따라 필수, 권장 및 우수기준으로 구분한다.
  의학교육기관의 기본적인 책무성 수행과 관련된 사항들은 필수로 규정하였으며, 미래지향적인 관점에서 교육의 질적인 향상과 관련된 사항들은 권장으로 규정하였다. 또한, 교육의 수월성 추구와 관련된 사항들을 우수기준으로 설정하였다.

- 세계적 수준의 의학교육 질 향상을 위하여 국제적 수준에 부합하도록 한다.
  세계의학교육연합회(WFME), 국제의학교육기구(IIME), 미국의학교육합동위원회(LCME), 영국의학협회(GMC) 등의 의학교육 평가기준을 상호 비교하여 우리나라 인정평가기준이 국제적 수준을 유지하도록 하였다.

- 정량적 평가기준과 정성적 평가기준이 균형 잡히도록 한다.
  의학교육기관의 책무성 수행을 객관적으로 측정·평가할 수 있는 평가항목은 정량적 기준으로 개발하고, 전문가의 전문성에 의해 주관적으로 판단될 수 있는 평가항목은 정성적 기준으로 개발하였다.

## 다. 평가인증기준

제2주기 의과대학 인정평가기준은 6개의 평가영역(대학운영체계, 교육목표와 교육과정, 학생, 교수, 시설·설비, 졸업후교육)과 21개 평가부문을 가지고 있다〈표 3-5〉. 평가기준은 필수기준 41개, 권장기준 34개, 우수기준 34개, 총 109개 문항으로 구성되어 있다. 필수기준은 모든 의과대학이 반드시 도달해야 하는 기준이며, 권장기준은 모든 의과대학이 도달하기를 제안하는 수준이다. 또한 우수기준은 의학교육의 수월성을 추구하기 위해 제시되는 기준을 의미한다.

〈표 3-5〉 제2주기 의과대학 인정평가기준 영역, 부문, 문항 수

| 평가영역 | 평가부문 | 문항 수 | 평가문항 수 | | |
|---|---|---|---|---|---|
| | | | 필수 | 권장 | 우수 |
| 1. 대학운영 체계 | 1-1 대학의 설립 | 3 | 3 | 0 | 0 |
| | 1-2 대학행정 및 운영 체계 | 4 | 4 | 0 | 1 |
| | 1-3 대학 행정 | 3 | 1 | 2 | 0 |
| | 1-4 대학발전계획 | 3 | 1 | 2 | 0 |
| | 1-5 개선노력 | 3 | 2 | 1 | 1 |
| | 소계 | 16 | 11 | 5 | 2 |
| 2. 교육목표와 교육과정 | 2-1 교육목표와 교육과정의 기본 틀 | 5 | 2 | 3 | 2 |
| | 2-2 기초의학 교육과정 | 3 | 3 | 0 | 0 |
| | 2-3 임상의학 교육과정 | 8 | 6 | 2 | 2 |
| | 2-4 인문·사회의학 교육과정 | 3 | 1 | 2 | 1 |
| | 2-5 수업방법과 강좌평가 | 3 | 3 | 0 | 2 |
| | 2-6 학생 학습평가 | 3 | 2 | 1 | 2 |
| | 소계 | 25 | 17 | 8 | 9 |
| 3. 학생 | 3-1 입학정책 및 학생선발 | 2 | 0 | 2 | 0 |
| | 3-2 학생지도체계 | 3 | 1 | 2 | 2 |
| | 3-3 학생복지 제도 및 시설 | 5 | 2 | 3 | 3 |
| | 3-4 졸업 후 진로 및 학습 성과 | 2 | 0 | 2 | 1 |
| | 소계 | 12 | 3 | 9 | 6 |
| 4. 교수 | 4-1 기초 및 임상 전임교수 | 5 | 2 | 3 | 4 |
| | 4-2 교수의 연구 및 학술활동 | 5 | 1 | 4 | 4 |
| | 4-3 교수개발 실태 | 3 | 3 | 0 | 3 |
| | 소계 | 13 | 6 | 7 | 11 |
| 5. 시설·설비 | 5-1 교육 시설·설비 | 4 | 3 | 4 | 4 |
| | 5-2 연구 관련 시설·설비 | 2 | 1 | 4 | 1 |
| | 소계 | 6 | 4 | 2 | 5 |
| 6. 졸업후교육 | 6-1 대학원 교육 | 3 | 0 | 3 | 1 |
| | 소계 | 3 | 0 | 3 | 1 |
| 합계 | | 75 | 41 | 34 | 34 |

## 라. 평가인증기준의 특징

- 평가인증기준을 필수기준, 권장기준, 우수기준으로 구분하였다.
  필수기준은 모든 의과대학이 반드시 도달해야 하는 기준, 권장기준은 모든 의과대학이 도달하기를 제안하는 수준의 기준, 우수기준은 의학교육의 수월성을 추구하기 위해 제시되는 기준을 의미한다. 우수기준을 추가함으로써 평가항목 혹은 영역별 평가결과의 의미가 커졌다.

- 균형 잡힌 정량 평가기준과 정성 평가기준으로 구성하였다.
  제1주기에서는 각 의과대학의 기본 교육인프라를 일정 수준 이상으로 개선, 향상시킬 필요가 있다는 인식하에 정량적 평가기준을 상당 부분 도입하였다. 그러나 제2주기 평가에서는 정량적, 지표적 기준들의 비중을 낮춘 반면 의학교육의 실제적 내용과 충실도를 판단하는 정성적 평가기준의 비중을 높였다.

- 졸업후교육 평가부문을 도입하였다.
  대학병원의 대학구조화와 대학원 정상화를 위한 평가기준을 포함하였다.

- 성인교육이론에 기초하였다.
  성인교육이론에 기초한 교육방법, 유급제도 등의 개선을 지속적으로 추진하도록 하였다.

## 마. 평가인증기준에 대한 평가

의평원 인증평가사업단은 제2주기 의과대학 인정평가사업에 대한 메타평가 중 제2주기 인정평가기준이 평가인증기준으로서 어느 정도 중요한지, 이 기준이 현재 우리나라 의과대학에서 시행되는데 어느 정도 적절한지를 의학교육전문가를 대상으로 포커스 집단조사를 실시하였다. 그 결과 모든 평가기준 중에서 몇 개의 기준을 제외한 대부분이 중요하고 적절한 것으로 나타났다.

중요도가 낮은 기준으로는 대학운영체계 영역에서 1개, 교육목표와 교육과정 영역

에서 1개가 있었다. 대학운영체계 영역은 동문과 지역사회에 대한 기준이었으며, 교육목표와 교육과정 영역은 보완대체의학 및 통합의학 내용이었다.

적절성의 경우에는 5개 영역에서 9개 평가기준이 적절성 점수가 낮았다. 교육과정 영역에서는 중요성과 마찬가지로 보완대체의학 및 통합의학, 학생 영역에서는 임상의학 이외의 타 분야 진출현황, 교수 영역에서는 기초 및 임상의 교수 구성, 시설·설비 영역에서는 대학부속병원 확보, 정보화시스템 운영, 교수실 확보 등이었으며, 졸업후교육 영역에서는 대학원 수업계획서 적절성 등이 다른 평가기준에 비해서 낮은 점수를 받았다.

결론적으로 메타평가 결과 제2주기 인정평가기준은 타당성이 높은 평가기준이라고 평가되었다. 각 영역에서 일부 중요성과 타당성이 낮게 나타난 기준들은 Post-2주기 평가인증기준 개발에 적용할 것을 제안하였다. 양적 기준으로 측정하지 못하는 질적인 측면에서의 부족함을 계속적으로 찾아내고, 각 의과대학의 특성화 및 수월성을 추구하며, 다양성을 존중할 수 있는 평가기준 개발에 지속적으로 노력해야할 것을 제언하였다.

## 3. Post-2주기 의과대학 평가인증기준

### 가. 평가인증기준 개발 과정

#### 1) 교육부 인정기관 지정 이전 평가인증기준 개발

의평원 의학교육인증단의 인증기준위원회에서는 Post-2주기 평가인증기준 개발을 위해 2010년부터 10회 이상의 회의와 워크숍을 거쳐 평가인증기준 마련 작업을 진행하였고, 2회의 공청회를 통해 의견을 수렴하여 최종 Post-2주기 평가인증기준을 2010년 11월 30일 의평원 심포지엄을 통해 발표하였다. 2011년 1월 실행위원회에서 Post-2주기 평가인증기준을 최종 승인하였고, 2012년부터 적용하였다〈표 3-6〉.

〈표 3-6〉 Post-2주기 평가인증기준 개발(2011)

| 연도 | 일자 | 내용 |
|---|---|---|
| 2010 | 3.23. | 제1차 회의<br>• 2010년 사업계획 추진 |
| | 4.16. | 제2차 회의<br>• Post-2주기 평가인증기준 및 Post-2주기 평가인증(안) 논의 |
| | 4.30. | 제3차 회의<br>• Post-2주기 평가인증기준 및 Post-2주기 평가인증(안) 논의 |
| | 5.13. | 제4차 회의<br>• Post-2주기 평가인증기준 및 Post-2주기 평가인증(안) 논의 |
| | 6.4. | 제5차 회의<br>• Post-2주기 평가인증기준 및 Post-2주기 평가인증(안) 논의 |
| | 7.1.-3. | 제6차 회의(전문위원 집중워크숍)<br>• 기준 및 규정 및 Post-2주기 평가인증 합동작업 |
| | 7.16. | 제7차 회의<br>• Post-2주기 평가인증기준 및 Post-2주기 평가인증(안) 논의 |
| | 7.30. | 제8차 회의<br>• Post-2주기 평가인증기준 및 Post-2주기 평가인증(안) 논의 |
| | 8.21. | 제10차 회의<br>• Post-2주기 평가인증기준 및 Post-2주기 평가인증(안) 논의 |
| | 8.31. | 제2차 인증전문위원 집중워크숍<br>• 기준 및 규정 및 Post-2주기 평가인증 합동작업 |
| | 9.13. | 제1차 평가인증기준 및 Post-2주기 평가인증을 위한 공청회 개최 |
| | 11.8. | 제11차 회의<br>• Post-2주기 평가인증기준 검토의견 논의 |
| | 11.15. | 제2차 평가인증기준 및 Post-2주기 평가인증을 위한 공청회 개최 |
| | 11.30. | 의평원 2010년 심포지엄 - Post-2주기 의과대학 평가인증 |
| 2011 | 1.24. | 실행위원회 Post-2주기 평가인증기준 최종 승인 |

*이후 지속적으로 기준과 가이드를 수정 보완함.

### 2) 교육부 인정기관 지정 이후 평가인증기준 개발

고등교육법에 의거하여 교육부장관이 지정한 인정기관으로 하여금 학부·학과·전공 및 학문 분야를 포함한 학교의 교육과정운영 등을 평가 및 인증하는 정부의 교육프로그램 평가·인증 인정기관 지정제도는 고등교육의 질적 향상 및 체계적 관리를 위해 2008년 도입되었다. 의평원은 2010년 11월 교육부에 인정기관 지정을 신청해 평가·인증의 합목적성, 인프라, 기준 및 방법, 실적 및 활용 등의 항목에 대해 심사받고, 2014년 5월 12일 인정을 받아 의학교육 평가인증기관으로서의 역할을 수행하게 되었다.

정부 인정기관으로 지정받기 위한 과정에서 평가인증기준에 대하여 정량적 평가기준을 정성적 평가기준으로 전환할 것을 요구받아 2013년 11월 29일 제4-005차 회의에서 기본기준 총 97개 중 정량적 평가기준 36개를 검토하였다. 36개의 정량적 평가기준 중 대학운영체계 2개, 기본의학교육과정 5개, 학생 2개, 교수 14개, 시설·설비 3개, 졸업후교육 1개 등 총 27개 기준을 정성적으로 수정하였고, 정량적 평가기준 9개는 교육과정 운영과 평가, 임상실습(실습기간, 방법 등)에 관한 내용으로서 구체적인 지침이 필요한 기준으로 판단하여 유지하기로 하였다. 수정 전·후의 Post-2주기 평가인증기준 비교표는 다음과 같다〈표 3-7〉.

〈표 3-7〉 Post-2주기 평가인증기준 수정 전·후 비교표

| | 수정 전 | 수정 후 |
|---|---|---|
| 대학운영체계 | **1-2-1 대학 운영에 필요한 업무가 적절하게 분장되어 있는가?**<br>[기본기준] 교육, 교수, 학생 업무가 행정적 연관성을 가지고 분장되어 있으며, 3개 분야에서 별도의 보직자가 임명되어 있다. | **1-2-1 대학 운영에 필요한 업무가 적절하게 분장되어 있는가?**<br>[기본기준] 교육, 교수, 학생 업무가 행정적 연관성을 가지고 분장되어 있으며, 별도의 보직자가 임명되어 있다. |
| 대학운영체계 | **1-5-3 외국 의과대학과의 교류는 적절한가?**<br>[기본기준] 국제협력을 위한 전담 기구가 있고 예산과 지원인력이 있다. 국외 연구, 교육, 실습 등의 학생 교류는 최소 2주 이상 참여하는 학생이 입학정원 기준 5% 이상이다. | **1-5-3 외국 의과대학과의 교류는 적절한가?**<br>[기본기준] 국제협력을 위한 전담 기구가 있고 예산과 지원인력이 있다. 국외 연구, 교육, 실습 등의 학생 교류는 적절히 운영되고 있다. |

| | 수정 전 | 수정 후 |
|---|---|---|
| 기본의학교육과정 | **2-2-1 교육목표는 구성원들에게 잘 인지되어 있으며, 대학은 교육목표 인지도를 높이기 위해 적절한 노력을 하고 있는가?**<br>**[기본기준]** 교육목표에 대한 대학 구성원의 인지도가 60% 이상이고, 대학은 이를 높이기 위해 다양한 노력을 하고 있다. 교육목표에 대한 대학 구성원의 인지도 조사는 최소 2년에 한 번 시행되고 있다. | **2-2-1 교육목표는 구성원들에게 잘 인지되어 있으며, 대학은 교육목표 인지도를 높이기 위해 적절한 노력을 하고 있는가?**<br>**[기본기준]** 교육목표에 대해 대학 구성원이 잘 인지하고 있으며, 대학은 이를 높이기 위해 다양한 노력을 하고 있다. 교육목표에 대한 대학 구성원의 인지도 조사를 주기적으로 시행하고 있다. |
| | **2-2-3 교육과정위원회는 교육과정 개발, 관리 및 평가 등을 위한 예산을 확보하고 적절하게 집행하고 있는가?**<br>**[기본기준]** 교육과정위원회의 연간 예산이 3천만 원 이상이다. | **2-2-3 교육과정위원회는 교육과정 개발, 관리 및 평가 등을 위한 예산을 확보하고 적절하게 집행하고 있는가?**<br>**[기본기준]** 교육과정위원회의 연간 예산이 적절하다. |
| | **2-2-5 학생교육관련 직접비용에 대한 지원은 적절한가?**<br>**[기본기준]** 학생교육 관련 직접비용이 연간 학생 1인당 등록금 대비 5%이상이다. | **2-2-5 학생교육관련 직접비용에 대한 지원은 적절한가?**<br>**[기본기준]** 연간 학생 1인당 등록금 대비 학생교육 관련 직접비용이 적절하다. |
| | **2-3-13 학생이 자유롭게 선택할 수 있는 임상실습 과정이 있는가?**<br>**[기본기준]** 학생이 자유롭게 선택할 수 있는 임상실습 과정이 최소한 2주 이상 편성되어 있다. | **2-3-13 학생이 자유롭게 선택할 수 있는 임상실습 과정이 있는가?**<br>**[기본기준]** 학생이 자유롭게 선택할 수 있는 임상실습 과정이 적절히 편성되어 있다. |
| | **2-3-16 의료인문학 과정 학습성과에 도달하기 위한 교육방법과 평가방법은 적절한가?**<br>**[기본기준]** 의료인문학 전체과정의 1/3 이상에서 단순 강의식 방법 이외의 다양한 방법이 시행되고 있다. 학습평가 방법도 단순 지필시험 이외의 다양한 방법이 1/3 이상이다. | **2-3-16 의료인문학 과정 학습성과에 도달하기 위한 교육방법과 평가방법은 적절한가?**<br>**[기본기준]** 의료인문학 전체과정에서 다양한 교육방법과 평가방법이 적용되고 있다. |

| | 수정 전 | 수정 후 |
|---|---|---|
| 학생 | **3-3-1 대학은 공정하게 운영하는 장학제도가 있으며, 지급 비율은 적절한가?**<br>**[기본기준]** 대학은 공정하게 운영하는 장학제도가 있으며, 등록금 대비 장학금 지급비율이 연 평균 10% 이상이다. | **3-3-1 대학은 공정하게 운영하는 장학제도가 있으며, 지급 비율은 적절한가?**<br>**[기본기준]** 대학은 공정하게 운영하는 장학제도가 있으며, 등록금 대비 장학금 지급비율이 적절하다. |
| 학생 | **3-4-1 임상의학 이외 분야의 진출을 위한 대학의 지원은 적절한가?**<br>**[기본기준]** 임상의학 이외 분야의 전문가를 확보하기 위한 정책(장학금 및 특별지원금 등)과 실적이 있거나, 최근 10년간 연간 입학정원의 1% 이상의 졸업생이 임상의학 이외의 분야로 진출하고 있다. | **3-4-1 임상의학 이외 분야의 진출을 위한 대학의 지원은 적절한가?**<br>**[기본기준]** 임상의학 이외 분야의 전문가를 확보하기 위한 적절한 정책(장학금 및 특별지원금 등)이 있거나, 졸업생이 임상의학 이외의 분야로 진출한 실적이 적절하다. |
| 교수 | **4-1-1 기초의학 전임교수가 적정 수 확보되어 있는가?**<br>**[기본기준]** 모든 의과대학은 세계의학교육협회가 권고하는 기초의학 교육을 위해 각 분야 (13개 분야)에 최소 1명 이상, 총 25명의 기초의학 교원을 확보하고 있다. | **4-1-1 기초의학 전임교수를 적절하게 확보하고 있는가?**<br>**[기본기준]** 모든 의과대학은 세계의학교육협회가 권고하는 기초의학 교육을 위해 각 분야별로 기초의학 교원을 적절하게 확보하고 있다. |
| 교수 | **4-1-2 의학교육학 전임교수가 적정 수 확보되어 있는가?**<br>**[기본기준]** 의학교육학교실(학과, 센터 등)이 개설되어 있으며, 의학교육 전임교수가 1인 이상 있다. | **4-1-2 의학교육학 전임교수를 적절하게 확보하고 있는가?**<br>**[기본기준]** 의학교육학교실(학과, 센터 등)이 개설되어 있으며, 의학교육 전임교수가 있다. |
| 교수 | **4-1-3 의료인문학 전임교수가 적정 수 확보되어 있는가?**<br>**[기본기준]** 의료인문학 분야 전임교수 1인 또는 전담교수 3인 이상을 확보하고 있다. | **4-1-3 의료인문학 전임교수를 적절하게 확보하고 있는가?**<br>**[기본기준]** 의료인문학 분야에 전임교수 또는 전담교수를 적절하게 확보하고 있다. |
| 교수 | **4-1-4 기초의학, 의학교육학, 의료인문학의 교육과 연구를 보조하는 인력은 적절한가?**<br>**[기본기준]** 기초의학, 의학교육학, 의료인문학의 교육과 연구를 보조하는 인력으로 대학에서 직접적으로 재정적 지원을 하는 조교, 연구원이 분야별로 전임교수 2인당 1명 이상이다. | **4-1-4 기초의학, 의학교육학, 의료인문학의 교육과 연구를 보조하는 인력은 적절한가?**<br>**[기본기준]** 기초의학, 의학교육학, 의료인문학의 교육과 연구를 보조하는 인력으로 대학에서 직접적으로 재정적 지원을 하는 조교, 연구원이 분야별로 적절하다. |

| | 수정 전 | 수정 후 |
|---|---|---|
| | **4-1-5 임상의학 전임교수가 적정 수 확보되어 있는가?**<br>**[기본기준]** 20개 이상 진료과와 지원과목에 전임교수가 총 85명 이상 있다. | **4-1-5 임상의학 전임교수를 적절하게 확보하고 있는가?**<br>**[기본기준]** 각 임상의학 전공과목별로 전임교수를 적절하게 확보하고 있다. |
| | **4-1-6 기초의학, 의학교육학, 의료인문학, 임상의학 전임교수의 구성은 적절한가?**<br>**[기본기준]** 의과대학 전체 교원 중 동일 대학 출신 비율 이 70% 이하이거나, 또는 최근 6년 이내에 임용된 신임교원 중 동일 대학 출신 비율이 70% 이하이다. | **4-1-6 기초의학, 의학교육학, 의료인문학, 임상의학 전임교수의 구성은 적절한가?**<br>**[기본기준]** 의과대학 전체 교원은 동일 대학 출신으로 편중되어 있지 않다. |
| | **4-2-1 교수들의 국내·외 연구실적이 적절한가?**<br>**[기본기준]** 전임 교수 100명당 국내·외 연구실적이 최근 2년 동안 연평균 100편 이상이다. | **4-2-1 교수들의 국내·외 연구실적이 적절한가?**<br>**[기본기준]** 전임 교수 국내·외 연구실적이 적절하다. |
| | **4-2-2 대학 외부로부터 받은 연구비 수준은 적절한가?**<br>**[기본기준]** 전임 교수들의 최근 2년간 연평균 외부 연구비 수혜 비율이 전체 교수의 10% 이상 이거나, 전임교수 1인당 연평균 수혜 연구비가 5백만 원 이상이다. | **4-2-2 대학 외부로부터 받은 연구비 수준은 적절한가?**<br>**[기본기준]** 전임 교수들의 최근 2년간 연평균 외부 연구비 수준이 적절하다. |
| | **4-2-3 대학 내부 연구비 수준은 적절한가?**<br>**[기본기준]** 전임교수들이 최근 2년 동안 대학 내부로부터 받은 연구비가 전임교수 1인 당 연평균 일백만 원 이상이다. | **4-2-3 대학 내부 연구비 수준은 적절한가?**<br>**[기본기준]** 전임교수들이 최근 2년 동안 대학 내부로부터 받은 연구비 수준이 적절하다. |
| | **4-3-2 교수업적평가 기준에 의학교육과 교수개발에 관련된 내용을 포함하고 있으며, 의학교육 관련 연수교육이나 자체 교수개발 프로그램의 참여 정도가 적절한가?**<br>**[기본기준]** 교수업적평가 규정에 전임교수들에게 의학교육과 관련된 연수교육이나 교수개발 프로그램에 의무적으 | **4-3-2 교수업적평가 기준에 의학교육과 교수개발에 관련된 내용을 포함하고 있으며, 의학교육 관련 연수교육이나 자체 교수개발 프로그램의 참여 정도가 적절한가?**<br>**[기본기준]** 교수업적평가 규정에 전임교수들에게 의학교육과 관련된 연수교육이나 교수개발 프로그램에 의무적으 |

| | 수정 전 | 수정 후 |
|---|---|---|
| | 로 참여해야 하는 연간 교육시간이 명시되어 있으며 의학교육 관련 연수교육에 최근 2년 동안 연간 3시간 이상 참석한 교수 비율이 50% 이상이다. | 로 참여해야 하는 연간 교육시간이 명시되어 있으며 의학교육 관련 연수교육에 적절하게 참석하고 있다. |
| | **4-3-3 의과대학 신임교원을 위한 의학교육 연수과정이 적절하게 시행되고 있는가?**<br>**[기본기준]** 의과대학 신임교원을 위한 의학교육 연수과정이 의무적으로 시행되고 있으며, 교원 발령 후 1년 이내에 최소 15 시간 이상 이수하고 있다. | **4-3-3 의과대학 신임교원을 위한 의학교육 연수과정이 적절하게 시행되고 있는가?**<br>**[기본기준]** 의과대학 신임교원을 위한 의학교육 연수과정이 의무적으로 적절하게 시행되고 있다. |
| | **4-3-5 교수들의 장·단기 외국 연수와 국내외 학회 참석을 위한 재정적 지원체제를 적절히 갖추고 있는가?**<br>**[기본기준]** 교수들의 장·단기 외국연수와 국내외 학회 참석을 위한 재정적 지원이 있어야 하며 최근 2년 동안 연평균 교수 1인당 2백만 원 이상 지급한다. | **4-3-5 교수들의 장·단기 외국 연수와 국내외 학회 참석을 위한 재정적 지원체제를 적절히 갖추고 있는가?**<br>**[기본기준]** 교수들의 장·단기 외국연수와 국내외 학회 참석을 위한 재정적 지원이 적절하다. |
| | **4-3-6 연구윤리에 관한 기본 교육이 시행되고 있는가?**<br>**[기본기준]** 연구윤리에 대한 의과대학의 자체 규정이 마련되어 있으며 연 1회 이상 정기적으로 연구윤리교육이 시행되고 있다. | **4-3-6 연구윤리에 관한 기본 교육이 시행되고 있는가?**<br>**[기본기준]** 연구윤리에 대한 의과대학의 자체 규정이 마련되어 있으며, 정기적으로 연구윤리교육이 시행되고 있다. |
| | **4-3-7 성희롱 예방교육이 시행되고 있는가?**<br>**[기본기준]** 성희롱 예방교육에 대한 대학의 자체 업무규정이 있으며 연 1회 이상 정기적으로 교육이 시행되고 있다. | **4-3-7 성희롱 예방교육이 시행되고 있는가?**<br>**[기본기준]** 성희롱 예방교육에 대한 대학의 자체 업무규정이 있으며, 정기적으로 교육이 시행되고 있다. |
| **시설설비** | **5-1-4 대학은 학생의 교육과 복지를 위한 시설·설비를 효율적으로 관리하고 있는가?**<br>**[기본기준]** 학생의 교육과 복지를 위한 시설·설비의 관리 인력이 1인 이상 있고, 적절한 예산이 배정되어야 한다. | **5-1-4 대학은 학생의 교육과 복지를 위한 시설·설비를 효율적으로 관리하고 있는가?**<br>**[기본기준]** 학생의 교육과 복지를 위한 시설·설비의 관리 인력이 있고, 적절한 예산이 배정되어야 한다. |

| | 수정 전 | 수정 후 |
|---|---|---|
| | **5-1-5 대학은 임상실습 교육을 위하여 대학부속 교육병원을 확보하고 있으며, 병원 내에 학생교육 시설을 갖추고 있는가?**<br>**[기본기준]** 대학은 임상실습 교육을 위하여 유효병상 500병상 이상의 대학부속 교육병원을 확보하고 있다. 병원 내에는 각종 교육시설을 갖추고 있으며, 학생 20명 당 최소 1개의 학생 전용공간이 확보되어 있다. | **5-1-5 대학은 임상실습 교육을 위하여 대학부속 교육병원을 확보하고 있으며, 병원 내에 학생교육 시설을 갖추고 있는가?**<br>**[기본기준]** 대학은 임상실습 교육을 위하여 유효병상 500병상 이상의 대학부속 교육병원을 확보하고 있다. 병원 내에는 각종 교육시설을 갖추고 있으며, 적절한 학생 전용공간이 확보되어 있다. |
| | **5-2-1 대학은 개인 교수실과 그 실내 설비를 확보하고, 행정지원 체계를 갖추고 있는가?**<br>**[기본기준]** 전임강사 이상 교원의 개인 교수실 확보 수준이 80%이상이며, 적절한 실내 설비를 갖추고 있다. | **5-2-1 대학은 개인 교수실과 그 실내 설비를 확보하고, 행정지원 체계를 갖추고 있는가?**<br>**[기본기준]** 대부분의 교수가 개인 교수실을 확보하고 있다. |
| 졸업후교육 | **6-1-3 학생선발과 학생지원체제는 적절한가?**<br>**[기본기준]** 특성화 된 대학원 편제에 합당한 다양한 경력을 소유한 학생을 선발하고 있으며, 등록금 총액 대비 10% 이상의 교내·외 장학금 또는 특별지원금이 있다. | **6-1-3 학생선발과 학생지원체제는 적절한가?**<br>**[기본기준]** 특성화 된 대학원 편제에 합당한 다양한 경력을 소유한 학생을 선발하고 있으며, 등록금 총액 대비 교내·외 장학금 또는 특별지원금이 적절하다. |

## 나. 평가인증기준 설정 방향 및 원칙

의평원의 인증제도위원회에서는 평가인증제도 개선 작업을 통하여 다음 평가주기부터는 2년마다 자체평가연구보고서를 의무적으로 제출하도록 함으로써 주기의 개념을 없애기 위해서 새로운 평가인증을 제3주기라고 하지 않고, Post-2주기로 명명하였다. 2012년부터 시행한 Post-2주기 평가인증기준은 제2주기 인정평가기준의 부족함을 인지하고 많은 부분을 보완하고자 노력하였다. 인증기준위원회에서 Post-2주기의 새로운 평가인증기준을 개발하는데 있어 다음과 같은 설정원칙에 근거하였다.

- 전국 의과대학 기본교육의 질 향상을 위해서 어느 수준 이상의 교육환경과 교육프로그램에 대한 표준화 기준을 개발한다(standardization).
- 세계화와 국제화에 따른 의학교육의 선진화를 위하여 국내 수준의 표준화를 넘어서 국제적 기준 추세에 맞추는 평가인증기준을 개발한다(global standard).
- 각 의과대학에서 수행하고 있는 의학교육의 특성화 및 수월성을 추구하면서 다양성을 존중할 수 있는 평가인증기준을 개발한다(excellence and diversity).
- 장기적이고 미래 지향적인 평가인증기준을 개발한다(future-oriented criteria).
- 교육의 질 평가를 위한 정성적 기준을 강화하도록 개발한다(qualitative criteria).
- 사회가 바라는 의사, 즉 인성교육을 강화할 수 있는 평가인증기준을 개발한다(medical humanity and professionalism).
- 성과바탕교육을 위한 평가인증기준을 개발한다(Outcome-Based education).
- 교육부 '고등교육 평가인증 인정기관' 지정에 대비하기 위한 대학 기본의학교육, 졸업후교육 및 평생교육에 관한 기준 등도 고려하여 개발한다.

## 다. 평가인증기준

### 1) 교육부 인정기관 지정 이전 평가인증기준

Post-2주기 평가인증기준은 총 6개 영역으로, 평가부문은 1영역 5개, 2영역 5개, 3영역 4개, 4영역 3개, 5영역 2개, 6영역 1개로 구성되어 있으며, 평가기준은 기본기준 97개, 우수기준 43개, 총 140개이다〈표 3-8〉.

〈표 3-8〉 Post-2주기 평가인증기준 영역, 부문, 기준 수(2010)

| 평가영역 | 평가부문 | 문항 수 | 평가기준 수 | |
|---|---|---|---|---|
| | | | 기본 | 우수 |
| 1. 대학운영체계 | 1-1 대학설립 | 3 | 3 | 0 |
| | 1-2 대학행정 및 운영 체계 | 6 | 6 | 2 |
| | 1-3 대학재정 | 3 | 3 | 0 |
| | 1-4 대학발전 계획 | 3 | 3 | 0 |
| | 1-5 개선노력 | 3 | 3 | 2 |
| | 소계 | 18 | 18 | 4 |
| 2. 기본의학교육과정 | 2-1 교육과정 개요 | 3 | 3 | 0 |
| | 2-2 교육과정 개발과 지원 | 5 | 5 | 3 |
| | 2-3 교육과정 구성과 운영 | 16 | 16 | 3 |
| | 2-4 학업성취 평가 | 3 | 3 | 3 |
| | 2-5 교육과정 평가와 개선 | 3 | 3 | 0 |
| | 소계 | 30 | 30 | 9 |
| 3. 학생 | 3-1 입학정책과 학생선발 | 4 | 4 | 1 |
| | 3-2 학생지도 체제 | 6 | 6 | 5 |
| | 3-3 학생복지와 안전 | 7 | 7 | 6 |
| | 3-4 졸업 후 진로 | 2 | 2 | 1 |
| | 소계 | 19 | 19 | 13 |
| 4. 교수 | 4-1 전임교수 | 6 | 6 | 6 |
| | 4-2 교수업무 | 5 | 5 | 3 |
| | 4-3 교수개발 | 7 | 7 | 3 |
| | 소계 | 18 | 18 | 12 |
| 5. 시설·설비 | 5-1 교육 시설·설비 | 7 | 7 | 3 |
| | 5-2 연구 시설·설비 | 2 | 2 | 1 |
| | 소계 | 9 | 9 | 4 |
| 6. 졸업후교육 | 6-1 대학원 교육 | 3 | 3 | 1 |
| | 소계 | 3 | 3 | 1 |
| 합계 | | 97 | 97 | 43 |

### 2) 교육부 인정기관 지정 이후 평가인증기준 개발

교육부 인정기관 지정 이후에는 인정기관지정 이전과 6개 평가영역, 20개 평가부문, 97개 평가문항은 동일하지만, 정량적 평가기준이 정성적 평가기준으로 수정되었다. 또한 2영역의 2-3 평가부문에 우수기준으로 '2-3-14 임상실습 책임교수가 지정되어 있고, 학생에 대한 지도감독과 피드백이 적절한가?'로 Resident-As-Teacher 내용을 추가하였다〈표 3-9〉.

〈표 3-9〉 Post-2주기 평가인증기준 영역, 부문, 기준 수(2014)

| 평가영역 | 평가부문 | 문항 수 | 평가기준 수 | |
|---|---|---|---|---|
| | | | 기본 | 우수 |
| 1. 대학운영체계 | 1-1 대학설립 | 3 | 3 | 0 |
| | 1-2 대학행정 및 운영 체계 | 6 | 6 | 2 |
| | 1-3 대학재정 | 3 | 3 | 0 |
| | 1-4 대학발전 계획 | 3 | 3 | 0 |
| | 1-5 개선노력 | 3 | 3 | 2 |
| | 소계 | 18 | 18 | 4 |
| 2. 기본의학교육과정 | 2-1 교육과정 개요 | 3 | 3 | 0 |
| | 2-2 교육과정 개발과 지원 | 5 | 5 | 3 |
| | 2-3 교육과정 구성과 운영 | 16 | 16 | 4 |
| | 2-4 학업성취 평가 | 3 | 3 | 3 |
| | 2-5 교육과정 평가와 개선 | 3 | 3 | 0 |
| | 소계 | 30 | 30 | 10 |
| 3. 학생 | 3-1 입학정책과 학생선발 | 4 | 4 | 1 |
| | 3-2 학생지도 체제 | 6 | 6 | 5 |
| | 3-3 학생복지와 안전 | 7 | 7 | 6 |
| | 3-4 졸업 후 진로 | 2 | 2 | 1 |
| | 소계 | 19 | 19 | 13 |

| 평가영역 | 평가부문 | 문항 수 | 평가기준 수 | |
|---|---|---|---|---|
| | | | 기본 | 우수 |
| 4. 교수 | 4-1 전임교수 | 6 | 6 | 6 |
| | 4-2 교수업무 | 5 | 5 | 3 |
| | 4-3 교수개발 | 7 | 7 | 3 |
| | 소계 | 18 | 18 | 12 |
| 5. 시설·설비 | 5-1 교육 시설·설비 | 7 | 7 | 3 |
| | 5-2 연구 시설·설비 | 2 | 2 | 1 |
| | 소계 | 9 | 9 | 4 |
| 6. 졸업후교육 | 6-1 대학원 교육 | 3 | 3 | 1 |
| | 소계 | 3 | 3 | 1 |
| 합계 | | 97 | 97 | 44 |

## 라. 평가인증기준의 특징

- 학습성과(Learning Outcome)중심의 교육을 적용하였다.
  학습성과바탕의 교육과정은 학생이 의과대학 졸업 시에 의사로서 일정 수준 이상의 역량을 보여줄 수 있는 자질 배양에 초점을 둔 것으로 평가인증기준에 반영되었다. 이는 의학교육에서 학생이 수행할 수 있는 자질 함양을 위한 역량중심 교육과정이 강조되고, 학습성과를 기반으로 하는 교육과정이 도입되는 국제적 추세를 반영한 것이다.

- 정성적 평가기준을 강화하였다.
  전문가 집단에 의한 정성적 평가가 단순하고 기계적인 정량적 평가보다 더 정확하다는 것은 입증된 사실이다. 특히 의학교육 평가인증은 서열화나 당락을 가르기 위한 평가가 아닌 의과대학에서의 교육의 환경과 프로그램의 수월성을 제고하며 이를 통해 학생들이 양질의 교육을 받을 권리를 보호하고, 더 나아가 진료를 받을 환자와 국민의 건강권을 담보하기 위한 평가이므로 전문가에 의한 정성적 평가가 단순한 정량적 평가보다 평가인증의 목적에 훨씬 부합하기 때문에 정

성적 평가기준을 강화하였다.

- 기본기준과 우수기준으로 구분하였다.
  제2주기 인정평가기준의 ‘필수기준’과 ‘권장기준’을 합쳐서 ‘기본기준(basic standard)’으로 정하였다. 이는 필수기준이라는 명칭이 ‘최소한 그 평가기준은 달성해야 하고 기준에 미달하면 인증이 불가한 절대적 기준’이라는 오해의 발생 소지를 고려한 조치였다. 기본기준은 모든 의과대학이 의학교육의 타당성을 확보하기 위하여 반드시 갖추어야 하는 기본항목이다.

- 의과대학의 교육과정개발에 활용할 수 있도록 기준을 구성하였다.
  교육목표와 교육과정 평가영역은 ‘기본의학교육과정’으로 수정하고 교육과정의 개발, 운영, 평가의 순으로 교육과정 개발절차에 따라 순서대로 정리함으로써 대학에서 교육과정개발 시에 쉽게 접근할 수 있도록 하였다.

- 학생 영역은 학생의 보호와 권익보장 측면을 강화하였다.

- 졸업후교육 영역은 기본적인 내용만을 다루었다.
  향후 전공의교육과 평생교육을 의과대학 평가에서 함께 고려해야 할 부분이므로 기본적인 내용만을 평가인증기준으로 하였다.

## 마. 평가인증기준에 대한 평가

Post-2주기 평가인증 후반에 실시한 메타평가에서 평가인증에 참여한 의과대학을 대상으로, 평가인증의 기본기준과 우수기준이 의과대학의 발전에 도움이 되었는지 설문하였다. 기본기준에 대해서는 각 대학 자체평가연구위원의 77.8%, 우수기준에 대해서는 52.6%가 대학 발전에 도움이 되었다고 응답하였다. 두 기준 모두 긍정응답이 50%를 상회하고 있으나, 기본기준에 대한 긍정응답비율이 우수기준에 비해 25.2% 높았다. 평가인증기준이 어느 정도 도움이 되었는지에 대한 질문에서는 우수기준보다는 기본기준에 더 도움이 된 것으로 나타났다.

# 4. ASK2019(Accreditation Standards of KIMEE 2019) 의학교육 평가인증기준

## 가. 평가인증기준 개발 과정

세계화의 흐름에 따라 고등교육에 대한 평가인증의 국가 간 연계 필요성이 증대하고 있고, 국내 학위의 국제 인증 및 외국 학위의 국내 인증을 위하여 국제적 수준에 상응하는 대학평가기준의 개발이 요구되는 상황이었다. 이러한 국제적 추세에 부응하여 의료와 관련 인력의 국제화를 촉진하고 의료행위의 최소 수준을 보장하여 의료인력의 국제적 활용도를 높이기 위하여 세계의학교육연합회(WFME)를 중심으로 의학교육의 국제표준화가 추진되었다. 우리나라도 가시화되고 있는 의료시장의 개방을 앞두고 국내 의과대학에 대한 국제적 인정의 필요성이 더욱 높아지고 있기 때문에 우리나라 의과대학 평가인증제도가 국제사회의 인정을 받을 수 있도록 체계적으로 준비하고 실행하는 것이 필요하다는 인식을 바탕으로 ASK2019 평가인증을 준비하였다.

의평원 의학교육인증단은 WFME이 인정하는 인증기관 신청을 위하여 2013년부터 전문가 회의, 워크숍 등을 실시하였고, WFME 방문평가단의 평가를 거쳐 2016년 9월 평가인증기관으로 WFME의 인정받았다. 이와 함께 WFME 평가기준을 근거로 우리나라 의학교육 상황에 적합한 평가인증기준을 마련하기 위하여 다수의 회의와 집중작업, 워크숍 등을 통해 ASK 평가기준(안)을 개발하였다. 2015년 1회, 2016년 2회 등 총 3회 공청회를 통해 의견수렴을 하였고, 한국의과대학·의학전문대학원협회, 대한기초의학협의회, 한국의학교육학회 등 새 평가인증기준 관련 유관기관들과의 간담회를 통해 수많은 수정과 정리 과정을 거쳐 ASK2018을 개발하였다. 이후 의학교육인증단 TFT회의를 개최하여 최종 정리하는 과정을 거쳤고, 2017년도 5월에 2019년부터 전면 시행할 것을 확정함에 따라 ASK2019로 명명하여 공표하였다. 내·외부 워크숍과 다수의 회의를 통해 ASK2019 적용을 위한 가이드라인을 개발하여 2018년 2월 의학교육 평가인증 설명회를 통해 발표하였다〈표 3-11〉.

〈표 3-11〉 ASK2019 개발

| 연도 | 일자 | 내용 |
|---|---|---|
| 2013 | 3.26. | 제1차 회의<br>• WFME 평가기준 도입을 위한 준비 작업 |
| | 4.22. | 제2차 회의<br>• WFME 평가기준과 Post-2주기 평가인증기준 비교 후 통합하는 방안 논의 |
| | 5.3. | 의평원 합동워크숍<br>• 2018년 WFME 평가기준 도입을 위한 연구 계획 |
| | 7.5. | 2013년 제1차 내부워크숍<br>• WFME 영역별 평가기준 번역을 검토하고 일부 내용 수정 |
| | 7.26. | 제3차 회의<br>• WFME 영역별 평가기준 번역을 검토하고 일부 내용 수정 |
| | 8.30. | 제4차 회의<br>• WFME 영역별 평가기준 번역을 검토하고 일부 내용 수정 |
| | 9.27.-28. | 2013년 제2차 내부워크숍<br>• WFME 영역별 평가기준 번역을 검토하고 일부 내용 수정 |
| | 11.15. | 제3차 교육부 인증기관 인정 및 WFME 인증기관 인정 관련 집중작업 개최 |
| | 11.29. | 제5차 회의<br>• Post-2주기 평가인증기준 중 정량적 기준 수정(안) 검토<br>• WFME 영역별 평가기준 번역을 재검토 |
| | 12.20.-21. | 제4차 교육부 인증기관 인정 및 WFME 인증기관 인정 관련 집중작업 |
| | 12.27. | 제6차 회의<br>• WFME 평가기준과 Post-2주기 평가인증기준을 함께 검토하여 추가할 내용이나 누락된 내용이 있는지 확인 |
| 2014 | 1.23. | 제5차 교육부 인증기관 인정 및 WFME 인증기관 인정 관련 집중작업 |
| | 11.25. | 제9차 회의<br>• WFME 평가기준 번역자료 전문가 검토결과 확인 |
| 2015 | 4.18.-19. | 2015년 제1차 내부워크숍<br>• WFME 평가기준 한글번역본 검토 |

| 연도 | 일자 | 내용 |
|---|---|---|
| | 7.10.-11. | 2015년 제2차 내부워크숍<br>• WFME 평가기준 한글번역본을 완성 및 평가기준에 따른 가이드라인 추가 |
| | 9.5. | 2015년 제4차 내부워크숍<br>• WFME 평가기준 가이드라인에 대한 수정작업<br>WFME와 KIMEE 평가기준을 비교분석하며, WFME 평가기준의 대항목, 소항목, 문항 수를 기본바탕으로 하여 KIMEE 평가기준을 매칭하고, 추가 또는 대체하는 작업을 진행 |
| | 10.22. | 제12차 회의<br>• 2015년 WFME 평가기준 설명회 준비 |
| | 11.14.-15. | 2015년 제5차 내부워크숍<br>• 의학교육 평가인증기준 개정 설명회 준비<br>• 평가기준개정에 대한 설정방향과 개정원칙 작성 |
| | 11.24. | 2015년 제1차 의학교육 평가인증기준 개정(안) 공청회 |
| | 12.5. | 2015년 제6차 내부워크숍<br>• WFME 평가기준(2015년 version) 검토 및 새 평가기준에 대한 수정·보완 건 |
| 2016 | 1.22.-23. | 2016년 제1차 내부워크숍<br>• WFME 평가기준 및 가이드라인 검토 |
| | 5.11. | 이화여대 국어문화원 교정 및 윤문작업 |
| | 6.14. | 2016년 제2차 의학교육 평가인증기준 개정(안) 공청회 개최<br>제2차 회의<br>• 의학교육 평가인증기준 개정(안) 가이드 작업 방향설정 |
| | 8.26. | 제3차 회의<br>• 의학교육 평가인증기준 개정(안) 검토 및 가이드라인 수정 건 |
| | 9.30.-10.1. | 평가인증 선진화 방안 논의를 위한 의평원 위원 워크숍 |
| | 10.28. | 제6차 회의<br>• 평가인증 선진화 방안 논의를 위한 의평원 위원 워크숍에서 받은 피드백에 따라 의학교육 평가인증기준 개정(안)을 재검토 |
| | 11.25. | 제7차 회의<br>• 평가인증 선진화 방안 논의를 위한 의평원 위원 워크숍에서 받은 피드백에 따라 의학교육 평가인증기준 개정(안)을 재검토<br>• 2016년 제2차 의학교육 평가인증기준 개정(안) 공청회 준비 |

| 연도 | 일자 | 내용 |
|---|---|---|
| | 12.2. | 2016년 제2차 의학교육 평가인증기준 개정(안) 공청회 |
| | 12.9. | 기초의학협의회 교육위원회, 의학교육 평가인증 개정(안)에 대한 검토의견 접수 |
| | 12.14. | 제8차 회의<br>• 의학교육 평가인증기준 개정(안)을 '평가인증기준 2018(ASK 2018)'로 명명<br>• 공청회에서 제기된 의견과 기초의학교육협의회에서 보낸 의견서를 검토하고 이에 따라 평가기준을 일부 수정함. |
| 2017 | 2.2. | 평가인증기준 개정(안) 관련 기초의학협의회 미팅 |
| | 2.8. | 실행위원회<br>• ASK2018 심의 |
| | 2.17. | 제9차 회의<br>• 의학교육 평가인증기준 개정(안) 검토 및 가이드라인 수정 |
| | 4.6. | 새 평가인증기준 관련 유관기관 간담회(의평원, 한국의과대학·의학전문대학원협회, 대한기초의학협의회, 한국의학교육학회) |
| | 4.28. | 제11차 회의<br>• 유관기관 간담회 등을 통해 최종 수정·보완된 새 평가인증 기준이 2019년 전면 시행으로 확정되어 ASK2019로 명명 |
| | 5.18. | 제12차 회의<br>• 새 의학교육 평가인증기준 'ASK2019' 공개 |
| | 8.25. | 제13차 회의<br>• ASK2019 가이드라인 작업 |
| | 9.22. | 제14차 회의<br>• ASK2019 가이드라인 작업 |
| | 10.19. | 제15차 회의<br>• 유한 의학교육 평가 컨퍼런스 워크숍 준비(참석자 의견 수렴)<br>• ASK2019 가이드라인 작업-'자체평가보고서 작성 가이드'마련 |
| | 11.24. | 제16차 회의<br>• ASK2019 가이드라인 작업-ASK2019 가이드라인 검토 및 논의<br>• ASK2019 도입을 위한 워크숍 준비 |
| | 12.1.-2. | ASK2019 도입을 위한 의평원 합동워크숍 |

| 연도 | 일자 | 내용 |
|---|---|---|
| | 12.20. | 제17차 회의<br>• 2018년 의학교육 평가인증 설명회 준비<br>• ASK2019 가이드라인 작업-1, 8영역 |
| 2018 | 1.5. | 제18차 회의<br>• ASK2019 가이드라인 작업-3, 6, 7, 9영역 |
| | 2.14. | ASK2019 기준 및 가이드 시행 |
| | 2.23. | 2018년도 의학교육 평가인증 설명회 |

*이후 지속적으로 기준과 가이드를 수정 보완함.

## 나. 평가인증기준 설정 방향 및 원칙

의평원은 기본의학교육의 질적 향상을 목적으로 WFME에서 제시한 Basic Medical Education WFME Global Standards for Quality Improvement(The 2015 Revision)를 근간으로 우리나라의 기본의학교육 상황을 고려하여 2018년부터 사용할 의학교육 평가인증 기준을 개정하였다.

- 전체적인 구조와 구성은 WFME Global Standards와 동일하게 개정한다.
  기본기준은 K (Korea basic standard), 우수기준은 H (High quality development standards)로 표현한다. 기본기준은 의과대학과 기본의학교육이 충족해야 하는 기준으로 인증의 목적에 해당한다. 우수기준은 의과대학과 기본의학교육의 바람직한 미래지향적인 기준으로, 국제적으로 합의된 모범 사례에 따르도록 하는 의학교육 개혁이 목적이다.

- 평가기준은 우리나라 의학교육 상황에 적절하도록 개정한다.
  1) WFME 기준과 Post-2주기 평가인증기준 간의 균형을 유지한다.
  2) WFME 기준 중 우리나라 의학교육 상황에 맞지 않는 것은 사용하지 않는다.
  3) WFME 기준 중 일부는 우리나라 의학교육 상황에 맞게 Post-2주기 평가인증기준에 맞추어 추가 또는 수정한다.
  4) Post-2주기 평가인증기준 중 기본의학교육과정이 아닌 것은 포함하지 않는다.

5) 우리나라에서 법적으로 규정되어 있는 것은 평가인증기준에 포함하지 않는다.

## 다. 평가인증기준

ASK2019는 총 9개의 영역으로 구성하였다. 1영역 사명과 성과는 4개 평가부문, 2영역 교육과정은 8개 평가부문, 3영역 학생평가는 2개 평가부문, 4영역 학생은 4개 평가부문, 5영역 교수는 2개 평가부문, 6영역 교육자원은 6개 평가부문, 7영역 교육평가는 4개 평가부문, 8영역 대학운영체계와 행정은 5개 평가부문, 9영역 지속적 개선은 1개의 평가부문으로 구성하였다. 평가인증기준 수는 기본기준 92개, 우수기준 51개, 총 143개이다〈표 3-12〉.

〈표 3-12〉 ASK2019 영역, 부문, 기준 수(2018)

| 평가영역 | 평가부문 | 평가인증기준 수 | | |
|---|---|---|---|---|
| | | 기본기준 | 우수기준 | 합계 |
| 1. 사명과 성과 | 1.1 사명 | 3 | 1 | 4 |
| | 1.2 대학의 자율성과 학문의 자유 | 1 | - | 1 |
| | 1.3 교육성과 | 3 | 1 | 4 |
| | 1.4 사명과 교육성과 수립 | 1 | 1 | 2 |
| | 소계 | 8 | 3 | 11 |
| 2. 교육과정 | 2.1 교육과정 | 3 | 1 | 4 |
| | 2.2 과학적 방법 | 3 | - | 3 |
| | 2.3 기초의학 | 2 | 1 | 3 |
| | 2.4 의료인문학 | 1 | 1 | 2 |
| | 2.5 임상의학과 술기 | 4 | 3 | 7 |
| | 2.6 교육과정의 구조, 구성, 기간 | 2 | 2 | 4 |
| | 2.7 교육과정 관리 | 2 | - | 2 |
| | 2.8 의료행위와 보건의료분야의 연계 | 1 | 1 | 2 |
| | 소계 | 18 | 9 | 27 |
| 3. 학생평가 | 3.1 평가방법 | 4 | 1 | 5 |
| | 3.2 평가와 학습의 관계 | 4 | 2 | 6 |
| | 소계 | 8 | 3 | 11 |

| 평가영역 | 평가부문 | 평가인증기준 수 | | |
|---|---|---|---|---|
| | | 기본기준 | 우수기준 | 합계 |
| 4. 학생 | 4.1 입학정책과 선발 | 1 | 3 | 4 |
| | 4.2 입학정원 | 1 | – | 1 |
| | 4.3 학생상담과 지원 | 6 | 3 | 9 |
| | 4.4 학생대표 | 2 | – | 2 |
| | 소계 | 10 | 6 | 16 |
| 5. 교수 | 5.1 채용과 선발정책 | 6 | 1 | 7 |
| | 5.2 교수활동과 개발 정책 | 6 | 1 | 7 |
| | 소계 | 12 | 2 | 14 |
| 6. 교육자원 | 6.1 시설 | 8 | 1 | 9 |
| | 6.2 임상실습 자원 | 3 | 1 | 4 |
| | 6.3 정보기술 | 1 | 2 | 3 |
| | 6.4 의학연구와 의과학자 양성 | 3 | 1 | 4 |
| | 6.5 교육 전문성 | 2 | 3 | 5 |
| | 6.6 교육적 교류 | 1 | 1 | 2 |
| | 소계 | 18 | 9 | 27 |
| 7. 교육평가 | 7.1 교육모니터링과 평가에 대한 체제 | 3 | 1 | 4 |
| | 7.2 교육자와 학생의 피드백 | 1 | 1 | 2 |
| | 7.3 학생과 졸업생의 수행 능력 | 1 | 1 | 2 |
| | 7.4 이해관계자의 참여 | 1 | – | 1 |
| | 소계 | 6 | 3 | 9 |
| 8. 대학운영체계와 행정 | 8.1 대학운영체계 | 4 | 2 | 6 |
| | 8.2 학장과 보직자 | 1 | 1 | 2 |
| | 8.3 교육예산과 자원 할당 | 2 | – | 2 |
| | 8.4 행정직원과 관리 | 1 | 1 | 2 |
| | 8.5 보건의료분야와의 상호작용 | 1 | 1 | 2 |
| | 소계 | 9 | 5 | 14 |
| 9. 지속적 개선 | 9.0 지속적 개선 | 3 | 11 | 14 |
| | 소계 | 3 | 11 | 14 |
| 합계 | 36 | 92 | 51 | 143 |

# 제4장 제언 및 결론

## 가. ASK2019 영역별 제언

사명과 성과 영역은 사명, 대학의 자율성과 학문의 자유, 교육성과, 사명과 교육성과 수립으로 구성되어 있다. 대학은 설립이념을 가지고 있으며, 설립이념을 기반으로 하여 대학구성원, 즉 학생, 교수, 의과대학 보직자, 본부 보직자, 보건의료 관계자, 지역사회의 의사회 등의 적극적인 의견수렴을 통하여 사명을 정해야 한다. 사명에는 의사상을 포함할 수 있다. 졸업성과의 경우 사명이 개발되기 전에 구성이 된 경우, 사명을 토대로 졸업성과와 의도한 교육성과에 대하여 신중한 재검토가 필요하다.

교육과정 영역은 교육과정 개요, 과학적 방법, 기초의학, 의료인문학, 임상의학과 술기, 교육과정의 구조, 구성, 기간, 교육과정 관리, 의료행위와 보건의료분야의 연계 등 총 8개 평가부문으로 구성되어 있다. Post-2주기 평가인증기준 기본의학교육과정 영역의 5개 평가부문 중 교육과정의 개요, 개발과 지원, 구성과 운영 부문을 보다 체계적이고 지속적인 교육과정이 운영될 수 있도록 세분화한 것이 특징이다. 또 ASK2019 교육과정 영역의 특징은 교수-학습방법에서 교육자중심의 학습을 학습자중심의 학습으로 개선한 것이다. 즉 의과대학은 자기주도 학습이 가능한 학습자중심의 교육과정을 운영하고, 교육자는 체계적인 시스템을 구축해서 이를 지원하는 것을 궁극적인 목표로 하고 있다. 각 교육과정은 사명과 성과에 근거하고 있으며, 현재와 미래의 의료 환경 변화에 맞춰 지속적으로 수정·보완할 것을 요구하고 있다. 기본의학교육과정이 졸업후교육과 평생학습이 가능한 교육과정으로 연계되고, 지역사회의 의견이나 요구가 반영되는 교육과정을 운영하는 것은 과거와 차별화된 교육과정이라고 할 수 있다. 이는 일차의료를 수행할 수 있는 실력과 인격을 갖춘 의사를 양성하는 프로그램에 교수, 학생, 직원, 동문, 학부모, 보건의료분야 이해관계자가 모두 참여해야 함을 의미한다. 이런 점에서 향후 교육과정의 지속적 개선과 질적 향상의 책임은 비단 교육자와 교육기관만의 몫이 아닌, 사회구성원 모두의 몫이 될 것이다.

학생평가 영역은 평가방법, 평가와 학습의 관계 총 2개 평가부문으로 구성되어 있다. Post-2주기 평가인증기준 기본의학교육과정 영역의 평가부문 중 학업성취도 평가부문을 보다 구체적이고 체계적인 평가기준으로 구성함으로써 학생평가의 질적 향상을 이룬 점이 특징이다. 학생평가의 원칙을 세우고, 그 원칙에 따라 실제 학생평가를 실행함으로써 학생평가를 제도적으로 운영할 것을 제안하고 있고, 학생평가를 지식, 술기, 태도 영역을 다양한 평가방법을 사용하여 기존의 지필 위주의 평가에서 벗어나 교육성과에 근거한 학생평가를 하도록 하고 있다. 또한 성과바탕교육에 근거하여 학생이 의도한 교육성과에 도달했는지 여부와 학습을 증진시킬 수 있는 목적의 학생평가를 강조하고 있다. 즉 교육성과에 적합한 체계성, 연계성, 타당성, 신뢰성에 근거한 학생평가가 필요하다.

학생 영역은 Post-2주기 평가인증기준에서 입학정책과 학생선발, 학생지도체제, 학생복지와 안전, 졸업 후 진로로 구성되었던 평가부문이 ASK2019에서는 입학정책과 선발, 입학정원, 학생상담과 지원, 학생대표로 변화하였다. 평가인증기준은 크게 변화하지 않았으나, Post-2주기 평가인증기준에서 우수기준이었던 유급률 분석을 통한 학업 지도, 유급 전 학생에게 소명의 기회부여, 학생대표의 위원회 참여 등이 기본기준으로 변경되었다. 이는 의과대학에서 유급생과 휴학생을 포함한 학업 지도의 중요성을 반영하고, 의과대학 전반적인 부분에서 학생의 의견수렴을 강조한 것으로 일방적이고 수동적인 교육에서 학생들의 능동적 참여를 강조하고 있다.

교수 영역은 채용과 선발정책, 교수활동과 개발정책의 두 가지 평가부문으로 구성되어 있다. Post-2주기 평가인증기준에서 전임교수, 교수업무, 교수개발의 3개 평가부문이었던 것이 축약되었다. Post-2주기에 강조되었던 국내외 연구실적과 내·외부 연구비 수혜실적, 부설연구소 활동 등의 항목이 제외되었다. 이는 연구업적이 대학평가에서 이미 중요한 부분을 차지하고 있고, 대학 차원에서 교수의 채용과 승진 등에 주요 항목으로 반영되고 있기 때문이다. ASK2019에서는 오히려 교수활동과 개발을 위한 대학차원에서의 지원이 이루어지고 있는지를 평가하고 있으며, 교수업적평가에서도 교육, 연구, 봉사 기능 간의 균형을 강조하고 있다. 또한 분야별 교수진의 구성이 전체 교육과정 운영에 적절하게 균형이 잡혀있는지, 교수 모두가 전체 교육과정을 숙지하도록 지원하는 정책이 있는지, 이를 바탕으로 통합을 위해 교수를

지원하고 있는지 평가하고 있다. 결론적으로 ASK2019에서는 교수채용과 교수활동의 적절성을 강조하고 있으며, 책무와 기능에 따른 교수채용정책과 교수업적평가를 요구하고 있다.

교육자원 영역은 시설, 임상실습자원, 정보기술, 의학연구와 의과학자 양성, 교육전문성, 교육적 교류의 6개 평가부문으로 구성되어 있다. Post-2주기 평가인증기준의 교육시설·설비, 연구시설·설비 평가부문이 6개의 평가부문으로 세분화되었다. 교육자원의 주요 특징은 학생중심으로 전환된 기준으로 볼 수 있다. 학생을 위한 임상실습자원, 학생을 위한 정보기술, 학생을 위한 의학연구, 학생교육을 위한 교육전문성, 학생교육을 위한 교류라는 관점에서 평가인증기준을 제시하고 있다. 6개의 평가부문 중에서도 주목할 만한 부분은 '교육전문성'이다. 이 부문은 Post-2주기 평가인증기준과 달리 새롭게 추가 보완되었다. 교육전문성이라 함은 의학교육의 과정, 실행, 문제점을 다루고, 이에 대한 연구경험이 있는 의사, 교육학자, 사회학자, 기관들을 지칭하고 있다. 이 기준을 반대로 생각해보면 의학교육의 전문성이 부족한 상태에서 교육과정이 설계되고, 실행될 때, 그 문제점을 발견하고 수정해 나가는 과정이란 매우 어려운 일이라는 것을 뜻한다. 최근 의학교육의 흐름은 교육의 과정 전체에 대하여 매우 많은 변화를 요구하고 있다. 새로운 교육과정에 대한 개발, 개선, 변화에 대한 요구는 피할 수 없기에, 이에 따른 제반 문제들을 해결해 나가기 위해서는 전략이 필요하다. 이는 교육전문성을 확보하는 것이며, 시대의 흐름에 부합한 당연한 선택일 것이다.

교육평가 영역에서는 ASK2019 평가인증의 기본방향인 교육 관련 활동의 지속적 개선을 위해 교육과정에 국한되지 않고 교육의 과정으로 확장한 평가를 강조하고 있다. 즉 교육과정을 포함하여 학생이 입학하는 시점부터 졸업할 때까지 이루어지는 교육과 관련된 모든 교육의 과정에 대한 계획 수립, 시행, 자료의 수집, 분석, 피드백을 통하여 개선해 나가는 체계적인 교육평가에 대한 기준으로 추가 보완된 영역이다. 따라서 Post-2주기 평가인증기준이 교육과정 평가에 중점을 두었다면 ASK2019의 교육평가 영역은 교육과정을 포함한 교육자원, 교수, 교직원, 학생, 의과대학의 문화, 학습환경 등 교육과 관련된 모든 활동에 대한 정기적인 모니터링과 평가를 체계적으로 시행하여 일회성이 아닌 지속적 개선을 위한 종합적 평가를 요구하고 있다.

대학운영체계와 행정 영역은 대학운영체계, 학장과 보직자, 교육예산과 자원할당, 행정직원과 관리, 보건의료분야와의 상호작용 등 5개 평가부문으로 구성되어 있다. 이 평가영역은 Post-2주기 평가인증기준과 유사한 부분을 고려하여 학장단이 변경되더라도 체계적으로 모든 학사, 교무, 예산, 집행 등이 실행될 수 있게 행정체계를 갖출 것을 요구하고 있다.

## 나. 결론

평가가 모든 것을 결정한다. 그러나 모든 평가는 본질적으로 한계를 갖는다. 평가는 모든 것을 결정할 정도로 중요하지만, 동시에 완벽한 평가란 존재할 수 없다. 어떤 형태의 평가가 도입되더라도 평가는 본질적 한계성을 가진다는 점을 인지하는 것이 필요하다.

지금까지 의학교육 발전의 역사는 평가인증 발전 역사와 궤를 같이 하고 있다고 해도 과언이 아닐 것이다. 의평원의 평가인증은 그동안 수많은 연구자와 교수자의 헌신으로 괄목할 만한 성과를 이루었다. 이러한 성과를 기반으로 평가인증기준 개선 후속작업은 다음의 내용을 고려할 것을 제안한다.

- 제도적, 문화적 특성을 고려한 평가(institution and culture based assessment)
- 미래를 지향하는 평가(future oriented assessment)
- 교육적 수월성을 지향하는 평가(excellence and diversity oriented assessment)
- 타당도와 신뢰도가 높은 정성적 평가(qualitative assessment))
- 소모적이지 않은 실제적 평가(authentic assessment)

## 참고자료


1. 한국의학교육평가원(2009), 한국의학교육평가원 설립 5주년 기념 의학교육 평가인증 10년사(1999-2009).
2. 안덕선, 제1기 기준위원회 회의록(2004.9.-2007.8.).
3. 김영창, 제2기 기준위원회 회의록(2007.9.-2010.2.).
4. 이정애, 제3기 기준위원회 회의록(2010.3.-2013.2.).
5. 윤희상, 제4기 기준위원회 회의록(2013.3.-2016.2.).
6. 김명곤, 박원균, 제5기 기준위원회 회의록(2016.3.-2018.12.)
7. 교육부 평가인정기관 인증신청자료(2010.12.28., 2011.1.19., 2012.1022, 2013.10.15., 2014.1.28.).
8. 한국의학교육평가원(2011), 의과대학인증평가의 메타평가(Post-2주기).

# 부록1: 역대 인증기준위원장 회고사

# 제1기 평가기준전문위원 시기 활동(2004.9-2007.8)

안덕선 (제1기 평가기준전문위원회 위원장)

## 1. 배경/근거

우리나라에서 의학교육의 질관리를 위한 평가인증제도가 도입된 지 어언 20년이 되고 있다. 도입 당시 정부의 무분별한 신설대학 인가로 계속적으로 출현하던 준비가 부족한 신설대학이 안고 있는 문제 해결과 의학교육의 질적 저하는 의료계의 심각하고 중대한 우려사안 이었다. 이런 의학계, 의료계 더나가 우리사회의 문제를 해결하고자 소수의 의학계 선각자들이 모여 우리나라 의학교육에 한 번도 시행되어 본적이 없는 의학교육 프로그램평가를 자체적으로 공부하고 연구하여 자력으로 평가인증에 관한 역량을 배양하여 제1주기 평가인증을 성공적으로 정착시키고 계속된 발전을 거듭하여 오늘날 한국의학교육평가원은 우리나라의 교육부와 세계의학교육연합회가 공식적으로 인정하는 평가인증기구가 되었다. 우선 이런 과정이 있기까지 많은 희생과 노력을 아끼지 않은 평가인증의 선구자 교수님들께 깊은 감사와 존경의 말씀을 올린다. 진정 어렵고 힘든 일은 전문직 사회에서 한 번도 해보지 않은 의과대학 전체의 질 평가와 관리 노력이었고 1주기 평가인증을 제도적으로 성공리에 정착시킨 1세대 평가인증 주체의 공로는 더 이상의 설명을 필요로 없게 한다. 2주기 평가인증은 이미 정착된 1주기 평가인증 체제에서 시작되었기에 이미 많은 난관이 극복되었고 의학교육의 수월성을 지향하기 위한 교두보적인 역할을 하기 위한 하부구조가 이미 완성된 이후에 진행되어 1주기 평가인증보다 유리한 위치에서 진행되었다고 하여도 과언은 아니다.

제2주기 의과대학 평가인증의 기준개발은 2004년 12월 31일 새로이 평가기준전문위원회가 구성되고 첫 모임이 시작됨으로 시작되었다. 제2주기를 맞이하여 당시 의과대학인정평가사업단 단장이었던 맹광호 교수님은 1주기 기준을 개발하기 위하여 우

선 1996년도에 출간된 대학교육협의회의 대학평가지침, 미국의 의과대학평가인증위원회[1] 호주 평가원[2])의 의과대학 평가인증 기준을 벤치마킹하여 우리나라의 실정에 부합되게 개발되었음을 밝혔다. 이어서 맹광호 교수님은 2주기 평가인증 기준개발은 1주기 평가인증 기준을 수정 보완하고 세계의학교육연합회[3]가 제시한 국제기준[4]에 맞추어 개발하여야 될 시점이라는 의견을 제시하였다.

제2주기 의과대학 평가인증의 시대적 배경은 2003년 세계의학교육연합회가 코펜하겐에서 세계의학교육연합회 학술대회를 개최하였고 당시 한국에서 많은 의과대학 교수들이 참가했었다. 세계의학교육연합회는 이 자리에서 기본의학교육[5], 졸업후교육[6], 평생전문직업성개발[7]의 의학교육의 전주기에 대한 3가지[8]의 국제기준을 제시하였다. 우리나라에서 많은 의과대학 교수들이 참가하게 된 계기는 정부의 강력한 의학교육 학제 개편정책으로 의학전문대학원 체제전환을 지원하기 위하여 많은 의학전문대학원에 교육과정개발을 위한 연구개발비가 지원되었기에 많은 의과대학 교수들이 새로운 의과대학의 기준에 관심을 가졌기 때문이다.

평가기준전문위원회의 제1차 위원회 회의에서는 제1주기 평가인증의 성공과 제1주기 평가인증에서 나타난 평가인증의 자체적인 개선점 등 평가인증의 전반적인 면에 열띤 논의가 이루어졌다. 주된 논의는 의학교육에 관한 프로그램의 평가는 일반 대학교육의 평가와 차이점이 존재한다는 것이 인정되어야 하고 일시적인 일회성 평가가 아닌 일정 주기를 갖고 주기적 평가체제를 확립이었다. 아울러 의학, 치의학, 한의학 분야에 대한 평가인증 기준의 차별성과 평가인증의 최종 판정결과에서 평가결과에 따른 교육기관별 등급이나 합격, 불합격의 개념이 적용될 수 있다는 우려가 제기되었

---

1) 미국의 의학교육평가인증위원회 LCME: Liason Committee on Medical Education
2) 호주 평가원 AMC: Australian Medical Council
3) 세계의학교육연합회 WFME: World Federation for Medical Education
4) Global Standards
5) Basic Medical Education
6) Post-Graduate Medical Education
7) Continuing Professional Development
8) Trilogy

다. 그리고 의과대학은 의학교육에 대한 프로그램평가 이외에 종합대학평가 같은 또 다른 평가로 이중적인 평가를 받는 일이 없어야 된다는 주장도 제기되었다.

한편, 프로그램평가와 대학전체를 평가하는 대교협의 대학교육평가와 차이점이 존재하고 이것은 곧 일반 학문분야의 교육평가와 의학교육과 같은 직업교육의 평가인증에 대한 명확한 구분이 필요하다는 주장도 제기되었다. 한편 전문직의 자율적인 그리고 독립적인 평가인증 제도를 구축하고 대학교육협의회의 대학평가와 중복적인 평가를 피하기 위하여 우선 2주기 의과대학 평가인증에서 대학교육협의회의 평가인증과 동등성을 확보하는 측면에서 대학원 교육이 포함되어야 한다는 의견과 절대평가와 상대평가 개념에 대한 문제도 논의되어야 할 것이라는 의견이 제시되었다.

그리고 제2주기 평가인증기준의 개발에는 종래의 기본기준과 우수기준 이외에 평가인증 결과판정과는 무관하나 우수기준을 상회 하는 최우수기준 설정하여 향후 다음 주기에서 최우수기준이 우수기준으로 채택될 수 있도록 하는 전략적인 방안도 제시되었다. 이런 경우 3단계의 성취수준 제도가 되어 차기 평가인증기준에 대한 충분한 사전 홍보와 공지가 되며 의과대학 교육발전의 방향을 제시할 수 있다는 장점이 있었다. 제1주기 평가인증은 신설의대의 수준 향상과 41개 모든 의과대학의 자발적인 참여를 유도하기 위하여 50개 평가인증 기준을 바탕으로 실시되었으나 주로 물리적 시설투자에 의한 정량적 평가기준이 중심이어서 제2주기는 의학교육의 내용과 과정을 살펴볼 수 있는 정성적인 혹은 질적인 평가로 전환되어야 한다는 공감대도 형성되었다. 평가인증의 기준개발과 아울러 41개 대학의 실제적 평가인증에서 평가자 간의 신뢰도 상승을 위한 평가 오차 범위 축소에 대한 연구주제의 제안도 있었고, 평가인증에 대한 평가자와 기관의 역량강화와 평가결과 신뢰도 확보를 위하여 평가인증에 참여하는 의과대학 교수를 대상으로 강력한 평가훈련과 교수개발이 필요하다는 의견들이 제시되었다.

## 2. 목적/원칙

2004년 12월에 시작된 제2주기 평가인증 기준개발을 위한 평가기준전문위원회의 첫 번째 활동으로 우선 평가인증의 목표를 설정하는 논리를 개발하고 이에 따른 평가인증 기준과 부차적인 계획을 수립하였다. 이것을 위하여 우선 제1주기 평가인증의 목표를 되새겨 보았다. 1주기 평가인증의 의미는 의학계의 자체적인 노력으로 최초로 의학교육에서 프로그램평가를 도입하였다는 사실과 평가인증제도의 성공적인 도입과 정착으로 당시 우리나라 41개 모든 의과대학이 자발적으로 참여하여 전문직의 자율규제 정신에 입각한 의학교육의 질 관리를 시도하여 의사집단의 단체적 전문직업성을 이룩하였다는 사실이다. 1주기 평가인증의 결과로 모든 의과대학이 의사양성을 위한 최소한의 기본적인 수준을 맞추기 시작하였다는 것도 의학교육의 역사상 매우 중요한 의미를 갖는다. 반면에 제2주기 평가인증의 목표 논리는 우리나라의 급속한 경제 사회적 발전으로 2004년 국민소득 2만 달러 도달이 예측되는 선진화 과정에 부합하는 의학교육의 질 향상을 위하여 본격적으로 선진국 진입을 위한 준비를 평가인증의 목표로 설정되었다. 이런 배경에서 잠정적으로 제2주기 평가인증에 한하여 통상적인 2단계 성취수준이 아닌 3단계 교육 성취수준을 제정한 것이다. 그러므로 제2주기 평가인증은 기본기준과 우수기준 그리고 영역별로 최우수기준을 개발하기로 결정하였고 최우수기준은 추후 제2주기가 끝난 이후 우수기준으로 전환하여 채택될 수 있는 전략을 수립하여 차기(포스트 2주기) 주기의 수준에 대한 예고를 하는 목적과 의학교육의 질적 향상의 방향을 제시하는 효과를 추구하였다. 달리 표현하자면 2주기 평가인증의 종료와 함께 차기 주기에서는 선진국과 동등한 의학교육의 수준에 도달한다는 것이 목표였다.

이와 같은 선진국 진입의 과도기적인 단계에서 2주기 평가인증도 1주기와 같이 의학교육의 프로그램 평가를 실시하는 선진국의 평가제도와 기준을 참조하여 평가인증 기준의 국제화도 달성하기로 결정하였다. 그러나 평가인증기준의 기본적 틀은 대학교육협회의 평가와 중복성을 피하고 의학계 자체로 실시하는 평가인증으로 대체할 수 있도록 하기 위하여 평가기준은 대학교육협회의 5가지 평가영역의 형식을 유지하기

로 결정하였다. 그리고 한 걸음 더 나아가 대학교육협의회의 대학원 평가기능과 동등성을 확보하고 대학과 연계된 졸업후교육에 관한 최소의 기준을 포함하기 위하여 6번째 평가영역으로 졸업후교육을 추가하기로 결정하였다.

## 3. 실행 과정/활동

제2주기 평가기준전문위원회는 평가인증 기준개발의 추진일정으로 2005년 5월에 개최되는 의학교육합동학술대회에서 제2주기 평가인증 기준에 대한 초안을 발표하기로 목표를 설정하는데 합의하였고 이를 위하여 부지런히 바쁘게 위원회를 가동하며 목표 일정을 달성하기 위한 노력을 하였다. 그리고 2005년 5월에 초안 완성의 목표를 달성하기 위하여 격주로 기준전문위원회를 개최하기로 결정하였다. 이러한 노력의 연장선에서 평가인증 기준개발의 추진일정에 맞추기 위하여 집중작업이 결정되어 2005년 4월 22-24일에는 부산 해운대 한화콘도에서 평가인증기준의 3단계별 기준인 필수, 권장, 최우수기준의 초안을 작성하였다. 이어서 2005년 5월 의학교육합동학술대회에 앞서 5월 25일 대구 인터불고호텔에서 당시 이종욱 의평원장의 인사말과 맹광호 인정평가사업단장의 인사말과 워크샵 일정소개로 “제2차 의과대학 인정평가 교수 워크샵”을 개최하였다. 의과대학 교수를 상대로 개발 중인 평가인증기준에 대한 설명과 평가영역별 분임토의를 개최하였다.

2주기 평가인증기준의 초안 발표 이후 새로운 기준에 따른 평가인증의 제도 개선을 위하여 의평원 산하 기획전문위원회와 기준전문위원회의 합동 연석위원회를 개최하였다. 평가인증에 결과에 따른 평가인증 부여 기간과 관련하여 인정유예로 1년의 기간을 그리고 개선사항의 정도에 따라 3년이나 5년의 인증기간을 부여하자는 의견을 제안하기로 결정하였고, 필수기준에서 한 항목 이상 충족하지 못할 경우 인증기준을 1년으로 권장기준에서 28개 이상을 충족하지 못할 경우 영역별로 15~20%, 인정기간을 3년으로 부여하고 재평가를 실시하는 안을 기획전문위원회에 건의하기로 하였다. 1주기에서 인증기간은 단일 4년으로 부여하던 것을 3년이나 5년으로 변경한 것은 평가인증에서 발견된 각 의과대학의 개선요구 조건에 따라 차등부여를 한 것이

고 대신에 1주기 용어와 제도였던 조건부인증이라는 용어는 더이상 사용하지 않기로 하였다. 우리나라에서 조건부인증이라는 용어는 매우 부정적인 의미와 의과대학에 대한 공식적인 등급을 부여하는 의미로 받아들여져 1주기에서 조건부인증을 받은 대학이 사회적으로 불명예로 간주하게 되었었다. 이 점을 개선하고자 인정기간의 차등부여를 통하여 조건부인증의 용어를 폐기하기고 결정하였다.

2주기 평가영역에서 신설된 제6영역인 대학원에 대한 평가기준은 추가로 개발하기로 하여 추후 검토하기로 하였다. 제6 졸업후교육 영역을 신설한 배경은 의과대학과 졸업후교육의 연계성을 강조하기 위한 의도도 포함되었었다. 그러나 졸업후교육에 대한 논란의 여지가 많아 대학원 교육으로 국한되어 기준을 개발하는 방향으로 추진되었다. 이후 각 대학과 공청회에서 올라온 여러 가지의 내용을 토대로 하여 평가인증기준에 대한 수정을 지속적으로 진행하였고, 2005년 11월 3일 “제2주기 인정평가를 위한 공청회”를 고려대학교 국제관에서 개최하였다.

2006년 1월 첫 회의에서 세계보건기구가 출간한 의과대학의 사회적 책무성에 관한 자료를 의평원에서 입수하여 각 대학에서 열람할 수 있도록 하자는 의견이 제시되었다. 1주기를 모두 경험한 우리나라의 모든 의과대학이 새로이 변경되고 선진국 진입을 위한 준비단계로 개발된 2주기 평가인증기준의 시행상 어려움을 호소할 것도 당연히 예상되고 각 의과대학에 새로운 평가인증 기준에 대한 설명과 설득하기 위하여 세계의학교육연합회의 국제기준과 비교 분석 작업을 하여 제2주기 평가인증기준에 대한 타당성을 확보할 필요성이 제기되었다. 그리고 2006년 5월 의학교육합동학술대회에서 제2주기 평가기준에 대한 최종안을 제시하고 설명회를 개최하기로 합의하였다. 세계의학교육연합회 기본의학교육 평가인증 기준과 2주기 평가인증 기준개발(안)과 비교하여 세계의학교육연합회의 평가인증 기준에서 현재 개발 중인 2주기 기준에서 누락된 항목을 일부 삽입하여 기술하기로 하여 2주기 평가인증 기준안을 재차 수정 보완하였다. 이후 지속적으로 기준에 대한 면밀한 검토와 수정 보강작업을 하였다. 당시 학장협의회와 2주기 평가인증 기준에 대한 의견 검토를 요청하여 검토의견을 취합하여 이를 반영하고자 다시 2주기 평가인증(안)에 대한 검토를 하였다.

26차 회의에서는 학장협의회에서 요청한 검토의견에 관한 검토와 결정사항을 도표로 만들어 정리하였고 아래와 같다.

| 학장협 검토의견 | 평가기준전문위원회 논의 및 결정사항 |
|---|---|
| • 1-1-2 [우수기준]은 현행 법 체계상에서는 어려우며 현실성이 없으므로 삭제해야 함. | • 1-1-2 설립주체와 대학의 분리에 대한 범위인 사권, 재정권 등 에 관한 설명을 추가. |
| • 1-1-3 사회적 책무성의 의미가 모호하므로 명확한 의미전달을 위하여 (주)를 추가해야 함. | • 1-1-3 설명이 충분하다고 보여짐. 의평원에 관련자료를 비치하고 있음. |
| • 1-2-1 [필수기준]의 "또한, 대학 운영을 위해 기본적으로 필요한 교육, 교수, 학생, 연구 담당 보직자(부학장급)가 임명되어 있어야 한다."는 대학의 규모나 특성에 따라 업무형태가 다른 점을 감안하여 "…등의 업무를 행정적 연관성과 관련하여 의과대학 보직자가 적절한 업무를 분담한다."로 변경해야 함. 또한 졸업 후 교육은 의과대학에서 시행하기 어려우며 대학원 교육으로 분류해야 할 것임. | • 1-2-1 최소기준으로 사료됨. 전문성이 있는 보직자(부학장급)가 필요함. 졸업후교육(대학원교육, 전공의교육, 평생교육)은 의과대학에서 반드시 필요함. 이와 관련하여 6-2-1에 우수기준으로 자세히 설명되어 있음. 또한 6-2-1에서 회의실적, 교육과정운영, 예산집행, 프로그램 등을 포함하여 내용강화하기로 함. |
| • 1-2-2 [우수기준]에서 "전결 가능 금액이 일회 1,000만원 이상이다"라는 문구 삭제 요청함. | • 1-2-2 삭제의 이유가 불충분함. |
| • 1-2-4 "대학 행정 구조"라는 용어 상에 문제가 있어 "대학 부속 병원이 지리적으로 떨어져 있는 경우 각 병원마다 교육과 연구를 담당하는 대학 행정 구조가 있는가?"를 "…를 지원하는 행정 구조가 있는가?"로 변경하며, 따라서 부록도 삭제해야 함. | • 1-2-4 지원의 경우 애매한 표현. '담당' 책임소재를 분명히 할 필요가 있음. |
| • 1-5-3 [권장기준]의 입학정원 기준 10% 이상은 현실성이 없으므로 5%로 조정해야 함. | • 1-5-3 4학년 전체에 대한 기준임을 밝힘. |
| • 2-2-1 [필수기준]에서 "기초의학 교육과 기생충학 관련 내용"에서 "기생충학"은 삭제해야 함. | • 2-2-1 기초내용이지 교실, 교과목이 아닌 의미가 아님. 우리나라를 비롯한 동아시아에서는 선택과목으로 채택되어 교육되고 있음. 삭제이유의 충분한 근거가 없음. |
| • 2-2-3 [필수기준]에서 "순수 기초의학 실험 실습비"에서 "순수"는 삭제해야 함. | • 2-2-3 순수는 삭제하고 [주]에서 기초의학 실험 실습비에 대한 구체적인 예를 기록하기로 함. |
| • 2-3-4 [필수기준]은 "최소 50주 이상"의 임상실습으로 이루어지며 임상실습 1주는 "최소 36시간"으로 조정해야 함. | • 2-3-4 55주로 깊은 토의로 통해 수정하기로 함. 1년 36주로 수용하기로 함.(5*8) |

| 학장협 검토의견 | 평가기준전문위원회 논의 및 결정사항 |
|---|---|
| • 2-3-5 [필수기준]에서 “핵심과의 외래 환자 중심 실습은 전체 실습 시간의 30% 이상 되어야 한다”는 삭제하되, [우수기준]을 “핵심과의 외래 환자 중심실습을 하는 학생들을 위한 별도의 Clinic이나 프로그램이 있다”로 설정하며, 부록으로 Clinic이나 프로그램의 운영실적으로 제시하게 함. | • 2-3-5 산출기준을 설명 8주중 2주는 외래환자 진료를 보는 것이 옳다고 생각됨. 최소 25% (예. 4주/1주)이상 으로 수정하기로 함. |
| • 2-3-6 [우수기준]의 “학생인턴제”에 대한 정확한 개념 설명이 필요하므로 (주)로 부연설명을 해야 함. | • 2-3-6 학생인턴제 [주]를 참고. 학생이 관람자. |
| • 2-5-2 [필수기준]은 [권장기준]으로 변경해야 함. | • 2-5-2 1주기에서 권장항목이었음. 기본 사항으로 간주됨. |
| • 3-1-2 [우수기준]에 전체 유급율을 3% 이하로 정한 근거가 의문임.적절한 근거가 있다면 (주)로 정리해주었으면 함. 또한 [우수기준]에 대학간 학점교류제도, 방학을 이용한 계절학기제도 등이 있는데, 현실적으로 의학과에서 이를 충족할 수 있는 대학은 없다고 생각되므로 이 항목은 삭제하는 것이 맞다고 생각됨. | • 3-1-2 현행 현황집 조사를 통해 결정한 사항임. [유급율 98년부터 02년간 5.85%~6.69%, 우수대학 중도탈락률 8.96~9.8%] 20%이상인 대학이 있었음(현황집 조사기준). 비율부분은 학교측 재량임. |
| • 3-2-1 [보고서 기술내용]의 (주)에서 “대여장학금” 제외라는 내용을 “동창회 기금을 이용한 대여 장학금 이외의 대여 장학금”으로 수정하는 것이 필요함. 또한 [우수기준]에서 장학금 지급비율을 20%로 정한 근거가 무엇인지 궁금함. 또한 장학금 지급 재원을 교내 재원보다는 교외재원을 활용하도록 독려하는 것이 바람직할 것이므로 단순한 20%보다는 외부 장학금이 차지하는 비율이 총장학금의 5% 이상이어야 할 것이라는 조항을 추가하는 것이 어떨까 함. | • 3-2-1 대여장학금 제외(단, 동창회 기금을 이용한 대여장학금은 포함)로 수정. |
| • 3-2-3 [우수기준]에서 “의대 전용 기숙사”라는 항목을 “재학생의 10% 혹은 희망 입사 학생의 50%를 수용하는 규모의 의대 전용 기숙사”로 변경하는 것이 좋겠음. | • 3-2-3 우수기준에서 '전부'를 삭제하고, "희망 입사학생 전원을 수용할 수 있는 규모의 의대전용기숙사"로 수정하기로 함. [주]를 통해 의대전용기숙사에 대한 의미(대학본부의 기숙사를 사용할 수도 있음)를 명확히 할 필요가 있음. |
| • 3-3-1 [우수기준]에서 최근 5년간 5% 항목은 2%로 조정하는 것이 바람직함. | • 3-3-1 우수기준이므로 그대로 수용하기로 함. |

| 학장협 검토의견 | 평가기준전문위원회 논의 및 결정사항 |
|---|---|
| • 4-1-1 [필수기준] “모든 의과대학은 세계의학교육협회가 권고하는 기초의학 교육을 위해 각 분야(13개 분야)에 최소 1명 이상, 총 30명의 기초의학 교원을 확보하고 있어야 한다.”를 “8개 기본분야(해부학, 생리학, 생화학, 병리학, 약리학, 미생물학, 예방의학, 면역학)에 최소 1명 및 기생충학, 유전학, 분자생물학, 생물물리학, 세포생물학분야 포함 총 25명”으로 변경해야 함. | • 4-1-1 WFME 기준을 참조 |
| • 4-1-2 [필수기준] (주)의 “단 겸무교수는 해당되지 않는다”는 타과의 관련 교수를 동원하여 내실 있는 교육을 하는 것은 가능한 일이므로 삭제함. (주) “예방의학(의료관리, 의료정책, 역학 등)과 법의학은 전공에 따라 인문사회의학으로 분류한다.”에서 이 분야는 기초의학 인력분야와 동시에 가능하다는 것을 명기해야 함. | • 4-1-2 [권장]으로 수정되었으며, 그 중 1인은 [주1] 중 한 전공의 전임교수를 확보하고 있다. |
| • 4-1-3 [권장기준]에서 (주)는 삭제해야 함. | • 그대로 유지하기로 함. |
| • 4-2-1 [필수기준]을 [권장기준]으로 변경해야 함. 또한 질적 평가가 함께 이루어질 수 있도록 제1저자, 교신저자는 1편으로 인정해준다는 (주)를 추가해야 함. | • 4-2-1 이전 기준과 비교할 때 타당하다고 생각되며, 개인평가와는 차이가 있음을 분명히 함. |
| • 4-3-1의 기준은 과다하며 현실성이 없으므로 [필수기준]의 “연간 교수 1인당 8시간 이상”을 4시간 이상으로, [우수기준]의 “최소 30시간 이상 실시”, “년 4회 이상”을 “최소 15시간 이상”, “년 2회 이상”으로 변경해야 함. | • 그대로 유지하기로 함. |
| • 4-3-2 [필수기준]에서 “연간 교수 1인당 200만원 이상”은 “100만원 이상”으로, [우수기준]의 ‘교수 1인당 500만원 이상“을 ”300만원 이상“으로 조정하며, 병원에서 재정적으로 지원한 경우도 인정한다는 내용을 (주)로 정리해야 함. | • 그대로 유지하기로 함. |
| • 4-3-3 [우수기준]에서 “계열별 전문교수의 경우 한 영역의 업적만으로 직위, 호봉승진이 가능하다(예: 연구, 진료, 교육의 단일 업적)”은 삭제해야 함. | • 그대로 유지하기로 함. |

| 학장협 검토의견 | 평가기준전문위원회 논의 및 결정사항 |
|---|---|
| • 5-1-1 [우수기준]을 [필수기준]에 포함시켜야 하며, “교육을 전담하는 인력”을 구체적으로 명시할 필요가 있음. | • 5-1-1 '교육 및 관리' 로 수정하기로 함. |
| • 5-1-2 [필수기준]에서 “실습학생 20명당 1개씩의 학생 전용 공간을 확보하고 있어야 한다.”를 [우수기준]에 포함시키되 “20명당 1개씩”은 삭제하고 (주)를 수정하여 병원내 실습학생 전용공간 1실을 확보하고 전공의, 간호사 등 타인력과 공동으로 사용하는 공간도 가능하게 해야 함. | • 5-1-2 '우수기준에서 필수기준을 충족하고' 를 첨부 |
| • 6-2 전공의 교육 및 평생교육은 병원에서 이미 시행하고 있는 내용으로 의과대학의 인정평가기준으로 정하기에는 현실성이 결여되어 인정평가 항목에서 삭제할 것을 요구함. | • 그대로 유지하기로 함. |
| • (기타 의견) 현행 계획서에 의하면 현지 방문조사에 소요되는 비용을 평가받는 대학에서 부담하는 것을 원칙으로 한다고 되어 있음. 평가원이 법인체로 출범한 이상 자체적으로 소요 예산을 확보하여 사용하여야 함. 전국의 의과대학(원)이 회원으로 회비를 정기적으로 내는 방법으로 소요 재원을 확보하더라도 이 평가사업을 위한 재원을 마련하고 이를 이용하여 평가를 하는 것이 바람직함. 대학별로 평가를 받을 때에 소요 경비를 부담시키는 것은 여러 가지로 바람직하지 않은 방법임. | • 기획전문위원회에서 논의할 사항임. |

2006년 4월에는 이미 2004년 12월에 개최된 2주기 평가기준전문위원회에서 제시된 의견에 따라 의평원의 2006년 사업으로 “평가자 오차 최소화 방안연구”를 양은배 교수에게 의뢰하였다. 그리고 학장협의회에서 평가기준에 관한 연속적인 수정요구사항에 대한 의견 검토를 지속적으로 논의하고 확인하였다. 이러한 지속적인 요구와 검토의 과정으로 2주기 평가인증기준의 최종(안)이 점차 윤곽을 잡아가고 있었다. 2006년 11월에는 의평원이 제시한 최종안에 대한 의과대학장협회의 2주기 평가인증기준에 대한 최종수정사항요청에 관한 9가지 내용을 검토하고 이에 대한 결정을 하

였다. 검토사항은 제31차 회의록 내 도표로 되어있다.

| | 수정 요구 사항에 따른 내용 |
|---|---|
| 수정요구 1 | 1) 제목의 '작 병원마다 교육과 연구를 담당하는 대학 행정 구조가 있는가?'를 각 병원마다 교육과 연구를 지원하는 행정구조가 있는가'로 수정하기로 함.<br>2) 부록의 '부속병원별 대학행정구조'를 부속병원별 교육연구지원 행정구조'로 수정하기로 함. |
| 수정요구 2 | 1) 필수기준에서 '기초의학 교육과 기생충학 관련 내용이 교육되어야 한다'를 '기초의학 교육내용이 교육되어야 한다'로 수정하기로 함. |
| 수정요구 3 | 1) 학장협의회에서 보고서기술내용, 권장기준, 우수기준 모두에서 "최근 5년간"을 "졸업후 10-20년간"으로 수정을 요구하였으나, 위원회 논의결과 수정을 보류하기로 함. |
| 수정요구 4. | 1) [주] "재정지원이라함은 연구비, 장학금 등을 포함한다"를 추가하기로 함.<br>2) 비치 : 1. '인사기록 및 급여대장'을 '인사기록 및 급여대장 또는 연구비 및 장학금 지급대장'으로 수정하기로 함. |
| 수정요구 5 | 1) 학장협의회에서 "필수기준 : 전임교수 1인당 국내 연구실적 연평균이 1.0이상이어야 한다"에서 연구실적 연평균 1.0을 0.5 또는 0편을 요구하였으나, 위원회 논의결과 1.0을 원안대로 유지하기로 결정함. |
| 수정요구 6 | 1) [보고서 기술내용]에 '단, 공동연구의 경우 1인당 평균 건수는 매 연구실적 1편을 저자수(N)으로 나누어 계산하되, 제1저자 또는 교신저자의 소속 대학이 다를 경우 연구 실적 1편의 70%를 제1저자 또는 교신 저자 1인에게 부여할 수 있고, 나머지 30%는 다른 공동연구자에게 균등하게 부여할 수 있다"를 적용하기로 함. |
| 수정요구 7 | 1) 필수기준의 '연간 교수 1인당 8시간'을 연간 교수1인당 4시간'으로 수정을 요구하였으나, 위원회에서 논의결과 '8시간'을 원안대로 유지하기로 함 |
| 수정요구 8 | 1) 학장협의회에서 필수기준 의 '연간 교수1인당 200만원'을 '연간 교수 1인당 100만원'으로 수정을 요구하였으나, 위원회 논의결과, '연간 교수 1인당 200만원'을 원안대로 유지하기로 함. |
| 수정요구 9 | 1) 필수기준과 우수기준에서 "학생전용공간"을 "학생 학습 공간"으로 수정하기로 함.<br>2) 참고자료2와 3에서 "학생전용공간"을 "학생 학습 공간"으로 수정하기로 함. |

## 4. 결과/성찰

2006년 2주기 평가인증기준전문위원회가 준비한 2주기 평가인증기준 최종본을 완성하고 2007년에는 최종본에 대한 설명과 홍보를 위한 작업과 각 의과대학이 2주기 평가인증 준비의 편의를 위한 예상질의응답과 평가지침서를 제작하였다. 2007년 3월에는 전라남도 진도에서 의과대학으로부터 들어온 평가인증 관련 질문에 대한 답변과 각 대학에 배포된 평가지침의 최종본을 확인하고 수정사항을 결정하였다. 35차 평가기준전문위원회는 3월 15~17일 전남 신안군 증도면 엘도라도 콘도에서 안덕선, 김선, 김명곤, 양은배. 이정애, 최금자 위원의 집중작업으로 양은배 교수가 수행한 평가인증의 오차를 최소화하기 위한 연구에서 제시한 실제 분석보고에 대한 토의와 결정이 있었고 무엇보다도 2주기 인정평가 평가자 지침 관련 분임토론 작업을 수행하였다. 이후에도 전문위원회는 의과대학으로부터 지속적으로 제기되고 있는 여러 가지 질문사항에 대한 답변을 제공하고 국문사용의 오류나 오타가 발견된 기준에 관해서 수정을 하였다. 이후 제38차 위원회가 2007년 8월 개최되어 의과대학 평가인정 평가기준안과 "평가인증오차축소방안"에 관한 보고서를 검토하였고 공식적으로 채택하여 의평원에 제출되었다. 2주기 평가인증 기준개발을 위해 기준전문위원회는 임기 내에 38회의 회의를 개최하였고 해운대와 수안보 그리고 증도에서 워크숍과 집중작업을 진행했다. 아울러 2005, 2006, 2007년 의학교육합동학술대회에서 평가인증에 관한 발표와 지속적인 논의를 하였다. 이외에도 세부사항의 논의를 위하여 의과대학장협회와 지속적인 논의를 통하여 상호 협력하고 노력하여 2주기 평가인증기준 개발을 완성하게 되었다. 2주기 평가인증 개발은 앞에서 열거한 바와 같이 많은 교수님들의 열정과 헌신을 바탕으로 개발되었고 평가대상 대학의 연합체와 같은 활동을 한 의과대학장협회의 동반된 노력에 완성이 가능하였다. 2주기 평가인증기준 개발을 위하여 애써주신 교수, 직원 그리고 의평원, 의과대학장협회 모두에게 심심한 감사의 말씀을 드린다.

## 5. 발전방향

세계의학교육연합회와 미국의 ECFMG의 공조 그리고 세계보건기구의 정책방향은 앞으로 기본의학교육에 대한 평가인증제도의 정착은 물론 보건의료인력 개개인에 대한 질관리 체제까지 확산할 것을 요구하고 있다. 이미 성과바탕의 개념을 중심으로 기본의학교육과 졸업후교육의 변화가 시작되었고 평생 전문직업성 개발도 궤를 같이하는 추세로 전환되고 있다. 우리나라의 기본의학교육에 대한 평가인증은 아직 성과바탕을 위한 질적인 평가로 전환되기에는 여러 가지 장애물이 존재한다. 75% 이상이 민간 사립의과대학으로 많은 대학의 재원은 등록금과 병원 수익에 의존하는데 병원경영의 성패에 따라 의학교육이 심각한 영향을 받을 수 있는 구조이고 여전히 재정적 투자의 순위를 점하고 있는 것은 의학교육보다는 병원경영이 우선이다. 비록 기본의학교육에서 평가인증으로 질 관리에 도달하였다고 하나 인턴과정과 전공의과정의 평가인증의 일관성의 결여로 평가인증으로 인한 선순환 구조를 구축하기 힘든 상태에 있다. 이제 성과바탕교육에서 제시하는 새로운 개념인 역량의 발달단계[9]와 위임가능 직무[10]를 이용하면 실제 의료현장에서 나타나는 역량의 간극이나 부족분에 대한 요구가 기본의학교육과 졸업후교육에 반영될 수 있을 것이라는 예측도 가능하다. 결국 현재의 평가인증의 방향은 전주기 의학교육이 연계될 수 있도록 성과지향평가인증제도의 구축이다. 이런 경우 의료현장에서 진료성과[11] 분석에 근거한 환류자료가 의과대학과 전공의 교육에 반영되어 의학교육의 평가인증이 분절된 질 관리가 아닌 의사의 역량과 직접적으로 연결될 수 있을 것이다.

9) Milestones
10) Entrusted professional activities
11) Clinical outcome

# 제2기 평가기준전문위원회 시기활동
# (2007.9.1. - 2010.2.28.)

김영창 (제2기 평가기준전문위원회 위원장)

제1기 위원회의 주요 사업은 새 평가기준 개발과 서면·현지 방문평가자 교육훈련 프로그램 운영과 대학자체평가 위원 능력 함양을 위한 워크숍 개최이었다면, 2기 위원회는 새 기준에 의한 2007년부터 시작된 2주기 평가인증 과정에서 나타난 문제점을 검토하여 지속적으로 개선하는 노력을 하였다. 또한 객관적인 평가를 위해서는 평가자들이 기준에 대한 명확한 이해가 필요하여 평가오차최소화 방안(안)에 따른 후속조치로 평가능력 제고를 위한 평가전문인력 양성교육 워크숍을 개최하였다. 또한 향후 의학교육의 변화를 수용하고 촉진하기 위해 Post 2주기 평가기준의 개선 필요성에 따라서 미국, 호주 및 유럽의 국제동향을 파악하였다.

2009년부터는 평가인증 시행을 주기개념이 아니고, 3년, 또는 5년의 인정기간에 따라서 시행하기로 변경하였다. 이에 따라서 Post-2주기 평가인증 또는 평가기준이란 명칭을 사용하기 시작하였다.

2기 위원회 위원 명단과 주요 활동은 다음과 같다

| 위원 명단 | 주요 활동 |
|---|---|
| 위원장 김영창 (순천향의대)<br>위 원 김 선 (가톨릭의대)<br>양은배 (연세의대)<br>김정수 (신, 전북의대)<br>서순팔 (신, 전남의대)<br>윤희상 (신, 경상의대)<br>간 사 김명곤 (고려의대) | - 제2주기 평가기준에 대한 질의 응답<br>- 제2주기 1차 년도의 평가인증결과를 통한 평가기준 적용의 문제점 파악과 Post 2주기 기준을 위한 자료 수집<br>- 평가 전문 인력 양성<br>- 신설의과대학 가인증 평가기준 개발 |

## 1. 2주기 평가기준에 대한 질의 응답

새로운 평가기준이 새롭게 개발된 관계로 대학이 질문이 많았다. 매번 위원회 회의마다 논의가 있었다. 논의 결과를 대학에 통보하고 전체적으로 평가기준 관련 질의사항에 대한 답변을 홈페이지에 올려 평가대상 대학들이 참고할 수 있도록 하였다(표 1).

〈표 1〉 질의응답 예시

| |
|---|
| [1-2-1] 대학 행정은 업무별로 구분되어 있고 인력 확보는 적절한가?<br>[우수기준] 교육, 학생, 교수, 연구 분야를 포함한 최소 7개 분야의 보직자가 임명되어 있으며, 그 활동실적이 있다.<br>※ 증명자료 : 업무회의록 및 활동실적, 보직수당 지급내역에 대한 질의입니다.<br><br>Q1. 의료원 조직(대학, 병원 동시 지원)에서 의과대학의 재정, 인사, 정보 등 모든 행정업무를 지원하는 경우, 이에 대한 보직도 7개 분야에 해당하는 다른 분야로 볼 수 있는지요?<br>ex) 경리팀장, 인사팀장, 의료정보팀장 등<br>A. 의료원 조직은 다른 단과대학 및 병원 업무를 관장하므로 원칙적으로 인정할 수 없습니다.<br><br>Q2. 다음 보직자도 교육, 학생, 교수, 연구 분야와 별개로 다른 분야 보직자로  보아 우수기준의 7개 분야에 포함시킬 수 있는지요?<br>- 연구부학장과는 별도로 의과대학 소속 임상의학연구센터소장 및 연구부장, 실험동물부장<br>- 의과대학 소속 의학박물관장 보직<br>- 의과대학 소속 교수가 본교 의예과 책임담당교수를 맡고 있는 경우<br>※ 발령은 본교에서 내고 보직수당은 의과대학에서 지급함.<br>- 지역적으로 떨어져 있는 산하병원의 교수지원을 위한 의과대학 부학장 보직<br>- 의과대학 소속 부설연구소장 보직<br>- 의과대학 소속 교실주임교수 보직(교수연구 및 행정지원)<br>- 의과대학 소속 대학원 행정파트장(의대 대학원 학사업무)<br>- 의과대학 학장 및 의과대학 사무팀장<br>A. 제시한 예 중 '지역적으로 떨어져 있는 산하병원의 교수지원을 위한 의과대학 부학장 보직'을 제외한 다른 것은 포함할 수 없습니다.<br><br>Q3. 보직자로 임명하여 발령을 받고(발령장수여) 활동하고 있으나, 내부사정으로 보직수당은 지급하지 않을 경우 평가기준에 해당하는 보직자로 볼 수 있는지요?<br>A. 보직자로 임명을 받고 보직수당을 받는 보직자만 보직자로 인정할 수 있습니다. |

## 2. 제2주기 1차 년도의 평가인정 결과를 통한 평가기준 적용의 문제점 파악과 Post-2주기 평가기준 개발을 위한 자료 수집

### 1) 위원회 집중 작업

기준위원들의 위원회 전반적 업무에 대한 이해를 증진시키기 위한 retreat 트레이닝과 2주기 1차 년도 평가 후 평가기준의 해석과 오차 최소화 방안 수립과 관련된 그동안의 연구, 개발 결과를 종합적으로 검토할 목적으로 기준위원회의 워크숍이 2008년 7월 2일부터 3일까지 진주 아시아레이크사이드호텔에서 개최되었다. 워크숍에서 2007년 대학별 평가기준 충족 결과와 평가기준 충족 결과에 따른 기준을 검토를 하였다.

평가기준과 관련하여 평가대학 관계자와 평가위원에 대한 보다 심도 있는 교육의 필요성이 있어 2주기 평가인증 1차년도 판정위원회 자료집 중 3개 대학이 미충족된 필수와 권장기준을 대상으로 충족여부 판정에 영향을 미치는 요인으로 1) 평가기준, 2) 평가자, 3) 피평가 대학 측면에서 분석하였다. 집중작업 결과 문제가 평가기준에 있을 경우 수정이나 보완을 하고, 평가자인 경우는 평가전문 인력 양성 워크숍을 통한 교육을 통해서, 피평가 대학에 있다면 자체평가단 워크숍을 통한 안내와 정보를 제공해서 평가의 객관성을 유지하는 방안을 제시하였다.

### 2) 인증평가단장과 기획전문위원회의 요청 사항을 검토하였다(표2).

〈표 2〉 검토요청 사항 (일부 예)

| 검토요청 사항 | 검토 내용 |
|---|---|
| 4-2-1과 4-1-2 항목<br>연구실적을 국내외로 구분한 근거가 여러 이유가 있으나 최근에 논문을 SCI 급에 발표하는 추세이므로 교수 및 학교입장에서 현실적으로 어려움이 많다 | 어려움을 인식하고 현실적 측면을 고려하여 몇 가지 해결 방안을 고려했으나, 2007년 평가에서 4-2-1은 두 대학만이 미충족되었고, 4-2-2 기준은 모든 대학이 충족되어 기준자체의 문제라기보다는 대학 자체의 문제도 간과할 수 없어 2008년 평가결과를 지켜본 후 수정 여부를 논의하기로 함 |

3) 2007년도 방문평가단 건의사항을 검토하였다(표 3).

〈표 3〉 방문평가단 건의사항

| 검토요청 사항 | 검토 내용 |
|---|---|
| 1-1-3 사회적 책무성 기준을 필수에서 권장으로 변경 | 긍정적 검토 |
| 1-5-2 제1주기 평가결과보고서 회람 및 공개 추가요청 | 수락 |
| 1-5-3 외국 교류 건에서 학생 수가 적은 대학의 기준완화 요청 | 수락하지 않음 |
| 4-1-3 '대학에서 직접적으로 재정적 지원' | '직접적' 문구 삭제 |

## 3. 평가 전문 인력 양성

제2주기 1차년도 평가인정 결과를 바탕으로 평가 인력의 전문성 제고를 위해서 평가전문 인력양성 워크숍을 매년 1회 개최하였다 (표4). 41개 대학으로부터 2-3명의 평가위원을 추천받아 평가전문 인력풀을 구성하였다.

〈표 4〉 평가전문 인력양성 워크샵

**인정평가 전문인력 양성을 위한 워크샵**

□ 일 시 : 2009. 1. 16(금)

□ 장 소 : 서울대학교 암연구소

□ 일 정

1차- 오전

| | | |
|---|---|---|
| 08:30 ~ 09:00 | 등 록 | |
| 09:00 ~ 09:15 | 인사말 | |
| | 이무상 한국의학교육평가원장 | |
| | 이윤성 인정평가단장 | |
| 09:15 ~ 09:35 | 국내 의과대학인정평가제도 | 김영창 교수 |
| 09:35 ~ 10:00 | 의과대학 인정제도의 세계적 동향 | 김 선 교수 |
| 10:00 ~ 10:10 | 휴 식 | |
| 10:10 ~ 12:00 | 영역별 분임토의 | 평가기준전문위원 |
| | 1. 대학운영체계 | 김영창 교수 |
| | 2. 교육목표 및 교육과정1 | 김선 교수 |

| | | |
|---|---|---|
| | 3. 교육목표 및 교육과정2 | 서순팔, 김명곤 교수 |
| | 4. 학생 | 윤희상 교수 |
| | 5. 교수 | 양은배 교수 |
| | 6. 시설설비 | 김정수 교수 |
| 12:00 ~ 12:30 | 종합토의 | |
| 12:30 | 폐회 | |
| | | |
| 2차- 오후 | | |
| 13:30 ~ 14:00 | 등 록 | |
| 14:00 ~ 14:15 | 인사말 | |
| | 이무상 한국의학교육평가원장 | |
| | 이윤성 인정평가단장 | |
| 14:15 ~ 14:35 | 국내 의과대학인정평가제도 | 김영창 교수 |
| 14:35 ~ 15:00 | 의과대학 인정제도의 세계적 동향 | 김 선 교수 |
| 15:00 ~ 15:10 | 휴 식 | |
| 15:10 ~ 17:00 | 영역별 분임토의 | 평가기준전문위원 |
| | 1. 대학운영체계 | 김영창 교수 |
| | 2. 교육목표 및 교육과정1 | 김선 교수 |
| | 3. 교육목표 및 교육과정2 | 서순팔, 김명곤 교수 |
| | 4. 학생 | 윤희상 교수 |
| | 5. 교수 | 양은배 교수 |
| | 6. 시설설비 | 김정수 교수 |
| 17:00 ~ 17:30 | 종합토의 | |
| 17:30 | 폐회 | |

## 4. 신설 의과대학 가인증 평가기준(안) 개발

2009년 초 이무상 원장이 교육부가 '의대신설 설립인가 준칙'을 새로 만들려는 움직임이 있어 미리 인증단에서 갖고 있는 기준과 규정을 기반으로 미국 LCME 의 신설대학 평가기준과 같은 가인증 평가기준 개발 연구를 제안하였다. 의과대학, 의학전문대학원 신설을 희망하는 대학이 의학교육의 현실을 정확히 인식할 수 있는 계기를 마련하고, 의학교육의 발전을 위한 정부와 대학에 대한 자문을 제공할 목적으로 가인증 평가기준(안)을 개발하였다.

LCME 기준과 같이 의대 설립 시 학생 선발부터 졸업까지 4년의 과정을 평가하도

록 하며, 현재 인증평가의 필수·권장기준만을 가인증 평가기준에 활용하기로 결정하였고, 2009년 8월경 가인증 평가기준(안) 초안이 마련되었다.

LCME 자료를 참고하기 위해서 2009년 11월 6일부터 12일까지 개최되는 미국 AAMC 학회에 박주현, 채수진, 김영창, 배정윤 등이 참석하여 LCME 담당자를 만나서 자료를 요청하였다. 김영창 위원장은 LCME가 보내 온 가인증 평가기준 자료를 규정(김영창), 대학운영체제(김명곤), 교육목표 및 교육과정(김 선), 학생, 교수(윤희상), 시설/설비(김정수) 영역별로 나누어 검토하였다.

가인증 규정(표 6)은 미국, 호주의 신설대학 평가인증의 규정을 벤치마킹하여 개발하여 의평원 규정에 삽입하였고, 의과대학 가인증 평가기준(안)을 2010년 1월에 완료하였다.

〈표 6〉 가인증 규정

**신설 의과대학/의학전문대학원 인증절차 규정**
**(The Rules of Accreditation Procedure for the Establishment of New Medical School)**

1. 대상
   신설 의과대학 또는 의학전문대학원의 학생 교육에 필요한 모든 교육과정 및 시설설비를 포함한 교육 환경은 가인증평가 대상이며, 첫 졸업생이 배출될 때까지 매년 인증평가를 받아야 한다.

2. 인증 종류
 (1). 가인증 (Preliminary Accreditation)
 (2). 임시인증 (Provisional Accreditation)

3. 평가 절차
 (1) 가인증 (Preliminary Accreditation)
  - 목적 ; 신설 의과대학 또는 의학전문대학원이 사회에서 원하는 능력있는 의사를 양성하기에 적절한 교육과정 및 교육환경을 갖추었는지를 평가한다.
  - 가인증 평가를 받고자하는 대학은 한국의학교육평가원(이하, 의평원)에 평가받기를 원하는 시점으로부터 18개월 전에 소정의 평가 소용비용과 더불어 가인증평가 신청서을 제출해야 한다.
  - 해당 대학은 가인증 평가기준에 따라서 자체평가 보고서를 작성한다. 언제 어떻게 교육과정이 완전인증(full accreditation) 을 위한 평가를 받을 것이라는 내용을 포함한 학교의 교육목적과 목표를 기술해야 하며, 인증평가사업단에서 제공하는 의학교육데이타베이스(KOMSIS: Korean Medical School Information System)에 자료를 입력해야 한다.
  - 자체평가보고서는 평가를 받고자하는 시점으로부터 6개월 전까지 의평원에 제출해야하고, 필

요시 의평원은 미비서류가 있으면 추가 자료를 요청할 수 있다.

- 의평원은 해당 대학과 상의 한 후 방문평가(limited survey) 일정을 결정하여 통고한다. 단, 가인증을 받기 전에는 학생모집광고 및 신입생모집은 할 수 없다. 그러하지 못하면 가인증 평가를 받을 수 없다.
- 평가방법은 완전인증 방법과 동일하다.
- 현지방문 평가 후에 모든 기준이 충족되었다고 판단되면 가인증(preliminary accreditation)이 되며 신입생을 모집할 수 있다. 만약 인증 후 2년 내에 신입생을 선발하지 못한다면 다시 가인증 평가를 신청해야 한다.
- 가인증평가를 통과하지 못한 경우 이의신청(appeal) 을 할 수 있다. 이의신청 절차는 완전인증 방법에 준한다. 가인증 결과에 대한 재평가는 인증평가 결과를 문서로 통보한 시점으로부터 1년 내에는 신청할 수 없다. 만약 인증을 받지 못한 상태에서 신입생이 들어오면 향후 평가받을 자격이 취소되고 그 학년이 졸업한 후에는 다시 신청할 수 있다.

(2) 임시인증 (Provisonal Accreditation)

- 목적 ; 임시인증이란 가인증을 받고 신입생을 모집한 후 처음에 계획되었던 교육과정 및 교육관련 시설설비가 재대로 이행되고 있는지 여부와 이에 따른 학생들의 만족도를 평가하여 의학교육의 질적 수준을 끌어올리고 유지하는데 있다.
- 대학은 가인증을 받고 신입생 모집한 후에도 자체평가 및 자료를 update 해야 한다.
- 대학은 가인증 획득 후 1년 후 (신입생이 2학년이 될 때) 적절한 시점에 처음 계획이 제대로 시행되고 있는지 여부에 대한 서류평가 및 현지방문 평가를 받아야 한다.
- 모든 교육과정 및 계획이 잘 이행되고 있으면 임시인증(provisional accreditation)을 받는다. 만약에 미비점이 사소한(minor)하다고 판단되면 임시인증을 받지만 개선 보고서를 제출해야 한다. 그러나, 미비점이 상당히 크다고 판단되면 다음과 같은 조치를 취할 수 있다. 첫째, 미비점이 비교적 일정기간(limited period) 내에 개선될 수 있다고 판단되면, 가인증 상태를 1년간 더 연장한다. 그런 경우 새로운 신입생을 더 모집 못하며 기존 학생들에게 대한 교육을 강화해야 한다. 의평원은 개선서류 또는 현지방문평가를 1년 내에 요구할 수 있다. 이때 다시 기준을 충족한다면 그때부터 신입생 선발이 가능하다.
  둘째, 미비점이 일정기간 내에 개선될 수 없고, 처음 평가 시 지적되었던 문제가 시정되지 않을 것으로 판단되면, 가인증을 취소할 수 있다. 해당 대학은 이에 대한 이의(appeal) 신청할 수 있다. 인증을 취소당한 경우 재학생은 가능한 다른 대학으로 전학조치를 취한다. 서면으로 해당대학에 취소결정을 통보 한 날로부터 1년 내에는 재평가를 요청할 수 없다.
- 임시인증을 받은 후에도 지속적으로 자체평가를 통해서 완전인증(full accreditation)을 받기 위해서 미비점 개선 및 보충을 지속해야 한다. 임시인증 상태를 유지하기 위해서는 가인증 후 신입생이 졸업하는 때까지 매년 받아야 된다.
- 3학년 말 또는 4학년 초에 완전인증을 위한 서면 및 현지 방문 평가를 받아야 한다. 이때 평가 시 나타난 미비점이 사소하다고 판단되면 개선보고서를 제출할 것을 전제로 완전인증을 받는다.

4. 그 외 평가단 구성, 현지방문 평가일정, 보고서 작성 및 인증 결과 판단은 완전 인증 규정을 따른다.

# 제3기 평가기준전문위원회(2010.3-2013.2)

이정애 (제3기 평가기준전문위원회 위원장)

## 1. 배경

한국의학교육평가원은 의과대학의 의학교육 기본과정부터 졸업 후 교육까지 공정하게 평가하여 인증함으로써 대학의 의학교육 수월성(excellence)과 사회적 책무성(social accountability) 확보를 목표로 노력해 왔다. 2019년이면 우리나라가 의과대학 평가인증제도를 도입한지 20년이 된다 하니 참으로 세월이 빠르게 지나가는 느낌이다. 한국의학교육평가원은 의학교육프로그램 평가인증 제도를 자체적으로 개발하여 41개 의과대학을 대상으로 제1주기(2000-2004) 평가인정을 성공적으로 실시하였다. 또한 제2주기(2007-2010) 평가인증은 제1주기 평가인정 제도를 더욱 발전시킴으로써 의학교육의 수월성과 각 대학의 특수성을 지향하기 위해 노력하였다.

2010년 제2주기(2007-2010) 평가인증이 모두 완료됨에 따라 새로운 Post-2주기 평가항목과 기준 개발이 필요하게 되었다. 2010년 3월 23일 이정애 교수를 평가기준 위원장으로 임명함으로써 Post-2주기 평가기준 개발 위원회가 활동을 시작하였다. 특히 Post-2주기에는 세계화와 국제화 시대에 걸맞은 국제기준 수준의 평가기준 개발이 필요하다는 의견이 대두되었다. 따라서 위원회는 2주기 평가기준 및 국제기준 등을 검토하여, 타당하고 신뢰 가능한 Post-2주기 평가기준을 가능한 2011년 상반기 내에 완료하기로 하였다.

## 2. 목적 및 원칙

2010년 3월에 시작된 평가기준개발 위원회는 새롭게 시작한 Post-2주기 위원회로, 우선 위원회의 목적과 역할을 분명히 설정하는 것이 중요하다는 중지를 모았으

며, 제1주기와 제2주기의 평가인증 목표와 결과를 분석해 보았다. 제1주기 평가인정은 최초의 자발적인 의학교육 프로그램의 평가체계로 의과대학의 기본적이고 필수적인 교육의 질을 평가한 반면에, 제2주기는 선진국 진입을 위한 더 높은 의학교육의 질 향상에 목표를 두었다. 이에 따라 Post-2주기는 세계화와 국제화에 걸맞은 의과대학 의학교육평가에 대한 객관성을 확보하기 위한 평가기준을 개발하는 것과 그 평가결과에 따라 평가항목과 기준을 보완하고 관리함으로써 선진국 수준의 의학교육 질 향상에 있다고 결정하였다.

일단 평가기준개발 위원회의 목적을 설정한 후에 위원회 전문위원들의 진지한 토론 끝에 세부적인 활동 목표를 다음과 같이 결정하였다.

(1) 새로운 평가항목과 기준 개발 및 가이드라인 개발
(2) 평가 전문인력 양성을 위한 교육
(3) 서면·현지 평가자를 위한 교육(워크숍)
(4) 각 대학의 자체평가 위원의 능력 개발(워크숍)
(5) 국제 평가기준에 대한 연구
(6) 새로운 평가기준 개정안에 대한 공청회 개최
(7) 교육부 고등교육 평가인증 인정기관 신청에 관련한 사항 등

그리고 Post-2주기 평가기준개발 위원회의 목적과 세부적 활동 목표를 결정한 다음에, 가장 중요한 Post-2주기 평가기준개발을 위해서 제1주기, 제2주기 및 국제기준 등을 면밀히 검토하여 Post-2주기 평가기준 개발의 기본 원칙(방향)을 다음과 같이 확정하였다.

(1) 의과대학 및 의학전문대학원의 사회적 책무성 수행 강화를 위한 기준
(2) 의학교육 질 향상을 위하여 국제적 수준에 도달하는 기준 개발
(3) 대학별 특성화 노력과 교육과정운영 등을 고려한 정성적 평가기준 개발
(4) 각 대학의 특수성과 수월성을 위한 우수기준의 개발
(5) 기초의학, 임상의학, 인문사회의학의 적절한 통합 또는 연계 교육 강화
(6) 성과중심(outcome-based) 교육 프로그램 및 성과중심 평가체계 실시

## 3. 실행과정 및 활동

### (1) 평가기준개발 위원회의 임원 및 담당 영역

전남대학교 의과대학 이정애 교수가 위원장을, 가톨릭대학교 의과대학 김선 교수가 간사를 맡았으며, 영역별 책임 교수를 지정하고 영역별 담당위원이 미리 평가기준 안을 연구해 와서 발표한 후 모든 위원들이 심도 있게 토론해서 잠정적 안으로 채택하였다.

〈표 1〉 담당영역별 전문위원

| 담당 영역 | 전문위원 |
|---|---|
| 운영체계 영역 | 이영환 교수(영남대학교 의과대학) |
| 교육 영역 | 김 선 교수(가톨릭대학교 의과대학) |
| 학생영역 | 윤희상 교수(경상대학교 의과대학) |
| 시설 및 설비 영역 | 백선용 교수(부산대학교 의과대학) |
| 졸업 후 교육 영역 | 이정애 교수(전남대학교 의과대학) |

### (2) 실행과정 및 활동

평가기준개발 위원회는 2010년 3월 23일 제1차 회의를 시작으로 신임 이정애 위원장이 위원진을 소개하고 앞으로 활발한 활동을 당부하였고, 김선 교수를 위원회 간사로 선임하였다. 안덕선 원장이 가능한 2011년 상반기 내 평가기준 개발을 완료해 줄 것을 요청하였고, 위원진은 월 1회 이상 그리고 필요시 집중 워크숍을 하기로 결정하였다.

1차 회의(2010.3.23)에서 영역별 담당 위원을 결정하고 담당 위원이 책임지고 담당영역의 항목 및 기준 안을 마련해 오기로 하였다. 또한 평가인증 전문인력 양성 프로그램 워크숍을 5월에, 집중워크숍을 7월과 8월에, 서면·현지 방문평가자 워크숍을 9월에, 대학 자체평가위원 능력 함양을 위한 워크숍을 11월에 개최하기로 결정하는

등 첫 회의부터 전문위원들의 열의가 대단하였다. 3차 회의(2010.4.30)에서 필수, 권장기준의 구분이 모호하고, 권장기준이 거의 충족되었다고 판단되어, Post-2주기의 목적에 부합하도록 제2주기에서 필수와 모든 권장기준과 일부 우수기준을 기본기준으로 상향조절하고 대학의 특수성을 고려한 우수기준 항목을 신설하는 것을 토의하였다. 따라서 담당위원들이 각 영역별로 항목과 기준을 검토해서 7월 집중워크숍에서 결정하기로 하였다.

2010년 5월 25일에 개최한 제5차 평가인증 전문인력 양성 프로그램 워크숍은 안덕선 원장과 임기영 인증단장의 인사로 시작하여 국내 의과대학인증평가제도 및 세계적 동향에 대한 발표가 있었으며, 평가항목 및 기준에 대한 교육은 분임토의 및 Role-Play 형식으로 진행하였는데 참가자들로부터 좋은 반응을 얻었다.

2010년 7월 1일부터 3일까지 2박 3일로 여수 디오션 리조트에서 1차 집중 워크숍을 진행하였는데, 주요 쟁점은 교육목표와 교육과정 및 성과중심 교육에 관한 것이었다. 김선 교수가 교육과정 평가기준 개정 배경과 상황을 설명한 후, 교육영역의 기준 설정 원칙을 전체 교육과정을 포괄하는 의미의 명칭과 하부영역은 교육과정의 개발 절차에 따라 구성하자고 제안하였다. 따라서 영역 2의 '교육목표와 교육과정'을 '기본의학교육과정'으로 명칭 변경을 하였다. 또한 Post-2주기는 선진국과 같은 성과중심(outcome-based)교육 프로그램을 평가하는 기준을 개발하자는 의견에 전문위원들의 우려 목소리도 있었지만 심도 있는 토론을 통하여 성과중심 교육과정 프로그램을 위한 평가기준 개발을 하기로 결정하였다. 또한 2주기의 필수, 권장, 우수기준의 3단계 구분을 기본기준과 우수기준 2단계로 구분하기로 하였다. 기본과 우수기준은 국제기준(세계의학교육연합회 WFME, 미국의학교육합동위원회 LCME, 호주의학협회 AMC 등)에 대한 연구를 바탕으로 세계화, 국제화 시대에 우리나라 의과대학들이 선진국과의 경쟁력을 위한 다양한 교육프로그램과 교육여건을 평가할 수 있는 기준으로 상향조정하였다. 그리고 대학의 특성화를 위한 새로운 '우수기준' 등을 개발하기로 하였다. 그리고 8월 31일 2차 집중워크숍을 개최하여 평가항목 및 기준 개발(안)의 초안을 거의 완성하였다.

2010년 9월 13일 제1차 공청회와 11월 15일 제2차 공청회 후에 취합된 의견에 따라 평가기준(안)을 검토하고 최종 수정 보완 작업을 하였다. 공청회 이전에도 수시로 41개 의과대학으로부터 의견과 질의가 있었고 이에 대한 답변과 설명을 충실히 하였으며, 또한 한국의과대학장협회로부터 쟁점이 되는 항목 등에 대해서는 협의 조정의 과정을 거쳐 평가기준의 적용과 해석에 대한 신뢰성을 높이도록 노력하였다. 위원들 간의 진지한 토론을 통해서 기준에 대한 수정 보완 작업이 계속해서 이루어졌다.

2011년 3월 18일(제13차 회의)에는 교육부 고등교육 평가인증 인정기관 신청 관련 건에 대해 4월 1일부터 2일까지 대전 유성호텔에서 집중작업을 하여 교육부에서 요구하는 사항 등에 대한 보완자료 및 추가 자료를 정리하였다.

〈표 2〉 교육부 고등교육 평가인증 인정기관 신청 관련 건에 관한 검토요청 사항 (일부 예)

| 검토요청 사항 | 검토 내용 |
|---|---|
| - 정성평가의 최소한의 객관성을 확보할 수 있는 Rubric에 대한 제시 | - 평가가이드라인을 영역별로 구체적으로 제시 |
| - '충족과 불충족' 이분법적 판정방법으로 평가하는데서 오는 문제점 해소평가방안 | - 대교협의 '미흡' 용어를 받아들여 서면평가서식을 수정하고 가이드라인을 마련 |
| - 학습성과를 구체적으로 반영한 의과대학과 의학전문대학원의 인증 구분 방안 | - 의학전문대학원의 평가기준을 따로 마련할 필요성을 인정하고 일부 영역의 기준 수준을 검토 |
| - 평가위원 풀에 대한 수준별 교육과정으로 평가위원의 전문성 제고 방안 | - 수준별(기본, 고급과정) 평가위원 교육과정 마련 |

2011년 5월 20일(제16차 위원회)에서 학습성과 지침 개발 연구프로젝트를 위한 연구TFT팀(이정애, 김선, 윤희상, 이영환, 박주현, 박귀화, 채수진)을 구성하고 7월까지 집중작업을 통해 학습성과 지침 개발(안)을 마련하였다. 다음은 교육목표 및 학습성과의 체계도를 간략히 정리한 것이며 이 체계도는 이후 관련 기준에 전반적으로 적용되었다.

| | |
|---|---|
| **교육목적 및 목표** (Mission Statement) | 대학의 이념 및 특성화가 반영된 교육목표 |
| **졸업성과** (Graduation Outcome) | 교육목표의 세부 항목을 의미하며, 졸업 시 반드시 요구되는 성과 |
| **과정학습성과** (Phase Learning Outcome) | * 학년별 또는 기초의학, 임상의학, 의료인문학 과정별 학습성과<br>* 졸업성과를 각 과정별로 보다 세분화 한 성과 |
| **단위과정별 학습성과** (Course or Unit Learning Ourcome) | * 단위과정별(예를 들면, 인체의 정상구조, 성장과 노화, 소화기 등)로 구분되어있는 학습성과<br>* 과정학습성과를 보다 세분화 한 성과 |
| **수업학습성과** (Learing Ourcome) | 단위과정별 학습성과를 개별 수업에 적용한 보다 세분화된 성과 |

[그림 1] 교육목표 및 성과의 체계도

2011년 6월 24일 가톨릭의대 MASTER센터에서 제6차 평가전문인력 양성을 위한 워크숍을 실시하였다. 이번 워크숍의 목적은 평가인증에 참여한 경험이 없는 교원을 대상(초급 과정)으로 의학교육 평가인증 제도 및 기준에 대한 이해와 평가인증 전문인력의 확보를 위한 것이었는데 약 80명이 참여하여 성공리에 목적을 달성하게 되었다. 2011년 10월 25일 개최한 제19차 회의에서는 서면·현지 방문평가 가이드 전 영역에 대한 검토를 통해 마지막 작업에 착수하였다. 또한 2011년 10월 28일에는 의학교육 평가인증 개정 규정에 관한 소개 및 평가인증 개정 기준에 대한 설명회를 개최하여 대학이 원활한 평가인증 자체평가 준비를 할 수 있도록 노력하였다.

2012년 2월 8일에는 의평원(임기영 단장, 김영창 위원장, 김명곤 간사)과 KAMC (임정기 이사장, 상임이사 및 전문위원 등)와의 간담회를 실시하고 의학교육 평가인증 기준 및 대학 부담금 인상에 관한 회의를 하였다. 의과대학장협의회에서 요청한 검토 의견에 관한 항목(몇 가지 예)들은 다음과 같다.

〈표 3〉 의과대학장협의회에서 요청한 검토의견에 관한 항목의 예

| 검토요청 사항 | 검토 내용 |
|---|---|
| 2-2-5 학생교육 관련 직접비용에 대한 지원은 적절한가? | 의과대학장협의회와 협의에 따라 조정 가능함 |
| 3-2-3 대학은 학생의 학습평가, 유급, 졸업 사정과 징계 조치에 대한 공정한 기준이 있고 이를 학생과 교수에게 홍보하고 있는가? | 대학은 학칙에 따라 학생의 학습평가, 유급, 졸업 사정과 징계조치를 적절하게 시행하고 있다. |
| 3-3-2 대학은 학생들에 대해 적절한 재정지원 노력을 하고 있는가? | 대학은 학자금 융자 외에 학생의 재정관리 상담을 위한 체제와 실적이 있다. |
| 4-2-1 교수들의 국내 외 연구실적이 적절한가? | 전임교수 100명당 국내외 연구실적이 최근 2년 동안 연평균 1편이다. |
| 4-2-3 대학 내부 연구비 수준은 적절한가? | 우수기준은 그대로하고, 기본기준은 100만원으로 낮춤 |
| 5-1-5 대학은 임상실습 교육을 위하여 대학부속 교육병원을 확보하고 있으며, 병원 내에 학생교육 시설을 갖추고 있는가? | 병원당, 병원에 나가는 실습학생당 최소 몇 개의 공간이 필요하다는 의견을 고려하기로 함 |

2012년 2월 21일에 대한의사협회 동아홀에서 이윤성 이사장의 축사로 시작하여 2011년 평가인증 인증서 전달식과 더불어 2012년 평가인증 설명회를 동시에 실시하였으며, Post 2주기인 첫해인 2012년에 평가 신청한 대학은 총 5개교로 고려, 서울, 울산, 인하, 을지의과대학으로 확인되었다.

이를 정리하면 20회 이상의 자체회의, 3회의 집중워크숍 등을 통하여 초안을 마련하였고, 의과대학 및 의학전문대학원의 의견과 질문 그리고 의과대학장협의회에서 요구한 의견 등에 대하여 지속적으로 검토, 보완, 수정 및 답변 과정 등을 거쳐서 결정한 최종(안)에 대해 2회의 공청회를 통해 검토 및 의견수렴한 후 〈Post-2주기 의과대학 인증평가기준〉을 최종 확정하였다. 이를 2011년 1월 30일에 의평원 심포지엄을 개최하면서 동시에 개정된 평가기준 최종(안) 발표를 실시하였다.

### (3) 활동 경과

2010년 3월 이후 위원회의 주요 활동 경과는 다음과 같다.

〈표 4〉 평가기준개발 위원회의 주요 활동 경과

| 일 시 | 회의 내용 |
| --- | --- |
| 2010.03.23. | 제1차 회의: 신임 위원장 임명 및 간사와 영역별 담당 전문위원 결정, 1년 계획을 세움 |
| 2010.07.01.-03. | 1차 집중워크숍, 학습성과 중심 교육프로그램 평가기준 개발 |
| 2010.08.31. | 2차 집중워크숍, 평가인증 항목 및 기준 최종(안) 논의 |
| 2010.09.13. | 평가기준 개정 제1차 공청회 |
| 2010.11.15. | 평가기준 개정 제2차 공청회 |
| 2011.03.11. | 교육부 평가인증 인정기관 신청 관련 간담회 |
| 2011.04.22. | 영역별 서면·현지방문 평가 가이드라인(안) 검토 |
| 2011.06.24. | 제6차 평가전문인력 양성 워크숍(초급자 대상) 학습성과 관련 지침을 위한 연구프로젝트 논의 |
| 2011.10.28. | Post-2주기 의학교육 평가인증 개정기준에 대한 설명회 |
| 2012.02.08. | 의평원-KAMC 간담회 개최 |
| 2012.02.21. | 2011년 평가인증 인증서 전달식 및 2012년 의학교육 평가인증 규정 및 평가인증 기준 설명회 |
| 2012.09.06. | 2012년 서면·현지방문평가단 워크숍, 위촉장 수여, 모의 서면평가 및 분임토의 |

## 4. 결과

Post-2주기의 평가기준 항목이 크게 달라진 점은 교육과정과 교육목표를 '기본교육과정'으로 개념을 바꾸면서 성과중심 교육과정과 성과중심 평가체계를 적용한 것이다. 많은 학교가 성과중심 교육과정이란 용어에 혼란스러워해서 학습성과 지침 개발 연구프로젝트를 통해서 [그림 1]과 같이 정의하였다.

제1주기 평가기준 항목은 교육목표 및 교육과정, 학생, 교수, 시설·설비, 행정·재정 등 다섯 영역을 평가하였으며, 평가기준은 필수(must) 및 권장(should) 기준으로 구분하고 필수기준 18개, 권장기준 32개로 총 문항 수는 50개였다. 또한 제2주기에는 평가영역은 6개 영역으로 대학운영체계, 교육목표와 교육과정, 학생, 교수, 시설·

설비, 졸업 후 교육으로 나누었고, 필수(must), 권장(should), 우수(excellence) 기준 각각 41개, 34개, 34개로 총 75개 문항 수이다.

그러나 Post-2주기에는 한 개의 평가항목 속에 2개 이상의 내용을 평가하게 되어 있는 항목들은 따로 분리해서 더욱 정확하게 기준을 정리하였다. 2주기의 필수기준과 권장기준을 기본기준(basic)으로 하고, 제2주기의 우수기준 항목 중 일부도 기본기준(basic)으로 상향 조절하였고, 대학의 특수성을 나타낼 수 있는 새로운 우수기준(quality development) 을 개발하였다. 즉 평가 문항은 기본 97개 항목, 우수 43개 항목으로 총 100개 항목으로 늘렸으며, 또한 항목 평가에 대한 구체적인 가이드라인을 만들고, 이를 평가자 교육 및 현지방문 평가에 활용토록 하였으며, 새로운 가이드라인에 대해서는 평가개요, 보고서 기술내용, 참고자료 등으로 구체적으로 기술하도록 하였다. 〈표 5〉는 Post-2주기 의과대학평가인증 평가영역별 평가부분 및 문항 수를 2주기와 비교한 표이다.

〈표 5〉 Post-2주기 의과대학평가인증 평가영역별 평가부분 및 문항 수

| 평가영역 | 문항 수 | | 개정 기준 | |
|---|---|---|---|---|
| | 2주기 | Post-2주기 | 기본 | 우수 |
| 1. 대학운영체계 | | | | |
| 소계 | 16 | 18 | 18 | 4 |
| 2. 기본의학교육과정 | | | | |
| 소계 | 25 | 30 | 30 | 9 |
| 3. 학생 | | | | |
| 소계 | 12 | 19 | 19 | 13 |
| 4. 교수 | | | | |
| 소계 | 13 | 18 | 18 | 12 |
| 5. 시설·설비 | | | | |
| 소계 | 6 | 9 | 9 | 4 |
| 6. 졸업 후 교육 | | | | |
| 소계 | 3 | 5 | 5 | 3 |
| 합계 | 75 | 99 | 99 | 45 |

요약하면, 평가항목의 내용과 기준이 선진국 수준으로 향상되어 의학교육의 질이 좋아지는데 일조를 하였다. 또한 평가 전문인력양성 교육과 서면·현지방문평가단 워크숍 및 가이드라인 등을 통하여 평가에 참여하는 교수들의 적극적 참여와 평가자의 전문성이 향상되어 평가자 간의 평가오차를 줄일 수 있도록 노력하였다고 생각한다. 다음 평가항목과 기준을 개정할 경우에는 입학생과 졸업생의 특성에 대해 파악할 수 있는 평가기준 및 학생의 인성을 파악할 수 있는 평가기준 등이 개선되었으면 한다.

# 제4기 인증기준위원회(2013.3-2016.2): Post-2주기:2015~2018

윤희상 (제4기 인증기준위원회 위원장)

## 1. 배경/근거(Post-2주기:2015~2018)

2013년 5월 3일~4일 남원에서 개최된 의평원 합동워크숍에서 Post-2주기 평가인증기준에 관한 논의 중 resident as teacher, inter-professional education, 학장의 임용기준 등을 새 기준에 추가하는 의논이 있었다.

Post-2주기(2012~2014) 평가인증기준을 사용 중 2013년 9월 9일 교육부의 인정기관 지정 조사 연구사업을 수행하고 있는 대교협 고등교육연구소 연구지원팀에서 2012년 12월에 제출한 의평원 답변서에 대한 소위원회 검토의견 및 추가 보완 요구사항에 대한 회신을 요청하였다. 이에 한국의학교육평가원은 교육부 인정기관 지정을 받기 위한 TFT를 구성하여 대응하였다. 교육부의 보완요구사항 중에는 Post-2주기 평가인증기준(2012~2014)에 있는 정량적인 내용의 기준들은 이번 인정기관 지정 심의를 통하여 성과 중심의 교육과 관련한 정성적인 인증기준으로 개정하고, 그 입증자료를 제출하도록 하였다.

## 2. 목적/원칙

Post-2주기(2012~2014) 평가인증기준 중 정량적 평가인증 기준을 정성적 기준으로 바꾸고, 애매한 평가인증기준과 [주] 설명을 명확하게 하거나 불필요한 내용은 삭제하고 일부 내용을 추가하였다. 인증기준의 일부 용어를 교정하고 통일시켰다. 인증기준이 변경된 경우는 각 해당 기준에 적합하게 비치 자료 내용을 변경하였다. Resident-As-Teacher 내용을 “2-3-14 임상실습 책임교수가 지정되어 있고, 학생에

대한 지도감독과 피드백이 적절한가?”라는 우수기준에 추가하여 반영하였다.

## 3. 실행 과정/활동

한국의학교육평가원 제4기 의학교육인증단 인증기준위원회(윤희상, 김미경, 유효현, 이강욱, 이종태, 허정식, 홍승재)는 2013년 5월 3일 남원에서 개최된 의평원합동워크숍 인증기준위원회 회의에서 Post-2주기 평가인증기준을 2012년도 평가기관 및 평가단 등의 피드백, 의평원 홈페이지의 Q&A 내용 검토 등을 근거로 평가기준을 지속적으로 검토하기로 하였다.

2013년 10월 2일 개최된 의학교육인증단 제3차 운영위원회 회의에서 교육부 인정기관 지정 관련 건에 관한 논의를 하였고 교육부 인정기관 인정 및 WFME 인증기관 인정 관련 TFT를 만들어 집중작업을 수행하도록 결정하였다. 2013년 10월 16일 제1차 집중작업을 시작으로 제5차 집중작업까지 수행하여 교육부 인정기관 인정을 위한 평가인증기준 내용 수정을 수행하였다.

2014년 1월 23일 개최된 제4-2차 실행위원회에서 Post-2주기 평가인증기준 개정안이 승인되어 2015년부터 적용하기로 하였다.

## 4. 결과/성찰

Post 2주기(2012~2014) 평가인증 기준 97개 문항, 97개 기본기준과 43개 우수기준을 아래와 같이 수정 및 추가하여 97개 문항, 97개 기본기준과 44개 우수기준의 Post 2주기(2015-2018) 평가인증 기준을 만들었다.

기본기준 38개 항목, 우수기준 25개 항목(2-3-14 신규 1개)

| | | |
|---|---|---|
| 1-2-1 | 교육, 교수, 학생, 연구 분야를 포함한 최소 7개 분야에 보직자가 임명되어 있으며, 해당 보직자의 구체적인 활동 실적이 있다. | 교육, 교수, 학생, 연구, 졸업후교육, 입학, 재정 등의 분야에 각 보직자가 임명되어 있으며, 해당 보직자의 구체적인 활동실적이 있다. |
| 1-5-1 | 관련 위원회의 연간 예산이 5천만 원 이상이며, 위원들의 전문성을 확보하기 위한 지속적인 노력이 있다. | 관련 위원회의 연간 예산이 충분하며, 위원들의 전문성을 확보하기 위한 지속적인 노력이 있다. |
| 1-5-3 | 최소 4주 이상 참여하는 학생 교류가 입학정원 기준 10% 이상이거나, 외국대학에서 학점취득이 가능하며, 이에 대한 구체적인 실적이 있다. | 외국대학에서 학점취득이 가능하며, 이에 대한 구체적인 실적이 있다. |
| 2-2-3 | 교육과정위원회가 월 1회 이상 정기적으로 개최되고, 그 예산이 연간 5천만 원 이상이다. | 교육과정위원회가 정기적으로 개최되고, 충분한 예산을 확보하고 적절하게 집행하고 있다. |
| 2-2-5 | 학생교육 관련 직접비용이 연간 학생 1인당 등록금 대비 20%이상이다. | 연간 학생 1인당 등록금 대비 학생교육 관련 직접비용이 우수하다. |
| 2-3-11 | 최소 70주, 주당 36시간에 준하는 임상실습이 이루어지고 있다. | 최소 72주, 주당 36시간에 준하는 임상실습이 이루어지고 있다. |
| 2-3-14 | | 교육 담당 전공의의 교육자로서 역할, 임무 등을 명시한 관련 규정이 있으며, 핵심과목 임상실습을 담당하는 교육전공의에 대해서는 학생 지도 감독과 피드백 방법에 관련한 교육과 훈련을 정기적으로 실시하고 있다. |
| 2-4-3 | 시기별 학습성과와 졸업성과 종합평가를 모두 시행하고 있으며, 그 결과를 진급과 졸업사정에 활용하고 있다. | 시기성과와 졸업성과 종합평가를 모두 시행하고 있으며, 그 결과를 진급과 졸업사정에 활용하고 있다. |
| 3-3-1 | 등록금 전액 대비 장학금 지급비율이 연 평균 20% 이상이다. | 등록금 전액 대비 장학금 지급비율이 우수하다. |
| 3-4-1 | 임상의학 이외 분야로 진출한 경우가 최근 10년간 연간 입학정원의 5% 이상이다. | 임상의학 이외 분야로 진출한 경우의 실적이 우수하다. |
| 4-1-1 | 13개 기초의학분야 중 90% 이상에서 교육경력 10년 이상인 전임교수가 1인 이상이 있다. | 각 기초의학 분야별로 교육경력이 우수한 전임교수가 확보되어 있다. |
| 4-1-2 | 의학교육학 전임교수가 2명 이상이거나, 전임교수 1명과 전담교수 3명 이상이다. | 의학교육학 전임교수 또는 전담교수를 충분히 확보하고 있다. |

| | | |
|---|---|---|
| 4-1-3 | 의료인문학 관련 교실(학과, 센터 등)이 개설되어 있고, 의료인문학 전임교수가 2인 이상이다. | 의료인문학 관련 교실(학과, 센터 등)이 개설되어 있고, 의료인문학 전임교수가 충분히 확보되어 있다. |
| 4-1-4 | 기초의학, 의학교육학, 의료인문학의 교육과 연구를 보조하는 인력으로 조교 또는 대학에서 직접적으로 재정적 지원을 하는 연구원이 전임교수 1인당 1명 이상이다. | 기초의학, 의학교육학, 의료인문학의 교육과 연구를 보조하는 인력으로 조교 또는 대학에서 직접적으로 재정적 지원을 하는 연구원이 충분하다. |
| 4-1-5 | 임상진료와 지원과의 90% 이상에서 교육경력 10년 이상인 전임교수가 1인 이상이 있다. | 각 임상의학 전공과목별로 교육, 연구, 진료 경력이 우수한 전임교수가 확보되어 있다. |
| 4-1-6 | 대학의 전체 교수 중 동일 대학 출신 비율이 50% 이하이다. 그리고 여교수 비율이 30% 이상이거나 주임교수 공모제가 실시되고 있다. | 기초의학과 임상의학교실에서 주임교수 공모제가 실시되고 있다. |
| 4-2-1 | 전임교수 100명당 국내·외 연구 실적이 최근 2년 동안 연평균 200편 이상이다. | 전임교수의 국내외 연구 실적이 우수하다. |
| 4-2-2 | 전임교수 1인당 연평균 수혜 연구비가 2천만 원 이상이다. | 전임교수의 최근 2년간 연평균 외부 연구비 수혜가 우수하다. |
| 4-2-3 | 전임교수들이 최근 2년간 대학 내부로부터 받은 연구비가 전임교수 1인 당 연평균 5백만 원 이상이다. | 전임교수의 최근 2년간 대학 내부로부터 받은 연구비가 우수하다. |
| 4-3-1 | 교수 업적 평가가 직급별(연구전임, 조교수, 부교수, 교수), 기능별(기초의학, 임상의학, 의료인문학, 의학교육학 등)로 세분화되어 있고, 직위승진에 관한 규정이 세분화되어 있고 별도의 업적 평가제도가 있다. | 교수 업적 평가가 직급별(연구전임, 조교수, 부교수, 교수), 기능별(기초의학, 임상의학, 의료인문학, 의학교육학 등)로 세분화되어 있고, 직급승진에 관한 규정이 세분화되어 있고 별도의 업적 평가제도가 있다. |
| 4-3-2 | 교수 업적으로 승진이 가능한 교수업적평가 규정이 있고, 강의식 이외의 학생교육과 관련된 교육활동(PBL, TBL 등 다양한 교육프로그램)에 대하여 인센티브제도가 마련되어 있다. 의학교육 관련 연수교육에 최근 2년 동안 연간 3시간 이상 참석한 교수 비율이 80% 이상이다. | 교수 업적 평가규정에 의학교육 연수 실적이 의무화되어 있으며, 교육업적으로 승진이 가능한 교수업적평가 규정이 있고, 강의식 이외의 학생교육과 관련된 교육활동(PBL, TBL 등 다양한 교육프로그램)에 대하여 인센티브제도가 마련되어 있다. 의학교육 관련 연수교육에 최근 2년 동안 참여한 실적이 우수하다. |
| 4-3-5 | 교수들의 장·단기 외국연수와 국내외 학회참석을 위한 안정적 재정을 확보하고 있으며, 학회참석을 독려하기 위해 교수 1인당 연간 5백만 원 이상 지급하고 있다. | 교수들의 장·단기 외국연수와 국내외 학회참석을 위한 재정적 지원이 우수하다. |

| | | |
|---|---|---|
| 5-1-6 | 매년 지출되는 예산이 교수와 학생 1인당 150만 원 이상이고, 전문 인력(의학 전문 사서) 1인이 담당하는 교수와 학생이 150명 이하이다. | 매년 지출되는 예산과 전문인력(의학전담 사서)이 충분하다. |
| 5-2-1 | 전임강사 이상 교원의 개인 교수실 확보 수준이 100%이고, 기초 및 임상 교실(과) 당 1명 이상의 행정업무 지원인력이 있다. | 전체 교수가 개인 교수실을 확보하고 있다. |
| 6-1-3 | 교내·외 장학금 또는 특별지원금이 등록금 총액 대비 30% 이상이며, 대학원생 전용 연구공간이 있다. | 교내·외 장학금 또는 특별지원금이 등록금 총액 대비 우수하며, 대학원생 전용 연구공간이 있다. |

## 5. 발전방향

의평원의 기본방침은 가능한 정량적 기준은 지양하려고 계획하고 있다. 우리나라의 평가인증 경험상 정량적 평가에서 바로 정성적 평가로 바꾸어 진행하는 것에 따른 부작용을 줄이기 위하여 단계적으로 정량적 평가 기준을 축소하고자 하였다. ASK2019 평가인증기준은 정성적 평가가 주가 된다.

# 제4기 인증기준위원회(2013.3-2016.2): WFME 기준 적용 준비

## 1. 배경/근거(WFME 기준 적용)

세계화, 국제화의 흐름에 따라 고등교육에 대한 평가 및 인증의 국가 간 연계 필요성이 증대하고 있고, 국내학위의 국제 인증 및 외국 학위의 국내 인증을 위하여 국제적 기준의 대학평가기준 개발이 요구되는 상황이다. 이러한 국제적 추세에 부응하여 의료와 관련 인력의 국제화를 촉진하고 의료행위의 최소 수준을 보장하여 의료 인력의 국제적 활용도를 높이기 위하여 WFME를 중심으로 의학교육의 국제표준화가 추진되었다.

WFME는 의과대학과 의학교육과정의 국가적 또는 국제적 평가인증 및 평가인정 체제를 구축하여 의학교육과정에 대한 최소한의 질적인 기준을 보장하고, 의학교육에 대한 책임을 가진 정부, 기구, 기관 등이 평가인증기준에 따른 의학교육의 질을 향상시키고 변화시키는 계획을 수립하도록 촉진하고, 의학교육과 의료 인력의 국제화를 증가시키고, 의료 활동의 최소 수준을 보장하여 의료 인력의 활용도를 높이고자 하였다.

우리나라도 가시화되고 있는 의료시장의 개방을 앞두고 국내 의과대학과 의학교육과정의 국제적 인정 필요성이 더욱 높아지고 있기 때문에 우리나라 의과대학 평가인증 제도가 국제 사회의 인정을 받을 수 있도록 체계적으로 준비하고 실행하는 것이 필요하게 되었다.

## 2. 목적/원칙

### 목적

한국의학교육평가원(KIMEE, Korean Institute of Medical Education and Evaluation)은 기본의학교육의 질적 향상을 목적으로 세계의학교육연합회(WFME,

World Federation for Medical Education)에서 제시한 Basic Medical Education WFME Global Standards for Quality Improvement (The 2015 Revision)를 근간으로 우리나라의 기본의학교육 상황을 고려하여 2018년부터 사용할 의학교육평가인증 기준을 개정한다.

## 원칙

1. 전체적인 구조와 구성은 WFME Global Standards와 동일하게 개정한다.

▹ 평가영역과 평가부문

; The WFME Global Standards는 9개 평가영역에 대한 36개의 평가부문으로 구성되어 있어, 이 형식과 동일하게 개정한다.

- 평가영역(평가부문 수): 1. 사명과 성과(4), 2. 교육과정(8), 3. 학생평가(2), 4. 학생(4), 5.교육과 연구지원 직원/교수진(2), 6. 교육자원(6), 7. 교육평가(4), 8. 거버넌스와 행정(5), 9.지속적인 개혁(1)

▹ 평가기준의 종류

; The WFME Global Standards는 기준을 기본기준(B, Basic standards)과 우수기준(Q, Quality development standards)으로 구분하고 있다. 우리나라도 기준을 기본기준과 우수기준으로 구분하고, 기본기준은 K (Korea basic standard), 우수기준은 H (High quality development standards)로 표현한다.

- 기본기준은 의과대학과 기본의학교육이 충족해야 하는 기준으로 인증의 목적에 해당한다.
- 우수기준은 의과대학과 기본의학교육의 바람직한 미래지향적인 기준으로, 국제적으로 합의된 모범 사례에 따르도록 하는 의학교육 개혁이 목적이다.

▹ 주(Annotations)

; 주(Annotations)는 용어의 이해를 돕기 위한 예를 들거나 설명을 위한 것으로 평가부문별로 마지막에 제시한다.

2. 평가기준은 WFME Global Standards를 우리나라 의학교육 상황에 적절하도록 개정한다.

1) WFME 기준과 Post-2주기 KIMEE 기준 간의 균형을 유지한다.

2) WFME 기준 중 우리나라 의학교육 상황에 맞지 않는 것은 사용하지 않는다.

3) WFME 기준 중 일부는 우리나라 의학교육 상황에 맞게 Post-2주기 KIMEE 기준에 맞추어 추가 또는 수정한다.

4) KIMEE 의학교육평가인증 기준 중 기본의학교육과정이 아닌 것은 사용하지 않는다.

5) 우리나라에서 법적으로 규정되어 있는 것은 평가기준에 포함시키지 않는다.

3. 실행 과정/활동

한국의학교육평가원 제4기 의학교육인증단 인증기준위원회(윤희상, 김미경, 유효현, 이강욱, 이종태, 허정식, 홍승재)는 2013년 3월 26일 첫 회의에서 위원별로 담당 영역의 WFME 기준과 Post-2주기 평가인증기준 비교표를 작성하는 것을 시작으로 2018년 WFME 국제기준 도입을 위한 준비를 시작하였다.

이후 정규 인증기준위원회 회의 및 집중 작업을 위한 내부워크숍(2014년 2회, 2015년 6회)을 통하여 WFME 평가인증기준 한글화 작업, WFME와 Post-2주기 평가인증기준 비교, 우리나라의 기본의학교육 상황에 적합한 평가인증기준안 개발을 수행하였다.

이런 일련의 작업을 통하여 2019년부터 사용할 의학교육평가인증 기준안을 2015년 11월 14일~15일 2015년 5차 내부워크숍에서 마무리하였고 2015년 11월 24일 의학교육 평가인증기준 개정(안) 설명회에서 1st version을 공개하였다.

# 부록2: 시기별 평가인증 기준

# 제1주기 의과대학 인정평가기준

## 필 수 항 목

1. 대학은 기술된 교육목표를 가지고 있는가?
   [필수조건] 기술된 대학교육목표
2. 인체의 기본구조와 기능 및 병인을 이해하는데 필요한 기초의학이 교육과정에 포함되어 있는가?
   [필수조건] 기초의학 8개(해부학, 생리학, 생화학, 병리학, 약리학, 미생물학, 기생충학, 예방의학) 관련 분야 교육내용 개설
3. 기초의학 관련 실습은 있는가?
   [필수조건] 기초의학 관련 실습(예: 해부, 조직, 병리, 생리, 생화학 등)
4. 임상실습이 충분한 기간 실시되고 있는가?
   [필수조건] 총 40주, 1,400시간(주당 35시간) 이상의 임상의학 실습
5. 임상실습 지침서를 학생들에게 제공하고 있는가?
   [필수조건] 일차의료 수준의 임상실습 지침서 제작 및 배포
6. 의학과 교육과정 중 의료윤리 등 의학과 관련된 인성교육 과목(인간관계론, 의학과 사회 등)이나 현장 체험 교육 프로그램을 최소한 1개 이상 개설하고 있는가?
   [필수조건] 의학 및 의료관련 인문사회계열 과목 최소 1개 이상 개설
7. 구체적이고 행동단어로 기술된 과목별 학습목표를 가지고 있는가?
   [필수조건] 학습목표가 반영된 강의계획서
8. 대학당국이나 학생들에 의한 교수평가 또는 강좌평가가 이루어지고 있는가?
   [필수조건] 대학당국이나 학생들에 의한 교수평가 또는 강좌평가
9. 대학의 교육목표 및 교육과정 개선을 위한 연구기구가 있는가?
   [필수조건] 교육목표 및 교육과정 개선을 위한 전담연구기구 또는 위원회 구성
10. 대학은 의과대학생의 학습 및 생활지도를 위한 지도교수체제를 갖고 있는가?
    [필수조건] 학습 및 생활지도를 위한 지도교수체제 확립
11. 학생 장학제도가 있는가?
    [필수조건] 학내외 장학제도 및 내용

12. 임상의학 담당 교원이 적정 수 확보되어 있는가?
   [필수조건] 20개 이상 진료 및 지원과목에 교수가 총 85명 이상, 각 과에는 10년 이상 교육경력 교원이 최소한 1명 이상
13. 최근 2년간 교수들의 국내 연구실적은 적절한가?
   [필수조건] 교수 1인당 국내 연구실적 평균이 1.0 이상
14. 교수업적평가(교육, 연구, 봉사)가 이루어지고 있는가?
   [필수조건] 교수업적 평가실시
15. 강의실과 실험실습실을 적정 수 확보하고 있는가?
   [필수조건] 의학과 전용 강의실 4개(의학과 3, 4학년용 병원내 강의실 포함)와 기초의학 실험실습실 3개 확보
16. 임상실습을 위한 병원을 적절하게 확보하고 있는가?
   [필수조건] 500병상 이상의 독립 교육병원 확보
17. 의과대학생을 위한 도서관이 있는가?
   [필수조건] 의과대학생을 위한 도서관 확보
18. 대학의 발전계획이 수립되어 있는가?
   [필수조건] 기술된 의과대학 발전계획서

## 권 장 항 목

1. 대학은 교육목표의 내용 속에 대학의 교육이념이나 특성화 의지를 반영하고 있는가?
2. 대학교육목표는 구성원들에게 잘 인지되어 있으며, 대학은 인지도를 높이기 위해 적절한 노력을 하고 있는가?
3. 대학의 교육목표가 실제 교육과정에 적절하게 반영되고 있는가?
4. 인체의 기본구조와 기능 및 병인을 이해하는데 필요한 기초의학 교육내용과 시간은 적절한가?
5. 임상의학 강의는 기본적인 일차 진료수준의 의료를 목표로 구성되어 있는가?
6. 임상실습 지침서의 내용은 학습목표와 관련한 기본수기 내용을 적절하게 반영하고 있으며, 그 내용이 실제 실습되고 있는가?
7. 임상실습을 위한 장소와 방법이 기본적인 일차의료수준의 교육이 되도록 다양하게 이루어져 있는가?

8. 기초의학과 임상의학의 연계교육이 적절히 이루어지고 있는가?
9. 구체적이고 행동적으로 기술된 과목별 학습목표가 적절히 활용되도록 노력하고 있는가?
10. 강의 이외에 문제해결 능력 향상을 위한 토론이나 발표가 수업에 다양하게 활용되고 있는가?
11. 교수 또는 교과목 평가의 결과가 적절히 수업에 반영되고 있는가?
12. 학생들의 학습평가가 필기시험이외에 과제물, 중간시험, 구두시험, 출석 등 다양한 방법을 통하여 적절하게 이루어지고 있는가?
13. 기초 및 임상과목별(또는 강의별) 학생평가가 적절하게 이루어지고 있는가?
14. 기초의학 실습과제물이 규칙적으로 주어지고 다음 수업에 반영되는 등 관리가 적절하게 이루어지고 있는가?
15. 임상실습평가는 지식, 태도 및 수기 등의 능력을 적절하게 평가하고 있는가?
16. 대학의 교육과정 개선 노력이 적절한가?
17. 최근 2년간 의학교육 평가나 개선 작업을 위해 지출한 예산(회의, 연수, 자료발간 등 - 단, 학생교재나 시험비용 제외)은 적절한가?
18. 학생생활지도 체제와 운영이 전문적으로 이루어지고 있는가?
19. 학생들의 건전한 자발적 학내외 활동이 적절하며 이에 대한 대학의 지원, 지도 체계가 적절한가?
20. 학생의 장학금 수혜액과 수혜학생 비율은 적절한가?
21. 재학생들의 기숙사 입실비율은 적절한가?
22. 학생복지 시설(기숙사, 식당, 매점, 운동시설 등) 및 제도(건강관리 등)가 고루 갖추어져 있고 그 수준도 적절한가?
23. 최근 5년간 학생들이 졸업 후 임상의학 이외 다른 분야에도 적절히 진출하고 있는가?
24. 최근 2년간 의사국가시험 합격률은 적절한가?
25. 적정수의 기초의학 교원을 확보하고 있는가?
26. 기초의학 교육과 연구를 위한 조교의 수는 적절한가?
27. 최근 2년간 대학 전체(교수 100명당 등재 논문 수)로 SCI(SCIE 포함) 및 Index Medicus 등재 논문수가 적절한가?
28. 최근 2년간 학외로부터 받은 연구비 수준은 적절한가?
29. 대학은 학내 의학교육 관련 교수연수기회를 적절히 마련하고 있는가?
30. 대학은 교수들의 해외학회 참석을 위한 재정적 지원체제(등록비, 항공료, 체제비

포함)를 갖고 있는가?

31. 교수업적평가(교육·연구·봉사)의 결과가 적절하게 활용되고 있는가?
32. 강의실과 실험실습실의 설비 및 관리실태가 적절한가?
33. 임상실습을 위한 병원 내에 학생교육 시설을 갖추고 있는가?
34. 의과대학생을 위한 도서 확보 및 운영실태는 적절한가?
35. 의과대학생을 위한 교육지원시설, 즉 전산실, 시청각 교재실, 자율학습실, 소그룹 토의실(PBL실 포함) 등이 적절히 갖추어져 있는가?
36. 전임강사 이상 전체 교원의 개인 교수실이 있고, 그 실내 설비는 잘 되어 있는가?
37. 기초 및 임상교수들의 연구시설 공간과 설비가 적절한가?
38. 대학 행정업무는 교무, 학생, 연구 및 서무 등 업무별로 구분되어 있으며, 인력의 확보는 적절한가?
39. 대학 행정업무 계획 및 예산집행에 관한 학장의 자율권과 책임경영 체제가 대학 예산과 학사계획 범위 내에서 확보되어 있는가?
40. 대학 운영과 관련한 의견수렴 기구(위원회 포함)가 구성되어 있고 적절하게 운영되고 있는가?
41. 대학운영을 위한 예산과 최근 2년간 예산변동 상황은 적절한가?
42. 학생 1인당 기초의학 실험실습비가 적정한가?
43. 대학의 발전 계획의 실현을 위한 대학본부와 재단의 의지와 노력은 적절한가?
44. 의과대학 발전을 위한 발전기금 규모와 모금계획은 적절한가?
45. 대학발전을 위한 동문 및 지역사회 등의 참여가 구체적이며 그 참여정도는 적절한가?

### 참고사항

1. 필수항목의 인력 및 시설 기준은 신설의과대학 준칙 최종안을 참고하였음.
2. 하나의 평가항목에 필수와 권장항목이 같이 포함되어 있는 경우는 필수와 권장항목으로 구분하여 제시하였음.

## 평가영역별 필수 및 권장 항목 수

| 영 역 | 부 문 | 문항 수 | |
|---|---|---|---|
| | | 필수 | 권장 |
| 교육목표 및 교육과정 | 교육목표의 구성과 교육과정 반영노력 | 1 | 2 |
| | 기초의학 교육과정 | 2 | |
| | 임상의학 교육과정 | 2 | 2 |
| | 기초의학 및 임상의학 연계교육 | | 1 |
| | 인성관련 교육과정 강좌 개설 | 1 | |
| | 수업방법과 강좌평가 | 2 | 1 |
| | 학생 학습평가 | | 4 |
| | 교육목표 및 교육과정 개선 노력 | 1 | 1 |
| | 소 계 | 9 | 11 |
| 학 생 | 학생지도 체제 | 1 | 1 |
| | 학생복지 제도 및 시설 | 1 | 2 |
| | 졸업 후 진로 및 학습성과 | | 2 |
| | 소 계 | 2 | 5 |
| 교 수 | 기초 및 임상교원 확보 | 1 | 2 |
| | 교수연구 및 학술활동 | 1 | 2 |
| | 교수개발 실태 | 1 | 2 |
| | 소 계 | 3 | 6 |
| 시설 · 설비 | 교육기본 및 지원 시설 | 3 | 1 |
| | 교수 시설 | | 2 |
| | 소 계 | 3 | 3 |
| 행정 · 재정 | 대학행정 및 운영 체계 | | 3 |
| | 대학재정 | | 2 |
| | 대학발전 계획 | 1 | 2 |
| | 소 계 | 1 | 7 |
| 합 계 | | 18 | 32 |
| | | 총 50 | |

# 평가영역별 자체평가연구 지침

## 1. 교육목표 및 교육과정 영역

### 1-1. 교육목표의 구성과 교육과정 반영 노력

| 평 가 기 준 |
| --- |
| 의과대학의 교육목표는 대학의 교육이념이나 특성이 잘 반영되고 의대 졸업생의 수행능력 범위와 임무 수준이 명료하게 기술되어 있어야 하며, 실제 교육과정에 구체적으로 반영되어야 한다. 또한 대학교육목표는 대학의 구성원들에게 잘 인지되어 있어야 한다. |

**1. 대학은 기술된 교육목표를 가지고 있으며(필수), 그 내용 속에 대학의 교육이념이나 특성화 의지를 반영하고 있는가?**

**[지침]** 교육목표 내용을 기술하고 대학이 이런 교육목표를 정하게 된 과정과 이유를 기술한다. 이와 관련해서 대학 설립자의 교육이념이나 특성화 의지를 함께 기술하는 것도 바람직하다. **[필수조건]** 기술된 대학교육목표

**2. 대학교육목표는 구성원들에게 잘 인지되어 있으며, 대학은 인지도를 높이기 위해 적절한 노력을 하고 있는가?**

**[지침]** 대학교육목표가 구성원들에게 잘 인지되어 있는지와 이를 위한 대학당국의 노력 내용을 기술한다(예: 공지, 세미나, 연수 등).

**3. 대학의 교육목표가 실제 교육과정에 적절하게 반영되고 있는가?**

**[지침]** 대학교육 목표로 기술된 내용들이 대학교육 과정에 어떤 내용으로 어떻게 반영되어 있는지를 교육목표 항목별로 기술한다. 즉, 교육목표 하나 하나에 대해서 이들이 어떤 교과목 또는 어떤 교육 프로그램을 통해 실천되고 있는지를 기술한다.

**▢ 교육목표의 구성과 교육과정 반영 노력 평가자료 ▢**

1. 대학교육목표
2. 개설교과목 및 대학교육목표 관련 교육내용

## 1-2. 기초의학 교육과정의 적절성

| 평 가 기 준 |
| --- |
| 의과대학 교육과정 중에 인체의 정상 구조와 기능, 그리고 질병의 발생원인과 그 과정에 관한 제반 기초의학 지식과 질병발생 관련 요인에 대한 질병역학적 지식 및 기본적인 실험수기를 일정수준 습득할 수 있도록 과목간의 균형은 물론 적절한 내용과 시간이 배정되어야 한다. |

**1. 인체의 기본구조와 기능 및 병인을 이해하는데 필요한 기초의학 내용이 교육과정에 포함되어 있으며(필수), 그 내용과 시간은 적절한가?**

**[지침]** 인체의 기본구조와 기능 및 병인을 이해하는데 필요한 기초의학 교과목, 즉 해부학(조직학 포함), 생리학, 생화학, 병리학, 약리학, 미생물학, 기생충학, 예방의학 및 기타 기초의학 관련 교과목이 어느 학년에 어떻게 개설되어 있는지 그 내용을 기술한다. 이들 내용이 통합강의 또는 문제바탕학습(PBL)에 포함된 경우 어디에 어떻게 어느 정도 포함되어 있는지와 그 적절성 여부를 기술한다. **[필수조건]** 기초의학 8개(해부학, 생리학, 생화학, 병리학, 약리학, 미생물학, 기생충학, 예방의학) 관련 분야 교육내용 개설

**2. 기초의학 관련 실습이 있으며(필수), 그 시간과 내용은 적절한가?**

**[지침]** 기초의학 관련 실습이 강의 내용을 이해하는데 도움이 되도록 시간이나 내용이 적절히 구성되어 있는지 실습 단원별 시간표와 함께 기술한다. **[필수조건]** 기초의학 관련 실습(예: 기초의학교 과목별 또는 통합실습과정 내용)

**▢ 기초의학 교육과정의 적절성 평가자료 ▢**

1. 교육과정 시간표
2. 실습 및 리포트 교재(비치)
3. 교실별 실습담당 교수 및 실습 지도계획

1-3. 임상의학 교육과정의 적절성

| 평 가 기 준 |
| --- |
| 의과대학 임상의학 교육과정은 일차 수준의 전인적 의료문제 해결에 필요한 기본 임상 지식과 기술이 학습되도록 그 내용과 시간배정이 적절히 이루어져야 한다. |

**1. 임상의학 수업은 기본적인 일차진료수준의 의료를 목표로 구성되어 있는가?**

[지침] 임상의학 수업이 어떻게 이루어지고 있으며, 교육내용이 학부교육 수준(예를 들어, 한국의과대학장협의회에서 채택한 대한의학회 제정 학습목표 A 수준)으로 구성되어 있는지와 실제 수업내용이 이에 일치하는지를 기술한다.

**2. 임상실습이 충분한 기간 실시되고 있는가?(필수)**

[지침] 임상실습 기간을 실습과목 또는 통합교육 주제별로 기술한다. [필수조건] 총 40주, 1,400시간(주당 35시간) 이상의 임상의학 실습

**3. 임상실습 지침서를 학생들에게 제공하고 있으며(필수), 그 내용에 학생들이 습득해야 할 기본수기 등이 잘 포함되어 있는가? 그리고 그 내용이 실제 실습되고 있는가?**

[지침] 학생들에게 제공하는 임상실습 지침서가 어떤 내용으로 만들어져 있는지를 밝히고 임상실습지침서 안에 포함된 기본 수기들이 어떤 것인지, 그리고 그 내용이 실제 실습에 어떻게 활용되고 있는지를 기술한다. [필수조건] 일차의료 수준의 임상실습 지침서 제작 및 배포

**4. 임상실습을 위한 장소와 방법이 기본적인 일차의료수준의 교육이 되도록 다양하게 이루어져 있는가?**

[지침] 임상실습 장소나 방법이 기본적인 일차진료 수준이 되도록 적절히 고려되고 있는지를 관련 자료와 함께 기술한다(3차 병원 내에서의 실습만으로는 일차수준의 의료교육실습이 어렵다는 것이 정론임).

**▢ 임상의학 교육과정의 적절성 평가자료 ▢**

1. 임상의학 강의 및 실습 시간표와 실습교육 관련 자료
2. 임상실습 지침서(비치)

## 1-4. 기초의학 및 임상의학 연계교육의 적절성

| 평 가 기 준 |
| --- |
| 최근의 의학교육은 기초의학과 임상의학 교육을 서로 연계함으로써 환자의 임상적 문제를 종합적으로 이해하고 이를 해결하는 능력을 키우는 방향으로 발전하고 있다. 따라서 대학은 여러 가지 형태로 기초의학과 임상의학 교육을 연계하는 교육과정을 연구 발전시켜야 한다. |

**1. 기초의학과 임상의학 교육의 연계방법과 내용은 적절한가?**

[지침] 기초의학과 임상의학 교육을 연계하기 위한 대학의 노력과 실천 내용을 기술한다. 기초 및 임상과목을 연계 통합과정으로 운영하는 대학은 통합교육 내용을 기술하고, 기초의학과 임상의학을 분리 교육하는 대학은 기초의학 교육과 임상의학 교육에서 각각 임상의학과 기초의학 관련 내용을 어떻게 보완적으로 교육하는지를 기술한다.

## 1-5. 인성관련 교육과정 강좌 개설의 적절성

| 평 가 기 준 |
| --- |
| 의학은 자연과학적 사고나 내용 교육만으로는 교육목표 달성이 어렵다. 따라서 의학 교육과정에 인성을 위한 교육과 체험 활동이 포함되어야 한다. |

**1. 의학과 교육과정 중 의료윤리 등 의학과 관련된 인성관련 교육 과목(인간관계론, 의학과 사회 등)이나 현장 체험 교육 프로그램을 최소한 1개 이상 개설하고 있는가? (필수)**

[지침] 정규 의학교육 과목 이외에 의학과 학생들을 위해 개설한 인성관련 과목 또는 현장 실습 교육과정을 열거하고 과목별로 교육목표와 내용을 기술한다. 이런 교육에 대한

학생들의 반응도 조사된바 있으면 기술한다. 의예과 교육에서 제공되는 인성 관련 과목에 대해서 함께 기술하는 것도 도움이 된다. [필수조건] 의학 및 의료관련 인문사회계열 과목 최소 1개 이상 개설

**▢ 인성관련 교육과정 강좌 개설의 적절성 평가자료 ▢**

1. 교육과정표
2. 인성관련 교육과정 자료

## 1-6. 수업과 강좌평가의 적절성

평 가 기 준

학생수업은 학생들의 학습효과를 최대한 높이기 위한 방법으로 강의계획서와 학습목표를 적절히 활용해야 하고 강의 이외에 문제해결 능력 향상을 위한 토론식 수업과 교육보조 자료를 유용하게 사용해야 한다. 한편, 수업은 교수자신 또는 학생들에 의해서 평가되고 그 결과를 적절하게 활용하여야 한다.

**1. 구체적이고 행동단어(Action Verb)로 기술된 과목별 학습목표를 가지고 있으며(필수), 대학은 이것이 적절히 활용되도록 노력하고 있는가?**

[지침] 대학에서 사용하고 있는 과목별 또는 강좌별 학습목표를 제시하고 이 학습목표가 잘 활용될 수 있도록 하기 위해 대학이 어떤 노력을 하고 있는지를 기술한다. [필수조건] 학습목표가 반영된 강의계획서

**2. 강의식 수업 이외에 학습효과를 높이기 위한 문제바탕학습(PBL)이나 토론 및 발표 수업이 다양하게 활용되고 있는가?**

[지침] 전통적인 집단강의 이외에 사용하고 있는 기타 교육방식들(PBL, 토론, 컴퓨터 보조학습, 발표, 자료실 운영 등)의 내용을 과목별 또는 강좌별로 기술한다. (참고; OSCE는 1-7-3 "임상실습평가"에 기술함)

**3. 대학당국이나 학생들에 의한 교수평가 또는 강좌평가가 이루어지고 있으며(필수), 그 결과가 적절히 수업에 반영되고 있는가?**

[지침] 대학당국이나 학생들에 의한 교수평가 또는 강좌평가 내용을 관련 서식과 함께 제시하고 평가결과의 활용내용을 기술한다. [필수조건] 대학당국이나 학생들에 의한 교수평가 또는 강좌평가 실시

**▢ 수업과 강좌평가의 적절성 평가자료 ▢**

1. 강의계획서(비치)
2. 교육보조자료 일람표
3. 소그룹 토의 수업(문제바탕학습 등) 실적
4. 대학당국이나 학생들에 의한 교수평가 또는 강좌평가서 및 개선 실적

## 1-7. 학생 학습평가의 적절성

| 평 가 기 준 |
| --- |
| 학습평가는 학습목표의 달성여부를 측정ㆍ평가할 수 있도록 그 원칙과 방법이 신뢰롭고 타당해야 하며 지식, 태도, 수기 및 행동영역을 평가할 수 있도록 전 교육과정을 통하여 체계적이고 다양한 방법으로 실시되어야 한다. |

**1. 학생들의 학습평가가 필기시험 이외에 과제물, 중간시험, 구두시험, 출석 등 다양한 방법을 통하여 적절하게 이루어지고 있는가?**

[지침] 학생들의 학습능력 평가내용(평가형태, 빈도, 내용, 비중 등)을 과목별로 기술하고, 이들 평가방식에 대한 학생들의 반응을 기술한다.

**2. 기초의학 실습과제물이 규칙적으로 주어지고 교수나 조교에 의해 개별 평가되는 등 관리가 적절하게 이루어지고 있는가?**

[지침] 기초의학 관련 과목 실습과제물의 형태, 빈도, 과제물 평가 및 활용 상태를 과목 또는 강좌별로 기술한다.

**3. 임상실습평가가 지식, 태도 및 수기 등의 능력에 따라 고르게 평가되고 있는가?**

[지침] 임상실습에서 학생들의 지식, 태도 및 수기능력을 그 영역별로 어떻게 평가하고 있는지 과목별로 기술한다.

**4. 기초의학 및 임상의학 교육성취도에 관한 종합평가 노력은 적절한가?**

[지침] 과목 또는 강좌별 평가 이외에 기초의학이나 임상의학을 전반적으로 이해하고 있는지를 평가하는 기초종합평가나 졸업시험 형태의 임상종합평가가 실시되고 있는지와 이것이 있는 경우 그 내용 및 효과를 기술한다.

**▢ 학생 학습평가의 적절성 평가자료 ▢**

1. 과목별 학습평가방법 일람표
2. 임상실습 평가표 및 평가방법(지식, 태도 및 수기평가) 관련자료
3. 기초 및 임상의학 교육 평가자료(비치)

## 1-8. 교육목표 및 교육과정 개선 노력의 적절성

| 평 가 기 준 |
| --- |
| 대학은 교육목표 및 교육과정 개선과 발전을 위한 조직을 운영하고 실제 교육과정 개선활동을 활발히 전개해야 한다. |

**1. 대학의 교육목표 및 교육과정 개선을 위한 연구기구가 있고(필수), 그 개선 노력이 적절한가?**

[지침] 대학의 교육목표를 검토하고 교육과정 개선을 위한 별도의 학과나 교실이 있는지와 위원회 성격의 연구기구가 있는지, 그리고 이들이 있는 경우, 그 구성 및 운영내용을 기술한다. 단, 교육목표 개선 노력은 목표제정 이후 개선노력 내용을 기술하고 향후 개선의 필요성과 계획이 있는지를 기술한다. 교육과정 개선 노력은 최근 2년 간의 노력을 실적과 함께 기술한다. [필수조건] 교육목표 및 교육과정 개선을 위한 전담연구기구 또는 위원회 구성

**2. 최근 2년간 의학교육 평가나 개선 작업을 위해 지출한 예산(회의, 연수, 자료발간 등)은 적절한가?**

[지침] 최근 2년간 학내 의학교육 관련 평가나 개선을 위한 회의 및 연수, 그리고 이를 위한 자료발간 등에 지출한 비용 내용을 근거자료의 제시와 함께 시행 연, 월, 일 별로 정리하여 기술한다. 단, 일반 교수연수 등에 포함시킨 의학교육 관련 발표 등을 위한 비용은 해당 강의, 강사료만 인정한다.

**▢ 교육목표 및 교육과정 개선 노력의 적절성 평가자료 ▢**

1. 교육과정 연구기구 및 관련 서류
2. 연구기구 회의 및 활동 실적
3. 의학교육 관련 평가 또는 개선 작업을 위해 지출한 예산 자료

## 2. 학생 영역

### 2-1. 학생지도 체제의 적절성

| 평 가 기 준 |
|---|
| 대학은 학생들의 학습 및 생활지도를 위한 제도와 체제를 갖추고 이를 활발히 운영해야 하며, 학생회나 자율적 동아리 활동에 대해서도 관심을 갖고 적절히 지도해야 한다. |

**1. 대학은 학생들의 학습 및 생활지도를 위한 지도교수체제를 갖고 있으며(필수), 그 체제와 운영이 전문적으로 이루어지고 있는가?**

[지침] 의학과 학생을 위한 학생지도교수제도의 내용과 대학 내 전문 상담체계(전문상담 연구소 등)가 갖추어진 경우 그 내용과 최근 2년간 실적을 기술한다. [필수조건] 학습 및 생활지도를 위한 지도교수체제 확립

**2. 학생들의 건전한 자발적 학내외 활동(학술발표 및 의료봉사 등 사회봉사 활동)이 적절하며, 이에 대한 대학의 지원 및 지도 체계가 적절한가?**

[지침] 최근 2년간 학생들의 자발적 연구, 학술활동 및 사회봉사 활동내용과 이들 활동에 참여하는 학생들의 규모를 기술하고(단, 대학이 계획하고 시행하는 행사는 제외), 이들 활동에 대한 대학당국의 지원내용을 기술한다. 단, 학생들의 취미활동을 위한 동아리 활동은 여기서 제외한다.

**▢ 학생지도 체제의 적절성 평가자료 ▢**

1. 학생지도와 상담 관련 내용 및 실적
2. 학생 학술활동 및 사회봉사활동 지원 실적 (2년간)

## 2-2. 학생복지 제도 및 시설의 적절성

| 평 가 기 준 |
|---|
| 대학은 장학제도를 포함한 학생복지 제도를 적절히 마련해야 하며 기숙사나 기타 복지시설을 갖추어 학생들의 면학분위기 조성에 노력해야 한다. |

**1. 학생 장학제도가 있으며(필수), 학생의 장학금 수혜액과 수혜학생 비율은 적절한가?**

[지침] 대학의 장학제도를 기술하고 최근 2년간 장학제도, 장학금 종류별 지급 인원 및 지급금액에 대한 내용을 기술하며, 이 금액이 등록금 전액에 해당하는 학생수로 볼 때 몇 %가 되는지를 기술한다(등록금 액수 미만의 수혜자는 환산 등가수로 계산함. 예: 50% 수혜자가 2명이면 1명으로 계산). [필수조건] 학내외 장학제도 및 내용

**2. 재학생들의 기숙사 입실비율은 적절한가?**

[지침] 기숙사 시설 현황과 의학과 4개 학년 학생의 몇 %가 기숙사에서 생활하고 있는지를 기술한다. 현재 기숙사가 없는 경우는 앞으로의 계획을 기술한다.

**3. 학생복지 시설(식당, 매점, 운동시설 등) 및 제도(건강관리 등)가 고루 갖추어져 있고 그 수준도 적절한가?**

[지침] 의학과 학생 복지시설(식당, 매점, 운동시설 등)이나 복지제도(건강관리 등)에 대한 실태를 기술한다.

**▢ 학생복지 제도 및 시설의 적절성 평가자료 ▢**

1. 최근 2년간의 장학금 수혜현황
2. 기숙사 시설 및 관리현황
3. 학생복지 및 편의시설 실태

## 2-3. 졸업 후 진로 및 학습성과(의사국시 합격)의 적절성

| 평 가 기 준 |
|---|
| 의과대학 졸업생 중에는 임상의학 이외의 분야 특히 기초의학 연구 및 교육분야로 진출하는 사람도 있어야 하고, 의사국가시험에도 되도록 전원 합격할 수 있어야 한다. |

**1. 대학 졸업생 가운데 임상의학 이외 분야로 진출한 사람의 수가 적절한가?**

[지침] 졸업생중 지난 5년간 이 대학을 졸업한 학생 수와 이중 임상의학 이외 분야로 진출한 사람이 있는지 그 내용을 기술한다.

**2. 최근 2년간 의사국가시험 합격률은 적절한가?**

[지침] 최근 2년간 의사국가시험 합격률을 재학생과 졸업생으로 나누어 기술하고, 전체 합격률을 전국 평균합격률과 비교하여 기술한다.

**▢ 졸업 후 진로 및 학습성과의 적절성 평가자료 ▢**

1. 지난 5년간 임상의학 이외 분야로 진출한 졸업생 명단
2. 국가시험 합격 현황

## 3. 교수 영역

### 3-1. 기초 및 임상교원 확보의 적절성

| 평 가 기 준 |
|---|
| 의과대학은 기초의학과 임상의학 교육을 충실히 할 수 있도록 능력 있는 전임교원을 적정 수 반드시 확보하고 있어야 한다. 단, 전임교원은 교육부 등록 교원에 한한다. |

**1. 적정수의 기초의학 교원을 확보하고 있는가?**

[지침] 기초의학 교원의 수를 전공 분야별로 기술하고, 이를 권장기준과 비교하여 현재의 상태를 기술한다. [권장기준] 대학별, 교실별 적정 기초의학 인력을 규정하기는 쉽지 않다. 다만, 최근 각 의과대학에서의 기초의학 교육 비중이 임상의학 교육에 비해 상대적으로 감소하고 있어 일단 학생교육을 위한 기초의학 교원 인력은 꼭 필요한 최소한의 인력으로 정하는 것이 바람직하다. 이런 원칙을 바탕으로 본 평가에서는 8개 기본과목에 각각 최소 3명(기생충학의 경우 1명), 전체로는 30명을 최소 기초의학 교육 인력으로 확보하는 것을 기준으로 한다. 각 분야별 교원들 가운데 교육경력 10년 이상인 교원 1인이 포함되어야 하며, 교육 경력 중 군 경력은 제외한다. 기생충학을 제외한 각 교실은 의사교원을 최소한 1명 이상 확보하여야 하며, 겸임교수는 정원에서 제외하나 대학에서 정규 급여를 지급하는 기금교수는 정원에 포함한다. 겸임교수라 함은 타 단과대학 및 타 대학교에 적을 두고 있으면서 해당 대학에 교수직을 겸임하고 있는 경우를 말한다. 기금교수는 교육부 정원에 해당되지는 않지만 교육, 연구 및 진료에 있어 전임교수와 같은 활동을 하는 것이 확인된 경우 이를 포함한다.  단, 임상의학 교실에 속하지 않은 전임연구교수는 성격상 기초의학 교원으로 인정할 수 있다. 이 경우 이들은 별도로 명기해야 한다. 한편, 기초 해부병리와 임상병리과가 통합된 대학의 경우 기초의학 병리학과 교수로는 6명까지만 인정한다.

**2. 기초의학 교육과 연구를 보조하는 조교의 수는 적절한가?**

[지침] 기초의학 분야의 교육과 연구를 수행하기 위한 조교의 명단을 교실 또는 관련 분

야별로 기술하고, 이들의 교육 및 연구지원 업무 비율을 각각 %로 기술한다.

**3. 임상의학 담당 교원이 적정 수 확보되어 있는가?(필수)**

[지침] 임상의학 교원의 수를 전공 분야별로 기술하고, 이를 필수 조건과 비교하여 현재의 상태를 기술한다. [필수조건] 20개 이상 진료 및 지원과목에 교수가 총 85명 이상 있어야 하며 각 과에는 10년 이상 교육경력 교원이 최소 1명 이상 있어야 함

**▢ 기초 및 임상교원 확보의 적절성 평가자료 ▢**

1. 기초 및 임상교수 현황
2. 인사기록부(비치)
3. 기초의학 과목별 조교현황

## 3-2. 교수연구 및 학술활동의 적절성

| 평 가 기 준 |
|---|
| 교수들은 우수한 연구업적을 많이 발표해야 하며, 보다 많은 학외 연구비 수혜를 위해 노력해야 한다. |

**1. 최근 2년간 교수들의 국내 연구실적이 적절한가? (필수)**

[지침] 최근 2년간 전체 교수들의 국내발표 연구실적(원저, 종설, 증례보고, 저서, 역서)을 교수 1인당 평균 건수로 기술한다. 단, 1인당 평균건수는 매 연구실적 1편을 저자 수(N)로 나누어 계산한다. 단, SCI(SCIE 포함) 및 Index Medicus 등재 논문은 3-2-2에서 기술한다. 교수 연구실적은 실적종류나 발표지의 수준, 저자 수 등에 따른 질적평가가 중요하나 여기서는 편의상 모든 실적을 함께 취급하고 계산의 편의를 위해 1/N로 점수화한다. [필수조건] 교수 1인당 국내 연구실적 연평균이 1.0이상이어야 함

**2. 최근 2년간 대학 전체(교수 100명당 등재 논문수)로 SCI 및 Index Medicus 등재 논문수가 적절한가?**

[지침] 최근 2년간 SCI(SCIE 포함) 및 Index Medicus 등재 논문 수를 교실별 교수 수와

함께 기술하고, 이를 교수 100명당 연평균 논문 수로 계산한다.

**3. 최근 2년간 학외로부터 받은 연구비 수준은 적절한가?**

[지침] 최근 2년간 학외로부터 받은 연구비에 대해서 교실별 연구자, 연구제목, 연구기관 및 연구비 총액표를 만들고 교수 1인당 연평균 수혜 연구비를 계산한다. 단, 대학연구관리부서에 연구관리비를 지불한 연구에 한한다.

**▢ 교수연구 및 학술활동의 적절성 평가자료 ▢**

1. 교수 연구실적 목록집(비치)
2. 교수 연구비 수혜현황 목록

## 3-3. 교수개발 실태의 적절성

| 평 가 기 준 |
| --- |
| 대학은 교수들로 하여금 교육자로서, 전공분야 교육연수 및 진료 종사자로서 계속 발전할 수 있도록 각종 연수의 기회를 제공하고 이를 지원하고 있으며, 교수업적을 평가하고 적절하게 활용해야 한다. |

**1. 대학은 학내 의학교육 관련 교수연수 기회를 적절히 마련하고 있으며 교수들의 참여 정도가 적절한가?**

[지침] 최근 2년간 대학이 자체적으로 주관했던 순수 의학교육 관련 연수와 일반 교수연수 중 의학교육 관련 프로그램이 포함되었던 것을 구분해서 개최장소, 일시 참석인원, 주요 내용 등을 기술한다.

**2. 대학은 교수들의 해외연수 및 국내외 학회 참석을 위한 재정적 지원체제(등록비, 항공료, 체제비 등)를 적절히 갖고 있는가?**

[지침] 교수들의 해외연수 및 학회 참석을 장려하기 위한 재정적 지원체계가 갖추어져 있는지를 최근 2년간 지원 실적 내용과 함께 기술한다. 안식년제도를 시행하는 경우 그 내용과 실적도 함께 기술한다.

**3. 교수업적평가(교육·연구·봉사)가 이루어지고 있으며(필수), 그 결과가 적절하게 활용되고 있는가?**

[지침] 지난해(2003.3-2004.2)에 실시한 교수업적평가 서식을 제시하고 평가내용, 기준 및 그 결과 활용 등에 대해 기술한다. [필수조건] 교수업적 평가 실시

**▢ 교수개발 실태의 적절성 평가자료 ▢**

1. 최근 2년간 대학주최 의학교육관련 연수회 시행 실적
2. 해외연수 및 학회참석 지원실적
3. 교수업적 평가자료(비치)

## 4. 시설 · 설비 영역

### 4-1. 교육 기본 및 지원 시설의 적절성

| 평 가 기 준 |
|---|
| 대학은 학생들의 교육을 위한 쾌적하고 충분한 교육기본 시설을 갖추어야 하며, 교육효과를 높이기 위한 도서실, 전산실, 시청각 교재실 등 지원 시설을 충분히 갖추고 있어야 한다. |

**1. 강의실과 실험실습실을 적정 수 확보하고 있으며(필수), 그 설비 및 관리 실태가 적절한가?**

[지침] 강의실과 실험실습실의 수를 필수조건과 비교하여 현 상태를 기술하고 설비 및 관리실태(인력관리, 예산 등)를 기술한다. [필수조건] 의학과 전용 강의실 4개(의학과 3, 4학년용 강의실이 병원 내에 있는 경우도 포함)와 기초의학 실험실습실 3개 확보

**2. 임상실습을 위한 기본 교육 병원을 적절히 확보하고 있으며(필수), 병원 내에 학생교육 시설을 갖추고 있는가?**

[지침] 임상실습 병원의 규모 및 활용상황을 필수조건과 비교하여 기술하고, 병원 내 학생교육을 위한 시설에 대해 기술한다. [필수조건] 500병상 이상의 독립 교육병원 확보

**3. 의과대학생을 위한 도서관이 있으며(필수), 의학 관련 도서확보 및 운영**

**실태는 적절한가?**

[지침] 의과대학생을 위한 도서관이 있는지 여부를 기술하고(중앙도서관에 포함되어 있을 경우, 의과대학생을 위한 전용 도서관의 구분이 어떻게 되어 있는지를 기술 함), 의학도서관(실)의 단행본, 학술지 및 비도서자료(CD ROM, on-line DB 포함) 확보실태를 종수별로 기술하고, 최근 2년간 도서관 연간 예산을 기술한다. 또한 도서관 이용을 위한 정보제공 서비스 실태를 기술한다. [필수조건] 의과대학생을 위한 도서관 확보

**4. 의과대학생을 위한 교육지원시설 즉, 전산실, 시청각 교재실, 자율학습실, 소그룹 토의실(PBL 실 포함) 등이 적절히 갖추어져 있는가?**

[지침] 의과대학생을 위한 교육지원시설의 수, 규모, 사용실적 및 관리실태를 기술한다.

**▢ 교육 기본 및 지원 시설의 적절성 평가자료 ▢**

1. 시설현황 (대학 및 병원)
2. 병원연보(비치)
3. 도서 확보 실태
4. 정보제공 서비스 자료(Newsletter, 목록집, 설명회 등)(비치)

## 4-2. 교수 시설의 적절성

| 평 가 기 준 |
| --- |
| 대학은 개인 교수실과 교수를 위한 연구 시설을 충분히 확보해야 한다. |

**1. 전임강사 이상 교원의 개인 교수실이 있고, 그 실내 설비는 잘 되어 있는가?**

[지침] 개인 교수실의 확보실태(전체 교원 수에 대한 개인 교수실 수의 비율)와 교수실의 실내설비(조명, 냉난방, 방음, 환기, 채광, Lan 설치 등) 상태를 기술한다.

**2. 기초 및 임상교수들의 연구시설 공간과 설비가 적절한가?**

[지침] 기초 및 임상각과의 독립 또는 공동 교수 실험연구실 확보현황과 연구기자재 확보 및 관리실태를 기술한다.

**▢ 교수 시설의 적절성 평가자료 ▢**

1. 개인 교수실 확보현황
2. 대학 내 연구시설 및 설비 현황(대학공동 및 교실별 시설)

## 5. 행정 · 재정 영역

### 5-1. 대학행정 및 운영 체계의 적절성

| 평 가 기 준 |
|---|
| 대학은 학사운영을 위한 적정 행정인력과 합리적인 조직 체계를 갖추어야 하며, 효율적인 대학운영을 위한 책임행정 체제를 갖추는 것이 바람직하다. |

**1. 대학 행정업무는 교무, 학생, 연구 및 서무 등 업무별로 구분되어 있으며 인력의 확보는 적절한가?**

[지침] 대학 행정지원업무가 교무, 학생, 연구 및 서무별로 구분되어 있는지와 이들 업무를 담당하는 인력 현황을 기술한다. 대학원에 재학중인 학생을 행정조교로 쓰는 경우 그 내용을 별도로 기술한다.

**2. 대학 행정업무 계획 및 예산 집행에 관한 학장의 자율권과 책임경영 체제가 대학 예산과 학사계획 범위 내에서 확보되어 있는가?**

[지침] 학교 행정에서 학장이 인사(교수, 조교, 직원 임용이나 관리)에 참여할 수 있는 범위와 학장전결 예산집행가능 범위를 기술한다. 또한 학장이 계획하고 결재한 사항의 집행정도를 기술한다.

**3. 대학 운영과 관련한 의견수렴 기구(위원회 포함)가 구성되어 있고 적절하게 운영되고 있는가?**

[지침] 대학 운영을 위한 주요 의견 수렴기구(위원회)와 의사결정 기구에 대하여 그 구성원, 업무분담, 운영계획 등을 기술한다. 이들 기구에서 결정된 내용이 실제 얼마나 반영되고 집행되는지를 기술한다.

**▢ 대학행정 및 운영 체계의 적절성 평가자료 ▢**

1. 대학 행정기구표 및 인력현황
2. 의사결정 기구 및 위원회 활동 자료

## 5-2. 대학재정의 적절성 및 확보노력

| 평 가 기 준 |
|---|
| 대학은 원활한 대학 운영을 위한 적정 예산을 확보해야 하며 건실한 대학 재정을 위한 노력을 해야 한다. |

**1. 대학운영을 위한 예산과 최근 2년간 예산변동 상황은 적절한가?**

[지침] 2004년도 주요 예산 내역을 기술하고 최근 2년간 예산변동 및 그 내용이 대학 교육 발전에 적절한 방향인지 여부를 기술한다.

**2. 학생 1인당 기초의학 실험실습비가 적정한가?**

[지침] 최근 2년간 학생 실험・실습용 기초의학 실험실습비 예산 내역을 기술하고 학생 1인당 배정된 기초의학 실험 실습비 (예산서상 학생경비 중 기초의학 학생실험실습비 총액 ÷ 전체 학생수)를 계산 제시한다. 의학과 과목 실험실습이 의예과에서 시행되는 경우라도 계산에서는 의예과를 제외한 의학과 학생을 전체 학생으로 간주한다.

**▢ 대학재정의 적절성 및 확보노력 평가자료 ▢**

1. 대학예산서(비치)

## 5-3. 대학발전 계획 정도의 적절성

| 평 가 기 준 |
| --- |
| 대학은 대학의 경쟁력 제고를 위한 실현 가능한 중ㆍ장기 발전계획을 가지고 있어야 하며 대학 발전에 대한 동문 등의 적극적인 참여를 유도해야 한다. |

**1. 대학의 발전 계획이 수립되어 있으며(필수), 그 실현을 위한 대학본부와 재단의 의지와 노력은 적절한가?**

[지침] 대학발전 계획서 내용을 소개하고 이 발전계획 추진을 위한 의과대학, 대학본부 및 재단의 의과대학 발전 지원 노력과 예산조달 내용 등을 기술한다. [필수조건] 기술된 의과대학 발전계획서

**2. 의과대학 발전을 위한 발전기금 규모와 모금계획은 적절한가?**

[지침] 2004년 3월 1일 현재 발전기금규모가 어느 정도인지 기술하고, 모금활동 내용 및 앞으로의 모금계획, 그리고 모금된 기금이 현재에도 대학발전에 쓰이고 있으면 어디에 어떻게 사용되고 있으며 앞으로의 집행계획은 어떤지 등을 기술한다.

**3. 대학발전을 위한 동문 및 지역사회 등 참여가 구체적이며 그 참여정도는 적절한가?**

[지침] 의과대학 발전을 위한 의과대학 동문회 등의 구체적인 지원활동, 사업계획과 실적을 기술한다.

**▢ 대학발전 계획 정도의 적절성 평가자료 ▢**

1. 대학발전 계획서(비치)
2. 대학발전에 대한 동문회 계획 및 참여실적 현황

# 제2주기 의과대학 인정평가기준

## 일 러 두 기

1. 제2주기 의과대학 인정평가 기준은 의과대학 또는 의학전문대학원 체제를 갖고 있는 대학 모두에게 적용되는 기준이다. 따라서 의과대학과 의학전문대학원의 의미를 구분하여 표기하지 않는 경우 의과대학이라 함은 의학전문대학원을 포함한다.

2. 인정평가 기준(안)의 세부 내용 중

   (1) 특별히 자료 제시 기간을 명시하지 않을 경우에는 최근 2년간의 자료를 기본 자료로 한다.(예: 2007년 평가대학은 2005년 3월 1일부터 2007년 2월 28일까지의 자료를 기본으로 한다)

   (2) 의예과와 의학과를 통합하여 단일 6년제 과정을 운영하는 대학의 경우에는 의예과를 포함하여 기술할 수 있으나, 의예과와 의학과를 구분하여 자료를 작성해야 한다.

   (3) 핵심과목이란 내과학, 외과학, 산부인과학, 소아과학, 정신과학 및 응급의학을 의미한다.

   (4) 필수기준은 모든 의과대학들이 반드시 도달해야 하는 기준이며, 권장기준은 모든 의과대학들이 도달하기를 제안하는 기준이다. 또한 우수기준은 의학교육의 수월성을 추구하기 위해 제시되는 기준이다.

   (5) 참고자료의 [부록]은 대학자체평가 보고서에 첨부하는 자료이며, [비치]는 현지방문평가시 평가위원이 확인하는 자료를 의미한다. 아울러 [현지확인] 자료는 비치할 수는 없으나 현지방문 평가시 평가위원이 확인하는 자료이다.

3. 의과대학 인정평가를 위한 평가기준은 제2주기 평가주기 동안에도 일정한 절차를 거쳐 수정, 보완 될 수 있다.

# 평가영역별 평가문항 수

| 평가영역 | 평가부문 | 문항 수 | 평가기준 수 | | |
|---|---|---|---|---|---|
| | | | 필수 | 권장 | 우수 |
| 1. 대학 운영 체계 | 1-1 대학의 설립 | 3 | 3 | 0 | 0 |
| | 1-2 대학행정 및 운영 체계 | 4 | 4 | 0 | 1 |
| | 1-3 대학재정 | 3 | 1 | 2 | 0 |
| | 1-4 대학발전계획 | 3 | 1 | 2 | 0 |
| | 1-5 개선노력 | 3 | 2 | 1 | 1 |
| | 소계 | 16 | 11 | 5 | 2 |
| 2. 교육목표와 교육과정 | 2-1 교육목표와 교육과정의 기본 틀 | 5 | 2 | 3 | 2 |
| | 2-2 기초의학 교육과정 | 3 | 3 | 0 | 0 |
| | 2-3 임상의학 교육과정 | 8 | 6 | 2 | 2 |
| | 2-4 인문 · 사회의학 교육과정 | 3 | 1 | 2 | 1 |
| | 2-5 수업방법과 강좌평가 | 3 | 3 | 0 | 2 |
| | 2-6 학생 학습평가 | 3 | 2 | 1 | 2 |
| | 소계 | 25 | 17 | 8 | 9 |
| 3. 학생 | 3-1 입학정책 및 학생선발 | 2 | 0 | 2 | 0 |
| | 3-2 학생지도체계 | 3 | 1 | 2 | 2 |
| | 3-3 학생복지 제도 및 시설 | 5 | 2 | 3 | 3 |
| | 3-4 졸업 후 진로 및 학습 성과 | 2 | 0 | 2 | 1 |
| | 소계 | 12 | 3 | 9 | 6 |
| 4. 교수 | 4-1 기초 및 임상 전임교수 | 5 | 2 | 3 | 4 |
| | 4-2 교수의 연구 및 학술활동 | 5 | 1 | 4 | 4 |
| | 4-3 교수개발 실태 | 3 | 3 | 0 | 3 |
| | 소계 | 13 | 6 | 7 | 11 |
| 5. 시설·설비 | 5-1 교육 시설·설비 | 4 | 3 | 1 | 4 |
| | 5-2 연구 관련 시설·설비 | 2 | 1 | 1 | 1 |
| | 소계 | 6 | 4 | 2 | 5 |
| 6. 졸업 후 교육 | 6-1 대학원 교육 | 3 | 0 | 3 | 1 |
| | 소계 | 3 | 0 | 3 | 1 |
| 합계 | | 75 | 41 | 34 | 34 |

# 1. 대학 운영 체계 영역

## 1-1 대학의 설립

**평 가 개 요**

대학은 건전하고 명시된 건학이념에 의하여 설립되고, 운영의 독립 및 자율이 보장되어야 하며, 고유의 설립 목적 이외의 어떠한 이득이나 이해, 갈등관계에 놓여서는 안 된다. 또한, 대학의 설립주체는 충분한 재원과 능력을 바탕으로 건학이념의 구현을 위한 사회적 책임과 사회 공익적 기여에 충실하여야 하며 대학의 발전을 위하여 최대의 노력을 경주하여야 한다.

### 1-1-1 대학은 기술된 건학이념을 갖고 있으며, 대학의 사회적 책임과 공공성 확보를 위한 내용을 포함하고 있는가?

**[보고서 기술내용]** 대학의 설립 배경과 과정, 건학이념 및 그 내용을 사회적 책임과 공공성 확보 측면에서 기술한다.

**[필수기준]** 기술된 대학의 건학이념이 있어야 한다.

**[참고자료]**

비치 : 1. 대학의 건학이념 관련 자료

### 1-1-2 의과대학의 자율성과 독립성이 보장되어 있는가?

**[보고서 기술내용]** 의과대학이 종합대학의 일부인 경우 대학본부와 의과대학의 재정과 학사에 관한 역학구도를 기술하고 의과대학의 자율성과 독립성에 대해 기술한다. 의료원체제를 갖춘 경우 의과대학 행정, 재정, 인사에 관한 운영의 독립성과 자율성을 기술한다. 설립주체가 재단인 경우 재단과  대학 간의 행정, 재정, 인사를 포함한 학사 운영에 관하여 기술한다.

[주] 자율성과 독립성은 분리된 개념이 아니다. 의과대학의 수행능력은 전문성에 바탕을 두어야 한다. 의과대학이 외부(대학본부, 재단)의 적절치 못한 영향력에 의하여 의과대학 업무수행의 지장을 초래하거나  업무상 수월성의 문제가 제기 되지 않도록 하여야 한다. 이 항목은 정량적인 지표보다는 의과대학의 업무(교육, 시설설비, 인사, 행재정 등)의 수행에 대한 스스로의 총괄적인 견해를 기술하는 것이다. 중요한 개념은 의과대학 운영의 전문성에 입각한 대학 운영이다. 예를 들어 의과대학 교수의 임용은 의과대학의 요구분석과 임용방식에 의한 것인가 아니면 행정편의에 바탕을 둔 획일화 된 정책에 의한 것인가 등의 문제이다.

**[필수기준]** 의과대학과 대학본부, 의료원, 재단 상호간에 의과대학 운영에 관한 독립성과 자율성이 존중되어 있고 이를 위한 제도적 장치가 마련되어 있어야 한다.

**[참고자료]**

부록: 1. 재단, 대학본부, 의료원과 의과대학의 역학구도를  보여주는 행정구조표

비치: 1. 의과대학의 분권화를 위한 학칙, 위원회자료, 실적보고서 등

현지확인: 1. 학장, 의무부총장, 총장, 재단이사장 면담

### 1-1-3 대학은 세계보건기구가 권장하는 사회적 책무성을 위한 노력을 하고 있는가?

**[보고서 기술내용]** 세계보건기구(WHO)가 권장하는 사회적 책무성을 수행하기 위해 교육, 연구, 진료 부문에서 대학이 노력하고 있는 내용을 기술한다. 또한 각 지역사회의 보건의료 관련 단체와의 연관성을 기술한다.

**[필수기준]** 사회적 책무의 수행과 관련한 의과대학의 교육, 연구, 진료 방침이 있고, 이러한 방침이 시행되고 있어야 한다.

[주] 사회적 책무성은 대학의 교육, 연구, 진료 활동이 소속된 지역사회나 국가의 보건 우선과제 지향적 이어야 하는 의무이다. 보건우선과제는 정부, 의료단체, 의료인과 공공의 공동에 의하여 선정 되어야 한다(WHO “social accountability"). WHO의 Social Accountability는 다음 자료를 참고한다.

Boelen, C., and Heck, J.E. 1995. Defining and measuring social accountability of medical schools. Unpublished document. WHO/HRH/95.7. Geneva: World Health Organization. 3.

**[참고자료]**

비치: 1. 사회적 책무성에 대한 대학의 방침 및 책무성 수행 실적보고서

현지확인: 1. 사회적 책무성에대한 인지도 및 활동 확인

## 1-2 대학 행정 및 운영 체계

**평 가 개 요**

대학은 행정업무를 교육, 교수, 학생, 연구, 졸업 후 교육, 정보, 입학, 재정 등으로 구분하여 이를 담당하는 책임자와 인력이 있어야 하며, 행정 업무 계획 및 예산 집행에 대한 자율권이 대학행정 책임자에게 있어야 한다. 또한, 대학 병원이 지역적으로 떨어져 있는 경우 교육과 연구의 표준화를 위한 구조가 있어야 한다.

### 1-2-1 대학 행정은 업무별로 구분되어 있고 인력 확보는 적절한가?

**[보고서 기술내용]** 대학 행정 업무가 교육, 교수, 학생, 연구, 졸업후 교육, 정보, 입학, 재정 등으로 구분되어 있는지와 담당 인력 현황을 기술한다. 교육, 교수 및 학생 분야를 반드시 포함하여 각 업무별로 보직자가 지정되어 있고 해당 업무가 보직자를 중심으로 기획되어 실행되고 있는 가를 기술한다. 대학원 재학생을 행정조교로 쓰는 경우 그 내용을 별도로 기술한다. 의과대학 행정 조직 내에 의예과, 간호학과, 의공학과, 대학원 등 타 학과가 함께 있는 경우 각각의 업무 분담과 담당 인력에 대해서 기술한다. 행정 조교, 대학원생 조교는 행정직원 수에는 포함하지 않는다.

[주] 업무의 관련성에 따라서 한 명의 보직자가 두 개의 업무를 맡을 수 있다.

**[필수기준]** 행정업무가 교육, 학생, 교수, 연구, 졸업 후 교육, 정보, 입학, 재정으로 구분되어 있고 행정업무 담당 직원이 최소 5명이상 되어야 한다. 또한, 대학 운영을 위해 기본적으로 필요한 교육, 교수, 학생, 연구 등의 업무를 행정적 연관성과 관련하여 의과대학 보직자가 적절한 업무를 분담한다.

**[우수기준]** 교육, 학생, 교수, 연구 분야를 포함한 최소 7 개 분야의 보직자가 임명되어 있으며, 그 활동 실적이 있다.

**[참고 자료]**

비치: 1. 업무 회의록 및 활동 실적

2. 보직 수당 지급 내역

**1-2-2 대학의 행정업무 계획 및 예산 집행에 관한 학장의 자율권과 책임경영 체제가 확보되어 있는가?**

**[보고서 기술내용]** 학장이 교수, 조교, 직원 임용 등 인사 관련 회의 및 의사결정 과정에 참여할 수 있는 범위와 학장이 자율적으로 사용할 수 있는 연간 총 금액 및 일회 전결 가능 범위를 기술한다. 또한, 학장 주관 하에 계획되고 결재된 사항이 어느 정도 집행되고 있는지를 기술한다.

[주] 학장이 자율적으로 집행할 수 있는 금액은 대학의 예산항목으로 반영되어 있는 업무추진비, 예비비 등을 의미하는 것은 아니다. 이 금액은 대학의 사명과 목표 달성을 위해 학장이 자율적으로 집행할 수 있는 정책적 재원을 의미한다.

**[필수기준]** 대학 본부(또는 의료원)로부터 위임 또는 승인 받은 대학의 행정 업무와 예산이 실제 학장의 책임 하에 자율적으로 집행되어야 한다. 학장이 주관하는 위원회에서 교수 채용 및 승진 자격 요건과 해외 연수 자격 요건 등을 논의하고 있다는 명문화된 규정이 있어야 한다. 실험 실습비와 경상비의 집행이 학장 전결로 이루어지며 의과대학 학사 일정이 학장 책임 하에 계획 관리되어야 한다.

**[참고 자료]**

비치: 1. 학장의 임무와 권한에 관한 규정

2. 전결 업무 규정

3. 의과대학 교수의 인사 규정

### 1-2-3 대학 운영을 위한 정책결정 구조와 절차가 적절하게 운영되고 있는가?

**[보고서 기술내용]** 대학 운영을 위한 정책결정 구조와 절차를 제시하고, 전체 교수 또는 주임교수 회의, 각종 위원회와 같은 기구의 역할에 대하여 기술한다. 또한, 각 의견 수렴 기구별 구성원, 역할분담, 활동실적 등을 기술하고 이들 기구에서 결정된 내용이 대학 운영에 얼마나 반영되고 집행되는지를 기술한다.

**[필수기준]** 대학 운영을 위한 정책결정 구조와 절차가 분명하게 확립되어 있어야 하며, 의견수렴기구로 교육, 학생지도 및 인사 관련 위원회는 반드시 구성되어 있고, 그 구성과 활동은 적절해야 한다.

[주] 인사 관련 위원회의 선출직 위원은 1/3이상 이어야 하며, 기타 위원회는 당연직 위원이 1/4이내 이어야 한다. 또한, 각 위원회는 직급별, 분야별 (기초, 임상 등)로 다양하게 구성되어 있어야 한다. 각종 위원회는 한 학기에 최소 1회 이상 개최되어야 한다. 당연직 위원에는 주임교수 및 병원보직자를 포함한다.

**[참고 자료]**

비치: 1. 각 위원회, 주임교수회의 및 전체 교수회의 업무 회의록 및 활동 실적

**1-2-4 대학 부속 병원이 지리적으로 떨어져 있는 경우 각 병원마다 교육과 연구를 지원하는 대학 행정 구조가 있는가?**

**[보고서 기술 내용]** 대학의 (여러) 부속병원들이 지리적으로 떨어져 있는 경우 교육과 연구를 총괄하는 보직자 또는 부서에 대해서 기술하고, 각 병원별로 교육과 연구를 담당하는 체계, 업무추진 상황 및 실적 등에 대해서 기술한다. 또한, 각 병원별 임상실습 교육을 표준화하기 위해 어떤 노력을 하고 있는지 기술한다.

**[필수기준]** 교육과 연구를 총괄하는 보직자가 임명되어 있고, 지리적으로 떨어져 있는 각 부속병원마다 교육과 연구를 지원하는 행정구조가 임명되어 있어야 한다. 또한 부속병원 상호간에는 학생 임상실습 교육을 표준화하기 위한 정기적인 회의가 개최되고, 회의 결과에 따른 집행실적이 있어야 한다.

**[참고 자료]**

부록: 1. 부속병원별 교육연구지원 행정구조

비치: 1. 교육과 연구 담당 총괄부서의 활동 자료

2. 병원별 담당 교수간의 회의 자료

## 1-3 대학 재정

**평 가 개 요**

대학은 교육관련 운영에 필요한 재원을 확보하기 위해 장단기 계획을 수립해야 한다. 또한 확보된 재원을 공정하고 합리적으로 배분하고 효율적으로 활용하며, 내·외부 감사제도를 통해 그 효과가 극대화 될 수 있도록 노력해야 한다.

### 1-3-1 대학 운영을 위한 예산편성제도는 적절한가?

**[보고서 기술내용]** 대학의 예산 편성 시기, 절차, 대학 사업계획과 예산의 연계성, 예산심의 과정 등에 대해서 기술한다.

**[권장기준]** 대학은 합리적인 예산편성을 위해 구성원의 의견을 수렴하여 반영하고 있으며, 대학의 사업계획과 예산은 적절하게 연계되어 있으며, 예산심의 과정이 합리적으로 이루어지고 있다. 또한, 의료원 체제를 가지고 있는 경우에는 의과대학의 독립된 예산이 책정되어 있다.

[주] 바람직한 예산 편성을 위해서는 각 교실(과)별 사업계획과 예산에 대한 의견 수렴 결과를 바탕으로 대학의 사업계획과 연관되어 검토되어야 한다.

**[참고자료]**

비치: 1. 대학의 예산 편성을 위한 의견 수렴 자료

2. 대학의 예산 편성을 위한 회의 자료

### 1-3-2 대학은 적절한 교육관련 재정을 확보하고 있는가?

**[보고서 기술내용]** 최근 2년간 교육관련 재정확보 현황을 항목별로 기술하고, 대학의 노력과 성과에 대해서 기술한다.

**[필수기준]** 최근 2년간 교육관련 재정(학생 실험·실습비, 교육과정 개발, 운영 및 평가비, 교수개발비, 국내·외 교수 연수 지원비, 각종 교육 세미나 개최비, 학생 봉사활동 지원비 등)이 등록금 인상에 비례하여 증가하여야 한다.

**[참고자료]**

비치: 1. 대학 예산 집행 내역 서류

### 1-3-3 대학의 내·외부 감사제도와 활용은 적절한가?

**[보고서 기술내용]** 대학의 합리적인 운영을 위해 내·외부 감사제도를 갖고 있는지, 감사제도의 절차와 시기, 감사에서 지적된 문제점, 감사결과의 활용과 개선 노력 등은 무엇인지 구체적으로 기술한다.

**[권장기준]** 대학의 내·외부 감사제도가 확립되어 있으며, 그 절차가 합리적으로 진행되고 있으며, 감사에서 지적된 문제점을 개선하기 위한 가시적인 노력이 있다.

**[참고자료]**

비치: 1. 최근 2년간 감사결과 및 결과 활용 실적 자료

## 1-4 대학 발전 계획

**평 가 개 요**

대학은 경쟁력 제고를 위한 실현 가능한 중장기 발전계획을 가지고 있어야 하며, 동문 등이 대학 발전에 적극적으로 참여할 수 있도록 해야 한다.

### 1-4-1 대학의 발전 계획이 수립되어 있으며, 대학과 재단의 의지와 노력은 적절한가?

**[보고서 기술내용]** 의과대학 발전계획서 내용을 소개하고, 발전계획의 추진을 위한 의과대학, 대학본부 및 재단 등의 지원 노력과 예산조달 내용 등을 기술한다.

**[필수기준]** 총장 또는 재단 이사장의 서명이 있는 기술된 의과대학 발전계획서가 있어야 하며, 발전추진위원회가 구성되어 연 2회 이상의 회의 개최 실적과 추진 예산(회의비, 홍보물 제작비 등)이 확보되어 있어야 한다. 또한 다양한 대학 구성원들이 의사결정과정에 참여한 실적과 발전계획을 구성원에게 알리기 위한 노력 등이 있어야 한다.

**[참고자료]**

비치: 1. 대학 발전 계획서

2. 대학 발전 추진위원회 회의록

3. 추진예산집행내역

### 1-4-2 대학 발전기금 규모와 모금계획은 적절한가?

**[보고서 기술내용]** 대학 발전기금의 규모, 모금활동 내용, 앞으로의 모금계획을 기술하고, 모금된 기금이 대학발전에 어떻게 사용되고 있으며, 또한 앞으로의 집행계획 등에 대해서 기술한다.

**[권장기준]** 의과대학 독자적으로 모금된 발전 기금이 있고, 발전기금을 관리하기 위한 위원회의 구성과 운영이 적절하며, 발전기금의 구체적 집행 내역 및 향후 집행 계획이 잘 수립되어 있다.

[주] 종합대학내 의과대학 발전기금으로 지정된 기금(지정기탁)이 있는 경우에는 발전기금을 관리, 운영하기 위한 위원회가 의과대학에 구성되어 있어야 하며, 동 위원회에서 발전기금의 집행 계획이 수립되고 관리되어야 한다.

**[참고자료]**

비치: 1. 발전기금 관리위원회 회의록

2. 발전기금 집행내역 관련자료 및 집행계획

3. 확보된 발전기금 확인 자료(통장 등)

**1-4-3 대학 발전을 위한 동문 및 지역사회의 참여와 이들의 적극적 참여를 위한 대학의 노력은 적절한가?**

**[보고서 기술내용]** 대학 발전을 위한 동문회 또는 지역사회의 구체적인 지원활동과 참여실적 등을 기술한다. 또한 동문 및 지역사회가 대학 발전에 적극적으로 참여할 수 있도록 대학이 어떤 노력을 하였는지에 대해서 기술한다.

**[권장기준]** 동문회 또는 지역사회 중심의 대학 발전 특별 기구가 구성되어 잘 운영되고 있으며, 동문회나 지역사회의 구체적인 참여 실적이 있다. 또한, 이들의 적극적인 참여를 위하여 대학이 적극적으로 노력하고 있다.

**[참고자료]**

비치: 1. 대학 발전 특별 기구 운영실적

2. 동문회 및 지역사회 활동 실적 보고서

## 1-5 개선노력

| 평 가 개 요 |
| --- |
| 대학은 교육의 질 관리를 위한 조직을 운영하여 제1주기 평가결과를 대학운영에 반영하고, 의학교육의 질 향상 및 국제화를 위해 지속적으로 노력해야 한다. |

### 1-5-1 대학은 질 관리 및 개선을 위한 기구가 있으며 적절하게 운영하고 있는가?

**[보고서 기술내용]** 대학의 질 관리 및 개선을 위한 상설기구 또는 위원회 성격의 연구기구가 있는지와 그 구성 및 운영 내용과 절차를 기술한다. 또한, 이러한 질 관리 기구의 주요 활동 실적을 기술한다.

[주] 대학은 빠르게 변하는 환경에 대한 적응과 지속성을 요구하는 역동적인 기관으로 질 관리 및 개선을 위한 대학 자체적인 기구를 갖고 있어야 하며 대학의 조직과 기능을 정기적으로 검토하고 개선해야 한다.

**[필수기준]** 대학의 지속적인 질 관리 및 개선을 위한 상설기구가 있어야 하며, 대학의 조직과 기능에 대해 정기적인 검토가 이루어지고 있어야 한다.

**[우수기준]** 대학의 질 관리 및 개선을 위한 상설기구는 대학의 경영목표의 달성 정도를 평가하고, 정기적으로 평가보고서를 발행하며 그 결과를 대학운영에 반영하고 있다.

**[참고자료]**

비치: 1. 정기적인 검토 내용을 포함하는 최근 3년의 관련 서류

2. 수정되고 실제 개선된 사항을 기록한 서류

3. 정기적으로 이루어진 자체 평가 자료

### 1-5-2 대학은 제1주기 평가결과를 대학운영에 반영하고 있는가?

**[보고서 기술내용]** 제1주기 의과대학 인정평가 결과보고서의 개선 요구 사항을 영역별로 기술하고, 각 요구 사항이 어떤 절차를 통해 대학 운영에 어떻게 반영되었는지, 개선에 따른 구체적인 효과는 무엇인지 기술한다.

**[필수기준]** 평가사업단의 제1주기 평가 결과를 구성원 모두가 공유하기 위한 대학의 구체적인 노력이 있어야 한다. 또한, 영역별 미비점과 개선점에 대한 단기, 중·장기 개선 계획이 수립되어 실천되고 있으며, 이러한 개선 계획에 따른 구체적인 개선 실적이 있어야 한다.

[주] 각 영역별 개선 요구사항에 대한 단기, 중·장기 개선 계획, 제1주기 인정평가 이후의 경과 기간, 대학의 지속적인 개선 노력 등을 종합적으로 고려하여 평가하되, 구체적인 개선 실적이 있어야 한다.

**[참고자료]**

비치: 1. 제1주기 의과대학 평가결과보고서

### 1-5-3 외국 의과대학과의 교류는 적절한가?

**[보고서 기술내용]** 대학은 연구와 교육의 국제화를 위해 외국 의과대학 및 관련 기관과의 교류가 이루어지고 있는지 그 현황과 집행 예산을 기술한다.

**[권장기준]** 국제협력을 위한 전담 보직이나 기구가 있고 예산과 사무공간이 있다. 해외 연구, 교육, 실습 등의 학생 교류실적은 입학정원 기준으로 5% 이상의 학생이 최소 2주 이상 참여하고 있다.

**[참고자료]**

비치: 1. 국제화 실적 예산집행 확인

[주] 실적보고서는 상호방문에 관한 서신교환, 교환학생 평가보고서 등 증빙서류

[주] 예산집행에는 학생이 자신의 부담이나 학교 측의 지원 증빙서류

# 2 교육목표와 교육과정 영역

## 2-1 교육목표와 교육과정의 기본 틀

**평 가 개 요**

대학은 의과대학 졸업생의 수행능력 범위, 교육이념과 특성이 잘 반영된 교육목표를 가지고 있어야 하며, 이 목표는 교육과정에 구체적으로 반영되어야 한다. 또한 대학은 자체 개발한 원칙과 원리에 부응하는 교육과정을 가지고 있어야 하며, 이 과정은 졸업생이 일하게 될 보건의료환경에 부합하여야 한다. 그리고 교육과정의 개선과 관리를 위한 기구가 있어야 하며, 이 기구에 권한과 책임, 예산이 배정되어 있어야 한다.

**2-1-1 대학은 졸업생들이 갖추어야 할 지식, 술기, 태도에 대하여 구체적으로 기술된 목표를 갖고 있으며, 그 내용 속에 대학의 교육이념과 특성화 의지 및 보건의료환경의 요구를 반영하고 있는가?**

**[보고서 기술내용]** 교육목표 내용을 기술하고 대학이 그러한 교육목표를 설정하게 된 과정, 배경 그리고 이유 등을 기술한다. 이와 관련하여 대학 설립자의 교육이념이나 특성화 의지 및 보건의료환경의 요구도 함께 기술한다.

**[필수기준]** 졸업생들이 갖추어야 할 지식, 술기, 태도에 대하여 구체적으로 기술된 목표가 있으며, 이것이 구성원의 합의나 관련 연구위원회의 조직적인 노력을 거쳐 합리적으로 만들어지고 그 내용 속에 대학의 교육이념, 특성화 교육목표 및 보건의료환경의 요구 등이 구체적으로 반영되어 있어야 한다.

**[참고자료]**

비치: 1. 의료환경 변화에 대한 모니터링 자료

**2-1-2 대학의 교육목표는 구성원들에게 잘 인지되어 있으며, 대학은 인지도를 높이기 위해 적절한 노력을 하고 있는가?**

**[보고서 기술내용]** 대학의 교육목표가 구성원들에게 잘 인지되어 있는지와 이를 위한 대학의 노력 내용을 구체적으로 기술한다.

[주] 교육목표를 구성원에게 인지하기 위한 대학의 노력은 공지, 세미나, 연수, 강의실 및 실습실 게시, 정기적 설문 결과 등에 대해서 기술한다.

**[권장기준]** 교육목표에 대한 대학 구성원의 인지도가 60%이상이고, 인지도를 높이기 위한 대학의 노력이 다양한 방법(교수세미나, 연수회, 설문조사 등)을 통해 이루어지고 있다.

**[참고자료]**

비치: 1. 교육목표 홍보를 위한 자료

2. 교육목표 인지도 향상을 위한 근거자료(세미나, 연수회, 설문지 등)

### 2-1-3 대학의 교육목표가 교육과정에 반영되고 있는가?

**[보고서 기술내용]** 교육목표로 기술된 내용들이 교육과정에 어떤 내용으로 어떻게 반영되어 있는지를 교육목표 항목별로 기술한다. 교육목표 하나하나에 대해서 이들이 어떤 교과목 또는 어떤 교육 프로그램을 통해 실천되고 있는지를 기술한다.

**[권장기준]** 대학이 설정한 교육목표를 달성하기 위하여 교육내용들이 교육과정에 구체적으로 반영되어 있다.

**[참고자료]**

비치: 1. 전학년 교육과정 시간표

2. 대학의 개설 교과목 및 교육 목표 관련 교육 내용 자료(교수요목, 수업계획서)

### 2-1-4 대학은 교육의 질적 향상을 추구하기 위한 교육과정을 운영하고 있는가?

**[보고서 기술내용]** 대학의 교육과정 유형과 그 구체적인 구성 및 기간을 기술한다.

[주] 교육과정의 유형은 다음과 같이 분류될 수 있으며, 대학에 따라서는 혼합 형태 또는 또 다른 형태의 교육과정을 운영할 수도 있다. 예) 1. 교과목 중심의 전통적인 교육과정, 2. 소집단 토론학습이 바탕이 되는 문제바탕학습 교육과정, 3. 기초의학간, 임상의학간, 기초-임상의학간 통합교육과정 등.

**[필수기준]** 대학의 특성을 반영할 수 있는 교육과정의 원칙과 원리를 자체적으로 개발하여야 하며, 이 원리와 원칙에 근거한 교육과정의 유형을 가지고 있어야 한다.

**[우수기준]** 미래의 의사에게 요구되는 자기주도 학습능력, 평생교육 태도 등의 함양을 위한 학생 중심 교육과정을 운영하고 있으며, 학사 일정이 전 학년에 걸쳐 균형을 이루고 있다.

[주] 학사 일정이 전 학년에 걸쳐 균형을 이루고 있어야 한다는 것은 의사국가시험 준비 등으로 마지막 학년도 학사일정이 조기에 종결되거나 파행적으로 운영되어서는 안 된다는 의미를 포함한다.

**[참고자료]**

비치: 1. 교육과정 개발을 위한 회의 자료 등

## 2-1-5 교육과정 개선과 관리를 담당하는 교육관련 위원회의 구성 및 권한과 책임은 적절한가?

**[보고서 기술내용]** 대학의 교육과정 개선과 관리를 담당하는 교육관련 위원회의 현황, 역할, 권한 및 책임 등을 기술하고, 최근 5년간 교육과정 개선의 절차, 방법 및 실적을 기술한다. 또한, 해당 위원회가 교육과정 개선을 위해 사용한 예산을 항목별로 구분하여 기술한다.

[주] 교육과정의 개선과 관리를 담당하는 위원회의 ① 교수, 학생, 행정직원의 참여 정도 ② 교육과정의 개발과 평가를 위한 교육전문가의 참여 여부 ③ 교실의 이해관계 및 정치적 영향과는 무관하게 교육과정을 관리 및 운영할 수 있는 대학 내규 등이 있는지에 대해서도 기술한다.

**[권장기준]** 교육과정 개선과 관리를 담당하는 위원회의 구성, 역할, 권한 및 책임 등이 분명하게 설정되어 있고, 최근 5년간 합리적인 과정을 통해 교육과정을 개선한 실적이 있다. 또한, 교육과정 개선을 위한 위원회의 연간 예산이 2,000 만 원 이상이다.

[주] 교육과정 개선과 관리를 담당하는 위원회의 예산에는 각종 교육관련 위원회의 예산을 모두 포함하여 계산할 수 있다. 그러나 각 위원회에서 집행한 실제 교육비용(OSCE, PBL 등의 직접 비용), 의학교육학과(교실, 실 등), 학생지도위원회 등의 예산은 포함하지 않는다. 즉, 교육관련 위원회의 운용을 위한 예산(회의비, 세미나비, 연구개발비, 워크숍비 등)을 의미한다.

**[우수기준]** 교육과정 개선과 관리를 담당하는 기구(위원회 포함)가 월 1회 이상 정기적으로 개최되고, 교육과정 개선과 관리를 위한 예산이 연간 5,000만 원 이상이며, 학생들이 졸업 후 일하게 될 의료환경에 대한 정보를 수집하고 사회로부터 받은 피드백을 교육과정 개선에 활용한다.

**[참고자료]**

비치: 1. 교육과정 개선 관련 기구의 결정사항 대비 개선 실적 비교표

2. 교육과정 개선과 관련된 기구(위원회 포함) 규정

3. 교육과정 개선과 관련된 기구의 회의록

4. 교육과정 개선을 위해 사용한 예산 집행 내역

5. 의료환경 변화에 대한 모니터링 자료

## 2-2 기초의학 교육과정

**평 가 개 요**

기초의학 교육과정은 생물학적 기전에 대한 이해와  과학적 사고력 및 판단력을 함양시키고 연구방법을 교육하는 것으로 인체의 정상 구조와 기능, 질병의 발생원인과 질병 역학적 지식, 기본적인 실험수기 등을 습득할 수 있도록 적절한 교육내용과 시간으로 편성되어야 한다.

### 2-2-1 기초의학 교육과정은 적절한가?

**[보고서 기술내용]** 인체의 기본구조와 기능 및 병인을 이해하는데 필요한 기초의학 교육내용이 어느 학년에 어떻게 개설되어 있는지 그 내용을 기술한다. 이들 내용이 통합강의 또는 문제바탕학습(PBL)에 포함된 경우 어디에 어떻게 어느 정도 포함되어 있는지를 기술한다.

[주] 기초의학 교육내용(교실 개념이 아님)으로는 세계의학교육협회(WFME)의 기준인 해부학(anatomy), 생리학(physiology), 생화학(biochemistry), 면역학(immunology), 병리학(pathology), 약리학(pharmacology), 미생물학(microbiology), 유전학(genetics), 분자생물학(molecular biology), 생물물리학(biophysics), 세포생물학(cell biology) 예방의학(preventive medicine) 및 기생충학(parasitology) 등을 포함한다.

**[필수기준]** 세계의학교육협회에서 권고하는 기초의학 교육내용이 교육되어야 한다. 또한, 기초의학 교육과정은 사회의 건강요구에도 부응할 수 있도록 구성되어 있어야 한다.

[주] 세계의학교육협회는 기초의학 교육과정이 ① 신체의 구조와 기능에 관한 분자생물(molecular), 세포(cellular), 기관(organ) 및 신체전부(whole body)수준의 내용 ② 비정상적(병적)인 신체구조와 기능에 관한 내용 ③ 질병의 자연사, 병적 상태에 대한 신체 방어기전 및 질병에 대한 신체반응 등에 관한 내용 ④ 질병발생과 관련되는 유전적, 환경적, 사회경제적 요인에 관한 내용 및 연구방법 등의 내용을 다루어 함을 강조하였다.

### 2-2-2 기초의학 관련 실습교육은 적절한가?

**[보고서 기술내용]** 기초의학 관련 실습이 기초의학 관련 강의의 내용을 이해하는데 도움이 되도록 내용이 적절히 구성되어 있는지 실습 단원별 시간표와 함께 기술한다. 또한 기초의학 관련 실습시간은 이론을 이해하는 데 도움이 되고 과학적 사고와 연구방법을 교육시킬 수 있는 수준이어야 하기 때문에 기초의학 관련 전체 교육시간의 어느 정도를 차지하는지 기술한다.

**[필수기준]** 기초의학 관련 과목에 대한 실습(예: 기초의학 교과목별 또는 통합실습과정 내용)이 있고, 그 내용이 학습목표 범위 내에서 이루어지고 있다.

**[참고자료]**

부록 : 1. 기초의학 실습현황 자료(실습계획 및 내용)

### 2-2-3. 학생 1인당 기초의학 실험 실습비가 적정한가?

**[보고서 기술내용]** 최근 2년간 학생 실험·실습용 기초의학 실험 실습비 예산 내역을 기술하고 학생 1인당 배정된 기초의학 실험 실습비 (예산서상 학생경비 중 기초의학 학생 실험 실습비 총액 ÷ 전체 학생 수)를 계산하여 제시한다. 의학과 과목 실험 실습이 의예과에서 시행되는 경우라도 계산에서는 의예과를 제외한 의학과 학생을 전체 학생으로 간주한다.

**[필수기준]** 학생 1인당 기초의학 실험 실습비가 30만 원 이상이다.

**[참고자료]**

부록: 1. 기초의학 교실별 학생 실험실습비 배정 기준표

비치: 1. 기초의학 실험 실습비 집행 관련 서류

## 2-3 임상의학 교육과정

**평 가 개 요**

임상의학 교육과정은 일차 진료 수준의 전인적 의료문제 해결에 필요한 기본 임상 지식과 술기를 습득할 수 있고, 진단과 치료에 대한 의사결정 과정에 근거바탕의학(EBM)을 활용할 수 있도록 편성되어야 한다.
학생들이 이러한 교육내용을 경험할 수 있는 실습장소, 교육방법이 신중하게 선정되어야 한다. 또한, 학생들이 습득한 지식과 술기를 객관적으로 평가해야 한다.

### 2-3-1 임상의학 수업은 일차 진료 수준의 의료를 목표로 구성되어 있는가?

**[보고서 기술내용]** 임상의학 수업이 한국의과대학장협의회의 최근 학습목표 수준으로 구성되어 있는지를 기술한다. 또한, 과목중심 강의형태이든, 통합강의 형태이든 학습목표들이 교육에 어떻게 반영되어 있는지, 학습목표에 따른 임상의학 수업계획서가 있는가를 기술한다.

[주] 임상의학 수업이 일차 진료 수준의 의료를 목표로 구성되어야 한다는 것은 의과대학 졸업생들이 졸업후 수련 교육에 필요한 기본적인 지식, 술기 및 태도를 함양하고, 최소한의 기본 진료능력을 갖추어야 한다는 것을 의미한다.

**[필수기준]** 임상의학 수업이 한국의과대학장협의회의 최근 학습목표 수준의 내용으로 구성되어 있고, 이에 따른 임상의학 수업계획서가 있으며, 실제 임상의학 수업에 반영되고 있어야 한다.

**[참고 자료]**

비치: 1. 임상의학 수업계획서 및 임상의학 시간표

### 2-3-2 임상실습에 대한 준비 교육이 있는가?

**[보고서 기술내용]** 학생들이 임상실습에 진입하기 전에 임상실습 준비 교육을 언제 어떻게 실시하고 있는지 기술한다. 임상실습 준비 교육 내용에 환자 면담 기법과 기본 술기에 대한 교육이 포함되었는지를 기술한다.

**[필수기준]** 환자 면담 기법과 기본 술기에 대한 교육이 포함된 임상실습 전 준비교육이 있어야 한다. 이러한 과정이 블록(block)으로 개설된 경우에는 최저 4주 이상, 지속적인 과정(longitudinal course)인 경우에는 최소 2학기 이상 개설되어야 한다.

[주] 임상실습 준비교육은 ICM(Introduction to Clinical Medicine), FCM (Fundamentals of Clinical Medicine)등을 의미한다. 블록 또는 지속적인 과정인 경우 학생 1인당 교육받는 시간은 40시간 이상이 되어야 한다.

### 2-3-3 임상실습 지침서가 학생들에게 제공되고 있으며, 실제로 활용되고 있는가?

**[보고서 기술내용]** 학생들에게 일차 진료 수준의 임상실습 지침서가 각 과별로 제작 및 배포되고 있는가를 기술한다. 임상실습 지침서에는 학생들에게 반드시 필요한 기본 술기를 고르게 포함하고 있는지와 그 내용이 실제 실습에 어떻게 활용되고 있는지를 기술한다. 또한 과별로 일차 의료 수준의 진료 능력을 갖추기 위해 필요한 흔한 증상, 징후 및 질병(최소한의 필수환자군)을 구체적으로 제시하고 있는지와 노력을 기술한다. 또한, 임상실습 중 학생의 환자 진료 범위와 책임에 대한 규정이 있으면 기술한다.

[주] 기본 술기란 활력징후 측정, 정맥 채혈, 혈액도말 검사, 심전도, 정맥 및 근육 주사, 요로 도자, 관장, 환부 소독 및 봉합, 비위관 삽입 등과 같은 간단한 진단 또는 치료 술기를 말하며, 이는 대학에 따라서 차이가 있을 수 있다.

[주] 최소한의 필수환자군 이란 일차 진료 수준의 능력을 갖추기 위해서 각 과별로 꼭 알아야 할 흔한 증상, 징후 또는 질병을 말한다.

**[필수기준]** 학생들이 반드시 습득해야 하는 기본 술기가 포함된 임상실습 지침서가 학생들에게 제공되고, 실제 활용되어야 한다.

**[우수기준]** 임상실습 지침서에는 기본 술기와 관찰 술기가 구분되어 기술되어 있으며, 실습과마다 최소한의 필수환자군을 제시하여 학생들이 경험할 수 있도록 하고 있다. 또한 임상실습 중에 학생이 할 수 있는 술기와 진료 범위에 대한 자체 규정이 있다.

[주] 관찰 술기란 실습 중 1회 이상 관찰해야 하는 술기들로서 이는 학교에 따라서 차이가 있을 수 있다. 예) 복막 천자, 늑막 천자 및 생검, 골수 천자를 통한 흡인 및 생검, 척수천자, 감염 (예: AIDS, 만성 간염 등) 환자의 정맥 채혈법, 연조직 세침 흡인술, 심낭천자, 중심정맥 카테터 삽입술, 혈액투석 및 복막 투석, 상 하부 위장관 내시경, 기관지 내시경, 심폐소생술. 또한 학생들은 술기의 목적과 방법 및 위험도를 설명할 수 있어야 한다.

**[참고자료]**

비치: 1. 각 과별 임상실습 지침서

### 2-3-4 임상실습이 충분한 기간 실시되고 있는가?

**[보고서 기술내용]** 임상실습 편성시기 및  과목의 구성과 각각의 실습 시간을 기술하며, 임상실습 편성 원칙 또는 근거 자료(설문결과 등)에 대해서 기술한다.

**[필수기준]** 최소 50주, 매주 36시간에 준하는 임상실습이 이루어져야 하며, 내과, 외과, 산부인과, 소아과, 정신과 및 응급의학과 실습은 반드시 포함되어야 한다.

### 2-3-5 임상실습을 위한 장소가 다양하며 외래 환자 중심 실습은 적절한가?

**[보고서 기술내용]** 임상실습 장소와 방법이 일차 진료 수준이 되도록 적절히 고려되고 있는지를 관련 자료와 함께 기술한다. 즉, 학생들의 임상실습 장소가 3차 병원 이외에 1, 2차 병원이 포함되어야 하며, 핵심과와 지원과(예를 들어, 영상의학과, 진단검사의학과) 및 가정의학과 등에서 외래 및 입원 환자 중심 실습이 어떻게 이루어지고 있는지 기술한다.

[주] 핵심과는 내과, 외과, 산부인과, 소아과, 정신과, 응급의학과를 의미하며, 응급의학과는 외래 실습으로 간주한다.

**[필수기준]** 핵심과, 지원과 및 가정의학과 임상실습은 3차 병원 이외의 1, 2차 병원에서도 이루어져야 하고, 핵심과의 외래 환자 중심 실습은 전체 실습 시간의 25% 이상 되어야 한다.

**[참고자료]**

부록: 1. 각 과별 임상실습 편성표(실습 장소, 실습 시간 표시)

## 2-3-6 임상실습 방법은 다양한가?

**[보고서 기술내용]** 핵심과를 중심으로 실습 학생 수와 병동 회진, 발표, 의무기록 작성, 소규모 강의, 수술실 관찰 및 지원, 학생 예진, 외래 참관, 임상 수기 관찰 및 시행 등과 같은 임상실습 방법을 자세하게 기술한다.

> [주] 학생인턴제 란 학생신분이면서도 병동에서 진료팀의 일원이 되어 회진, 수술 참관 및 보조, 증례발표, 기본적 임상 술기(문진, 신체검사, 처치 등), 의무 기록지 작성, 및 당직 등의 인턴에 준하는 역할을 수행하면서 환자진료에 직접 참여하는 임상실습 형태이다.

**[필수기준]** 병동 회진, 발표, 의무기록 작성, 소규모 강의, 수술실 관찰 및 지원, 외래 참관, 임상 수기 관찰 및 시행 등과 같은 다양한 방법을 통해서 임상실습 교육이 이루어져야 한다.

**[우수기준]** 외래 실습 시 학생이 예진을 시행하고 있으며, 병동 실습 시에는 학생인턴제를 시행하고 있다. 또한, 기본 술기의 습득을 위해서 술기실습실을 활용한다. 진단과 치료에 대한 의사결정 과정에 근거바탕의학(EBM)을 활용한다.

**[참고자료]**

부록: 1. 각 과별 임상실습 시간표

현지 확인: 1. 술기실습실

### 2-3-7 학생이 자유롭게 선택할 수 있는 임상실습 과정이 있는가?

**[보고서 기술 내용]** 임상실습 과정 중 학업성취가 부족한 부분을 보충하거나, 더욱 심화시킬 목적으로 학생들이 자유롭게 선택하여 임상실습을 받을 수 있는 과정이 있는지에 대해서 기술한다.

[주] 학생들이 자유롭게 선택한다는 것은 실습 장소에 구애받지 않고 학생들이 희망하는 곳에서 실습을 할 수 있다는 것을 의미한다.

**[권장기준]** 임상실습 과정 중 학업성취가 부족한 부분을 보충하거나, 더욱 심화시킬 목적으로 학생들이 자유롭게 선택하여 임상실습을 할 수 있는 과정이 최소한 2주 이상 편성되어 있다.

**[참고자료]**

비치: 1. 학생들이 자유롭게 선택할 수 있는 과정 편성표 및 실제 학생 선택 현황

### 2-3-8 임상실습 책임교수가 지정되어 있고, 학생에 대한 지도감독과 되먹임이 적절한가?

**[보고서 기술내용]** 모든 임상실습 장소(원외인 경우 임상 지도의, 또는 외래 교수)의 책임교수 또는 교육 전담 전공의 명단을 기술하고, 학생에 대한 지도 감독과 되먹임의 시기, 방법, 내용 및 횟수 등에 대해서 자세히 기술한다.

**[권장기준]** 임상실습 책임교수와 교육 담당 전공의가 과별로 임명되어 있고, 학생에 대한 지도 감독과 되먹임을 하고 있다.

**[참고자료]**

비치: 1. 각 과별 시행 자료

현지 확인: 1. 학생 면담

## 2-4 인문 · 사회의학 교육과정

**평 가 개 요**

의학 교육과정에 인간과 생명, 질병과 환자, 그리고 의사의 역할과 사회에 대한 포괄적 이해와 성찰을 위한 인문 · 사회의학 교육이 포함되어야 한다.

### 2-4-1 인문 · 사회의학 관련 과목이나 강좌를 개설하고 있는가?

**[보고서 기술내용]** 순수 의학 관련 교과목 이외에 의학과 학생들을 위해 개설한 인문 · 사회의학 과목 또는 강좌를 열거하고 과목별로 교육목표, 내용, 교육방법 등을 기술한다.

[주] 인문·사회의학의 분야는 의사학, 의료윤리, 의학교육, 의료정보, 철학, 어문학, 사회학, 경영학, 의사법학, 인류학, 심리학, 행동과학, 보완대체의학 등을 의미한다. 예방의학(의료관리, 의료정책, 역학 등)과 법의학은 교육내용에 따라 인문·사회의학 분야로 분류 가능하다.

**[필수기준]** 임상관련 문제에 대한 판단과 도덕적 의료행위를 가능하게 해주는 행동과학, 의료윤리, 의료관련법규, 기타 인문 · 사회의학 과목이나 강좌가 학년별로 최소 1개 이상 개설되어 있고, 의학전문직업성(medical professionalism)과 의사소통에 관한 수업내용이 포함되어야 한다.

**[우수기준]** 임상에서 실제적으로 적용할 수 있는 태도와 의사소통술 등을 습득할 수 있도록 다양한 교육방법(역할극, 시청각 매체 활용, 표준화 환자, 각종 컨퍼런스 참여 등)이 활용되고 있다.

**[참고자료]**

부록: 1. 인문 · 사회의학의 교육목표, 교육내용 및 방법

비치: 1. 인문 · 사회의학 수업계획서

### 2-4-2 인문 · 사회의학과 관련된 현장체험학습 또는 사회봉사 프로그램이 있는가?

**[보고서 기술내용]** 인문 · 사회의학 관련 현장체험 학습이나 사회봉사 프로그램을 열거하고 그 내용을 기술한다. 이런 교육에 대한 학생들의 반응을 기술한다.

**[권장기준]** 인문 · 사회의학에 관련된 현장체험학습 또는 사회봉사 프로그램이 개설되어 있으며, 대학의 행정적, 재정적 지원 실적이 있고, 학생의 현장체험학습 및 사회봉사활동 참여율이 20% 이상이다.

**[참고자료]**

비치: 1. 현장체험학습 또는 사회봉사 프로그램 계획서 및 실적

2. 학생 반응도 조사 설문지 및 결과분석 자료

### 2-4-3 보완대체의학 또는 통합의학에 대한 교육이 있는가?

**[보고서 기술내용]** 보완대체의학(complementary and alternative medicine) 또는 통합의학(integrative medicine)의 이해를 돕기 위해 어떻게 접근하고 있는지를 기술한다.

[주] 미국 국립보건원 산하 기관인 NCCAM(National Center for Complementary and Alternative Medicine)의 정의에 의하면 보완대체의학이란 정통의학(conventional medicine) 이외의 모든 비주류 의학과 민간요법을 통틀어 지칭하고 통합의학이란 정통의학과 보완대체의학을 합친 의학을 말한다. 이러한 보완대체의학의 주제로는 침구학, 자석요법, 약초요법, 무용치료, 카이로프랙틱, 음악치료, 아유르베다, 맛사지 요법, 요가, 기공 등이 있다.

**[권장기준]** 보완대체의학 또는 통합의학에 대한 학생들의 이해를 돕기 위한 시간이 있다.

**[참고자료]**

비치: 1. 보완대체의학 또는 통합 의학 관련 수업 교재

## 2-5 수업방법과 강좌평가

| 평 가 개 요 |
|---|
| 대학의 수업은 학습목표와 수업계획서에 기초하여 학생들의 학습효과를 최대한 높이기 위한 방법으로 이루어져야 하며, 교수 또는 학생들에 의해서 평가되고 그 결과가 적절하게 활용되어야 한다. |

### 2-5-1 구체적으로 기술된 과목별 학습목표를 가지고 있으며, 대학은 이것이 적절히 활용되도록 노력하고 있는가?

**[보고서 기술내용]** 대학에서 사용하고 있는 과목별 또는 강좌별 학습목표를 제시하고 이 학습목표가 잘 활용될 수 있도록 하기 위해 대학이 어떤 노력을 하고 있는지를 기술한다.

**[필수기준]** 학습목표가 반영된 수업계획서가 있고, 수업계획서를 지키도록 지시한 공문, 세미나, 교수회의 등을 연 2회 이상 실행하여야 한다.

**[참고자료]**

비치: 1. 학습목표가 반영된 수업계획서

2. 수업계획서를 지키도록 지시한 공문, 세미나, 교수회의 등의 실적

## 2-5-2 강의식 수업 이외에 다양한 수업방법이 활용되고 있는가?

**[보고서 기술내용]** 학습효과를 높이고 스스로 학습할 수 있는 능력을 함양하기 위하여 전통적인 강의식 수업 이외에 학생들이 능동적으로 학습에 참여할 수 있는 학습방법들(문제바탕학습, 협력학습, 세미나, 인터넷 보조학습, 발표, 자료실 운영 등)을 과목별 또는 강좌별로 기술한다.

[주] 객관구조화진료시험(OSCE)은 임상실습 평가 영역에서 기술한다.

**[필수기준]** 능동적으로 학습에 참여할 수 있는 학습방법들(문제바탕학습, 협력학습, 세미나, 인터넷 보조학습, 발표, 자료실 운영 등)을 최소 3가지 이상 수업에 활용하고, 전체 학생이 매 학기 1과목 이상에서 문제바탕학습, 협력학습, 세미나학습, 발표학습, 또는 온라인학습 등을 경험하도록 해야 한다.

**[우수기준]** 의과대학 전체 교과목 중 실습 이외의 교과목 1/3 이상에서 문제바탕학습, 협력학습, 세미나학습, 발표학습, 또는 온라인학습 등의 방법 중 한 가지 이상의 방법으로 수업이 진행되고 있다.

**[참고자료]**

현지확인: 1. 강의식 수업 이외의 교육방법 운영 시설(PBL방, PC학습실, 자료실 등)

### 2-5-3 대학에서 실시하는 강좌평가가 있으며, 그 결과가 수업에 적절히 반영되고 있는가?

**[보고서 기술내용]** 대학에서 실시하는 강좌평가 내용을 관련 서식과 함께 기술하고 평가결과의 활용내용을 기술한다.

**[필수기준]** 대학에서 실시하는 강좌평가가 전체 교과목의 80% 이상에서 시행되고 있으며, 그 결과를 교수와 학생에게 알려주고 이를 수업개선에 활용하여야 한다.

**[우수기준]** 모든 강좌에서 평가가 시행되고 있을 뿐만 아니라 그 결과가 교수, 학생 등 이해관계자 모두에게 공개되고 있다.

**[참고자료]**

부록: 1. 강좌평가서식

비치: 1. 대학에서 실시하는 강좌평가서 및 개선 실적

## 2-6 학생 학습평가

**평 가 개 요**

학생들의 학업성취에 대한 평가는 지식뿐만 아니라 술기 및 태도 영역을 포함해야 하며, 타당하고 신뢰로운 방법으로 이루어져야 한다. 또한, 기초의학 및 임상의학 전반에 대한 학생들의 학업성취도를 정기적으로 측정하고 그 결과를 활용하여야 한다.

### 2-6-1 학생들의 학업성취도에 대한 평가는 지식, 술기 및 태도 영역에서 다양한 방법으로 이루어지고 있는가?

**[보고서 기술내용]** 지식, 술기 및 태도 영역에 대한 학생들의 학습 평가내용(평가형태, 빈도, 내용, 비중 등)을 수업 과목별로 기술하고, 이들 평가방식에 대한 학생들의 반응을 기술한다.

[주] 임상실습에 대한 학업성취도 평가는 2-6-2 문항에서 기술하며, 여기서는 수업에서의 학업성취도에 대해서 다룬다.

**[권장기준]** 학생들의 지식, 술기 및 태도 영역에 대한 학업성취도를 평가하기 위해서 전체 수업 과목의 60% 이상에서 다양한 방법을 사용하고 있다.

**[우수기준]** 전체 수업 과목에서 지식, 술기, 태도 영역에 대한 총합평가(summative evaluation)성격의 학업성취도 평가가 다양한 방법으로 이루어지고 있으며, 학생 피드백을 목적으로 하는 형성평가(formative evaluation)를 전체 수업 과목의 반 이상에서 실시되고 있다.

**[참고자료]**

비치: 1. 기초, 임상, 인문사회과목의 학습평가자료

현지확인: 1. 학생들의 학습평가의 개선을 위한 대학의 노력과 실적 점검

2. 2-3개 교과목을 무작위로 표집하여 평가계획에 따라 실제 평가가 이루어지고 있는지를 확인

### 2-6-2 임상실습 평가가 적절하게 실시되고 있는가?

**[보고서 기술내용]** 임상실습에서 학생들의 지식, 술기 및 태도를 그 영역별로 어떻게 평가하고 있는지 평가기준을 과목별로 기술하고, 임상실습교육에 대한 학생들의 평가내용을 기술한다.

**[필수기준]** 임상실습 평가는 실습과목별로 지식, 술기, 태도에 관한 명확한 지침에 근거하고 있어야 한다. 또한, 지식수준 평가를 위한 필기나 구술시험, 발표나 과제물, 태도평가를 위한 출석이나 실습 참여도, 그리고 술기평가를 위한 체크리스트, 진찰능력, 의료인과의 관계, 동료와의 관계, 의사소통능력, 발표능력 등 임상에서 필요한 최소능력이 점검되고 있어야 한다.

**[우수기준]** 모든 실습과에 자체적인 임상실습 평가표가 개발되어 있고 평가는 평가표대로 진행되고 있다. 또한, 평가결과는 실습 종료시점에 학생이 입회한 가운데 통보되고 이때 최소교육목표(최소한의 필수환자군) 도달이 안 되었을 경우 부족분에 대한 대안 교육을 실시한다. 또한, 학생들에 의한 임상실습 교육평가가 실시간으로 이루어지고 대학은 평가결과를 공개하여 임상실습 교육 개선에 반영하고 있다.

**[참고자료]**

비치: 1. 2-3개 임상실습 과목에 대한 평가서 및 내용

2. 임상실습 교육에 대한 학생들의 평가표

현지확인: 1. 핵심과 임상실습에 대한 학생평가 점검 및 선택과정의 실습에 대한 무작위 표출 2-3개과 점검

2. 학생면담을 통한 임상실습 교육에 대한 평가

### 2-6-3 기초의학 및 임상의학 교육성취도에 관한 종합평가 노력은 적절한가?

**[보고서 기술내용]** 과목 또는 강좌별 평가 이외에 기초의학이나 임상의학을 전반적으로 이해하고 있는지를 평가하는 기초 종합평가나 졸업시험 형태의 임상 종합평가가 지식, 태도, 술기영역에서 실시되고 있는지와 그 내용 및 효과를 기술한다.

**[필수기준]** 기초 종합평가나 졸업시험 형태의 임상 종합평가 등 학생들의 교육성취도를 종합적으로 평가하는 노력이 있으며, 이러한 종합평가의 효과를 분석한 실적이 있어야 한다. 종합평가는 임상수행능력 평가를 위한 다양한 수행평가를 포함하고 있어야 한다 (예, OSCE, CPX 등) .

**[참고자료]**

비치: 1. 기초, 임상 종합평가와 실기측정인 수행평가 내용 및 평가분석 자료

# 3 학생 영역

## 3-1 입학 정책 및 학생 선발

| 평 가 개 요 |
|---|
| 대학은 건학이념과 사회적 책무성에 기초한 입학정책을 갖고 있어야 하며, 학생선발방법과 기준이 합리적이고 절차는 공정해야 한다. |

### 3-1-1 대학은 학생선발방법을 명시한 입학정책을 가지고 있는가?

**[보고서 기술내용]** 대학의 입학정책과 학생선발제도에 대해 기술한다. 학생선발방법 및 기준이 합리적인지와 학생선발의 절차가 공정한지를 기술한다.

**[권장기준]** 대학의 건학이념과 사회적 책무성에 맞는 다양한 학생선발방법을 명시한 입학정책이 있고 선발방법과 절차가 공정하다.

**[참고자료]**

비치: 1. 학생선발 제도 관련 자료

2. 학생 선발위원회 관련 서류

### 3-1-2 대학은 학생선발제도의 개선을 위한 노력을 하고 있는가?

**[보고서 기술내용]** 입학정책과 학생선발제도의 적절성을 평가하고, 그 결과를 기초로 한 개선 실적 및 계획을 기술한다.

[주] 입학정책과 학생선발제도의 적절성 평가는 학생선발기준 및 절차에 대한 평가와 입학한 학생들의 학업성취도 등에 대한 평가에 기초한다. 개선 실적 및 계획에는 입학정책 관련 기구 및 위원회 등의 활동을 기술한다.

**[권장기준]** 대학은 입학정책과 학생선발제도를 정기적으로 평가하고 있으며 선발제도와 졸업생이 갖추고 있어야 할 자질과의 관계를 명시하고 있다.

**[참고자료]**

비치: 1. 입학제도에 대한 학내외 감사자료

2. 입학정책 관련 기구 및 위원회 활동실적

## 3-2 학생지도 체제

| 평 가 개 요 |
| --- |
| 대학은 학생들의 학습, 생활, 진로의 3가지 분야를 위한 학생 지도 체제를 갖추고 이를 활발히 운영해야 하며, 학생들이 교내·외 전문 활동을 할 수 있도록 관심을 갖고 적절히 지도해야 한다. |

### 3-2-1 대학은 적절한 학생 지도체제를 갖고 있으며, 그 체제와 운영이 전문적으로 이루어지고 있는가?

**[보고서 기술내용]** 의과대학의 학생 지도체제를 학습, 생활, 그리고 진로 지도 부분으로 나누어 기술하고 이를 총괄하는 체제로 학생지도위원회가 구성되어 활발하게 운영되고 있는지를 기술한다. 의과대학 전체 학생을 대상으로 하여 학년별, 그룹별, 개인별 지도체계가 있는지와 정기적으로 지도교수와 학생이 만나는 체제가 확립되어 있는지 기술한다. 또한 의과대학 내에 전문 상담체계(상담실, 상담연구소 등)가 갖추어진 경우 그 역할과 최근 3년간 실적을 기술한다.

**[필수기준]** 의과대학의 학생 지도를 담당하는 학생지도위원회를 구성하여 활발하게 운영하여야 하고, 학생지도 분야를 학습, 생활, 그리고 진로 지도로 나누어 전문적이면서도 통합적으로 관리하여야 한다.

**[우수기준]** 의과대학 학생의 원활한 학교생활을 위한 전체학생, 학년별, 그룹별, 개인별 지도를 위한 지도 체제가 확립(예: 학생 지도위원회 위원장 등의 역할이 명확하게 규정되어 있고, 학생들에게 공지되고 이해하고 있는가?)되어있고 운영이 전문적으로 이루어지고 있다(예: 상담실이 설치되어 있고, 상담원이 1인 이상 전업제로 고용되어 활동하고 있는가?).

[주] 학생 생활지도 및 상담이 전문적으로 운영된다는 것은 이를 전담하는 전문인력이 있다는 의미이다. 의과대학의 학생과, 학생담당 부학장 등이 지도하는 경우에는 전문적 운영으로 해석하지 않는다. 의과대학이 아닌 본교에 상담센터가 있는 경우에는 지리적으로 학생들의 접근성이 용이하며, 의과대학 학생을 위한 별도의 전문상담체제가 갖추어져 있고, 실적이 있으면 인정한다.

**[참고자료]**

부록: 1. 학생지도 체제 관련 자료

2. 학생 전체, 학년별, 그룹별, 개인별 지도교수 현황 관련 자료

비치: 1. 학생 지도교수 제도 관련 서류 (상담일지 포함)

2. 전문 상담 체제 관련 자료 (전문상담 연구소 및 연구원)

### 3-2-2 학생들의 학습, 생활, 진로 지도 내용은 적절한가?

**[보고서 기술내용]** 학생들의 학습, 생활 및 진로 지도 내용을 기술한다. 특히, 학습 지도는 의과대학이 유급생과 휴학생이 많다는 특수 상황을 고려하여 학습부진 학생에 대한 지도 과정 및 지도 실적을 기술한다. 또한, 생활지도는 학생들의 주거, 학교생활, 교우관계 등을 분석하고 지도하기 위한 대학의 노력을 기술하며, 학생들의 진로지도를 위해 대학이 가지고 있는 프로그램 또는 진로지도 실적을 관련 자료와 함께 기술한다.

[주] 진로지도 결과 졸업생의 진로 등에 대한 내용은 3-3-1의 졸업생의 진로와 관련된 평가문항에서 다룬다.

**[권장기준]** 대학은 학생들의 학습지도를 위하여 정기적으로 학생들의 학업성취 정도를 확인하고, 학습부진 현상을 보이고 있는 학생들을 적극적으로 지도하고 있다. 또한, 생활지도 및 진로지도를 위해 교수와 학생간에 정기적인 만남이 이루어지고 있으며, 그 결과를 기록하여 보존하고 있다.

**[우수기준]** 학습부진 학생에 대한 구체적인 지도 실적이 있고, 학년별 유급율의 급격한 변동이 있는 경우 문제점을 분석하는 등의 노력이 있다. 또한, 유급학생을 구제하기 위한 제도(대학간 학점 교류 제도, 방학을 이용한 계절학기 제도 등)가 있다.

**[참고자료]**

부록: 1. 연도별 학생 유급 현황

비치: 1. 학생 생활 및 진로 지도 실적 (상담일지 포함)

현지확인: 1. 일부 교수 및 학생면담을 통한 지도교수 제도 운영 확인

### 3-2-3 대학은 학생들의 건전한 교내·외 전문 활동을 권장하고, 적절한 지도와 지원을 하고 있는가?

**[보고서 기술내용]** 최근 2년간 학생들의 자발적인 학술, 연구, 사회봉사, 동아리 활동내용과 이들 활동에 참여하는 학생들의 규모를 기술하고(단, 대학이 계획하고 시행하는 행사는 제외), 이들 활동에 대한 대학의 지원내용을 기술한다. 또한 대학이 학생들의 전문 활동을 권장하는 과정에서 학생들과 연관된 문제에 대한 학생대표의 참여에 관련된 내용도 기술한다.

[주] 의과대학 학생의 자발적 학술, 연구, 사회봉사, 동아리활동은 장차 의사로 일생을 살아가는데 있어서 매우 중요한 경험인 동시에 교육이다.

**[권장기준]** 대학은 학생들의 자발적인 교내·외 활동을 지원하고 있으며, 전문 활동을 권장하고 지원하는 단계에서 학생 대표를 참여시키고 있다. 또한, 학생 활동과 학생 조직(학생 자치 조직과 교육 위원회에의 참여, 기타 학생관련 위원회나 조직에의 참여 그리고 사회적 활동들을 포함)이 장려되고 활성화되어 있다. 또한 대학은 학생들의 자발적인 전문 활동을 재정적으로 지원하고 있다.

**[참고자료]**

부록: 1. 학생 기초의학 및 임상연구 참여  관련 자료

2. 학생의 학술, 연구, 사회봉사, 동아리 발표 현황 관련 자료

3. 학생 활동 지원과 관련된 예·결산 자료

비치: 1. 학생의 학술, 연구, 사회봉사, 동아리 발표 현황 관련 자료

2. 학생활동 지원 실적

현지확인: 1. 의학과 학년별 학생 대표단(총대) 및 동아리 대표 약 2-3명 면담

## 3-3 학생 복지 제도 및 시설

**평 가 개 요**

대학은 장학 제도를 갖추어 학생에게 경제적 도움을 주어야 하고 학생 복지를 위한 기숙사나 기타 복지 시설을 갖추어야 한다. 대학은 학생들이 질병 치료를 위해 쉽게 병원을 이용할 수 있도록 해야 한다.

### 3-3-1 학생 장학제도가 있으며, 장학금 지급 비율은 적절한가?

**[보고서 기술내용]** 대학의 장학제도를 기술하고 최근 3년간 장학제도, 장학금 종류별 지급 인원 및 지급 금액에 대한 내용을 기술한다. 또한, 최근 3년간 등록금 전액 대비 교내외 장학금 총액의 비율은 얼마인지도 기술한다.

[주] 등록금 전액 대비 장학금 지급비율= (학생 1인당 장학금/평균 등록금) x 100
동창회 기금을 이용한 대여 장학금 이외의 장학금 제외

**[필수기준]** 학생 장학제도가 수립되어 있으며, 등록금 대비 장학금 지급비율이 연 평균 10 % 이상이 되어야 한다.

**[우수기준]** 등록금 전액 대비 장학금 지급비율이 연 평균 20 % 이상이다.

**[참고자료]**

부록: 1. 학내·외 장학 제도 및 내용

### 3-3-2 장학생 선발은 공정한가?

**[보고서 기술내용]** 장학생 선발 관련 규정, 기준의 엄정성, 선발기준의 다양성, 절차의 합리성, 선발 결과 등을 구체적으로 기술한다.

**[권장기준]** 장학생 선발 규정이 있고, 다양하고 엄정한 기준과 합리적인 절차에 따라서 선발하고 있다.

**[참고자료]**

비치: 1. 교내·외 장학회 운영 및 장학생 선발 관련 규정

### 3-3-3 재학생들의 주거 현황을 분석하고 있으며 기숙사의 운영 실태는 적절한가?

**[보고서 기술내용]** 재학생의 주거 현황을 기술한다. 기숙사가 있는 경우, 입실 현황, 기숙사의 수요 대비 공급 비율 및 타 지방 학생들을 위한 우선 배정 여부에 대하여 기술한다. 기본시설(냉난방, 채광, 욕실 등)과 편의시설 (세탁실, 휴게실, 공동취사실 등)을 갖추고 있으며 위생적으로 관리되고 있는지, 시설 만족도 조사 결과와 의학과 학생들을 위한 배려(규정) 유무에 대해서도 기술한다. 그리고 현재 기숙사가 없는 경우는 앞으로의 계획을 기술한다.

**[권장기준]** 학생의 주거 및 기숙사 입실 현황에 대한 조사 자료가 있으며, 기숙사의 수요 대비 공급 비율을 파악하여 타 지방 학생들을 우선 배정하고 있다. 기본시설(냉난방, 채광, 욕실 등)과 편의시설 (세탁실, 휴게실, 공동취사실 등)을 위생적으로 관리하고 있으며, 시설 만족도 조사를 하여 운영에 활용하고 있다. 또한 의학과 학생들을 위한 배려 규정이 있다.

**[우수기준]** 희망 입사학생 전원을 수용할 수 있는 규모의 의대전용기숙사가 있으며, 실습병원이 떨어져 있는 경우 임상실습 중인 학생을 수용할 수 있는 기숙사 시설을 실습 병원에서 제공하고 있다.

[주] 의대전용 기숙사에는 독립가옥 또는 본부 기숙사의 일부라도 의대생이 사용하는 공간이 있는 경우도 포함 됨

**[참고 자료]**

부록: 1. 학생 주거 및 기숙사 입실 현황 조사결과

2. 타 지방 학생의 기숙사 입실비율

비치: 1. 기숙사 시설 및 관리 자료

2 . 시설 만족도 조사 자료

현지확인: 1. 기숙사 시설 확인 및 학생 면담

### 3-3-4 학생복지 및 편의시설이 고루 갖추어져 있고 그 수준도 적절한가?

**[보고서 기술내용]** 학업에 충실할 수 있는 면학 분위기 조성을 위하여 학생회방, 동아리방, 매점, 남·여학생 휴게실, 식당, 자동판매기, 학생 개인용 캐비넷, 현금지급기 등의 복지 및 편의 시설을 갖추고 있는 지를 기술한다. 이러한 시설들이 학교 내에 학생들의 생활공간 주위에 위치하고 있어 편리하게 이용할 수 있는가와 시설의 면적이 충분한지를 기술한다. 또한, 편의 및 복지 시설의 만족도를 조사하여 운영과 관리에 활용하고 있는지를 기술한다.

**[필수기준]** 남·여 휴게실, 식당, 매점, 운동 시설, 자동판매기, 개인용 캐비넷, 학생회방, 동아리방, 현금지급기 등이 적절하게 갖추어져 있어야 한다.

**[참고자료]**

부록: 1. 복지 및 편의 시설 현황

비치: 1. 복지 및 편의 시설 운영 및 관리 자료

현지확인: 1. 복지 및 편의시설

### 3-3-5 학생들의 건강관리와 상담을 담당하는 직원이나 체계가 있는가?

**[보고서 기술내용]** 학생 개인의 건강관리와 상담을 위한 체계(보건소, 양호실, 병원을 직접 이용 등), 인력 현황 및 이용 실태를 기술한다. 또한 학생 건강관리 규정과 시설이 대학 내에 있는지와 최근 3년간 건강관리 및 상담 자료를 기술한다.

**[권장기준]** 학생 건강관리 규정과 시설이 대학 내에 있다. 학생들의 건강관리 및 상담을 담당하는 직원이 보건소 또는 양호실에 있으며, 또는 학생이 필요한 경우 병원을 쉽게 이용할 수 있는 체계가 있다. 또한 심리검사(예: MMPI, 문장완성검사, 성격검사 등)를 실시하여 예비의사로서 학생의 심리상태를 파악하고 학생지도에 활용하고 있다.

**[우수기준]** 학생들의 건강관리를 위해 입학, 의학과 진입 및 임상실습 진입 시에 각각 건강검진, 예방접종 및 예방활동을 실시하고 있다.

**[참고자료]**

부록: 1. 건강관리 및 상담 직원과 체계에 대한 규정

2. 최근 3년간 이용실태 및 활동 업적 자료

현지확인: 1. 학생 면담

## 3-4 졸업 후 진로 및 학습 성과

**평 가 개 요**

의과대학 졸업생 중에는 임상의학 이외의 분야, 특히 기초의학 연구 및 교육 분야로 진출하는 사람도 있어야 하고, 의사국가시험에도 되도록 전원 합격할 수 있어야 한다.

### 3-4-1 임상의학 이외 분야의 진출을 위한 대학의 지원은 적절한가?

**[보고서 기술내용]** 졸업후 10-20년간 임상의학 이외의 분야로 진출한 졸업생 현황과 대학의 노력을 기술한다. 임상의학 이외의 분야라 함은 대한의학회에서 정하고 있는 임상의학 분야의 전공과목을 제외한 것이다.

**[권장기준]** 임상의학 이외 분야의 전문가를 확보하기 위한 정책(장학금 및 특별지원금 등)과 실적이 있거나, 졸업후 10-20년간 연간 입학정원 1%의 졸업생이 임상의학 이외의 분야로 진출하고 있다.

[주] 특별지원금은 생활비, 학회 참여 지원비, 기타 학업관련 비용을 의미하고 기초의학자 양성 정책을 포함한다.

[주] 사람 수는 반올림하지 않는다.

**[우수기준]** 우리나라에서의 기초의학 인력과 보건행정 및 기타 의사가 필요한 분야의 인력 규모로 볼 때, 의사가 필요한 분야로 진출한 경우가 졸업후 10-20년간 연간 입학정원의 5%이상 이다.

**[참고자료]**

부록: 1. 졸업생의 진로 현황

### 3-4-2 의사국가시험 합격률은 적절한가?

**[보고서 기술내용]** 최근 5년간 의사국가시험 합격률을 재학생과 졸업생으로 나누어 기술하고, 전체 합격률을 전국 평균합격률과 비교하여 기술한다.

**[권장기준]** 의사국가시험의 합격률을 매년 전국의과대학의 합격률과 비교하여 급격한 변동(예를 들면, 급격한 불합격자의 증가)이 있는지를 분석하여 문제점을 개선하려는 노력을 하고 있다.

**[참고자료]**

부록: 1. 최근 5년간 의사국가시험 합격률 현황 및 분석 자료

2. 의사국가시험 불합격자를 위한 방안 자료

# 4 교수 영역

## 4-1 기초 및 임상 전임교수

| 평 가 개 요 |
| --- |
| 의과대학은 기초의학과 임상의학 교육을 충실히 할 수 있도록 능력 있는 적정 수의 전임 교수를 확보하고 있어야 한다. |

### 4-1-1 기초의학 전임교수가 적정 수 확보되어 있는가?

**[보고서 기술내용]** 기초의학 전임교수의 수를 전공 분야별로 기술하고, 이를 필수기준과 비교하여 현재의 상태를 기술한다. 아울러, 최근 5년간의 기초의학 전임교수 임용 및 퇴직 현황을 기술한다.

**[필수기준]** 모든 의과대학은 세계의학교육협회가 권고하는 기초의학 교육을 위해 각 분야(13개 분야)에 최소 1명 이상, 총 25명의 기초의학 교원을 확보하고 있어야 한다.

[주] 분야는 과목 또는 교실을 의미하는 것이 아니라 교육내용을 의미한다.

[주] 겸임교수는 정원에서 제외하나 대학에서 정규 급여를 지급하는 기금교수는 정원에 포함한다. 겸임교수라 함은 타 단과대학 및 타 대학교에 적을 두고 있으면서 해당 대학에 교수직을 겸임하고 있는 경우를 말한다. 기금교수는 교육부 정원에 해당되지는 않지만 교육, 연구 및 진료에 있어 전임교수와 같은 활동을 하는 것이 확인된 경우 이를 포함한다.  단, 임상의학 교실에 속하지 않은 전임연구 교수는 성격상 기초의학 교원으로 인정할 수 있다. 이 경우 이들은 별도로 명기해야 한다. 한편, 기초 해부병리와 임상병리과가 통합된 대학의 경우 기초의학 병리학과 교수로는 6명까지만 인정한다.

**[우수기준]** 13개 기초의학 교육영역 중 90%이상에서 교육경력 10년 이상인 전임교수가 1인 이상이 있다.

[주] 기초의학 전임교수의 교육경력은 의과대학 졸업 후의 고등교육 기관 근무 경력으로 조교 경력을 포함하여 교육 관련 분야 경력을 의미한다. .

**[참고자료]**

부록: 1. 기초 전임교수 현황

비치: 1. 인사기록 (급여대장 포함)

### 4-1-2 인문 · 사회의학 전임교수가 적정 수 확보되어 있는가?

**[보고서 기술내용]** 인문 · 사회의학 전임교수의 수를 전공 분야별로 기술하고, 이를 필수기준과 비교하여 현재의 상태를 기술한다. 아울러, 최근 5년간의 인문 · 사회의학 전임교수 임용 및 퇴직 현황을 기술한다.

**[권장기준]** 인문 · 사회의학 분야 전임교수 5인 이상을 확보하고 있어야 한다.

[주] 인문 · 사회의학의 분야는 의사학, 의료윤리, 의학교육, 의료정보, 철학, 어문학, 사회학, 경영학, 의사법학, 인류학, 심리학, 행동과학 등의 전공을 의미한다. 단, 겸무교수는 해당되지 않는다.

[주] 예방의학(의료관리, 의료정책, 역학 등)과 법의학은 전공에 따라 인문 · 사회의학으로 분류한다.

### 4-1-3 기초 및 인문 · 사회의학의 교육과 연구를 보조하는 인력은 적절한가?

**[보고서 기술내용]** 기초 및 인문 · 사회의학의 교육과 연구를 보조하는 조교의 명단을 교실 또는 관련 분야별로 기술하고, 이들의 교육 및 연구지원 업무 비율을 각각 %로 기술한다.

**[권장기준]** 기초 및 인문 · 사회의학의 교육과 연구를 보조하는 인력으로 대학에서 직접적으로 재정적 지원을 하는 조교, 연구원 이 교수 2인당 1명 이상이다.

[주] 조교, 연구원의 1인당 재정지원의 기본수준은 적정급여과 대학원 전액등록금을 합한 것을 의미한다. 재정지원이라 함은 연구비, 장학금 등을 포함한다.

**[우수기준]** 기초 및 인문 · 사회의학의 교육과 연구를 보조하는 인력으로 조교, 또는 대학에서 직접적으로 재정적 지원을 하는 연구원을 포함하여 교수 1인당 1명 이상이다.

**[참고자료]**

부록: 1. 기초 및 인문 · 사회의학의 교육과 연구보조인력 현황

비치: 1. 인사기록 및 급여대장 또는 연구비 및 장학금 지급 대장

### 4-1-4 임상의학 전임교수가 적정 수 확보되어 있는가?

**[보고서 기술내용]** 임상의학 전임교수의 수를 전공 분야별로 기술하고, 이를 필수 조건과 비교하여 현재의 상태를 기술한다. 아울러, 최근 5년간의 임상의학 전임교수 임용 및 퇴직 현황을 기술한다.

**[필수기준]** 20개 이상 진료 및 지원과목에 전임교수가 총 85명 이상 있어야 한다.

**[우수기준]** 임상진료 및 지원과의 90%이상에서 교육경력 10년 이상인 전임교수가 1인 이상이 있다.

[주] 임상의학 전임교수의 교육경력은 의과대학 졸업 후의 교육 기관 근무 경력으로 강사급(전임의, 연구 및 임상강사 등) 경력을 포함하여 교육 관련 분야 경력을 의미한다. 단, 인턴 및 전공의 수련 경력은 포함하지 않는다.

**[참고자료]**

부록: 1. 임상 전임교수 현황

비치: 1. 인사기록 (급여대장)

### 4-1-5 기초, 인문사회 및 임상 전임교수의 구성은 적절한가?

**[보고서 기술내용]** 의과대학 교원을 기초의학, 임상의학, 인문・사회의학 및 전체 교원으로 구분하여 성별, 연령별, 직급별, 출신학교별, 최종학력별로 그 현황을 기술한다.

**[권장기준]** 의과대학 전체 교원 중 동일 대학 출신 비율이 70% 이하이다.

**[우수기준]** 신임교원 임용 시 의과대학 전체 교원 중 동일 대학 출신 비율이 50% 이하이고 여교수 비율이 30 % 이상이거나 기초 및 임상교실에서 주임교수 공모제가 실시되고 있다.

**[참고자료]**

부록: 1. 기초, 인문사회 및 임상 전임교수 출신학교별 , 남녀별 현황

2. 주임교수 공모제 규정 및 실적

## 4-2 교수의 연구 및 학술활동

| 평 가 개 요 |
| --- |
| 교수는 성실히 연구를 수행하고 우수한 연구업적을 많이 발표하여 학문발전에 기여해야 한다. 또한, 대학은 교수들이 연구를 효과적으로 수행할 수 있도록 부설연구소 내실화 및 행정, 재정적 지원을 위해 노력해야 한다. |

### 4-2-1 교수들의 국내 연구 실적이 적절한가?

**[보고서 기술내용]** 최근 2년간 전임교수들의 국내발표 연구실적(원저, 종설, 증례보고, 저서, 역서)을 교수 1인당 평균 건수로 기술한다. 단, 공동연구의 경우 1인당 평균건수는 매 연구실적 1편을 저자 수(N)로 나누어 계산하되, 저자 간 소속(대학)이 다를 경우 제1저자와 교신저자는 연구실적 1편을 7할로 계산한다.

[주] SCI급과 Index Medicus 등재 논문은 4-2-2에서 기술한다.

**[필수기준]** 전임 교수 1인당 국내 연구실적 연평균이 1.0 이상이어야 한다.

**[우수기준]** 전임 교수 1인당 국내 연구실적 연평균이 2.0 이상이다.

**[참고자료]**

비치: 1. 최근 2년간의 연구실적 목록 (DB로도 제시 가능)

### 4-2-2 SCI급 과 Index Medicus 등재 논문수가 적절한가?

**[보고서 기술내용]** 최근 2년간 SCI급과 Index Medicus 등재 논문 수를 교실별 전임교수 수와 함께 기술하고, 이를 교수 100명당 연평균 논문 수로 계산한다. 단, 공동연구의 경우 1인당 평균건수는 매 연구실적 1편을 저자 수(N)로 나누어 계산하되, 제1저자 또는 교신저자의 소속 대학이 다를 경우 연구실적 1편의 70%를 제1저자 또는 교신저자 1인에게 부여할 수 있고, 나머지 30%는 다른 공동 연구자에게 균등하게 부여할 수 있다.

[주]: SCI급에는 SCI, SCIE, SSCI, A& HCI 등을 포함

**[권장기준]** 전임교수들의 최근 2년간 SCI급과 Index Medicus 등재 논문 수가 교수 100명당 연평균 30편 이상이다.

**[우수기준]** 전임 교수들의 최근 2년간 SCI급과 Index Medicus 등재 논문 수가 교수 100명당 연평균 60편 이상이다.

**[참고자료]**

비치: 1. 최근 2년간의 SCI급과 Index Medicus 등재 논문목록

### 4-2-3 대학 외부로부터 받은 연구비 수준은 적절한가?

**[보고서 기술내용]** 최근 2년간 대학 외부로부터 받은 연구비에 대해서 교실별 연구자, 연구제목, 연구기관 및 연구비 총액표를 만들고 전임교수 1인당 연평균 수혜 연구비를 계산하여 기술한다.

**[권장기준]** 전임 교수들의 최근 2년간 연평균 외부 연구비 수혜 비율이 전체 교수의 10% 이상 이거나, 전임교수 1인당 연평균 수혜 연구비가 500만 원 이상이다.

**[우수기준]** 전임교수 1인당 연평균 수혜 연구비가 1,000만 원 이상이다.

**[참고자료]**

비치: 1. 최근 2년간 대학 외부 연구비 수혜 현황 목록

2. 최근 2년간 대학 외부 연구비 수혜 현황 증빙자료

### 4-2-4 대학 내부 연구비 수준은 적절한가?

**[보고서 기술내용]** 최근 2년간 학내로부터 받은 연구비에 대해서 교실별 연구자, 연구제목, 연구기관 및 연구비 총액표를 만들고 전임교수 1인당 연평균 수혜 연구비를 계산하고, 연간 예산대비 연구비 비율도 계산하여 기술한다.

**[권장기준]** 전임 교수들이 최근 2년간 대학 내부로부터 받은 연구비가 전임교수 1인 당 연평균 100만 원 이상이거나, 연간 예산대비 연구비 비율이 3.0 % 이상이다.

**[우수기준]** 전임교수들이 최근 2년간 대학 내부로부터 받은 연구비가 전임교수 1인 당 연평균 300만 원 이상이거나, 연간 예산대비 연구비 비율이 5.0 % 이상 이다.

**[참고자료]**

비치: 1. 최근 2년간 대학 내부 연구비 수혜현황 목록

2. 최근 2년간 대학 내부 연구비 수혜현황 증빙자료

### 4-2-5 부설연구소의 연구 및 학술활동은 활발한가?

**[보고서 기술내용]** 대학이 운영하는 부설연구소 유무, 부설연구소 운영현황(전담 인력 포함)과 연구소를 통한 최근 2년간 교수들의 연구 및 학술활동 지원현황(연구비 지급액수 및 건수)을 기술한다.

**[권장기준]** 대학 자체의 연구기금으로 운영하는 부설 연구소가 있고, 부설 연구소 연구기금에 의해 운영된 최근 2년간 학술활동 지원 경비 및 연구비 지급 실적이 있다.

[주] 부설연구소 연구기금에 의해 운영된 연구비 지급 액수 및 지급 건수 등은 대학의 규모를 고려하여 평가자가 판단한다.

**[참고자료]**

부록: 1. 최근 2년간 부설 연구소 연구기금에 의해 지원된 학술활동 및 경비지원 내역과 지급된 연구비 지급액수 및 연구비 지급 건수 목록

비치: 1. 최근 2년간 부설 연구소 운영내역(회의자료 및 예·결산 관련자료)과 연구기금에 의해 지급된 최근 2년간 연구비 지급액수 및 지급 증빙자료

## 4-3 교수개발 실태

**평 가 개 요**

대학은 교수들의 교육, 연구 및 진료 능력 개발을 위한 각종 연수 교육 기회를 제공해야 하며, 합리적인 교수 업적평가 제도를 갖추고 적절하게 활용하여야 한다.

### 4-3-1 대학은 학내외 의학교육 관련 교수연수 기회를 적절히 마련하고 있으며 교수들의 참여 정도가 적절한가?

**[보고서 기술내용]** 최근 2년간 대학이 자체적으로 주관했던 순수 의학교육 관련 연수와 일반 교수연수 중 의학교육 관련 프로그램이 포함되었던 것을 구분해서 개최장소, 일시 참석인원, 주요 내용 등을 기술한다. 의학교육관련 연수 활동에는 교수들의 단합과 리더십, 그리고 조직문화의 향상을 위한 다양한 활동도 포함시킬 수 있다.

**[필수기준]** 교원을 대상으로 하는 의학교육 관련 연수 교육은 재직 교수 기준으로 연간 교수 1인당 4시간 이상이어야 한다.

[주] 재직교수에는 장 · 단기 해외 연수자는 제외한다.

**[우수기준]** 의과대학 신임교원을 위한 의학교육 연수과정이 최소 30시간 이상 실시되고, 교수업적평가 규정에 의학교육 연수 실적이 의무화되어 있다. 또한, 년 4회 이상 대학이 주관하는 의학교육 연수 관련 활동이 있다.

**[참고자료]**

비치: 1. 의학교육 관련 세미나 워크샵 등 관련 자료(책자, 프로그램 참석자 명단 등)

**4-3-2 대학은 교수들의 장 · 단기 해외연수 및 국내외 학회 참석을 위한 재정적 지원체제를 적절히 갖고 있는가?**

**[보고서 기술내용]** 교수들의 장·단기 해외연수 및 국내외 학회 참석을 장려하기 위한 재정적 지원체계(등록비, 항공료, 체제비 등)가 갖추어져 있는지를 최근 2년간 지원 실적 내용과 함께 기술한다. 연구년(안식년 포함) 제도를 시행하는 경우 그 내용과 실적도 함께 기술한다.

**[필수기준]** 교수들의 장·단기 해외연수 및 국내외 학회 참석을 위한 재정적 지원이 있어야 하며 연간 교수 1인당 100만 원 이상 지급해야 한다.

[주] 재정적 지원에는 병원의 지원도 포함한다.

**[우수기준]** 교수들의 장·단기 해외연수 및 국내외 학회 참석을 위한 안정적 재정을 확보하고 있으며, 학회참석을 독려하기 위해 연간 교수 1인당 500만 원 이상 지급하고 있다.

**[참고자료]**

비치: 1. 최근 2년간 장기 및 단기 해외연수를 위한 재정지원 실적자료.

2. 단기학회 참가를 장려하기위한 재정적 지원체계 규정.

### 4-3-3 교수업적평가가 이루어지고 있으며 그 결과가 적절하게 활용되고 있는가?

**[보고서 기술내용]** 전년도 교수업적평가(교육·연구·봉사) 서식을 제시하고 평가내용, 기준 및 그 결과 활용 등에 대해 기술한다.

**[필수기준]** 교수업적 평가제도가 정립되어 있고 교수들의 직위승진과 호봉승진에 반영되고 있어야 한다.

**[우수기준]** 교수업적평가가 직급별, 기능별로 세분화되어 있고(예: 조교수, 부교수, 정교수, 인문사회, 기초, 임상, 연구전임 등), 직위승진, 호봉승진에 관한 규정이 세분화되어 있다. 교육, 임상, 연구 등 계열별 전문교수가 양성되고 별도의 업적평가제도가 있다.

**[참고자료]**

부록: 1. 교수 업적평가 제도 및 현황(평가기준, 실제 평가자료 등)

# 5 시설·설비 영역

## 5-1 교육 시설·설비

| 평 가 개 요 |
| --- |
| 대학은 학생 교육을 위한 충분한 기본시설과 지원시설을 갖추어야 하며, 이를 효율적으로 관리하고 유지·운영하여야 한다. |

### 5-1-1 대학은 학생 교육을 위한 각종 시설·설비를 충분하게 갖추고, 효율적으로 관리·유지·운영하고 있는가?

**[보고서 기술내용]** 대학의 강의실, 실험실습실 및 교육지원 시설(강의실외 학습·토론 공간, 컴퓨터실, 술기실습실, 체육시설 등) 현황과 각 시설의 여건을 기술한다. 강의실 및 교육지원 시설은 각 시설의 용도에 적합한 시설과 여건(멀티미디어 시설, 조명, 냉난방, 방음, 환기 및 청결 상태 등)을 갖추고 있는지, 실험실습실은 교육과정에 부합하는 시설·설비를 갖추고 있는지 기술한다. 또한, 학생 교육을 위한 각종 시설, 설비 등의 관리·유지·운영 체계에 대해서 기술한다.

**[필수기준]** 교육과정의 특성과 학생 규모를 고려한 충분한 수의 학생 전용 강의실 및 교육지원시설을 갖추고 있고, 각 시설은 용도에 적합한 설비와 여건(멀티미디어 시설, 조명, 냉난방, 방음, 환기 및 청결 상태 등)을 갖추어야 한다. 실험실습실은 교육과정에 필요한 기본시설 및 실험실습장비를 갖추고, 이러한 시설, 설비를 관리·유지·운영하기 위한 인력과 적절한 규모의 예산이 편성·집행되어야 한다.

[주] 대학의 강의실, 실험실습실 및 교육지원 시설은 전체 학생 수 및 교육과정의 특성을 고려하여 적절성을 판단한다. 또한, 시설·설비의 관리 및 활용실태는 ① 시설배치 ② 관리조직(인력, 역할분담) ③ 관리 예산 및 지원 ④ 업무의 효율성 및 효과성 ⑤ 심미성 ⑥ 공해방지시설의 적절성 등을 중심으로 평가한다.

**[우수기준]** 학생 임상교육을 위한 적정한 규모의 술기실습실(Clinical Skills Lab) 또는 표준화환자를 이용한 교육 및 평가시설을 갖추고 있으며, 교육 및 관리를 전담하는 인력이 확보되어 있다.

[주] 적정한 규모의 술기실습실이란 임상술기 실습을 위한 기본장비를 갖추고 있는지, 학생수 대비 그 규모는 적절한지, 특정 전공교실에 포함되지 않고 독립된 공간에 설치되어 있어 학생들이 편리하게 이용할 수 있는지 등을 의미한다. 또한, 표준화 환자를 이용한 교육 및 평가시설은 대학의 교육과정에 따라 학생 임상실습 교육 및 평가에 지속적으로 활용되고 있는 시설이어야 한다. 우수기준은 술기실습실 등과 관리 인력이 있고 실제로 교육과정과 연계되어 효과적인 운영이 되고 있는 경우에만 인정한다.

**[참고자료]**

비치: 1. 실험실습실 기자재 보유 목록 (실험실습실에 확보된 기자재 목록만 비치)

현지확인: 1. 강의실, 실험실습실, 교육지원시설 확인

2. 교육기본시설 및 지원시설 관리 상태 확인

3. 시설관리 예산 집행 실적 확인

4. 시설 및 설비 관리 직원 면담

### 5-1-2 임상실습 교육을 위한 대학부속 교육병원을 확보하고 있으며, 병원 내에 학생교육 시설을 갖추고 있는가?

**[보고서 기술내용]** 임상실습 교육을 위한 대학부속 교육병원의 규모, 교육병원으로서의 역할, 병원 내 학생 교육 시설 및 학생 학습공간에 대해 기술한다. 임상실습 교육이 이루어지는 병원이 여러 개인 경우에는 각각에 대해서 기술한다. 병원의 학생 학습공간은 필수 기준을 참고하여 확보된 수, 실 면적 및 실제 활용에 대해서 기술한다.

**[필수기준]** 대학은 교육과정이 의도하는 임상실습 경험을 학생들에게 제공하기 위해 교육병원으로서의 역할에 적합한 유효병상 500병상 이상의 대학부속 교육병원을 확보하고 있어야 한다. 효과적인 임상실습 교육을 위하여 병원 내에 각종 교육시설을 갖추고 있으며, 실습학생 20명당 1개씩의 학생 학습공간을 확보하고 있어야 한다.

[주] 병원 내 각종 교육시설은 임상실습 교육과정에 따라 학생교육을 위해 실제로 사용되는 시설을 의미한다. 단, 술기실습실이나 표준화환자 이용 시설 등은 5-1-1에서 기술하며, 도서관 관련 시설은 5-1-3에서 기술한다. 또한 학생 전용 공간에서 전공의, 간호사 등 타 인력과 공동으로 사용하는 공간은 제외하며, 학생들이 임상실습 기간 중에 학습과 휴식을 취할 수 있는 공간만을 계산한다. 실습병원이 지리적으로 떨어져 있는 경우에는 각 병원별로 학생 교육시설 및 학생 전용공간이 갖추어져 있어야 한다.

**[우수기준]** 필수기준을 충족하고, 병원 내 학생 전용 공간이 핵심실습 과목별(내과학, 외과학, 산부인과학, 소아과학, 정신과학, 응급의학)로 확보되어 있다.

**[참고자료]**

현지확인: 1. 대학부속 교육병원의 규모와 역할 확인

2. 교육병원내 교육지원시설 및 학생 학습공간 확인

3. 학생면담: 병원 내 교육지원시설 및 학생 학습공간 활용 실태 확인

**5-1-3 대학은 교수의 연구와 학생의 교육을 지원하기 위한 학술정보서비스 체계를 구축하고 편리하게 이용할 수 있도록 하고 있는가?**

**[보고서 기술내용]** 교수의 연구와 학생의 교육을 지원하기 위한 학술정보서비스 체계에 대해서 기술한다. 도서관의 CD-ROM, on-line DB를 포함한 단행본, 학술지, 전자저널 등의 확보 실태와 그 적절성을 기술하며, 학술정보서비스와 관련된 인력, 최근 3년간의 예산의 변동 상황을 기술한다. 대학 및 교육병원이 지리적으로 떨어져 있는 경우에는 각 단위별로 학술정보서비스 체계를 기술한다.

[주] 학술정보서비스가 대학 본부의 중앙도서관에 포함되어 있을 경우, 의과대학 교수 및 학생을 위한 학술정보서비스가 어떻게 이루어지고 있는지 기술한다. 전자도서를 포함한 단행본, 전자저널을 포함한 학술지, CD-ROM, on-line DB 등은 의학과 관련된 것만 기술한다. 인력 및 예산도 별도로 구분하여 기술한다.

**[필수기준]** 의과대학 교수의 연구와 학생의 교육을 지원하기 위한 독립적인 학술정보서비스 체계가 구축되어 있어야 한다. 교수와 학생들이 중요한 학술도서 및 학술지에 쉽게 접근할 수 있고, 매년 지출되는 예산(전자도서를 포함한 단행본, 전자저널을 포함한 학술지, CD-ROM, on-line DB 등)이 교수와 학생 1인당 50만 원 이상이다. 교수의 연구 및 교육과 학생의 학습을 지원하기 위한 전문 인력(의학전문 사서)이 1명 이상 확보되어 있다.

[주] 학술정보서비스 체계가 독립적으로 구축되어 있다는 것은 대학의 도서관 또는 온라인 시스템을 통한 학술정보서비스가 의과대학 교수의 연구와 학생의 교육을 지원하기 위해 독립적으로 운영되고 있는가를 의미한다. 또한, 대학과 병원이 지리적으로 떨어져 있는 경우에도 동일한 학술정보서비스를 제공받을 수 있어야 한다.

[주] 접근성은 전자저널을 포함한 학술도서 및 학술지의 열람 및 출력의 용이성을 의미하며, 교수와 학생 1인당이라 함은 전체 교수 수와 학생 수를 합한 총 수에서의 1인당을 의미한다.

**[우수기준]** 매년 지출되는 예산이 교수와 학생 1인당 150만 원 이상이고, 전문 인력(의학 전문 사서) 1인이 담당하는 교수와 학생이 150명 이하이다.

**[참고자료]**

현지확인: 1. 의학 전용도서관의 규모와 시설, 도서 및 저널 확보 상태

2. 전산화 프로그램을 통한 학술정보서비스 실태

3. 교수·학생 면담: 학술정보서비스의 접근성 확인

4. 의학 전용도서관의 최근 5년간 예결산 자료

**5-1-4 대학은 교육정보화 시스템을 구축하고, 구성원이 편리하게 이용할 수 있도록 하고 있는가?**

**[보고서 기술내용]** 대학의 교육정보화 시스템 구축 및 활용 현황에 대해서 기술한다. 교육정보화 시스템에는 학사관리 시스템, 홈페이지 활용, E-Learning 시스템 등에 대해서 기술하고 그 적절성을 기술한다. 또한, 교육정보화 시스템 구축 및 활용과 관련된 최근 3년간의 집행 예산을 함께 기술한다.

**[권장기준]** 대학의 학사 및 교육과정 운영과 관련된 교육정보화시스템이 구축되어 있으며, 구성원들이 편리하게 이용할 수 있다.

**[우수기준]** E-Learning 시스템이 갖추어져 있으며, 실제 활용되고 있다.

[주] E-Learning은 교수·학습 자료를 단순히 제공하는 수준이 아니라 상호작용이 가능한 시스템이어야 하며, 시스템을 통하여 교육내용이 제공되고, 실제 교수·학습이 이루어지며, 학습관리가 이루어짐을 의미한다.

**[참고자료]**

현지확인: 1. 교육정보화 시스템 시연

2. 교수, 학생, 직원 면담: 교육정보화시스템의 활용도 확인

## 5-2 연구 관련 시설과 설비

| 평 가 개 요 |
| --- |
| 대학은 전임강사 이상 교원의 개인 교수실과 교수들의 연구 공간과 시설을 충분히 확보해야 한다. |

### 5-2-1 대학은 개인 교수실과 그 실내 장비를 적절하게 갖추고 있는가?

**[보고서 기술내용]** 전임강사 이상의 개인 교수실의 확보실태(전체 교원 수에 대한 개인 연구실 수의 비율)와 교수실의 실내 설비(조명, 냉난방, 방음, 환기, 채광, Lan 설치 등) 상태를 기술한다.

**[필수기준]** 전임강사 이상 교원의 개인 교수실 확보 수준이 80%이상은 되어야 하며, 적절한 실내 설비를 갖추고 있어야 한다.

[주] 개인 교수실은 교수 단독으로 사용하는 공간으로 진료실 겸용으로 사용하는 공간은 제외하며, 적정한 크기의 공간이어야 한다.

**[우수기준]** 전임강사 이상 교원의 개인 교수실(진료실 겸용 제외) 확보율이 100% 이상이고, 크기 및 실내 설비가 잘 갖추어져 있다.

**[참고자료]**

부록: 1. 전임강사 이상의 전 교수의 개인 교수실 확보 실태

현지확인: 1. 교수실의 크기, 내부 실내 설비 및 주위 환경 확인

### 5-2-2 대학은 교수들의 연구를 위한 충분한 공간과 시설 및 설비를 갖추고 있는가?

**[보고서 기술내용]** 각 과의 독립 또는 교수 공동의 실험연구실 확보 현황과 연구 기자재 확보 및 관리 실태를 기술한다.

**[권장기준]** 각과가 독립 또는 공동 연구실을 가지고 있고, 그 시설 및 시설 내 연구기자재가 잘 관리되고 있다. 각과의 독립 또는 공동 연구실 규모가 충분하고, 우수한 연구 기자재가 확보되어 있으며, 교수들이 각 연구공간을 효율적으로 사용할 수 있도록 지원되고 있다.

**[참고자료]**

부록: 1. 연구시설 현황

비치: 1. 교수 연구 공간과 시설의 확보 현황 및 운용 상태

2. 개인 혹은 공동 연구기자재의 종류와 보유 현황 및 관리 상태

# 6 졸업 후 교육 영역

## 6-1 대학원 교육

**평 가 개 요**

대학은 학구적인 환경 조성을 위해 대학원 교육과정을 적절하게 편성하여 운영하여야 하며, 다양한 경력자가 대학원에 진학할 수 있도록 하여야 한다. 또한, 대학원생을 지도하는 의학교육 프로그램은 지적인 도전을 촉진시키는 환경에서 수행되어야 한다.

### 6-1-1 대학원 교육은 체계적인 계획하에 충실하게 수행되고 있고 학위관리는 적절한가?

**[보고서 기술내용]** 전공과목별 교육과정과 그 해당 강의 계획서를 제시하고 이 계획서가 잘 지켜지도록 대학이 노력한 내용과 학습평가 방법을 기술한다. 또한 학위관리는 어떻게 하고 있는가를 기술하고 특성화 된 대학원편제(학·석사, 석·박사 통합, 의사박사 복합, 학제간 복합학위제도 등)와 그 운용내용을 기술한다.

**[권장기준]** 전공과목별로 학부와 연계성이 있는 학습목표가 포함된 수업계획서가 있고, 대학은 이 계획서에 충실하게 강의와 학습평가가 진행되도록 공문, 교수회의 등을 실시하고 있으며, 관련규정에 합당하게 학위관리를 하고 있다.

**[우수기준]** 특성화 된 대학원편제(학·석사, 석·박사 통합, 의사박사 복합, 학제간 복합학위제도 등)와 학위종류(MD + PhD, MD + MBA, MD + JD, MD + MPH 등)가 운용되고 있다.

**[참고자료]**

부록: 1. 전공과목별 교육과정 및 특성화 된 대학원편제

비치: 1. 전공과목별 수업계획서
2. 전공과목별 학습평가결과
3. 구체적인 학위관리 규정
4. 수업계획서 준수와 엄격한 학위관리를 하도록 지시한 공문 또는 교수회의 내용
5. 학부와 대학원에 모두 개설되어 있는 학과의 교육과정의 연계정도 확인

### 6-1-2 대학원 업무체계는 적절한가?

**[보고서 기술내용]** 대학원 업무를 전담하는 기구 및 인력현황을 기술한다.

**[권장기준]** 대학원 업무를 전담하는 기구 및 인력과 그 운용을 위한 예산 및 그 집행이 합리적이다.

**[참고자료]**

부록: 1. 대학원 행정기구표

비치: 1. 대학원 업무를 전담하는 기구 및 인력과 예산 및 집행현황

### 6-1-3 학생선발 및 학생지원체제는 적절한가?

**[보고서 기술내용]** 최근 2년간 대학원생의 경력에 따른 구성과 납부 등록금 총액 대비 장학금 총액의 비율, 연구지원 시설 및 실험실습비의 확보상태를 기술한다.

**[권장기준]** 특성화 된 대학원편제에 합당한 다양한 경력을 소유한 학생을 확보하고 있으며, 납부 등록금 총액 대비 30% 이상의 교내외 장학금 또는 특별 지원금이 있으며, 대학원생 전용 연구공간이 있다.

**[참고자료]**

부록: 1. 최근 2년간 대학원생의 경력(학부전공 포함)에 따른 구성

2. 최근 2년간 납부 등록금 총액 및 장학금 총액과 실험실습비의 확보상태

비치: 1. 최근 2년간 전체 대학원생의 학부전공 확인서류

2. 최근 2년간 장학금 지급내역

3. 실험실습비의 지급내역

4. 대학원생을 위한 연구 공간 확인

# Post-2주기 의학교육 평가인증기준

## 평가영역·부문별 기준 수

| 평가 영역 | 평가부문 | 문항 수 | 기준 수 | |
|---|---|---|---|---|
| | | | 기본 | 우수 |
| 1. 대학 운영 체계 | 1-1 대학설립 | 3 | 3 | 0 |
| | 1-2 대학행정 및 운영 체계 | 6 | 6 | 2 |
| | 1-3 대학재정 | 3 | 3 | 0 |
| | 1-4 대학발전 계획 | 3 | 3 | 0 |
| | 1-5 개선노력 | 3 | 3 | 2 |
| | 소계 | 18 | 18 | 4 |
| 2. 기본의학교육과정 | 2-1 교육과정 개요 | 3 | 3 | 0 |
| | 2-2 교육과정 개발과 지원 | 5 | 5 | 3 |
| | 2-3 교육과정 구성과 운영 | 16 | 16 | 4 |
| | 2-4 학업성취 평가 | 3 | 3 | 3 |
| | 2-5 교육과정 평가와 개선 | 3 | 3 | 0 |
| | 소계 | 30 | 30 | 10 |
| 3. 학생 | 3-1 입학정책과 학생선발 | 4 | 4 | 1 |
| | 3-2 학생지도 체제 | 6 | 6 | 5 |
| | 3-3 학생복지와 안전 | 7 | 7 | 6 |
| | 3-4 졸업 후 진로 | 2 | 2 | 1 |
| | 소계 | 19 | 19 | 13 |
| 4. 교수 | 4-1 전임교수 | 6 | 6 | 6 |
| | 4-2 교수업무 | 5 | 5 | 3 |
| | 4-3 교수개발 | 7 | 7 | 3 |
| | 소계 | 18 | 18 | 12 |
| 5. 시설·설비 | 5-1 교육 시설·설비 | 7 | 7 | 3 |
| | 5-2 연구 시설·설비 | 2 | 2 | 1 |
| | 소계 | 9 | 9 | 4 |
| 6. 졸업 후 교육 | 6-1 대학원 교육 | 3 | 3 | 1 |
| | 소계 | 3 | 3 | 1 |
| 합계 | | 97 | 97 | 44 |

의학교육 평가인증 기준

# 01 대학 운영 체계

## 1-1 대학의 설립

**평가개요**

대학은 건전하고 명시된 설립이념에 의하여 설립되고, 운영의 독립과 자율이 보장되어야 하며, 고유의 설립 목적 이외의 어떠한 이득이나 이해, 갈등관계에 놓여서는 안 된다. 또한, 대학의 설립주체는 충분한 재원과 능력을 바탕으로 설립이념의 구현을 위한 사회적 책임과 사회 공익적 기여에 충실하여야 하며 대학의 발전을 위하여 최대의 노력을 기울여야 한다.

### 1-1-1 대학은 설립이념을 갖고 있으며, 그 설립이념은 대학의 사회적 책임과 공공성 확보를 위한 내용을 포함하고 있는가?

**기본기준**

대학 설립이념이 기술되어 있다.

**보고서 기술내용**

대학 설립이념을 사회적 책임과 공공성 확보 측면에서 기술한다.

* 대학이라 함은 의과대학·의학전문대학원을 지칭함

**참고자료**

비치 : 1. 대학의 설립이념 관련 자료

## 1-1-2 대학의 자율성과 독립성이 보장되어 있는가?

### 기본기준

대학과 대학본부, 의료원, 학교법인 상호 간에 대학 운영에 관한 독립성과 자율성이 존중되어 있고, 이를 위한 제도적 장치가 마련되어 있다.

### 보고서 기술내용

대학이 종합대학의 일부이면 대학본부와 의과대학의 재정과 학사에 관한 역학 구도를 기술하고 의과대학의 자율성과 독립성에 대해 기술한다. 의료원 체제이면 대학 행정, 재정, 인사에 관한 운영의 독립성과 자율성을 기술한다. 설립주체가 학교법인이면 대학 간의 행정, 재정, 인사를 포함한 학사 운영에 관하여 기술한다. 이미, 독립채산제로 전환되어 있으면 전환하기 위한 과정과 관련 기구의 실적을 기술한다.

[주] 자율성과 독립성은 분리된 개념이 아니다. 대학의 수행능력은 전문성에 바탕을 두어야 한다. 의과대학이 외부(대학본부, 학교법인)의 적절치 못한 영향력에 의하여 의과대학 업무수행의 지장을 초래하거나 업무상 수월성의 문제가 제기되지 않도록 하여야 한다. 이 항목은 정량적인 지표보다는 대학업무(교육, 시설·설비, 인사, 행·재정 등)의 수행에 대한 총괄적인 견해를 기술하는 것이다. 중요한 개념은 의과대학 운영의 전문성에 입각한 대학 운영이다. 예를 들어, 대학교수 임용은 대학의 요구분석과 임용방식에 의한 것인가, 아니면 행정편의에 바탕을 둔 획일화 된 정책에 의한 것인지 등의 문제이다.

#### 참고자료

부록 : 1. 재단, 대학본부, 의료원과 의과대학의 역학구도를 보여주는 행정구조표
비치 : 1. 의과대학의 분권화를 위한 학칙, 위원회자료, 실적보고서 등
현지확인 : 1. 학장, 의무부총장, 총장, 재단이사장 면담

## 1-1-3 대학은 세계보건기구가 권장하는 사회적 책무성을 위한 노력을 하고 있는가?

### 기본기준

대학은 사회적 책무의 수행과 관련한 교육, 연구, 진료 방침을 갖고 있으며, 이러한 방침이 시행되고 있다.

### 보고서 기술내용

세계보건기구(WHO)가 권장하는 사회적 책무성을 수행하기 위해 교육, 연구, 진료 부문에서 대학이 노력하고 있는 내용을 기술한다. 또한, 각 지역사회의 보건의료 관련 단체와의 연관성을 기술한다.

[주] 사회적 책무성은 대학의 교육, 연구, 진료 활동이 소속된 지역사회나 국가의 보건우선과제 지향적이어야 하는 의무이다. 보건우선과제는 정부, 의료단체, 의료인과 사회가 공동으로 선정하여야 한다(WHO "social accountability").

**참고자료**

비치 : 1. 사회적 책무성에 대한 대학의 방침과 책무성 수행 실적보고서
현지확인 : 1. 사회적 책무성에 대한 인지도와 활동 확인

## 1-2 대학 행정 및 운영 체계

**평가개요**

대학은 행정업무를 교육, 교수, 학생, 연구, 졸업 후 교육, 입학, 재정 등으로 구분하여야 하고, 이를 담당하는 책임자와 행정인력이 전문화되어 있어야 하며, 행정 업무 계획과 예산 집행에 대한 자율권이 대학 행정 책임자에게 있어야 한다. 또한, 대학 병원이 지역적으로 떨어져 있으면 교육과 연구를 표준화하기 위한 구조가 있어야 한다.

### 1-2-1 대학 운영에 필요한 업무가 적절하게 분장되어 있는가?

**기본기준**

교육, 교수, 학생 업무가 행정적 연관성을 가지고 분장되어 있으며, 별도의 보직자가 임명되어 있다.

**보고서 기술내용**

대학 행정 업무가 교육, 교수, 학생, 연구, 졸업 후 교육, 입학, 재정 등으로 구분되어 있는지와 담당 인력 현황을 기술한다. 교육, 교수 및 학생 분야를 반드시 포함하여 각 업무별 보직이 구분되어 있으며, 해당 업무가 보직자를 중심으로 기획, 실행되고 있는지 기술한다.

[주] 보직자란 별도의 보직 수당을 지급받는 자를 의미하며, 업무의 관련성에 따라서 한 명의 보직자가 두 개의 업무를 맡을 수 있다.

**우수기준**

교육, 교수, 학생, 연구, 졸업 후 교육, 입학, 재정 등의 분야에 각 보직자가 임명되어 있으며, 해당 보직자의 구체적인 활동 실적이 있다.

**참고자료**

비치: 1. 대학 행정 업무 체계도
2. 분야별 업무 회의록과 구체적인 활동 실적
3. 보직 수당 지급 내역

부록 : 1. 행정 기구표와 인력 구성

## 1-2-2 대학의 행정업무가 구분되어 있고, 행정직원은 분장업무별로 적절하게 확보되어 있는가?

### 기본기준

행정업무가 교육, 교수, 학생, 연구, 졸업 후 교육, 입학, 재정 등으로 구분되어 있고, 행정업무 담당 직원이 최소 5명 이상이다.

### 보고서 기술내용

대학 행정 업무가 교육, 교수, 학생, 연구, 졸업 후 교육, 입학, 재정 등으로 구분되어 있는지와 담당 인력 현황을 기술한다. 교육, 교수 및 학생 분야를 반드시 포함하여 각 업무별 행정직원이 별도로 확보되어 있는지 기술한다. 대학원 재학생을 행정조교로 쓰고 있으면 그 내용을 별도로 기술한다. 의과대학 행정 조직 내에 의예과, 간호학과, 의공학과, 대학원 등 타 학과가 함께 있으면 각각의 업무 분담과 담당 인력에 대해서 기술한다. 행정 조교, 대학원생 조교는 행정직원 수에는 포함하지 않는다.

[주] 업무의 관련성에 따라서 한 명의 보직자가 두 개의 업무를 맡을 수 있다.

#### 참고자료

비치: 1. 대학 행정 업무 체계도
2. 분야별 업무 회의록과 구체적인 관련 활동 실적

## 1-2-3 학장단의 전문성을 개발하고 유지하기 위한 체제가 적절한가?

### 기본기준

학장단의 직무 관련 전문성을 확보하기 위한 지속적인 개선 노력이 있다.

### 보고서 기술내용

임명 전후에 학장단의 전문성을 개발, 유지하기 위한 대학 내의 제도적 장치나 노력을 기술한다. 학장단의 구성에 이르기까지의 과정과 해당 분야에 대한 전문성을 확보하기 위한 워크숍, 세미나, 관련 학회 등에 참여한 실적을 기술한다.

[주] 학장단이라 함은 학장과 보직교수를 의미한다.

### 우수기준

교육, 교수개발, 연구역량 강화 등을 포함한 다양한 분야의 경력이 학장의 리더십 자격 요건으로 명시되어 있다.

[주] 학장 선임대상자의 자격요건 등이 선임규정 또는 대학의 규정/운영세칙/내규 등에 명시되어 있어야 한다. 보직예고제 등이 운영되고 있는지를 기술한다.

**참고자료**

비치 : 1. 학장단의 경력 현황
2. 학장선거 관리 규정
3. 학장단의 전문 분야별 학회나 워크숍 참석 현황

## 1-2-4 대학의 행정업무 계획과 예산 집행에 관해 학장이 자율권을 가지고 있으며, 책임경영 체제가 확보되어 있는가?

### 기본기준

대학 본부(또는 의료원)로부터 위임(또는 승인) 받은 대학의 행정 업무와 예산집행 권한이 학장에게 있고, 학장은 자신의 책임 하에 자율적으로 집행하고 있다.

[주] 학장이 주관하는 위원회에서 교수 채용, 승진 자격 요건과 외국 연수 자격 요건 등을 논의하고 있다는 명문화된 규정이 있어야 한다. 실험 실습비와 경상비의 집행이 학장 전결로 이루어지며, 의과대학 학사 일정이 학장 책임 하에 계획 관리되어야 한다.

### 보고서 기술내용

학장이 교수, 조교, 직원 임용 등 인사 관련 회의 및 의사결정 과정에 참여할 수 있는 범위를 기술하고, 자율적으로 사용할 수 있는 연간 총 금액, 전결 가능 업무와 금액을 기술한다. 또한, 학장 주관 하에 계획되고 결재된 사항이 제대로 집행되고 있는지를 기술한다.

[주] 학장이 자율적으로 집행할 수 있는 금액은 대학의 예산항목으로 반영되어 있는 업무추진비, 예비비 등을 의미하는 것은 아니다. 이 금액은 대학의 사명과 목표 달성을 위해 학장이 자율적으로 집행할 수 있는 정책적 재원(discretional fund)을 의미한다.

#### 참고자료

비치 : 1. 학장의 임무와 권한에 관한 규정
2. 전결 업무 규정
3. 의과대학 교수의 인사 규정

## 1-2-5 대학 운영을 위한 정책결정 구조와 절차가 적절하게 운영되고 있는가?

### 기본기준

대학 운영을 위한 정책결정 구조와 절차가 적절하다.

### 보고서 기술내용

대학 운영을 위한 정책결정 구조와 절차를 제시하고, 전체 교수 또는 주임교수 회의, 교육, 학생지도와 인사 관련 위원회를 포함한 각종 위원회와 같은 기구의 역할에 대하여 명문화된 규정을 근거로 기술한다. 또한, 각 의견 수렴 기구별 구성원, 역할분담, 활동실적 등을 기술하고 이들 기구에서 결정된 내용이 대학 운영에 얼마나 반영되고 집행되는지를 기술한다.

[주] 인사 관련 위원회의 선출직 위원은 1/3이상이어야 하며, 기타 위원회는 당연직 위원이 1/4이내이어야 한다. 또한, 각 위원회는 직급별, 분야별(기초, 임상 등)로 다양하게 구성되어 있어야 한다. 각종 위원회는 한 학기에 최소 1회 이상 개최되어야 한다.

#### 참고자료

부록 : 1. 각종 위원회와 같은 기구의 역할에 대하여 명문화된 규정
비치 : 1. 각 위원회, 주임교수회의, 전체 교수회의 회의자료 및 회의록
2. 교육, 학생지도와 인사 관련 위원회 활동실적

## 1-2-6 대학 부속 병원이 지리적으로 떨어져 있으면, 병원마다 학생 교육과 연구를 지원하는 대학 행정 구조가 있는가?

### 기본기준

지리적으로 떨어져 있는 각 부속병원마다 학생 교육과 연구를 총괄하는 보직자가 임명되어 있고, 교육과 연구를 지원하는 행정구조가 있다.

### 보고서 기술내용

대학의 (여러) 부속병원들이 지리적으로 떨어져 있으면 학생 교육과 연구를 총괄하는 보직자 또는 부서에 대해서 기술하고, 병원별로 학생 교육과 연구를 담당하는 체계, 업무추진 상황과 실적 등에 대해서 기술한다. 또한, 각 병원별 임상실습 교육을 표준화하기 위해 어떤 노력을 하고 있는지 기술한다.

**[주]** 부속병원 상호간에는 학생 임상실습 교육을 표준화하기 위한 정기적인 회의를 개최하여야 하고, 회의 결과에 따라 실제로 집행된 실적이 있어야 한다. 그리고 모든 교육 장소에서 제공하는 프로그램의 개발, 수행과 평가 작업에 학장단이 직접 관여하여야 한다.

### 참고자료

부록 : 1. 부속병원별 교육 연구지원 행정구조
비치 : 1. 교육과 연구 담당 총괄부서의 활동 자료
2. 병원별 담당 교수간의 회의 자료
3. 학장단의 실습 병원 방문 기록
4. 실습 학생 출입 현황 자료

## 1-3 대학 재정

**평가개요**

대학은 교육관련 운영에 필요한 재원을 확보하기 위해 장단기 계획을 수립하여야 한다. 또한 확보된 재원을 공정하고 합리적으로 배분하고 효율적으로 활용하며, 내·외부 감사제도를 통해 그 효과를 극대화할 수 있도록 노력하여야 한다.

### 1-3-1 대학 운영을 위한 예산편성제도는 적절한가?

**기본기준**

대학은 합리적인 예산편성제도를 마련하여 운용하고 있다.

**보고서 기술내용**

대학의 예산편성 시기, 구성원의 의견 수렴 과정과 예산편성 절차, 대학의 사업계획과 예산의 연계성, 예산심의 과정 등에 대하여 기술한다. 의료원 체제이면, 의과대학의 독립된 예산이 책정되어 있다.

[주] 대학은 예산을 편성할 때, 부서(교실, 과, 통합교과목)별 사업계획과 예산에 대한 의견을 수렴하여야 한다.

**참고자료**

비치 : 1. 대학의 예산 편성을 위한 의견 수렴 자료
2. 대학의 예산 편성을 위한 회의 자료

## 1-3-2 대학은 적절한 교육관련 재정을 확보하고 있는가?

### 기본기준

교육관련 재정이 적절히 편성되어 있다.

### 보고서 기술내용

교육관련 재정(학생 실험·실습비, 교육과정 개발과 운영비, 교수개발비, 국내외 교수 연수 지원비, 교육관련 세미나 개최비, 학생 봉사활동 지원비 등)확보 현황을 항목별로 기술하고, 대학의 노력과 성과에 대하여 기술한다. 모든 자료는 최근 6년간의 자료를 연도별로 작성하여 변화의 추이를 확인할 수 있도록 한다.

**참고자료**

비치 : 1. 대학 예산 집행 내역 서류
2. 교육관련 재정확충을 위한 활동 서류

## 1-3-3 대학 내·외부 감사제도와 활용은 적절한가?

### 기본기준

대학 내부 또는 외부 감사제도가 있고, 그 활용이 적절하다.

### 보고서 기술내용

대학을 합리적으로 운영하기 위한 내·외부 감사제도가 있는지 기술하고, 감사제도의 절차와 시기, 감사결과의 활용실태, 감사에서 지적된 문제점을 개선하기 위한 노력 등은 어떠한지 구체적으로 기술한다.

#### 참고자료

비치 : 1. 감사결과서(시정 명령 등)
2. 감사 결과 활용이나 개선 실적

## 1-4 대학 발전 계획

**평가개요**

대학은 경쟁력 제고를 위한 실현 가능한 중·장기 발전계획을 가지고 있어야 하고, 동문 또는 지역사회 등이 대학 발전에 적극적으로 참여할 수 있도록 하여야 한다.

### 1-4-1 대학의 발전 계획이 수립되어 있으며, 대학본부 또는 학교법인의 지원은 적절한가?

**기본기준**

대학 발전 계획이 기술되어 있고, 대학본부 또는 학교법인의 지원은 적절하다.

**보고서 기술내용**

대학 발전계획 내용을 소개하고, 발전계획을 추진하기 위한 대학, 대학본부, 학교법인 등의 지원 노력과 예산조달 내용 등을 기술한다. 또한 다양한 대학 구성원들이 의사결정과정에 참여한 실적과 구성원들에게 발전계획을 알리기 위한 노력 등을 기술한다.

**참고자료**

비치 : 1. 대학 발전 계획서
2. 대학 발전 추진위원회 회의록
3. 발전계획 추진 관련 예산집행내역
4. 대학본부의 지원 실적과 관련 예산
5. 학교법인의 지원 실적과 관련 예산

## 1-4-2 대학 발전기금이 있고, 그 운용은 적절한가?

### 기본기준

대학 발전기금이 있고, 그 운용은 적절하다.

### 보고서 기술내용

대학 발전기금의 규모, 모금활동 내용, 앞으로의 모금계획을 기술하고, 모금된 기금을 대학발전에 사용한 실적과 향후 집행 계획 등에 대하여 기술한다.

[주] 종합대학 내 대학 발전기금으로 지정된 기금(지정기탁)이 있으면, 발전기금을 관리, 운영하기 위한 위원회가 대학에 구성되어 있어야 하고, 동 위원회에서 발전기금의 집행 계획을 수립하고 관리하여야 한다.

#### 참고자료

비치 : 1. 발전기금 관리위원회 회의록
2. 발전기금 집행내역 관련 자료와 집행계획
3. 확보된 발전기금 확인 자료(잔고증빙자료 등)

## 1-4-3 대학 발전을 위한 동문 또는 지역사회의 참여가 있고, 이들의 적극적인 참여를 유도하기 위한 대학의 노력은 적절한가?

### 기본기준

대학 발전을 위한 동문 또는 지역사회의 참여가 있고, 이들의 적극적인 참여를 유도하기 위한 대학의 노력이 적절하다.

### 보고서 기술내용

대학 발전기금 기부금 납부 이외에 대학 자체 특별기구를 통하여 동문 또는 지역사회가 참여한 구체적 활동이나 실적 등을 기술한다. 또한 동문과 지역사회가 대학 발전에 적극적으로 참여할 수 있도록 대학이 어떤 노력을 하였는지에 대하여 기술한다.

#### 참고자료

비치 : 1. 대학 발전 특별 기구 운영실적
2. 동문과 지역사회가 참여한 활동 실적 보고서
3. 동문과 지역사회의 참여 범위나 권한 등에 관한 규정

## 1-5 개선노력

**평가개요**

대학은 지속적인 질 관리를 위한 기구를 상설 운영하여 지난 주기 평가결과를 대학운영에 반영하고, 의학 교육의 질 향상과 국제화를 위해 지속적으로 노력하여야 한다.

### 1-5-1 대학은 질 관리와 개선을 위해 자체평가업무를 수행하는 상설기구가 있으며, 이를 적절하게 운영하고 있는가?

**기본기준**

대학의 지속적인 질 관리와 개선을 위해 자체평가 업무를 수행하는 상설기구가 있고, 운영 예산이 적절하다.

**보고서 기술내용**

대학의 질 관리와 개선을 위하여 규정에 의해 상설화된 기구 또는 위원회 성격의 연구기구(예, 자체평가위원회, 질 관리위원회, 대학발전연구위원회 등)가 있는지와 그 기구의 규정, 구성, 역할, 권한, 예산과 행정지원 현황을 기술한다.

[주] 대학의 질 관리와 개선을 위한 상설기구는 대학 경영목표의 달성 정도를 평가하고, 정기적으로 평가보고서를 발행하며 그 결과를 대학운영에 반영하고 있어야 한다. 또한 위원들의 전문성 확보를 위한 제도적인 장치나 구체적인 노력이 있어야 한다.

[주] 대학의 질 관리와 개선을 위한 상설기구는 최소한 회의비, 워크숍, 외부평가 자문비, 정책개발 연구비, 프로그램 개발비, 연구위원 활동비, 보고서 작성비 등을 지원하는 예산이 구체적으로 편성되어 있어야 하고, 집행되어야 한다.

**우수기준**

관련위원회의 연간 예산이 충분하며, 위원들의 전문성을 확보하기 위한 지속적인 노력이 있다.

**참고자료**

비치 : 1. 관련 기구의 규정
2. 관련 기구의 예산과 집행내역
3. 정기적인 검토 내용을 포함하는 관련 서류
4. 수정되고 실제 개선된 사항을 기록한 서류
5. 2년마다 실시된 자체 평가 자료

## 1-5-2 대학은 평가결과를 대학운영에 반영하여 지속적인 개선 노력을 하고 있는가?

### 기본기준

평가인증 결과를 대학운영에 반영하여 지속적으로 개선한 실적이 있다.

### 보고서 기술내용

최종평가보고서의 개선 요구 사항을 영역별로 기술하고, 각 요구 사항이 어떤 절차를 통해 어떻게 대학 운영에 어떻게 반영되었는지 기술한다. 평가결과를 구성원 모두가 공유하기 위한 대학의 구체적인 노력과 함께 영역별 미비점을 개선하기 위해 단기, 중·장기 개선 계획을 수립하여 실천되고 있는지와 구체적인 개선 실적을 기술한다.

[주] 각 영역별 개선 요구사항에 대한 단기, 중·장기 개선 계획, 평가인증 이후의 경과 기간, 대학의 지속적인 개선 노력 등을 종합적으로 고려하여 평가하되, 구체적인 개선 실적이 있어야 한다.

#### 참고자료

비치 : 1. 이전 평가인증 최종보고서
2. 보고서 내용에 따른 개선 실적

## 1-5-3 외국 의과대학과의 교류는 적절한가?

### 기본기준

국제협력을 위한 전담 기구가 있고 예산과 지원인력이 있다. 국외 연구, 교육, 실습 등의 학생 교류는 적절히 운영되고 있다.

### 보고서 기술내용

대학은 연구와 교육의 국제화를 위해 외국 의과대학이나 관련 기관과의 교류가 이루어지고 있는 현황과 집행 예산을 기술한다.

[주] 실적보고서는 상호방문에 관한 서신교환, 교환학생 평가보고서 등의 증빙서류

[주] 예산집행에는 학생 자부담이나 학교 측의 지원 증빙서류

### 우수기준

외국대학에서 학점취득이 가능하며, 이에 대한 구체적인 실적이 있다.

#### 참고자료

비치 : 1. 국제협력 전담 기구 회의록
2. 국제화 실적 예산집행 내역
3. 외국 의과대학에서 취득한 학점 인정 근거와 운영 실적

의학교육 평가인증 기준

# 02 기본의학교육과정

## 2-1 교육과정 개요

**평가개요**

대학은 교육이념 및 특성이 반영된 교육목적, 교육목표, 졸업성과를 가지고 있어야 하며, 이를 교육과정에 구체적으로 반영하여야 한다. 대학은 자체 개발한 교육과정 개발 원칙을 가지고 있어야 하며, 이를 지속적으로 평가하여야 한다.

### 2-1-1 교육이념 및 특성이 반영된 교육목적과 교육목표를 가지고 있는가?

**기본 기준**

교육목적과 교육목표가 기술되어 있고, 교육목표에 대하여 주기적인 평가를 한다.

**보고서 기술내용**

교육목적과 교육목표를 기술하고 그러한 교육목표를 설정하게 된 근거와 개발 절차 및 배경 등을 기술한다. 교육목적과 교육목표는 구성원의 합의 또는 관련 위원회의 조직적인 노력을 거쳐 합리적으로 만들어야 하고, 대학의 교육이념, 특성화, 대한민국 의사의 역할과 덕목, 보건의료환경 변화 요구 등을 적절히 반영하여야 한다. 또한 교육목표에 대하여 주기적인 평가를 하고 있는지 기술한다.

## 2-1-2 교육목표에 바탕을 둔 졸업성과를 가지고 있는가?

### 기본기준

대학은 교육목표에 바탕을 둔 구체적인 졸업성과를 가지고 있고, 졸업성과의 개선을 위해 정기적인 평가를 한다.

### 보고서 기술내용

교육목표에 바탕을 둔 구체적인 졸업성과를 기술하고, 이것이 어떠한 절차를 거쳐 개발되었는지 자세히 기술한다. 또한 졸업성과의 개선을 위해 정기적인 평가를 시행하고 있는지 여부를 기술한다.

[주] 졸업성과는 2014년 대한의사협회에서 발표한 ‘2014 한국의 의사상’을 참조한다.

## 2-1-3 교육과정은 교육목표와 졸업성과를 달성하기 위하여 적절한가?

### 기본기준

대학은 자체 개발한 교육과정의 원칙에 입각하여 교육과정을 운영하고, 그 교육과정에는 교육목표와 졸업성과가 반영되어 있다. 교육과정의 원칙은 지속적으로 평가되고 있다.

### 보고서 기술내용

대학이 자체 개발한 원칙을 바탕으로 하여 교육과정의 큰 틀을 기술하고, 교육과정에 교육목표와 졸업성과가 어떻게 반영되고 있는지를 항목별로 구분하여 구체적으로 기술한다. 교육과정의 원칙이 지속적으로 평가 및 검토되고 있는지를 기술한다.

[주] 원리와 원칙에는 교육과정의 기본방향/전략, 교육과정 설계원칙, 교육내용 편성원칙, 교수학습법, 교육평가, 교육지원체제 등의 주요 내용을 요약하여 기술한다.

#### 참고자료

비치 : 1. 교육과정 원칙 개발 관련 회의 자료

## 2-2 교육과정 개발과 지원

**평가개요**

대학은 교육목표를 구성원에게 인지시켜야 하며, 교육목표 및 졸업성과를 달성하기 위하여 교육과정 개선과 관리를 담당하는 기구를 설치하고 이 기구에 권한과 예산을 배정하여야 한다. 대학은 교육과정의 충실한 시행을 위해 다양한 교육방법을 개발하여 적용하고 교육에 직접 필요한 예산을 확보하여야 한다.

### 2-2-1 교육목표는 구성원들에게 잘 인지되어 있으며, 대학은 교육목표 인지도를 높이기 위해 적절한 노력을 하고 있는가?

**기본기준**

교육목표에 대해 대학 구성원이 잘 인지하고 있으며, 대학은 이를 높이기 위해 다양한 노력을 하고 있다. 교육목표에 대한 대학 구성원의 인지도 조사를 주기적으로 시행하고 있다.

**보고서 기술내용**

교육목표가 구성원들에게 잘 인지되어 있는지를 구체적인 설문지 항목과 함께 기술하고, 교육목표를 알리기 위한 대학의 노력(교수세미나, 연수회, 설문조사 등)을 구체적으로 기술한다.

[주] 교육목표를 구성원에게 인지시키기 위한 대학의 노력에는 공지, 세미나, 연수, 강의실 및 실습실 게시 등이 있다. 교육목표 인지도를 조사하기 위한 설문지 항목이 적절한지 기술하고, 또한 주기적으로 시행한 설문 결과 등에 대해서 기술한다.

**참고자료**

부록 : 1. 교육목표 조사 설문지
비치 : 1. 교육목표 홍보물

## 2-2-2 교육과정 개선과 관리를 담당하는 교육관련 위원회의 활동은 적절한가?

### 기본기준

교육과정 개선과 관리를 담당하는 위원회의 구성, 역할, 권한 및 책임 등이 분명하게 설정되어 있다. 위원회는 독립적으로 구성되어 있고, 위원회 활동의 연속성을 위한 제도적인 장치가 마련되어 있다.

### 보고서 기술내용

대학의 교육과정 개선과 관리를 담당하는 교육관련 위원회의 현황, 역할, 권한 및 책임 등을 기술한다. 교육과정위원회의 구성 과정을 기술하고 위원회 활동의 연속성을 위한 제도적인 장치가 무엇인지 기술한다. 그리고 교육과정위원회의 구체적인 활동 사항을 기술한다.

[주] 교육과정의 개선과 관리를 담당하는 위원회의 ① 교수, 학생, 행정직원의 참여 정도 ② 교육과정의 개발과 평가를 위한 교육전문가의 참여 여부 ③ 교실(학과)의 이해관계 및 정치적 영향과는 무관하게 교육과정을 관리 운영할 수 있는 대학 내규 등이 있는지에 대해서도 기술한다.

#### 참고자료

부록 : 1. 교육과정 운영 규정
비치 : 1. 교육과정 운영 시행 세칙
2. 교육과정 위원회 회의록

## 2-2-3 교육과정위원회는 교육과정 개발, 관리 및 평가 등을 위한 예산을 확보하고 적절하게 집행하고 있는가?

### 기본기준

교육과정위원회의 연간 예산이 적절하다.

### 보고서 기술내용

대학의 교육과정 개발, 관리 및 평가를 담당하는 교육과정위원회가 교육과정 개선을 위해 사용한 최근 2년간의 예산과 집행 내용을 항목별로 구분하여 구체적으로 기술한다.

[주] 교육과정위원회라 함은 교육과정을 직접적으로 개발, 관리 및 평가하는 교육관련 위원회를 의미하며 대학마다 명칭은 달리할 수 있다.

[주] 교육과정위원회의 예산에는 각종 교육 관련 위원회의 예산을 모두 포함하여 계산할 수 있다. 그러나 각 위원회에서 집행한 실제 교육비용(OSCE, PBL 등의 직접 비용), 의학교육학과(교실, 실 등), 학생지도위원회 등의 예산은 포함하지 않는다. 즉, 교육 관련 위원회의 운영을 위한 예산(회의비, 세미나비, 연구개발비, 워크숍비 등)을 의미한다.

### 우수기준

교육과정위원회가 정기적으로 개최되고, 충분한 예산을 확보하고 적절하게 집행하고 있다.

**참고자료**

| |
|---|
| 비치 : 1. 교육과정위원회 예산 집행 내역 |

## 2-2-4 효과적인 교육과정을 운영하기 위해 다양한 교육방법을 개발하여 적용하고 있는가?

### 기본기준

수업 성과와 수업방법이 반영된 수업계획서가 있고, 전체 수업의 5% 이상이 다양한 교육방법으로 진행된다.

### 보고서 기술내용

임상실습 과정을 제외한 전체 교육시간을 산정하고, 각각의 교육방법이 차지하는 비율을 기술하되 교육과정의 원칙에 따른 교육방법에 대하여 기술한다.

[주] 다양한 교육방법은 전통적 강의방식과 실험실습을 제외한 PBL, TBL 등을 말한다.

### 우수기준

수업계획서가 학생들에게 미리 공지되고, 전체 수업의 20% 이상이 다양한 교육방법으로 진행된다.

#### 참고자료

비치 : 1. 수업계획서

## 2-2-5 학생교육 관련 직접비용에 대한 지원은 적절한가?

### 기본기준

연간 학생 1인당 등록금 대비 학생교육 관련 직접비용이 적절하다.

### 보고서 기술내용

학생교육에 필요한 직접비용의 예산과 집행내용을 항목별로 기술한다.

[주] 학생교육 관련 직접경비에는 기초의학 실험실습비, 표본제작, CPX와 OSCE 관련 비용, 교재비, 학생 교육용 컴퓨터프로그램, 표준화환자 인건비, 시험문항 개발비, 임상술기실습실 소모품, PBL과 TBL 운영비, 임상실습 관련 비용 등을 포함한다.

### 우수기준

연간 학생 1인당 등록금 대비 학생교육 관련 직접비용이 우수하다.

#### 참고자료

비치 : 1. 학생교육 관련 직접비용 집행 내역

## 2-3 교육과정 구성과 운영

**평가개요**

대학은 교육목표와 졸업성과를 달성하기에 적절한 기초의학, 임상의학, 의료인문학 교육과정을 개발하고, 통합을 위해 노력하여야 한다. 그리고 교육과정의 효율적 운영을 위한 책임교수제도를 도입하고 적극 지원하여야 한다. 기초의학, 임상의학, 의료인문학 교육과정별 학습성과를 개발하고 지속적으로 평가하여야 한다.

### 2-3-1 교육목표와 졸업성과를 달성하기 위한 기초의학, 임상의학, 의료인문학 교육과정의 구성은 적절한가?

**기 본 기 준**

대학은 교육목표와 졸업성과를 달성할 수 있는 교육과정을 운영하고 있으며, 구체적인 운영지침이 있다.

**보고서 기술내용**

교육목표와 졸업성과를 달성하기 위한 교육과정이 어떻게 구성되어 있는지 기초의학, 임상의학, 의료인문학 교육과정을 중심으로 실습 과정을 포함하여 각각 기술한다. 교육과정이 통합교육으로 이루어지고 있다면 각각의 과정이 통합교육과정 안에 어떻게 반영되어 있는지를 기술한다. 교육과정의 운영지침을 구체적으로 기술한다.

[주] 의료인문학의 분야는 어문학, 사학, 철학, 윤리학, 사회학, 법학, 경영학, 인류학, 심리학, 예술 등의 전공을 의미한다.

[주] 통합의학, 보완대체의학은 의료인문학 분야에 함께 기술한다.

[주] 교육과정 운영지침은 교육과정 계획, 실행, 분석/평가, 피드백, 개선 등 운영에 관한 명문화된 지침을 의미한다.

**참고자료**

부록 : 1. 기초의학, 임상의학, 의료인문학 교육과정 구성 현황표

비치 : 1. 기술되어 있는 교육과정 운영지침

## 2-3-2 졸업성과에 바탕을 둔 시기성과가 개발되어 있는가?

### 기본기준

대학은 시기성과를 개발하고, 이 성과는 대학의 졸업성과를 반영하고 있다.

### 보고서 기술내용

시기성과를 구체적으로 기술한다. 시기성과가 대학의 졸업성과와 어떻게 연계되어 있는지 기술한다.

**참고자료**

| |
|---|
| 비치 : 1. 시기성과가 포함된 교육과정 개요 또는 지침 |

## 2-3-3 기초의학, 임상의학, 의료인문학 교육과정의 통합은 적절한가?

### 기본기준

기초의학, 임상의학, 의료인문학 교육과정의 통합을 위한 교육과정을 운영하고 있다.

### 보고서 기술내용

기초의학, 임상의학, 의료인문학 교육과정은 서로 어떻게 연계되어 있는지 기술한다. 각각의 교육과정을 통합하기 위한 대학 차원의 노력과 지원체계를 기술한다.

#### 참고자료

비치 : 1. 통합교육과정 수업계획서

## 2-3-4 통합교육과정의 책임교수제도가 있으며, 권한과 책임은 적절한가?

### 기본기준

통합교육과정 운영을 위한 책임교수제도가 있으며, 책임교수의 권한과 책임에 대한 규정이 있다.

### 보고서 기술내용

통합교육과정을 위한 책임교수제도를 기술하고, 책임교수의 권한과 책임은 무엇인지 구체적으로 기술한다.

[주] 통합교육과정은 여러 과목을 통합한 형태로 묶은 단위과정(예, 인체의 정상구조, 혈액 및 종양, 성장과 노화 등)을 의미하며, 각각의 단위과정을 운영하는 책임교수가 별도로 지정되어 있는지를 의미한다.

#### 참고자료

부록 : 1. 책임교수의 권한과 책임에 관한 규정

## 2-3-5 교육과정의 과정성과와 수업 성과가 설정되어 있고, 수업에 반영되고 있는가?

**기본기준**

과정별로 구체적인 성과가 있고, 이 성과는 수업에 반영되어 있다.

**보고서 기술내용**

과정성과를 구체적으로 기술하고, 수업에 어떻게 반영되고 있는지 기술한다.

**참고자료**

| |
|---|
| 비치 : 1. 성과가 반영된 과정별 수업계획서 |

## 2-3-6 교육목표와 졸업성과를 달성하기 위한 기초의학 교육내용은 적절한가?

### 기본기준

기초의학 교육내용은 대학의 교육목표와 졸업성과를 달성하기에 적합하게 구성되어 있다.

### 보고서 기술내용

기초의학 교육과정은 세계의학교육협회가 강조하는 ① 신체의 구조와 기능에 관한 분자생물(molecular), 세포(cellular), 기관(organ) 및 신체전부(whole body)수준의 내용 ② 비정상적(병적)인 신체구조와 기능에 관한 내용 ③ 질병의 자연사, 병적 상태에 대한 신체 방어기전 및 질병에 대한 신체반응 등에 관한 내용 ④ 질병발생과 관련되는 유전적, 환경적, 사회경제적 요인에 관한 내용 및 연구방법 등의 내용을 포함하고 있는지 기술한다. 또한 기초의학실습은 어떤 내용으로 실시되는지 기술하며, 기초의학 교육의 실행을 위한 자원이 무엇인지 기술한다.

#### 참고자료

비치 : 1. 기초의학 교육관련 과정별 수업계획서

## 2-3-7 교육목표와 졸업성과를 달성하기 위한 임상의학 교육내용은 적절한가?

### 기본기준

임상의학 교육내용은 대학의 교육목표와 졸업성과를 달성하기에 적합하도록 구성되어 있다.

### 보고서 기술내용

임상의학 교육내용은 교육목표와 졸업성과를 달성하기 위해 어떻게 구성되어 있는지 기술한다.

**참고자료**

비치 : 1. 임상의학 교육관련 과정별 수업계획서

## 2-3-8 임상실습에 대한 준비교육이 있는가?

### 기본기준

환자 면담 기법과 기본 술기에 대한 교육이 포함된 임상실습 전 준비교육이 있다. 이러한 과정이 블록(block)으로 개설된 경우에는 최소 4주 이상, 지속적인 과정(longitudinal course)이면 최소 2학기 이상 개설되어 있다.

### 보고서 기술내용

학생이 임상실습에 진입하기 전에 임상실습 준비 교육을 언제, 어떻게 실시하고 있는지 기술한다. 임상실습 준비교육 내용에 환자 면담 기법과 기본 술기에 대한 교육이 포함되었는지를 기술한다.

[주] 임상실습 준비교육은 ICM(Introduction to Clinical Medicine), FCM(Fundamentals of Clinical Medicine) 등을 의미한다. 블록 또는 지속적인 과정이 있으면, 학생 1인당 교육받는 시간이 40시간 이상 되어야 한다.

#### 참고자료

비치 : 1. 임상실습 준비교육 관련 자료

## 2-3-9 임상실습 지침서가 학생들에게 제공되고 있으며, 실제로 활용되고 있는가?

### 기본기준

학생이 반드시 습득하여야 하는 기본 술기가 포함된 임상실습 지침서가 학생들에게 제공되고 있으며, 실제로 활용되고 있다.

### 보고서 기술내용

학생들에게 임상실습 지침서가 각 과별로 제작 및 배포되고 있는지를 기술한다. 학생들에게 반드시 필요한 기본 술기가 임상실습 지침서에 고르게 포함하고 있는지와 그 내용이 실제 실습에 어떻게 활용되고 있는지를 기술한다. 또한 학생이 기본적인 진료능력을 습득할 수 있도록 과별로 흔한 증상, 징후 및 질병(최소한의 필수 환자군)을 구체적으로 제시하고 있는지 기술하고, 임상실습 중 학생의 환자 진료 범위와 책임에 대한 규정이 있으면 기술한다.

[주] 기본 술기란 활력징후 측정, 정맥 채혈, 혈액도말 검사, 심전도, 정맥 및 근육 주사, 요로 도자, 관장, 환부 소독 및 봉합, 비위관 삽입 등과 같은 일차 진료 수준의 진단 술기, 치료 술기 및 환자안전 관련 술기를 말하며, 이는 대학에 따라서 차이가 있을 수 있다.

[주] 최소한의 필수환자군이란 일차 진료 수준의 능력을 갖추기 위해서 각 과별로 꼭 알아야 할 흔한 증상, 징후 또는 질병을 말한다.

### 우수기준

임상실습 지침서에는 기본 술기와 관찰 술기가 구분되어 기술되어 있으며, 실습 과마다 최소한의 필수환자군을 제시하여 학생이 경험할 수 있도록 하고 있다. 또한, 임상실습 중에 학생이 할 수 있는 술기와 진료 범위에 대한 자체 규정이 있다.

[주] 관찰 술기란 실습 중 1회 이상 관찰하여야 하는 술기로써, 이는 학교에 따라서 차이가 있을 수 있다(예, 복막 천자, 늑막 천자 및 생검, 골수 천자를 통한 흡인 및 생검, 척수천자, 감염 환자의 정맥 채혈법, 연조직 세침 흡인술, 심낭천자, 중심정맥 카테터 삽입술, 혈액투석 및 복막 투석, 상·하부 위장관 내시경, 기관지 내시경, 심폐소생술). 또한 학생들은 술기의 목적과 방법 및 위험도를 설명할 수 있어야 한다.

#### 참고자료

비치 : 1. 임상실습 지침서

## 2-3-10 임상실습은 학생이 의사의 역할을 습득할 수 있는 방법으로 실시되고 있는가?

### 기본기준

임상실습은 단순 관찰과 같은 수동적인 방법 이외에 의료진의 일부로 실제 진료에 참여하는 등 의사로서의 업무를 익힐 수 있도록 구성되어 있다.

### 보고서 기술내용

학생이 임상실습 과정에서 의사로서의 역할을 습득하기 위해 어떤 방법을 제공하고 있는지 기술한다. 예를 들면, 수술처치, 환자진찰, 초진환자면담, 모의처방, 의무기록 작성 등을 어떻게 학생들에게 수행하도록 하는지를 구체적으로 기술한다.

### 우수기준

학생 전원이 4주 이상 참여할 수 있는 학생 인턴제가 실시되고 있다.

[주] 학생인턴제는 ① 핵심과목(내과, 외과, 산부인과, 소아청소년과, 정신과, 응급의학과) 위주로 실시한다. ② 입원환자 중심의 실습(처방, 처치, 당직 등)이어야 한다. ③ 기존의 학생 임상실습과 차별화하여 운영되어야 한다.

#### 참고자료

| |
|---|
| 비치 : 1. 임상실습 지침서 |

## 2-3-11 임상실습이 충분한 기간 실시되고 있는가?

### 기본기준

최소 52주, 주당 36시간에 준하는 기간 동안 임상실습이 이루어지되, 내과, 외과, 산부인과, 소아청소년과, 정신건강의학과, 응급의학과 실습은 반드시 포함되어야 한다.

### 보고서 기술내용

임상실습 편성시기와 과목의 구성, 각각의 실습 시간을 기술하며, 임상실습 편성 원칙과 근거 자료에 대해서 기술한다.

### 우수기준

최소 72주, 주당 36시간에 준하는 임상실습이 이루어지고 있다.

## 2-3-12 임상실습을 위한 장소가 다양하며 외래 환자 중심 실습은 적절한가?

### 기본기준

임상실습은 상급종합병원 이외에 종합병원, 병·의원에서도 이루어지고, 핵심과의 외래 환자 중심 실습은 전체 실습 시간의 25% 이상이다.

### 보고서 기술내용

임상실습의 장소와 방법이 일차 진료 수준에 적절한지를 관련 자료와 함께 기술한다. 즉, 학생들의 임상실습 장소가 상급종합병원 이외에 종합병원, 병·의원이 포함되어야 하며, 핵심과에서 외래 및 입원 환자 중심 실습이 어떻게 이루어지고 있는지 기술한다.

[주] 핵심과는 내과, 외과, 산부인과, 소아청소년과, 정신건강의학과, 응급의학과를 의미하며, 응급의학과는 외래 실습으로 간주한다.

#### 참고자료

비치 : 1. 임상실습 지침서

## 2-3-13 학생이 자유롭게 선택할 수 있는 임상실습 과정이 있는가?

### 기본기준

학생이 자유롭게 선택할 수 있는 임상실습 과정이 적절히 편성되어 있다.

### 보고서 기술내용

임상실습 과정 중 학업성취가 부족한 부분을 보충하거나 더욱 심화시킬 목적으로 학생이 자유롭게 선택할 수 있는 임상실습 과정이 있는지에 대하여 기술한다.

[주] 학생이 자유롭게 선택한다는 것은 실습 장소에 구애받지 않고 학생이 희망하는 곳(기초, 임상, 국내외 연구기관 등)에서 실습을 할 수 있다는 것을 의미한다.

#### 참고자료

비치 : 1. 자유 선택 임상실습 현황표(개설 과정, 장소 등)

## 2-3-14 임상실습 책임교수가 지정되어 있고, 학생에 대한 지도감독과 피드백이 적절한가?

### 기본기준

임상실습 책임교수와 교육 담당 전공의가 과별로 임명되어 있고, 학생에 대한 지도 감독과 피드백을 하고 있다.

### 보고서 기술내용

모든 임상실습 장소(원외인 경우 임상 지도의, 또는 외래 교수)의 책임 교수 또는 교육 전담 전공의 명단을 기술하고, 학생에 대한 지도 감독과 피드백의 시기, 방법, 내용 및 횟수 등에 대하여 자세히 기술한다.

### 우수기준

교육 담당 전공의의 교육자로서 역할, 임무 등을 명시한 관련 규정이 있으며, 핵심과목 임상실습을 담당하는 교육전공의에 대해서는 학생 지도 감독과 피드백 방법에 관련한 교육과 훈련을 정기적으로 실시하고 있다.

[주] 대학부속병원 전공의는 피교육자인 동시에 학생교육에 참여하는 교육자와 평가자로서 역할을 수행하고 있다. 교육자로서 전공의(Resident as Teacher) 교육/훈련 프로그램에는 효과적인 임상실습지도술, 학생과의 대화술, 학생평가방법, 교육자 윤리 등을 포함한다.

#### 참고자료

비치 : 1. 각 과별 학생지도와 피드백 결과가 반영된 임상실습지침서
2. 교육자로서 전공의 관련 규정, 교육/훈련 실적

## 2-3-15 교육목표와 졸업성과를 달성하기 위한 의료인문학 교육과정은 적절한가?

### 기본기준

교육목표와 졸업성과를 달성하기 위한 의료인문학 교육과정을 전 학년에 걸쳐 운영하고 있다. 의료인문학 교육과정에 통합의학, 대체의학 등의 내용도 포함되어 있다.

### 보고서 기술내용

의료인문학 교육과정은 대학의 교육목표와 졸업성과를 달성하기 위해 어떻게 구성되어 있는지를 구체적으로 기술한다. 이 과정의 과목들이 졸업성과를 달성하기 위해 전 학년에 걸쳐 어떻게 반영되고 있는지를 기술한다. 이 과정에 통합의학, 대체의학 등도 함께 기술한다.

#### 참고자료

비치 : 1. 의료인문학 관련 수업자료

## 2-3-16 의료인문학 교육 관련 과정성과에 도달하기 위한 교육방법과 평가방법은 적절한가?

### 기본기준

의료인문학 교육과정에서 다양한 교육방법과 평가방법이 적용되고 있다.

### 보고서 기술내용

의료인문학 교육 관련 과정성과를 달성하기 위해 어떠한 교육방법 및 학습평가 방법을 적용하는지에 대해 기술한다. 특히 단순 강의식 수업 이외에 사용하는 다양한 교육방법에 대해 기술한다.

[주] 단순 강의식 이외의 방법이란 수업 시간의 50% 이상을 토론, 발표, 질의응답 등으로 진행하는 것을 의미하며, 나아가 소집단을 대상으로 한 팀바탕학습, 문제바탕학습, 토론식 수업 등을 말한다.

#### 참고자료

비치 : 1. 의료인문학 교육 관련 수업계획서
2. 교육방법 및 평가 관련 자료

## 2-4 학업성취도 평가

**평가개요**

대학은 학생의 학업성취도와 수행능력을 학습성과에 맞게 평가하고 학생에게 피드백 하여야 한다.

### 2-4-1 성과에 따라 학생들의 학업성취도를 평가하고 있으며, 그 결과를 학생들에게 피드백하고 있는가?

**기본기준**

과정별로 설정된 성과를 확인할 수 있는 평가방법을 적용하고 있으며, 전체 학생에게 1회 이상 피드백을 주는 과정이 전체 과정의 1/3 이상이다.

**보고서 기술내용**

과정별로 사전에 설정된 성과를 평가하기 위한 전략 또는 평가결과 등을 기술한다. 학업성취도에 대한 피드백은 어떠한 방법과 내용으로 제공되는지를 구체적으로 기술한다.

**우수기준**

전체 과정 중 10% 이상이 형성평가를 시행하고 있다.

[주] 피드백이란 단순히 학생들의 시험점수나 등수만을 제공하는 것이 아니라 시험문제 풀이, 학생의 현재 학업성취 도달 여부, 장점과 단점 등을 구체적으로 제공하는 것이다.

**참고자료**

비치 : 1. 학업성취도 평가관련 자료
2. 학업성취도에 대한 피드백 자료
3. 형성평가 실시 자료

## 2-4-2 임상실습 과정에서 학생의 수행능력은 적절하게 평가되고 있는가?

### 기본기준

임상수행능력평가를 1회 이상 실시하며, 그 결과를 임상실습 성적에 반영하고 있다.

### 보고서 기술내용

학생들의 임상수행능력을 평가하기 위해 CPX와 OSCE를 어떤 방법(학년, 사례 수 등)으로 시행하는지 구체적으로 기술한다. 또한 그 결과가 각각의 핵심과목(내과, 외과, 산부인과, 소아청소년과, 정신건강의학과, 응급의학과)에 어느 정도 비율로 반영되는지 기술한다.

[주] 임상수행능력평가는 CPX와 OSCE를 말한다.

### 우수기준

모든 핵심과목에서 해당 과목별로 실습기간 중 임상수행능력평가를 시행하고, 그 결과를 실습 성적에 반영하고 있다.

#### 참고자료

비치 : 1. 임상수행능력평가 자료
2. 평가결과를 성적에 반영한 자료

## 2-4-3 대학은 시기성과 또는 졸업성과에 바탕을 둔 학업성취도를 종합적으로 평가하고 있으며, 그 방법은 적절한가?

### 기본기준

시기성과 또는 졸업성과를 확인할 수 있는 종합평가를 시행하고 있다.

### 보고서 기술내용

시기성과 또는 졸업성과를 확인할 수 있는 종합평가를 어떠한 방법(시험시기, 횟수, 내용 등)으로 실시하는지 구체적으로 기술한다.

### 우수기준

시기성과와 졸업성과 종합평가를 모두 시행하고 있으며, 그 결과를 진급과 졸업 사정에 활용하고 있다.

**참고자료**

비치 : 1. 시기별 학업 성취도 평가 및 분석 자료

## 2-5 교육과정 평가와 개선

**평가개요**

대학은 전체 교육과정을 지속적으로 평가하고 그 결과를 교육과정 개선에 활용하여야 한다.

### 2-5-1 과정성과를 정기적으로 검토하여 교육과정 개선에 반영하고 있는가?

**기본기준**

과정성과의 적절성 여부, 수업과 평가에 반영하는 정도를 매년 검토하고 수업과 평가를 개선하거나 성과를 개선한 실적이 있다.

**보고서 기술내용**

과정성과를 검토한 연간 실적을 기술한다. 또한 검토한 결과를 해당 단위과정에 어떻게 개선하여 반영하였는지를 구체적으로 기술한다. 성과 내용의 변화가 있을 때에는 그 배경과 결과를 기술한다.

**참고자료**

비치 : 1. 교육과정 성과 검토관련 자료
2. 성과를 반영한 교육과정 개선자료

## 2-5-2 시기성과와 졸업성과가 교육과정에 반영되고 있는지를 검토하여 교육과정을 개선하고 있는가?

### 기본기준

시기성과와 졸업성과가 교육과정에 반영되고 있는지 검토하여 실제 교육과정 개선에 활용하고 있다.

### 보고서 기술내용

시기성과와 졸업성과가 교육과정에 적절하게 반영되고 있는지를 검토한 과정과 절차를 기술한다. 또한, 그 결과 교육과정이 개선된 실적을 기술한다.

[주] 시기성과와 졸업성과의 검토와 교육과정에의 반영 정도는 대학 차원의 교육과정 관련 위원회가 실행하는 것을 의미한다.

**참고자료**

비치 : 1. 시기(또는 학년별) 성과와 졸업성과의 검토 자료
2. 관련 위원회 회의록 등
3. 시기(또는 학년별) 성과와 졸업성과를 반영한 교육과정 개선 자료

## 2-5-3 대학은 교육과정 운영에 대한 전반적인 평가를 실시하고 있으며, 개선에 활용하고 있는가?

### 기본기준

전체 교육과정이 적절한지에 대한 평가를 연 1회 실시하고 있으며, 그 결과를 개선에 활용한 실적이 있다.

### 보고서 기술내용

전체 교육과정을 어떠한 방법으로 평가하고 있는지 구체적으로 기술한다. 또한, 그 결과 교육과정이 개선된 실적을 기술한다.

[주] 교육과정 평가란 단순 학업성취도 평가 결과만을 의미하는 것은 아니며, 학생과 교수의 의견 수렴, 학생의 강좌평가, 전문가 면담, 외부 평가 결과 등이 포함된다.

#### 참고자료

비치 : 1. 교육과정 평가 자료
2. 관련 위원회 회의록 등

의학교육 평가인증 기준

# 03 학생

## 3-1 입학정책과 학생선발

**평가개요**

대학은 교육이념과 사회적 책무성에 기초한 입학정책을 갖고 있어야 하며, 학생선발방법과 기준이 합리적이고 절차는 공정해야 한다.

### 3-1-1 대학은 학생선발방법을 명시한 입학정책이 있는가?

**기본기준**

대학의 교육이념과 사회적 책무성에 맞는 다양한 학생선발방법을 명시한 입학정책이 있고 이를 홍보하고 있다.

**보고서 기술내용**

대학의 입학정책과 학생선발제도 및 홍보에 대해 기술한다. 쉽게 이해할 수 있는 학생선발기준의 공개 여부 등을 포함하여 기술한다. 편입생을 선발할 경우 입학 및 공동 학위 프로그램의 선수과목 등의 입학 요구사항을 기술한다.

**참고자료**

비치 : 1. 학생선발 제도 관련 자료
2. 대학 편람과 입학안내 책자 또는 관련 정보가 수록된 웹 사이트의 인쇄본

## 3-1-2 대학은 학생선발제도의 개발과 개선을 위한 노력을 하고 있는가?

### 기본기준

대학은 학생 선발의 기준과 절차를 개발하고 개선하기 위한 노력을 하고 있다. 편입생을 선발할 경우 의과대학의 학생선발 관련 위원회가 전문적이고 지속적으로 운영되어야 한다.

### 보고서 기술내용

입학정책과 학생선발제도의 적절성을 정기적으로 평가하고 있으며, 그 결과를 기초로 한 개선 실적 및 계획을 기술한다. 또한 선발기준이 교육목표와 연계성이 있는가를 기술한다. 편입생을 선발할 경우 의과대학 학생선발 관련 위원회의 구성과 역할을 기술한다. 위원회의 학생 선발 관련 교육과 운영방법을 기술한다.

[주] 입학정책과 학생선발제도의 적절성 평가는 학생선발기준 및 절차에 대한 평가와 입학한 학생들의 학업성취도 등에 대한 평가에 기초한다. 개선 실적 및 계획에는 입학정책 관련 기구 및 위원회 등의 활동을 기술한다. 편입생을 선발할 경우 학생선발 관련 위원회는 교내·외전문가로 구성되어 있어야 한다.

#### 참고자료

부록 : 1. 학생선발 관련 위원회의 규정과 위원 일람표(편입생 포함)
2. 위원회 외부 인원 명단과 그 역할 일람표(편입생 포함)

비치 : 1. 최근 2년간 입학제도에 대한 교내·외 감사자료
2. 최근 2년간 학생선발 관련 위원회 규정과 위원회 활동 실적
3. 최근 2년간 입학제도에 대한 교내·외 평가자료

## 3-1-3 대학의 학생선발 방법, 절차와 기준은 적절한가?

### 기본기준

대학의 학생선발 방법, 절차와 기준이 합리적이고 공정하다.

### 보고서 기술내용

대학의 학생선발 방법, 절차, 기준이 합리적이고 공정한지를 기술한다. 입학 지원서 접수, 면접, 합격 통보 등을 포함한 전반적인 학생 선발 과정을 기술한다.

#### 참고자료

부록 : 1. 학생선발 과정에 사용되는 서류 양식
비치 : 1. 최근 2년간의 학생선발 관련 서류

## 3-1-4 대학은 의사가 되는데 필요한 인성을 평가하는 학생선발 방안이 있는가?

### 기본기준

대학은 의사가 되는데 필요한 인성을 평가하는 학생선발 방안이 있다.

### 보고서 기술내용

지원자의 인성평가방법을 기술한다. 편입생을 선발할 경우 대학 입학에 필요한 최소 필수 과정이나 과목을 모두 기술한다.

### 우수기준

의사가 되는데 필요한 인성을 가진 학생을 선발하기 위한 체계적이고 심층적인 면접을 실시하고 있다.

[주] 심층면접은 전체 면접시간이 1인당 1시간 이상이다.

**참고자료**

비치 : 1. 입학 안내 책자(편입생 포함)
2. 면접시험에 사용되는 양식(편입생 포함)

현지확인 : 1. 면접 담당 교수 면담
2. 학생면담

## 3-2 학생 지도 체제

**평가개요**

대학은 학생들의 학습, 생활, 진로의 3가지 분야를 위한 학생 지도 체제를 갖추고 이를 활발히 운영해야 하며, 학생들이 교내·외 전문 활동을 할 수 있도록 관심을 갖고 적절히 지도해야 한다.

### 3-2-1 대학은 적절한 학생 지도 체제를 갖추고 있으며, 그 체제와 운영이 전문적으로 이루어지고 있는가?

**기본기준**

학생지도위원회를 구성하여 운영하고 있고, 학생지도 분야를 학습, 생활, 진로지도로 나누어 전문적이고 통합적으로 관리하고 있다.

**보고서 기술내용**

의과대학의 학생 지도 체제를 학습, 생활, 진로지도 부분으로 나누어 기술하고 이를 총괄하는 체제로 학생지도위원회가 구성되어 활발하게 운영되고 있는지를 기술한다. 의과대학 전체 학생을 대상으로 하여 학년별, 그룹별, 개인별 지도체계가 있는지와 정기적으로 지도교수와 학생이 만나는 체제가 확립되어 있는지 기술한다.

**우수기준**

의과대학 학생의 원활한 학교생활을 위하여 전체학생, 학년별, 그룹별, 개인별 지도를 위한 지도 체제가 확립(예: 학생 지도위원회 위원장 등의 역할이 명확하게 규정되어 있고, 학생들에게 공지되고 이해하고 있는가?)되어 있고, 운영이 전문적으로 이루어지고 있다.

**참고자료**

부록 : 1. 학생 지도 체제 관련 자료
2. 학생 전체, 학년별, 그룹별, 개인별 지도교수 현황 관련 자료
비치 : 1. 학생 지도교수 제도 관련 서류 (상담일지 포함)
2. 학생지도위원회 보고서
현지확인 : 1. 일부 해당 교수와 학생 면담

## 3-2-2 학생들의 학습 지도 내용은 적절한가?

### 기본기준

대학은 학생들이 자율적으로 상담을 받을 수 있는 체계를 갖추고 있다. 정기적으로 학생들의 학업성취 정도를 확인하고, 학습부진 학생들을 위한 프로그램이 있다.

### 보고서 기술내용

학생들이 자율적으로 성적이나 유급 결정자가 아닌 교수로부터 학업 상담을 받을 수 있도록 개발한 제도적 장치를 기술한다. 학생들의 학습 지도 내용을 기술한다. 특히 학습 지도는 의과대학이 유급생과 휴학생이 많다는 특수 상황을 고려하여 학습부진 학생에 대한 지도 과정 및 지도 실적을 기술한다.

### 우수기준

학습부진 학생에 대한 구체적인 지도 실적이 있고, 학년별 유급학생 비율에 급격한 변동이 있는 경우 문제점을 분석하는 등의 노력이 있다. 또한 유급학생을 구제하기 위한 방안이 마련되어 있다.

**참고자료**

부록 : 1. 학업상담 체제
　　　2. 연도별 학생 유급 현황
비치 : 1. 학업부진 학생 지도 자료
　　　2. 유급학생 비율 및 문제점 분석 자료
현지확인 : 1. 학업상담교수 포함 일부 교수 및 학생면담을 통한 학습지도 내용과 운영 확인

## 3-2-3 대학은 학생의 학습 평가, 유급, 졸업 사정과 징계 조치 등을 학칙에 따라 적절하게 시행하고 있는가?

### 기본기준

대학은 학생의 학습 평가, 유급, 졸업 사정과 징계 조치 등에 관한 학칙을 가지고 있고, 이를 적절하게 시행하고 있다.

### 보고서 기술내용

대학에서 시행 중인 학생의 학습 평가, 유급, 졸업 사정, 징계 조치 등의 학칙과 절차를 기술하고, 이를 학생과 교수에게 홍보하는 방법을 기술한다. 학생에게 유급이나 졸업 유예 등을 사전에 통보하고, 소명의 기회를 주고 있는지를 기술한다.

### 우수기준

학생은 학점이 확정되기 전 자신의 임상 실습 성적을 포함한 학습 성과 기록을 검토하고 성적, 유급, 졸업유예, 퇴학 등의 결정에 대한 소명의 기회가 보장되고, 실적이 있다.

[주] 학생의 공식적인 소명 절차가 보장되기 위해서는 학생 평가를 담당하지 않는 교수로 이루어진 관련 위원회가 있으며, 이를 학생들에게 공지하고 있어야 한다.

#### 참고자료

부록 : 1. 학생의 학습 평가, 유급, 졸업 사정과 징계 조치의 대학규정
2. 관련 위원회 규정
비치 : 1. 학생과 교수에 대한 홍보자료
2. 관련 위원회 최근 2년간 활동과 실적
현지확인 : 1. 위원회 위원 면담, 학생 면담

## 3-2-4 학생들의 생활 지도 내용은 적절한가?

### 기본기준

학생들의 생활 지도를 위해 교수와 학생 간에 정기적인 만남이 이루어지고 있으며, 그 결과를 기록하여 보존하고 있다.

### 보고서 기술내용

학생들의 생활 지도 내용을 기술한다. 생활 지도는 학생들의 주거, 학교생활, 교우관계 등을 분석하고 지도하기 위한 대학의 노력을 기술한다.

#### 참고자료

비치 : 1. 학생 생활 지도 실적 (상담일지 포함)
현지확인 : 1. 일부 교수 및 학생 면담

## 3-2-5 학생들의 진로 지도 내용은 적절한가?

### 기본기준

학생들의 진로 지도를 위한 프로그램과 실적이 있다.

### 보고서 기술내용

학생들의 교내·외 선택 과목과 졸업 후 진로(전공 선택, 병원과 전공의 선택 상담 등) 선택에 대한 학생 진로 지도 실적에 대해 기술한다. 학생지도를 수행하는 조언자(멘토) 관련 자료와 학생들에게 홍보되는 방법을 기술한다.

[주] 진로 지도 결과 졸업생의 진로 등에 대한 내용은 3-3-1의 졸업생의 진로와 관련된 평가문항에서 다룬다.

### 우수기준

학생의 교내·외 선택 과목 선택, 졸업 후 진로 지도와 자문 등의 진로 지도를 책임지고 수행하는 신뢰할 수 있는 조언자(멘토)와 조언자(멘토)양성 프로그램이 있다.

#### 참고자료

비치 : 1. 학생 진로 지도 프로그램과 운영실적(상담일지 포함)
2. 홍보자료
3. 최근 2년간 조언자(멘토)양성 프로그램 운영 실적

현지확인 : 1. 조언자와 학생 면담

## 3-2-6 대학은 학생들의 건전한 교내·외 전문 활동을 권장하고, 적절한 지도와 지원을 하고 있는가?

### 기본기준

대학은 학생들의 건전한 교내·외 전문 활동을 권장하고, 적절한 지도와 지원을 하고 있다.

### 보고서 기술내용

최근 2년간 학생들의 자발적인 학술, 연구, 사회봉사, 동아리 활동내용과 이들 활동에 참여하는 학생들의 규모를 기술하고(단, 대학이 계획하고 시행하는 행사는 제외), 이들 활동에 대한 대학의 지원내용을 기술한다. 또한 대학이 학생들의 전문 활동을 권장하는 과정에서 학생들과 연관된 문제에 대한 학생대표의 참여에 관련된 내용도 기술한다.

### 우수기준

교육과 학생 관련 위원회에 학생 대표단이 참여하고 있다.

#### 참고자료

부록 : 1. 학생 기초의학 및 임상연구 참여 관련 자료
2. 학생의 학술, 연구, 사회봉사, 동아리 발표 현황 관련 자료
3. 학생 활동 지원과 관련된 예·결산 자료
4. 학생 대표단이 참가하는 대학의 교육과 학생 관련 위원회 현황

비치 : 1. 학생의 학술, 연구, 사회봉사, 동아리 발표 현황 관련 자료
2. 학생활동 지원 실적
3. 최근 2년간 학생 대표단이 참가하는 대학의 교육과 학생 관련 위원회 활동 자료

현지확인 : 1. 의학과 학년별 학생 대표단(총대) 및 동아리 대표 약 2-3명 면담

# 3-3 학생 복지와 안전

**평가개요**

대학은 장학 제도를 갖추어 학생에게 경제적 도움을 주어야 하고 학생 복지를 위한 기숙사나 기타 복지 시설을 갖추어야 한다. 대학은 학생들이 임상 실습 시 노출되는 감염과 환경위험을 예방할 수 있는 대책이 있어야 하며, 질병 치료를 위해 쉽게 병원을 이용할 수 있도록 해야 한다. 대학은 학생들이 부당한 행위로 인한 피해를 받지 않도록 해야 한다.

## 3-3-1 대학은 공정하게 운영하는 장학제도가 있으며, 지급 비율은 적절한가?

**기본기준**

대학은 공정하게 운영하는 장학제도가 있으며, 등록금 대비 장학금 지급비율이 적절하다.

**보고서 기술내용**

대학의 장학제도와 장학생 선발 관련 규정, 기준의 엄정성, 선발기준의 다양성, 절차의 합리성, 선발 결과 등을 구체적으로 기술하고 최근 2년간 장학제도, 장학금 종류별 지급 인원 및 지급 금액에 대한 내용을 기술한다. 최근 2년간 등록금 전액 대비 교내외 장학금 총액의 비율은 얼마인지도 기술한다.

[주] 등록금 전액 대비 장학금 지급비율 = (학생 1인당 장학금/평균 등록금) × 100

[주] 동창회 기금을 이용한 대여 장학금은 포함. 그 이외의 대여 장학금은 제외(예, 국가장학금)

**우수기준**

등록금 전액 대비 장학금 지급비율이 우수하다.

**참고자료**

부록 : 1. 교내·외 장학 제도 및 내용
2. 학자금 융자 관련 체제

비치 : 1. 교내·외 장학회 운영 및 장학생 선발 관련 규정과 관련 회의록
2. 최근 2년간 장학금 지급 내역
3. 학자금 융자 지원 사례

현지확인 : 1. 학생 면담

## 3-3-2 대학은 학생들에 대해 적절한 재정지원 노력을 하고 있는가?

### 기본기준

필요 시 학생은 대학의 도움으로 학자금 융자를 받을 수 있다.

### 보고서 기술내용

학생이 대학과 연계되어 학자금 융자를 받을 수 있는 체제를 기술한다. 대학이 학생의 재정 관리 상담을 위해 운영 또는 개발 중인 체제가 있는지 기술하고, 이에 대한 실적을 기술한다.

### 우수기준

대학은 학자금 융자 외에 학생의 재정 관리 상담을 위한 체제와 실적이 있다.

**참고자료**

부록 : 1. 대학과 연계된 학자금 융자 체제
2. 학생의 재정 관리 상담 체제와 학자금 융자 이외 금융 지원 서비스
비치 : 1. 학자금 융자 사례
2. 학생의 재정 관리 상담 사례와 금융 서비스 지원 사례

## 3-3-3 학생들의 주거 현황을 파악하고 있으며 기숙사의 운영 실태는 적절한가?

### 기본기준

대학은 학생들의 주거 현황을 파악하고 있으며 기숙사의 운영 실태는 적절하다.

### 보고서 기술내용

재학생의 주거 현황을 기술한다. 기숙사가 있으면, 입실 현황, 기숙사의 수요 대비 공급 비율 및 타 지방 학생들을 위한 우선 배정 여부에 대하여 기술한다. 기본시설(냉난방, 채광, 욕실 등)과 편의시설(세탁실, 휴게실, 공동취사실 등)을 갖추고 있으며 위생적으로 관리되고 있는지, 시설 만족도 조사 결과와 의학과 학생들을 위한 배려(규정) 유무에 대해서도 기술한다. 그리고 현재 기숙사가 없으면 앞으로의 계획을 기술한다.

### 우수기준

희망자 전원을 수용할 수 있는 전용 기숙사가 있으며, 실습병원이 떨어져 있는 경우 임상실습 중인 학생을 수용할 수 있는 기숙사 시설을 실습 병원에서 제공하고 있다.

[주] 의대 전용 기숙사에는 독립가옥 또는 본부 기숙사의 일부라도 의대생이 사용하는 공간이 있는 경우도 포함.

#### 참고자료

부록 : 1. 학생 주거 및 기숙사 입실 현황 조사결과
2. 타 지방 학생의 기숙사 입실비율
비치 : 1. 기숙사 시설 및 관리 자료
2. 시설 만족도 조사 자료
현지확인 : 1. 기숙사 시설 확인 및 학생 면담

## 3-3-4 학생들의 건강관리를 담당하는 직원이나 체계가 있는가?

### 기본기준

학생 건강관리 규정과 시설이 대학 내에 있다. 학생들의 건강관리를 담당하는 직원이 보건소 또는 양호실에 있거나, 학생이 필요한 경우 병원을 쉽게 이용할 수 있는 체계가 있다.

### 보고서 기술내용

학생 개인의 건강관리를 위한 체계(보건소, 양호실, 병원 직접이용 등), 인력 현황 및 이용 실태를 기술한다. 학생이 원하는 경우 외부 의료기관에서 진료 받는 체계가 있으면 기술한다. 학생 건강관리 규정과 시설이 대학 내에 있는지와 최근 2년간 건강관리 자료를 기술한다. 학생들의 예방 접종 정책과 절차를 기술한다.

### 우수기준

학생들의 건강관리를 위해 입학, 의학과 진입 및 임상실습 진입 시에 각각 건강검진을 실시하고 있고, 학생이 원하는 경우 외부 의료기관을 이용할 수 있는 연계체계가 있다. 학생들은 우리나라 성인 예방접종 지침에 따른 예방접종이 시행된 지를 확인받은 후 임상 실습을 시작하고 있다.

#### 참고자료

부록 : 1. 건강관리 체계에 대한 규정
2. 건강관리 시설 인력 현황 및 최근 2년간 활동 업적 자료
3. 외부 의료기관 이용체계
4. 최근 2년간 예방접종 실적

비치 : 1. 최근 2년간 건강 검진 자료
2. 학생들의 예방접종 관련 서류와 실적

현지확인 : 1. 학생 면담
2. 건강관리 시설 및 외부 의료기관 연계체계 확인

## 3-3-5 학생들을 위한 상담 체계는 적절한가?

### 기본기준

학생을 위한 상담체계가 있으며, 심리검사를 실시하여 그 결과를 학생지도와 상담에 활용하고 있다.

### 보고서 기술내용

학생 상담을 위한 체계를 기술한다. 의학을 공부하면서 발생하는 정서적, 신체적 스트레스에 대해 학생들이 쉽게 적응할 수 있도록 도와주는 대학의 상담 프로그램을 기술한다. 학생이 필요로 할 때 정신과 또는 심리 치료를 외부 의료 전문가로부터 받는 체계가 있으면 기술한다.

### 우수기준

학생 상담실이 있고, 전임 상담원이 1인 이상 활동하고 있다. 또한 필요 시 학생이 외부 의료 전문가로부터 정신과 또는 심리 치료를 받을 수 있다.

[주] 전임 상담원이 활동한다는 것은 상담을 전담하는 전문 인력이 있다는 의미이다. 의과대학의 학생 담당 부서 직원, 학생담당 부학장 등이 지도하는 경우에는 전문적 운영으로 해석하지 않는다. 의과대학이 아닌 본교에 상담센터가 있는 경우에는 지리적으로 학생들의 접근이 용이하며, 의과대학 학생을 위한 별도의 전문 상담체제가 갖추어져 있고, 실적이 있으면 인정한다.

#### 참고자료

부록 : 1. 개인 상담 프로그램과 2년간의 실적
　　　2. 전임 상담원과 체계에 대한 규정
　　　3. 외부 의료전문가 이용체계
비치 : 1. 전문 상담 체제 관련 자료(전문상담 연구소 및 연구원)
　　　2. 심리검사 자료와 상담 활용 실적
　　　3. 전임 상담원의 상담 실적
현지확인 : 1. 상담 프로그램 담당 교수와 전임 상담원 면담
　　　　　2. 학생 면담

## 3-3-6 임상 실습 전 학생에게 감염과 환경 위험에 대한 예방과 대처 방법을 교육하고 있는가?

### 기본기준

학생을 위한 감염과 환경 위험에 대한 예방과 대처방법을 임상 실습 전 교육하고 있다.

### 보고서 기술내용

학생이 감염 및 환경 위험에의 노출을 예방할 수 있는 방법과, 노출되었을 때(예, 주사바늘 부상) 준수해야 할 절차를 언제 교육받는지 기술한다. 또한 학생이 감염 및 환경 위험에 노출된 경우 대학의 노출 후 관리 및 치료 대책과 필요한 재정 마련에 대해 기술한다.

### 우수기준

대학은 학생이 감염과 환경 위험에 노출된 경우 이에 대한 관리 및 치료 대책과 이에 필요한 재정을 마련하고 있다.

#### 참고자료

부록 : 1. 감염 및 환경 위험 예방과 대처 교육 실적
　　　2. 대학의 노출 후 관리 및 치료 대책 관련 규정
비치 : 1. 교육과정 일람표와 교육 자료
　　　2. 감염관련 지침과 예산 편성표
　　　3. 최근 2년간의 재정 집행 실적
현지확인 : 1. 학생 면담

## 3-3-7 대학은 학생들이 부당한 행위로 인한 피해를 받지 않도록 예방하고 보호하는 방안이 있는가?

### 기본기준

대학은 학생들이 학생, 교수, 기타 대학 구성원의 부당한 행위로 인한 피해를 받지 않도록 예방 교육과 홍보 활동을 하고 있으며, 피해 학생을 보호 및 구제하는 방안이 있다.

### 보고서 기술내용

학생들이 학생, 교수, 기타 대학 구성원의 부당한 행위(집단 따돌림, 언어폭력, 폭행, 희롱 등)로 인한 피해를 받지 않도록 시행하는 예방 교육과 홍보 활동을 실적과 함께 기술한다. 학생이 부당한 대우를 받은 경우, 이를 보고하는 경로와 조사 체계와 피해 학생을 보호 및 구제하는 방안을 기술 한다.

#### 참고자료

부록 : 1. 학생을 부당한 행위로부터 예방하기 위한 교육 내용, 교육 및 홍보 실적
2. 부당한 행위 발생 시 이를 보고하는 경로와 조사 체계, 피해 학생 보호 및 구제 방안

비치 : 1. 최근 2년간 부당한 행위 사례 및 처리 자료

현지확인 : 1. 일부 학생, 교수, 교직원 면담

## 3-4 졸업 후 진로

**평가개요**

대학은 학생들이 임상의학뿐만 아니라 이외 분야에 진출할 수 있도록 적극 지원해야 하고, 의사국가고시 시험에 합격할 수 있도록 해야 한다.

### 3-4-1 임상의학 이외 분야의 진출을 위한 대학의 지원은 적절한가?

**기본기준**

임상의학 이외 분야의 전문가를 확보하기 위한 적절한 정책(장학금 및 특별지원금 등)이 있거나, 졸업생이 임상의학 이외로 진출한 실적이 적절하다.

[주] 특별지원금은 생활비, 학회 참여 지원비, 기타 학업관련 비용을 의미하고 기초의학자 양성 정책을 포함한다.

**보고서 기술내용**

지난 10년간 임상의학 이외의 분야로 진출한 졸업생 현황과 대학의 노력을 기술한다. 임상의학 이외의 분야라 함은 대한의학회에서 정하고 있는 임상의학 분야의 전공과목을 제외한 것이다.

**우수기준**

임상의학 이외 분야로 진출한 경우의 실적이 우수하다.

[주] 군복무, 대체복무자는 제외하고, 사람 수는 반올림하지 않는다.

**참고자료**

부록 : 1. 졸업생의 진로 현황

## 3-4-2 의사국가시험 결과를 분석하여 문제점을 개선하려는 노력을 하고 있는가?

### 기본기준

의사국가시험 결과를 분석하여 문제점을 개선하려는 노력을 하고 있다.

### 보고서 기술내용

최근 2년간 의사국가시험 합격률을 실기와 필기, 재학생과 졸업생으로 나누어 기술하고, 문제점에 대한 개선노력을 기술한다.

#### 참고자료

부록 : 1. 최근 2년간 의사국가시험 합격률 현황 및 분석 자료
2. 의사국가시험 불합격자를 위한 방안과 문제점 개선 실적

의학교육 평가인증 기준

# 04 교수

## 4-1 전임교수

**평가개요**

대학은 기초의학과 임상의학, 의료인문학 등 교육을 충실히 할 수 있도록 능력 있는 전임교수를 적정 수 확보하고 있어야 한다.

### 4-1-1 기초의학 전임교수를 적절하게 확보하고 있는가?

**기본 기준**

대학은 세계의학교육협회가 권고하는 기초의학 13개 분야별로 교원을 적절하게 확보하고 있다.

**보고서 기술내용**

기초의학 전임교수 수를 전공 분야별로 기술하고, 이를 기본기준과 비교하여 현재의 상태를 기술한다. 아울러, 최근 2년간의 기초의학 전임교수 임용과 퇴직 현황을 기술한다.

[주] 분야는 과목 또는 교실을 의미하는 것이 아니라 교육내용을 의미한다.

[주] 겸임교수는 정원에서 제외하나 대학에서 정규 급여를 지급하는 기금교수는 정원에 포함한다. 겸임교수라 함은 타 단과대학 및 타 대학교에 적을 두고 있으면서 해당 대학에 교수직을 겸임하고 있는 것을 말한다. 기금교수는 교육부 정원에 해당되지는 않지만, 교육, 연구와 진료에서 전임교수와 같은 활동을 하는 것이 확인된 경우 이를 포함한다.

[주] 기초의학 분야는 세계의학교육협회(WFME)의 기준인 해부학(anatomy), 생리학(physiology), 생화학(biochemistry), 면역학(immunology), 병리학(pathology), 약리학(pharmacology), 미생물학(microbiology), 유전학(genetics), 분자생물학(molecular biology), 생물물리학(biophysics), 세포생물학(cell biology) 예방의학(preventive medicine) 및 기생충학(parasitology) 등을 포함한다.

## 우수기준

각 기초의학 분야별로 교육경력이 우수한 전임교수가 확보되어 있다.

[주] 기초의학 전임교수의 교육경력은 의과대학 졸업 후 고등교육기관 근무 경력으로 조교 경력을 포함하여 교육 관련 분야 경력을 의미한다.

### 참고자료

부록 : 1. 기초 전임교수 현황

비치 : 1. 인사기록 (급여대장 포함)

## 4-1-2 의학교육학 전임교수를 적절하게 확보하고 있는가?

### 기본기준

의학교육학교실(학과, 센터 등)이 개설되어 있으며, 의학교육 전임교수가 있다.

### 보고서 기술내용

의학교육학 전임교수의 수를 기술하고, 이를 기본기준과 비교하여 현재의 상태를 기술한다. 아울러, 최근 2년간 의학교육 전임교수 임용과 퇴직 현황을 기술한다.

[주] 전임교수는 해당 분야의 전공자로서 연구강사 이상의 신분이거나, 의과대학 교수인 경우 행정적으로 의학교육학교실(학과, 센터 등)에 소속되어 타 교실에 겸직으로 발령되어 있지 않은 자로서 근무시간의 80% 이상을 의학교육 업무에 할애해야 한다.

### 우수기준

의학교육학 전임교수 또는 전담교수를 충분히 확보하고 있다.

[주] 전담교수는 의학교육학교실(학과)에 겸임으로 발령되어 있고, 별도의 업적평가와 승진규정이 있으며 의학교육 관련 업무에 대한 근거 자료를 제시할 수 있어야 한다.

## 4-1-3 의료인문학 전임교수를 적절하게 확보하고 있는가?

### 기본기준

의료인문학 분야에 전임교수 또는 전담교수를 적절하게 확보하고 있다.

### 보고서 기술내용

의료인문학 전임교수 또는 전담교수의 수를 전공 분야별로 기술하고, 이를 기본기준과 비교하여 현재 상태를 기술한다. 아울러, 최근 2년간의 의료인문학 전임교수 또는 전담교수의 임용과 퇴직 현황을 기술한다.

[주] 전임교수는 해당 분야의 전공자로서 연구강사 이상의 신분이거나, 의과대학 교수인 경우 행정적으로 의료인문학 교실(학과, 센터 등)에 소속되어 타 교실에 겸직으로 발령되어 있지 않은 자를 의미한다.

[주] 전담교수는 의과대학 교수로서 의료인문학 교육과정에 소속된 자를 의미한다.

### 우수기준

의료인문학 관련 교실(학과, 센터 등)이 개설되어 있고, 의료인문학 전임교수가 충분히 확보되어 있다.

## 4-1-4 기초의학, 의학교육학, 의료인문학의 교육과 연구를 보조하는 인력은 적절한가?

### 기본기준

기초의학, 의학교육학, 의료인문학의 교육과 연구를 보조하는 인력으로 대학에서 직접적으로 재정적 지원을 하는 조교, 연구원이 분야별로 적절하다.

### 보고서 기술내용

기초의학, 의학교육학, 의료인문학의 교육과 연구를 보조하는 조교의 명단을 교실 또는 관련 분야별로 기술하고, 이들의 교육과 연구지원 업무 비율을 기술한다.

[주] 조교, 연구원의 1인당 재정지원의 기본수준은 적정급여와 대학원 전액등록금을 합한 것을 의미한다. 재정지원이라 함은 연구비, 장학금 등을 포함한다.

### 우수기준

기초의학, 의학교육학, 의료인문학의 교육과 연구를 보조하는 인력으로 조교 또는 대학에서 직접적으로 재정적 지원을 하는 연구원이 충분하다.

[주] 조교, 연구원은 교실 또는 학과별로 계산한다.

#### 참고자료

부록 : 1. 기초의학, 의학교육학 및 의료인문학의 교육과 연구보조인력 현황
비치 : 1. 인사기록 및 급여대장 또는 연구비 및 장학금 지급 대장

## 4-1-5 임상의학 전임교수를 적절하게 확보하고 있는가?

### 기본기준

각 임상의학 전공과목별로 전임교수를 적절하게 확보하고 있다.

### 보고서 기술내용

임상의학 전임교수의 수를 전공 분야별로 기술하고, 이를 기본 조건과 비교하여 현재의 상태를 기술한다. 아울러, 최근 2년 간 임상의학 전임교수 임용과 퇴직 현황을 기술한다.

[주] 임상의학 전임교수의 교육경력은 의과대학 졸업 후의 교육 기관 근무 경력으로 강사급(전임의, 연구강사, 임상강사 등) 경력을 포함하여 교육 관련 분야 경력을 의미한다. 단, 인턴과 전공의 수련 경력은 포함하지 않는다.

### 우수기준

각 임상의학 전공과목별로 교육, 연구, 진료경력이 우수한 전임교수가 확보되어 있다.

**참고자료**

부록 : 1. 임상 전임교수 현황
비치 : 1. 인사기록 (급여대장)

## 4-1-6 기초의학, 의학교육학, 의료인문학, 임상의학 전임교수의 구성은 적절한가?

### 기본기준

의과대학 전체 교원 중 동일 대학 졸업자 비율이 적절하다.

### 보고서 기술내용

의과대학 교원을 기초의학, 의학교육학, 의료인문학, 임상의학과 전체 교원으로 구분하여 성별, 연령별, 직급별, 출신학교별, 최종학력별로 그 현황을 기술한다.

### 우수기준

기초의학과 임상의학교실에서 주임교수 공모제가 실시되고 있다.

**참고자료**

부록 : 1. 기초의학, 의학교육학, 의료인문학 및 임상의학 전임교수의 성별, 연령별, 직급별, 출신학교별, 최종학력별 현황
2. 주임교수 공모제 규정 및 실적

## 4-2 교수업무

**평가개요**

교수는 성실하게 연구를 수행하고 우수한 연구업적을 많이 발표하여 학문발전에 이바지하여야 한다. 또한, 대학은 교수들이 연구를 효과적으로 수행할 수 있도록 부설연구소를 내실화하고 행정, 재정적 지원을 위해 노력하여야 한다.

### 4-2-1 교수들의 국내외 연구 실적이 적절한가?

**기본기준**

전임교수의 국내외 연구 실적이 적절하다.

**보고서 기술내용**

최근 2년간 전임교수들의 국내외 발표 연구 실적(원저, 종설, 증례보고, 저서, 역서)을 교수 100명당 연평균 논문 수로 계산한다.

**우수기준**

전임교수의 국내외 연구 실적이 우수하다.

**참고자료**

비치 : 1. 최근 2년간의 연구 실적 목록 (DB로도 제시 가능)

## 4-2-2 대학 외부로부터 받은 연구비 수준은 적절한가?

### 기본기준

전임교수의 최근 2년간 연평균 외부 연구비 수준이 적절하다.

### 보고서 기술내용

최근 2년간 대학 외부로부터 받은 연구비에 대해서 교실별 연구자, 연구제목, 연구기관과 연구비 총액 표를 만들고 전임교수 1인당 연평균 수혜 연구비를 계산하여 기술한다.

[주] 제 1, 2, 3 상(Phase I, II, III)의 임상시험연구비는 외부로부터 받은 연구비에 포함한다.

### 우수기준

전임교수의 최근 2년간 연평균 외부 연구비 수혜가 우수하다.

#### 참고자료

비치 : 1. 최근 2년간 대학 외부 연구비 수혜 현황 목록
2. 최근 2년간 대학 외부 연구비 수혜 현황 증빙자료

## 4-2-3 대학 내부 연구비 수준은 적절한가?

### 기본기준

전임교수의 최근 2년 동안 대학 내부로부터 받은 연구비 수준이 적절하다.

### 보고서 기술내용

최근 2년 동안 학내로부터 받은 연구비에 대해서 교실별 연구자, 연구제목, 연구기관 및 연구비 총액 표를 만들고 전임교수 1인당 연평균 수혜 연구비를 계산한다.

### 우수기준

전임교수의 최근 2년간 대학 내부로부터 받은 연구비가 우수하다.

**참고자료**

비치 : 1. 최근 2년간 교내 연구비 수혜현황 목록
2. 최근 2년간 교내 연구비 수혜현황 증빙자료

## 4-2-4 부설연구소의 연구 및 학술활동은 활발한가?

### 기본기준

의과대학 자체의 연구기금으로 운영하는 부설연구소가 있고, 부설연구소 연구기금에 의해 운영된 최근 2년간 학술활동 지원 경비와 연구비 지급 실적이 있다.

### 보고서 기술내용

의과대학이 운영하는 부설연구소 유무, 부설연구소 운영현황(전담 인력 포함)과 연구소를 통한 최근 2년 간 교수들의 연구와 학술활동 지원현황(연구비 지급액수와 건수)을 기술한다.

[주] 부설연구소 연구기금에 의해 운영된 연구비 지급 액수와 지급 건수 등은 대학의 규모를 고려하여 평가자가 판단한다.

#### 참고자료

부록 : 1. 최근 2년간 부설 연구소 연구기금에 의해 지원된 학술활동 및 경비지원 내역과 지급된 연구비 지급액수 및 연구비 지급 건수 목록

비치 : 1. 최근 2년간 부설 연구소 운영내역(회의자료 및 예·결산 관련자료)과 연구기금에 의해 지급된 최근 2년간 연구비 지급액수 및 지급 증빙자료

## 4-2-5 교수의 학회활동 등 사회봉사활동이 보장되고 있는가?

### 기본기준

교수의 학생교육, 연구와 진료 이외의 학회활동 등 사회봉사활동이 보장되고 있다.

### 보고서 기술내용

교수의 학생교육, 연구와 진료 이외의 학회활동 등 사회봉사활동이 보장되고 있는지 기술한다.

#### 참고자료

비치 : 1. 최근 2년간 사회봉사활동 실적
2. 관련 교수복무규정
3. 출장기록 등

## 4-3 교수개발

**평가개요**

대학은 교수들의 교육, 연구와 진료 능력 개발을 위한 각종 연수 교육 기회를 제공하여야 하며, 합리적인 교수 업적 평가 제도를 갖추고 적절하게 활용하여야 한다.

### 4-3-1 교수 업적 평가가 이루어지고 있으며 그 결과가 적절하게 활용되고 있는가?

**기본기준**

교수 업적 평가 제도가 정립되어 있고 교수들의 직급승진에 반영하고 있다.

**보고서 기술내용**

전년도 교수 업적 평가(교육·연구·봉사) 서식을 제시하고 평가내용, 기준과 그 결과 활용 등에 대해 기술한다.

**우수기준**

교수 업적 평가가 직급별(연구전임, 조교수, 부교수, 교수), 기능별(기초의학, 임상의학, 의료인문학, 의학교육학 등)로 세분화되어 있고, 직급승진에 관한 규정이 세분화되어 있고 별도의 업적 평가 제도가 있다.

**참고자료**

부록 : 1. 교수 업적 평가제도 및 현황(평가기준, 실제 평가자료 등)

## 4-3-2 교수 업적 평가 기준에 의학교육과 교수개발에 관련된 내용을 포함하고 있으며, 의학교육 관련 연수교육이나 자체 교수 개발 프로그램의 참여 정도가 적절한가?

### 기본기준

교수 업적 평가 규정에 전임교수들에게 의학교육과 관련된 연수교육이나 교수 개발 프로그램에 의무적으로 참여해야 하는 연간 교육시간이 명시되어 있고, 교수들이 적절하게 참석하고 있다.

### 보고서 기술내용

교수업적평가 규정에 의학교육 관련 연수교육이나 자체 교수개발 프로그램에 대한 연간 의무적인 참여시간이 명시되어 있는지, 또는 이와 관련된 인센티브 제도가 있는지에 대하여 기술한다. 최근 2년간 자체적으로 주관했던 순수 의학교육 관련 연수와 교수개발 프로그램에 대하여 개최장소, 일시 참석인원, 주요 내용 등을 기술한다.

[주] 재직교수에 장·단기 해외 연수자는 제외한다.

### 우수기준

교수 업적 평가 규정에 의학교육 연수 실적이 의무화되어 있으며, 교육업적으로 승진이 가능한 교수 업적 평가 규정이 있고, 강의식 이외의 학생교육과 관련된 교육활동(PBL, TBL 등 다양한 교육 프로그램)에 대하여 인센티브제도가 마련되어 있다. 의학교육 관련 연수 교육에 최근 2년 동안 참여한 실적이 우수하다.

#### 참고자료

비치 : 1. 의학교육 관련 세미나 워크숍 등 관련 자료(책자, 프로그램 참석자 명단 등)
부록 : 1. 교수 업적 평가제도 및 현황(평가기준, 실제 평가자료 등)

## 4-3-3 신임교원을 위한 의학교육 연수과정이 적절하게 시행되고 있는가?

### 기본기준

신임교원을 위한 의학교육 연수과정이 의무적으로 적절하게 시행되고 있다.

### 보고서 기술내용

신임교원을 위한 의학교육 연수가 의무화되어 있는지 기술하고 자체 또는 외부 의학교육 프로그램에 대하여 기술한다.

**참고자료**

비치 : 1. 의학교육 관련 세미나 워크숍 등 관련 자료(책자, 프로그램 참석 신임교원 명단 등)

## 4-3-4 교수 업적 평가 기준에 봉사에 관한 내용이 포함되어 있는가?

### 기본기준

교수 업적 평가 기준에 교수의 학생교육이나 학술활동 이외의 학회활동이나 공공 목적의 사회봉사활동에 대한 평가기준이 마련되어 있다.

### 보고서 기술내용

교수 업적 평가 기준에 사회봉사활동에 대한 평가기준이 마련되어 있는지 또는 이와 관련된 인센티브 제도가 있는지 기술한다.

## 4-3-5 교수들의 장 · 단기 외국 연수와 국내외 학회 참석을 위한 재정적 지원체제를 적절히 갖추고 있는가?

### 기본기준

교수들의 장·단기 외국연수와 국내외 학회 참석을 위한 재정적 지원이 적절하다.

### 보고서 기술내용

교수들의 장·단기 외국연수와 국내외 학회 참석을 장려하기 위한 재정적 지원체계(등록비, 항공료, 체제비 등)가 갖추어져 있는지를 최근 2년간 지원 실적 내용과 함께 기술한다. 연구년(안식년 포함) 제도를 시행하는 경우 그 내용과 실적도 함께 기술한다.

[주] 재정적 지원에는 병원의 지원도 포함한다.

### 우수기준

교수들의 장·단기 외국연수와 국내외 학회 참석을 위한 재정적 지원이 우수하다.

#### 참고자료

비치 : 1. 최근 2년간 장기 및 단기 해외연수를 위한 재정지원 실적자료
2. 단기학회 참가를 장려하기위한 재정적 지원체계 규정

## 4-3-6 연구윤리에 관한 기본 교육이 시행되고 있는가?

### 기본기준

연구윤리에 대한 의과대학의 자체 규정이 마련되어 있으며 정기적으로 연구윤리교육이 시행되고 있다.

### 보고서 기술내용

교수를 대상으로 연구윤리에 관한 기본적인 교육이 이루어지고 있는지 기술한다.

#### 참고자료

부록 : 1. 연구윤리에 대한 자체규정 및 교육 현황(규정, 교육자료 및 출석자료 등)

## 4-3-7 성희롱 예방교육이 시행되고 있는가?

### 기본기준

성희롱 예방교육에 대한 대학의 자체 업무규정이 있으며 정기적으로 교육이 시행되고 있다.

### 보고서 기술내용

교수를 대상으로 성희롱 예방에 관한 기본적인 교육이 이루어지고 있는지 기술한다.

**참고자료**

부록 : 1. 성희롱 예방교육에 대한 자체규정 및 교육 현황(규정, 교육 자료와 출석자료 등)

# 05 시설·설비

## 5-1 교육 시설·설비

**평가개요**

대학은 학생 교육을 위하여 교육과정과 일치하는 충분한 교육기본 시설과 교육지원 시설을 갖추고 효율적으로 관리해야 한다.

### 5-1-1 대학은 학생 교육을 위한 교육기본 시설을 충분하게 갖추고 있는가?

**기본기준**

대학은 학생 교육을 위한 교육기본 시설을 충분하게 갖추고 있다.

**보고서 기술내용**

교육과정의 내용과 학생 규모를 고려한 충분한 수의 교육기본 시설의 현황과 각 시설의 용도에 적합한 설비와 여건을 기술한다. 실험실습 기자재의 사용 실적에 대하여 기술한다.

[주] 대학의 교육기본 시설은 학생 전용 강의실과 실험 실습실을 의미한다.

[주] 시설의 설비와 여건은 멀티미디어 설비, 조명, 냉난방, 방음, 환기, 청결 상태 등을 의미한다.

**참고자료**

비치 : 1. 실험 실습실의 기자재 보유 목록
(실험 실습실에 놓여 있는 실험실습용 기자재 목록만 해당함.)
2. 실험 실습실의 기자재 사용 일지

현지확인 : 1. 강의실의 위치와 설비 여건을 확인
2. 실험 실습실의 위치 및 기자재와 설비 여건을 확인

## 5-1-2 대학은 학생 교육을 위한 교육지원 시설을 충분하게 갖추고 있는가?

### 기본기준

대학은 학생 교육을 위한 교육지원 시설을 충분하게 갖추고 있다.

### 보고서 기술내용

교육과정의 내용과 학생 규모를 고려한 충분한 수의 교육지원시설의 현황과 각 시설의 용도에 적합한 설비와 여건을 기술한다. 학생 임상교육을 위한 적정한 규모의 술기실습실(Clinical Skills Lab.)과 표준화 환자를 이용한 교육 및 평가시설을 기술한다. 이러한 교육지원 시설과 교육과정의 연계성을 기술한다.

[주] 교육지원 시설은 강의실외의 학습토론 공간으로 문제바탕학습실, 팀바탕학습실, 소그룹토의실, 자율학습실, 정독실, 컴퓨터실, 임상술기실습실, 표준화 환자를 이용한 교육과 평가시설, 컴퓨터기반시험(computer-based test)을 실시할 수 있는 멀티미디어실 등을 의미한다. 교육과정에 맞추어 전 학년이 교육지원 시설을 이용하여야 한다.

[주] 적정한 규모의 술기실습실이란 임상술기 실습을 위한 기본 장비를 갖추고 있으며, 학생 수 대비 그 규모는 적절하며, 특정 전공교실에 포함되지 않고 독립된 공간에 설치되어 있어 학생들이 편리하게 이용할 수 있어야 한다. 표준화 환자를 이용한 교육과 평가 시설은 대학의 교육 과정에 따라 강의와 실습 교육 및 평가에 지속적으로 활용되고 있는 시설이어야 한다.

#### 참고자료

비치 : 1. 각 교육지원 시설의 배치도
2. 시설의 기자재와 설비의 목록
현지확인 : 1. 교육지원 시설의 위치와 기자재 및 설비의 목록 확인

## 5-1-3 대학은 학생복지 및 편의시설을 고루 갖추고 있으며, 그 수준은 적절한가?

### 기본기준

학생회방, 동아리방, 남·여 휴게실, 운동시설, 식당, 매점, 자동판매기, 개인용 캐비닛 등을 적절하게 갖추고 있다.

### 보고서 기술내용

학업에 충실할 수 있는 면학분위기 조성을 위하여 학생회방, 동아리방, 남·여 휴게실, 운동시설, 식당, 매점, 자동판매기, 개인용 캐비닛 등의 복지 및 편의 시설을 갖추고 있는지를 기술한다. 이러한 시설들이 학교 내에 학생들의 생활공간 주위에 위치하고 있어 편리하게 이용할 수 있는지와 시설의 면적이 충분한지를 기술한다.

**참고자료**

부록 : 1. 복지 및 편의 시설 현황
비치 : 1. 복지 및 편의 시설 운영 및 관리 자료
현지확인 : 1. 복지 및 편의 시설의 설비와 여건

## 5-1-4 대학은 학생의 교육과 복지를 위한 시설·설비를 효율적으로 관리하고 있는가?

### 기본기준

학생의 교육과 복지를 위한 시설·설비의 관리 인력이 있고, 적절한 예산이 배정되어야 한다.

### 보고서 기술내용

학생 교육을 위한 교육기본 시설과 교육지원 시설, 학생복지 및 편의시설 등의 관리체계에 대해서 기술하며, 각 시설·설비의 만족도를 조사하여 운영과 관리에 활용하고 있는지를 기술한다. 교육과 복지시설의 운영과 관리에 대한 만족도 설문조사와 그 결과에 따른 개선 실적을 기술한다.

[주] 시설·설비의 관리와 활용실태는 ① 시설배치 ② 관리조직(인력, 역할분담) ③ 관리 예산과 지원 ④ 업무의 효율성과 효과성 ⑤ 심미성 ⑥ 공해방지시설의 적절성 등을 중심으로 평가한다.

#### 참고자료

비치 : 1. 시설관리 예산 집행서
2. 각종 시설·설비를 관리·유지·운영하는 직원의 명단
3. 교육과 복지시설과 이들의 운영과 관리에 대한 만족도 설문조사 결과표 (학기별)

현지확인 : 1. 교육기본 시설, 교육지원 시설, 복지·편의시설의 관리 상태 확인
2. 시설과 설비 관리 직원 면담

## 5-1-5 대학은 임상실습 교육을 위하여 대학부속 교육병원을 확보하고 있으며, 병원 내에 학생교육 시설을 갖추고 있는가?

### 기본기준

대학은 임상실습 교육을 위하여 유효병상 500병상 이상의 대학부속 교육병원을 확보하고 있다. 병원 내에는 각종 학생 교육시설을 갖추고 있으며, 적절한 학생 전용공간이 확보되어 있다.

### 보고서 기술내용

임상실습 교육을 위한 대학부속 교육병원의 규모, 교육병원으로서의 역할, 병원 내 학생 교육 시설과 설비 및 학생 전용공간에 대해 기술한다. 임상실습 교육이 이루어지는 병원이 여러 개이면 각각에 대해서 기술한다. 병원의 학생 전용공간으로 확보된 실의 수, 면적 및 설비와 실제 활용에 대해서 기술한다. 병원 내 학생교육 시설과 설비 및 학생전용 공간에 대한 만족도 조사와 이를 활용한 개선실적을 기술한다.

[주] 병원 내 각종 학생교육 시설은 임상실습 교육과정에 따라 학생교육을 위해 실제로 사용하는 시설을 의미한다. 단, 술기실습실과 표준화환자 이용 시설 등은 5-1-2에서 기술하며, 도서관 관련 시설은 5-1-6에서 기술한다. 또한 학생 전용 공간에서 전공의, 간호사 등 타 인력과 공동으로 사용하는 공간은 제외하며, 학생들이 임상실습 기간 중에 학습과 휴식을 취할 수 있는 공간만을 계산한다. 실습병원이 지리적으로 떨어져 있으면 각 병원별로 학생 교육시설(병원자료와 인터넷 검색이 가능한 컴퓨터, 개인사물함 등이 구비된 자율학습실)이 갖추어져 있어야 한다.

[주] 임상실습이 협력병원에서도 이루어질 경우, 협력병원은 부속병원과 같은 조건의 시설·설비를 갖추고 있어야 한다.

### 우수기준

학생인턴제를 위한 당직실이 확보되어 있다.

#### 참고자료

부록 : 1. 병원 내 교육시설의 배치도
2. 학생인턴제 규정

비치 : 1. 병원 내 학생교육 시설의 위치 및 사용에 대한 만족도 조사(학생 설문조사표)와 이를 활용한 개선실적

현지 확인 : 1. 대학 부속교육병원의 규모와 역할 확인
2. 교육병원 내 교육시설 및 학생 전용공간 확인
3. 학생면담: 병원 내 학생교육 시설과 학생전용공간 활용실태 확인

## 5-1-6 대학은 교육과 연구를 지원하기 위한 학술정보서비스 체계를 구축하고 편리하게 이용할 수 있도록 하고 있는가?

### 기본기준

교육과 연구를 지원하기 위한 독립적인 학술정보서비스 체계가 구축되어 있으며, 매년 지출되는 예산이 적절하다. 교육과 연구를 지원하기 위한 전문 인력(의학전담 사서)이 확보되어 있다.

### 보고서 기술내용

교수의 연구와 학생의 교육을 지원하기 위한 학술정보서비스 체계에 대해서 기술한다. 도서관의 전자도서를 포함한 단행본, 전자저널을 포함한 학술지, CD-ROM, on-line DB 등의 확보 실태와 그 적절성 및 구입을 위한 최근 2년간의 예산을 기술한다. 대학과 교육병원이 지리적으로 떨어져 있으면 각 단위별로 학술정보서비스 체계를 기술한다.

[주] 학술정보서비스가 대학 본부의 중앙도서관에 포함되어 있으면, 의과대학 교수 및 학생을 위한 학술정보서비스가 어떻게 이루어지고 있는지 기술한다. 전자도서를 포함한 단행본, 전자저널을 포함한 학술지, CD-ROM, on-line DB 등은 의학과 관련된 것만 기술한다. 인력 및 예산도 별도로 구분하여 기술한다.

[주] 학술정보서비스 체계가 독립적으로 구축되어 있다는 것은 대학의 도서관 또는 온라인 시스템을 통한 학술정보서비스가 의과대학 교수의 연구와 학생의 교육을 지원하기 위해 독립적으로 운영되고 있는지를 의미한다. 또한, 대학과 병원이 지리적으로 떨어져 있는 경우에도 동일한 학술정보서비스를 제공받을 수 있어야 한다.

[주] 접근성은 전자저널을 포함한 학술도서 및 학술지의 열람 및 출력의 용이성을 의미한다.

[주] 매년 지출되는 예산은 교수와 학생을 합한 총 수에서 1인당으로 계산한다.

### 우수기준

매년 지출되는 예산과 전문인력(의학전담 사서)이 충분하다.

#### 참고자료

부록 : 1. 의학전담사서 현황표

현지 확인 : 1. 의학 전용도서관의 규모와 시설, 도서 및 저널 확보 상태
2. 전산화 프로그램을 통한 학술정보서비스 실태
3. 교수·학생 면담: 학술정보서비스의 접근성 확인
4. 의학 전용 도서관의 최근 2년간 예결산 자료

## 5-1-7 대학은 교육정보화 시스템을 구축하고, 구성원이 편리하게 이용할 수 있도록 하고 있는가?

### 기본기준

대학의 학사 및 교육과정 운영과 관련된 교육정보화 시스템이 구축되어 있으며, 구성원들이 편리하게 이용할 수 있다.

### 보고서 기술내용

대학의 교육정보화 시스템의 구축과 활용 현황에 대해서 기술한다. 교육정보화 시스템에는 학사관리 시스템, 홈페이지 활용, e-learning 시스템 등에 대해서 기술하고 그 적절성을 기술한다. 교육정보화 시스템의 구축 및 활용과 관련된 최근 2년간의 집행 예산을 함께 기술한다.

[주] e-learning은 교수·학습 자료를 단순히 제공하는 수준이 아니라 상호작용이 가능한 시스템이어야 하며, 시스템을 통하여 교육내용이 제공되고, 실제 교수·학습이 이루어지며, 학습관리가 이루어짐을 의미한다. e-learning을 통한 교과목 개설(사이버 강좌 등) 등으로 실제 활용되고 있음을 증명할 수 있어야 한다.

### 우수기준

e-learning 시스템이 갖추어져 있으며, 실제 활용되고 있다.

#### 참고자료

현지확인 : 1. 교육정보화 시스템 시연
2. 교수, 학생, 직원 면담: 교육정보화시스템의 활용도 확인
3. 교육과정 내 e-learning 통한 평가자료 제시

## 5-2 연구 시설·설비

**평가개요**

대학은 개인 교수실과 교수들의 연구 공간과 시설을 충분히 확보하고, 행정지원 체계를 갖추고 있어야 한다.

### 5-2-1 대학은 개인 교수실과 그 실내 설비를 확보하고, 행정지원 체계를 갖추고 있는가?

**기본기준**

개인 교수실이 충분히 확보되어 있고, 행정지원 체계를 갖추고 있다.

**보고서 기술내용**

전임강사 이상의 개인 교수실의 확보현황(전체 교원 수에 대한 개인 연구실 수의 비율)와 교수실의 크기와 실내 설비(조명, 냉난방, 방음, 환기, 채광, LAN 설치 등) 상태를 기술한다. 기초의학 및 임상의학 교실(과)별 교수의 행정업무 지원체계를 기술한다.

[주] 개인 교수실은 교수 단독으로 사용하는 공간으로 진료실 겸용으로 사용하는 공간은 제외하며, 적정한 크기의 공간이어야 한다.

**우수기준**

전체 교수가 개인 교수실을 확보하고 있다.

**참고자료**

부록 : 1. 개인 교수실 확보 실태
2. 교실(과)별 행정 업무지원 인력 현황서
현지확인 : 1. 교수실의 크기, 내부 실내 설비 및 주위 환경 확인
2. 행정업무지원 인력면담

## 5-2-2 대학은 교수들의 연구를 위한 충분한 공간과 시설 및 설비를 갖추고 있는가?

### 기본기준

대학은 교수들의 연구를 위한 충분한 공간과 시설 및 설비를 갖추고 있다.

### 보고서 기술내용

각 과별 연구실과 공동 연구실의 확보 현황과 연구기자재 확보 및 관리 실태를 기술한다. 교수들이 각 연구공간을 효율적으로 사용할 수 있도록 지원하고 있는지를 기술한다.

**참고자료**

부록 : 1. 연구시설 현황

비치 : 1. 교수 연구 공간과 시설의 확보 현황 및 운용 상태

2. 과별 연구실과 공동 연구실의 기자재의 종류와 보유 현황 및 관리 상태

의학교육 평가인증 기준

# 06 졸업 후 교육

## 6-1 대학원 교육

**평가개요**

대학은 학구적인 환경 조성을 위해 대학원 교육과정을 적절하게 편성하여 운영하여야 하며, 다양한 경력자가 대학원에 진학할 수 있도록 하여야 한다. 또한, 대학원생을 지도하는 의학교육 프로그램은 지적인 도전을 촉진시키는 환경에서 수행되어야 한다.

### 6-1-1 대학원 교육은 체계적인 계획 하에 충실하게 수행되고 있는가?

**기본기준**

전공과목별로 학습목표가 포함된 수업계획서가 있고, 교육은 수업계획서대로 충실하게 수행되고 있다.

**보고서 기술내용**

전공과목별 교육과정과 해당 수업계획서를 제시하고 이 계획서가 잘 지켜지도록 대학이 노력한 내용과 학습평가 방법을 기술한다. 또한, 학위관리는 어떻게 하고 있는가를 기술하고 특성화된 대학원 편제(학·석사, 석·박사 통합, 의사박사 복합, 학제간 복합학위제도 등)와 그 운용내용을 기술한다.

**참고자료**

부록 : 1. 전공과목별 교육과정
비치 : 1. 전공과목별 수업계획서
2. 전공과목별 학습평가결과
3. 수업계획서를 준수하도록 지시한 공문 또는 교수회의 내용
4. 학위관리 현황

## 6-1-2 대학원 업무체계는 적절한가?

### 기본기준

대학원 업무를 전담하는 기구 및 인력과 그 운용을 위한 예산과 집행이 합리적이다.

### 보고서 기술내용

대학원 업무를 전담하는 기구 및 인력현황을 기술한다. 또한 운용을 위한 예산과 그 집행 현황 등을 기술한다.

#### 참고자료

부록 : 1. 대학원 행정기구표
비치 : 1. 대학원 업무를 전담하는 기구 및 인력 현황
2. 예산 및 집행 현황

## 6-1-3 학생선발과 학생지원체제는 적절한가?

### 기본기준

특성화 된 대학원 편제에 합당한 다양한 경력을 소유한 학생을 선발하고 있으며, 등록금 총액 대비 교내·외 장학금 또는 특별지원금이 적절하다.

### 보고서 기술내용

최근 2년간 대학원생의 경력에 따른 구성과 등록금 총액 대비 장학금 총액의 비율, 연구지원 시설, 실험실습비의 확보상태를 기술한다.

### 우수기준

교내·외 장학금 또는 특별 지원금이 등록금 총액 대비 우수하며, 대학원생 전용 연구공간이 있다.

**참고자료**

부록: 1. 최근 2년간 대학원생의 경력(학부전공 포함)에 따른 구성
2. 최근 2년간 납부 등록금 총액 및 장학금 총액과 실험실습비의 확보상태

비치: 1. 최근 2년간 전체 대학원생의 학부전공 확인서류
2. 최근 2년간 장학금 지급내역
3. 실험실습비의 지급내역
4. 대학원생을 위한 연구 공간 확인

# ASK2019 의학교육 평가인증기준

## ASK2019
## (Accreditation Standards of KIMEE 2019)
## 의학교육 평가인증 기준

### ■ 명칭

2019년 의학교육 평가인증부터 전면 도입할 새 의학교육 평가인증 기준을 ASK2019(Accreditation Standards of KIMEE 2019)라 함.

### ■ 원칙

한국의학교육평가원(KIMEE, Korean Institute of Medical Education and Evaluation)은 기본의학교육의 질 향상을 목적으로 세계의학교육연합회(WFME, World Federation for Medical Education)에서 제시한 Basic Medical Education WFME Global Standards for Quality Improvement(The 2015 Revision)를 근간으로 우리나라의 기본의학교육 상황을 고려하여 2019년부터 전면 시행할 의학교육 평가인증 기준을 마련함.

### ■ 방향

**1. ASK2019의 전체적인 구조와 구성은 WFME Global Standards를 근간으로 함.**

**가. 평가영역과 평가부문** :

- 9개 평가영역에 대한 36개 평가부문으로 구성
- 평가영역(평가부문 수) : 1. 사명과 성과(4), 2. 교육과정(8), 3. 학생평가(2), 4. 학생(4), 5. 교수(2), 6. 교육자원(6), 7. 교육평가(4), 8. 대학운영체계와 행정(5), 9. 지속적 개선(1)

**나. 평가기준의 종류**

- ASK2019는 기본기준과 우수기준으로 구분하고, 기본기준은 **K(Korea basic standard)**, 우수기준

은 **H**(High quality development standards)로 표현함.

**주)** WFME Global Standards는 Basic standards(B)와 Quality development standards(Q)으로 구분

- 기본기준 **K**는 의과대학(의학전문대학원 포함; 이하 '의과대학')과 기본의학교육이 충족해야 하는 기준으로 인증의 목적에 해당함.
- 우수기준 **H**는 의과대학과 기본의학교육의 미래지향적인 기준으로, 국제적으로 합의된 모범 사례에 준하여 대학이 자발적으로 의학교육의 개혁을 시행함을 목적으로 함.

**다. [주](Annotations)**

- **[주]**는 용어의 이해를 돕기 위하여 설명을 하거나 예를 들기 위한 것으로 각 기준 하단에 제시함.

**2. ASK2019의 내용은 WFME Global Standards를 근간으로 하되, 우리나라의 문화 및 의학교육 환경을 적절하게 반영함.**

- 우리나라에서 법적으로 규정되어 있는 것은 평가인증기준에 포함하지 않음.
- 기본의학교육에 속하지 않는 기준은 사용하지 않음.
- 우리나라 의학교육 상황에 맞지 않는 기준은 사용하지 않음.
- 우리나라 의학교육 상황에 맞게 Post-2주기 KIMEE 기준에 맞추어 추가 또는 수정함.

## 평가영역·부문별 기준 수

| 평가영역 | 평가부문 | 평가인증기준 수 | | |
|---|---|---|---|---|
| | | 기본기준 | 우수기준 | 합계 |
| 1. 사명과 성과 | 1.1 사명 | 3 | 1 | 4 |
| | 1.2 대학의 자율성과 학문의 자유 | 1 | - | 1 |
| | 1.3 교육성과 | 3 | 1 | 4 |
| | 1.4 사명과 교육성과 수립 | 1 | 1 | 2 |
| | 소계 | 8 | 3 | 11 |
| 2. 교육과정 | 2.1 교육과정 | 3 | 1 | 4 |
| | 2.2 과학적 방법 | 3 | - | 3 |
| | 2.3 기초의학 | 2 | 1 | 3 |
| | 2.4 의료인문학 | 1 | 1 | 2 |
| | 2.5 임상의학과 술기 | 4 | 3 | 7 |
| | 2.6 교육과정의 구조, 구성, 기간 | 2 | 2 | 4 |
| | 2.7 교육과정 관리 | 2 | - | 2 |
| | 2.8 의료행위와 보건의료분야의 연계 | 1 | 1 | 2 |
| | 소계 | 18 | 9 | 27 |
| 3. 학생평가 | 3.1 평가방법 | 4 | 1 | 5 |
| | 3.2 평가와 학습의 관계 | 4 | 2 | 6 |
| | 소계 | 8 | 3 | 11 |
| 4. 학생 | 4.1 입학정책과 선발 | 1 | 3 | 4 |
| | 4.2 입학정원 | 1 | - | 1 |
| | 4.3 학생상담과 지원 | 6 | 3 | 9 |
| | 4.4 학생대표 | 2 | - | 2 |
| | 소계 | 10 | 6 | 16 |
| 5. 교수 | 5.1 채용과 선발정책 | 6 | 1 | 7 |
| | 5.2 교수활동과 개발 정책 | 6 | 1 | 7 |
| | 소계 | 12 | 2 | 14 |
| 6. 교육자원 | 6.1 시설 | 8 | 1 | 9 |
| | 6.2 임상실습 자원 | 3 | 1 | 4 |
| | 6.3 정보기술 | 1 | 2 | 3 |
| | 6.4 의학연구와 의과학자 양성 | 3 | 1 | 4 |
| | 6.5 교육 전문성 | 2 | 3 | 5 |
| | 6.6 교육적 교류 | 1 | 1 | 2 |
| | 소계 | 18 | 9 | 27 |
| 7. 교육평가 | 7.1 교육 모니터링과 평가에 대한 체제 | 3 | 1 | 4 |
| | 7.2 교육자와 학생의 피드백 | 1 | 1 | 2 |
| | 7.3 학생과 졸업생의 수행 능력 | 1 | 1 | 2 |
| | 7.4 이해관계자의 참여 | 1 | - | 1 |
| | 소계 | 6 | 3 | 9 |
| 8. 대학운영체계와 행정 | 8.1 대학운영체계 | 4 | 2 | 6 |
| | 8.2 학장과 보직자 | 1 | 1 | 2 |
| | 8.3 교육예산과 자원 할당 | 2 | - | 2 |
| | 8.4 행정직원과 관리 | 1 | 1 | 2 |
| | 8.5 보건의료분야와의 상호작용 | 1 | 1 | 2 |
| | 소계 | 9 | 5 | 14 |
| 9. 지속적 개선 | 9.0 지속적 개선 | 3 | 11 | 14 |
| | 소계 | 3 | 11 | 14 |
| 합계 | 36 | 92 | 51 | 143 |

ASK2019
# 01 사명과 성과

## 1.1 사명

### 기본기준

**K.1.1.1 의과대학은 대학의 사명을 정하고, 이를 구성원들과 보건의료분야의 관계자들이 인지하도록 노력하고 있다.**

*자체평가연구보고서 작성 가이드*

가이드-1. 명시된 사명을 가지고 있다.
가이드-2. 사명은 구성원의 합의 또는 관련 위원회의 조직적인 노력을 거쳐 합리적으로 만들어졌다.
가이드-3. 사명을 홈페이지, 홍보자료 등을 통해 대학의 구성원과 보건의료분야 관련자들에게 알린다.

*방문평가단 평가 가이드*

| 기준 | 핵심어 | 가이드 | 근거자료 |
|---|---|---|---|
| K.1.1.1 | • 대학의 사명<br>• 구성원<br>• 보건의료분야 관련자 | • 명시된 사명이 적절하게 기술되었는지 확인한다.<br>• 사명이 구성원의 합의 또는 관련 위원회의 조직적인 노력을 거쳐 합리적으로 만들어졌는지 확인한다.<br>• 사명을 홈페이지, 홍보자료 등을 통해 대학의 구성원과 보건의료분야 관련자들에게 알리고 있는지 확인한다. | • 사명<br>• 사명 개발 절차와 배경<br>• 사명을 알린 홍보자료 |

**[주]**

- 의과대학은 의학전문대학원을 포함한다.
- 사명은 대학교의 설립이념을 바탕으로 의과대학이 추구하는 교육이념이 기술된 것을 의미한다.
- 의과대학 구성원은 의과대학의 학장과 보직자, 교직원, 학생을 포함하며, 다른 관련 이해관계자들도 포함한다.
- 보건의료분야는 공공 혹은 민간 의료 서비스 전달 체계와 의학 연구 기관을 포함한다.

## 기본기준

**K.1.1.2 의과대학의 사명에는 기본 수준의 진료역량, 다양한 의학 분야 활동에 필요한 기본적인 조건, 그리고 졸업 후 교육과 평생 학습하는 의사양성을 위한 내용 등이 명시되어 있다.**

*자체평가연구보고서 작성 가이드*

가이드-1. 사명에는 기본 수준의 역량을 갖춘 의사를 배출하기 위한 교육전략이 있다.
가이드-2. 다양한 의학 분야에서 활동하기 위한 적절한 기본을 갖춘 의사, '기본의학교육 졸업성과'와 '한국의 의사상'에서 제시한 의사의 역할을 수행할 수 있는 의사, 졸업 후 의학교육을 위한 준비가 되어 있는 의사, 평생학습을 위해 노력하는 의사를 배출하기 위한 교육전략이 있다.

*방문평가단 평가 가이드*

| 기준 | 핵심어 | 가이드 | 근거자료 |
|---|---|---|---|
| K.1.1.2 | • 기본 수준의 역량<br>• 의사 양성 | • 사명에 기본 수준의 역량을 갖춘 의사를 배출하기 위한 교육전략이 있는지 확인한다.<br>• 다양한 의학 분야에서 활동하기 위한 적절한 기본을 갖춘 의사, '기본의학교육 졸업성과'와 '한국의 의사상'에서 제시한 의사의 역할을 수행할 수 있는 의사, 졸업 후 의학교육을 위한 준비가 되어 있는 의사, 평생학습을 위해 노력하는 의사를 배출하기 위한 교육전략이 있는지 확인한다. | • 사명 |

**[주]**

- 다양한 의학 분야는 임상의 모든 분야, 의료관리, 의학연구 등을 의미한다.
- 졸업 후 교육은 학위과정, 전문의 수련과정을 포함한다.
- 평생학습이란 평가, 감사(audit), 성찰 혹은 인정된 전문직업성 평생개발(Continuing Professional Development; CPD)/평생교육(Continuing Medical Education; CME) 활동을 통해, 최신 지식과 기술을 습득하는 전문적 책임성을 의미한다. 전문직업성 평생개발(CPD)에는 의사들이 환자들의 요구에 따라 지식, 술기, 태도를 유지, 개선, 발전, 향상시키기 위해 행하는 공식적 그리고 비공식적 모든 활동을 포함한다. 전문직업성 평생개발(CPD)은 의료행위의 지식과 술기의 지속적 교육을 나타내는 평생교육(CME)보다 광범위한 개념이다.

## 기본기준

### K.1.1.3 의과대학은 사명에 대학의 사회적 책무를 명시하고 있다.

| *자체평가연구보고서 작성 가이드* |
|---|
| 가이드-1. 사명에 지역사회의 건강 요구, 보건의료시스템 요구와 사회적 책무의 다양한 측면을 포함하고 있다.<br>가이드-2. 사명에 사회적 책무수행과 관련된 교육, 연구, 진료방침이 있다. |

*방문평가단 평가 가이드*

| 기준 | 핵심어 | 가이드 | 근거자료 |
|---|---|---|---|
| K.1.1.3 | • 사회적 책무 | • 사명에 지역사회의 건강 요구, 보건의료시스템 요구와 사회적 책무의 다양한 측면을 포함하고 있는지 확인한다.<br>• 사명에 사회적 책무수행과 관련된 교육, 연구, 진료방침이 있는지 확인한다. | • 사명 |

**[주]**

- 지역사회의 요구를 이행한다는 것은 지역 공동체, 특히 보건의료와 보건의료 관련 분야 공동체와의 상호작용, 지역사회의 보건의료 문제에 관한 관심, 지식을 표명하는 교육 과정의 조정을 내포한다.
- 사회적 책무(social accountability)는 사회, 환자, 보건의료, 보건의료 관련 분야의 요구에 반응하며, 의료 서비스, 의학 교육, 의학 연구의 경쟁력을 강화하여 국가와 세계 의학 발전에 기여할 수 있는 자발적 의지와 능력을 포함한다. 이러한 사회적 책무는 학교 자체 원칙에 기초하며, 학교의 자율성을 존중한다.
  사회적 책무는 때로 사회적 책임(social responsibility)과 사회적 대응(social responsiveness)과 같은 의미로 사용한다. 의과대학의 사회적 책무를 넘어서는 문제에 있어서도 의과대학은 그것에 대한 지지와 정책에 대한 관심 유도를 해야 한다.

## 우수기준

### H.1.1.1 의과대학은 사명에 의학연구와 국제보건의료와 관련된 내용을 포함하고 있다.

*자체평가연구보고서 작성 가이드*

가이드-1. 사명에 의학연구 관련 내용을 명시하고 있다.
가이드-2. 사명에 국제보건의료 관련 내용을 명시하고 있다.

*방문평가단 평가 가이드*

| 기준 | 핵심어 | 가이드 | 근거자료 |
|---|---|---|---|
| H.1.1.1 | • 의학연구<br>• 국제보건의료 관련 내용 | • 사명에 의학연구 관련 내용을 명시하고 있는지 확인한다.<br>• 사명에 국제보건의료 관련 내용을 명시하고 있는지 확인한다. | • 사명 |

**[주]**

- 의학연구는 기초의학, 의료인문학, 임상의학분야의 과학적 연구를 포함한다(H.6.4.1 참조).

## 1.2 대학의 자율성과 학문의 자유

### 기본기준

**K.1.2.1 의과대학은 자율성이 보장되고, 교수와 교직원이 교육과정의 설계와 실행, 그에 필요한 자원 활용 등에 있어 자율성을 보장하고 있다.**

*자체평가연구보고서 작성 가이드*

가이드-1. 의과대학은 외부로부터의 자율성이 보장되고 있다.
가이드-2. 교육과정의 설계에 있어 교수 혹은 교육과 연구지원 직원과 행정직이 책임을 감당하는 정책을 수립하고 실행하기 위한 자율성이 있다.
가이드-3. 교육과정 실행을 위해 인적, 물적 자원을 자율적으로 활용하고 독립성을 보장받을 수 있는 제도적 장치가 있다.

*방문평가단 평가 가이드*

| 기준 | 핵심어 | 가이드 | 근거자료 |
|---|---|---|---|
| K.1.2.1 | • 교수<br>• 교직원<br>• 교육과정의 설계와 실행<br>• 자원 | • 의과대학이 외부로부터의 자율성이 보장되고 있는지 확인한다.<br>• 교육과정의 설계에 있어 교수 혹은 교육과 연구지원 직원과 행정직이 책임을 감당하는 정책을 수립하고 실행하기 위한 자율성이 있는지 확인한다.<br>• 교육과정 실행을 위해 인적, 물적 자원을 자율적으로 활용하고 독립성을 보장받을 수 있는 제도적 장치가 있는지 확인한다. | • 교육과정 원칙 개발 자료<br>• 교육과정 관련 규정 |

**[주]**

- 대학의 자율성은 교육과정의 설계(평가부문 2.1, 2.6 참조), 평가(평가부문 3.1 참조), 학생 입학 (평가부문 4.1, 4.2 참조), 직원모집/선발(평가부문 5.1 참조)과 고용조건, 연구(평가부문 6.4 참조), 자원 할당(평가부문 8.3 참조)과 같은 핵심 분야에 관해 의사결정을 할 수 있도록 정부와 다른 단체(지역 당국, 종교단체, 민간 협동조합, 전문가 집단, 노동조합, 기타 이익공동체)로부터 적절한 독립성을 포함한다.
- 학문의 자유는 교직원과 학생들의 표현의 자유, 탐구의 자유, 출판의 자유를 포함한다.
- 대학의 자율성은 실제 교육과정 운영 시 교직원과 학생들이 서로 다른 관점을 가지고 임상의학 및 기초의학 문제를 설명하고 분석하는 것을 허용한다.

## 1.3 교육성과

### 기본기준

**K.1.3.1 의과대학은 사명에 근거하여 졸업성과를 규정하고 있다.**

*자체평가연구보고서 작성 가이드*

가이드-1. 사명에 근거한 졸업성과의 개발 절차가 적절하다.
가이드-2. 졸업성과에 기본수준 단계의 지식, 술기, 태도에 대한 역량을 규정하고 있다.
가이드-3. 졸업성과에는 다양한 의학 분야의 활동을 위한 적절한 역량, 보건의료 분야에서의 미래의 역할, 지속적인 졸업 후 교육, 지역사회의 건강 요구, 보건의료 전달체계 요구, 사회적 책무에 대한 다양한 측면을 규정하고 있다.

*방문평가단 평가 가이드*

| 기준 | 핵심어 | 가이드 | 근거자료 |
|---|---|---|---|
| K.1.3.1 | • 사명<br>• 졸업성과 | • 사명에 근거한 졸업성과의 개발 절차가 적절한지 확인한다.<br>• 졸업성과에 기본수준 단계의 지식, 술기, 태도에 대한 역량을 규정하고 있는지 확인한다.<br>• 졸업성과에 다양한 의학분야에서의 활동을 위한 적절한 역량, 보건의료 분야에서의 미래의 역할, 지속적인 졸업 후 교육, 지역사회의 건강 요구, 보건의료 전달체계 요구, 사회적 책무에 대한 다양한 측면을 규정하고 있는지 확인한다. | • 사명<br>• 졸업성과 |

**[주]**

- 졸업성과 혹은 역량이란 학생이 학습 종료 시점에 보여줄 것으로 기대되는 지식, 술기, 태도를 기술한 것을 의미한다.
  의과대학에서 명시한 의학과 의료행위 영역에서의 성과에는 (1) 기초의학분야, (2) 공중보건과 인구의학을 포함한 행동과학과 사회과학, (3) 의료행위와 관련된 의료윤리, 인간의 권리와 의료법규, (4) 진단과정, 치료과정, 의사소통능력, 질병의 치료와 예방, 건강증진, 재활, 임상 추론과 문제해결의 관점에서 임상 술기를 포함한 임상의학분야, (5) 평생학습 수행 능력과 의사로서의 다양한 역할과 관련한 의료전문성과 관계된 전문직업성을 보여줄 수 있는 능력 등을 포함한다.
  학생이 졸업 시 갖추어야 될 특성과 성과는 (1) 학자와 과학자, (2) 진료의사, (3) 커뮤니케이터, (4) 교육자, (5) 관리자, (6) 전문직업인 등으로서의 의사라는 용어로 그 범주를 나눌 수 있다.
- 졸업성과는 '2014 한국의 의사상'과 '기본의학교육 졸업성과(한국의과대학·의학전문대학원협회, 2017)'를 참조할 수 있다.

기본기준

**K.1.3.2 의과대학은 학생이 동료 학생, 교수진, 보건의료 인력, 환자와 보호자를 존중하고 이에 따라 적절하게 행동할 수 있도록 명시하고 있다.**

| *자체평가연구보고서 작성 가이드* |
|---|
| 가이드-1. 학생이 동료 학생, 교수진, 기타 보건의료 인력, 환자와 보호자를 존중하고 적절하게 행동할 수 있도록 명시한다. |

*방문평가단 평가 가이드*

| 기준 | 핵심어 | 가이드 | 근거자료 |
|---|---|---|---|
| K.1.3.2 | • 학생 행동 규정 | • 학생이 동료 학생, 교수진, 기타 보건의료 인력, 환자와 보호자를 존중하고 적절하게 행동할 수 있도록 명시되어 있는지 확인한다. | • 관련 규정 또는 자료 |

기본기준

**K.1.3.3 의과대학은 의도한 교육성과를 공개하고 있다.**

| *자체평가연구보고서 작성 가이드* |
|---|
| 가이드-1. 의도한 교육성과를 공개하고 있는지 확인한다. |

*방문평가단 평가 가이드*

| 기준 | 핵심어 | 가이드 | 근거자료 |
|---|---|---|---|
| K.1.3.3 | • 의도한 교육성과 | • 의도한 교육성과를 공개하고 있는지 확인한다. | • 교육성과 |

**[주]**

- 의도한 교육성과는 졸업성과의 하위 개념이며, 각 교육과정 또는 교과과정의 성과를 말한다.

## 우수기준

**H.1.3.1 의과대학은 졸업성과와 졸업 후 교육성과 간의 연계, 의학연구에 있어 학생 참여, 국제보건의료와 관련된 성과를 규정하고 있다.**

*자체평가연구보고서 작성 가이드*

가이드-1. 졸업성과와 졸업 후 교육성과와의 연계를 규정하고 있다.
가이드-2. 졸업성과는 의학 연구에 있어 학생 참여를 규정하고 있다
가이드-3. 졸업성과는 국제보건의료와 관련된 성과를 규정하고 있다.

*방문평가단 평가 가이드*

| 기준 | 핵심어 | 가이드 | 근거자료 |
|---|---|---|---|
| H.1.3.1 | • 졸업성과<br>• 졸업 후 교육성과<br>• 학생 연구 참여<br>• 국제보건의료 | • 졸업성과와 졸업 후 교육성과와의 연계를 규정하고 있는지 확인한다.<br>• 졸업성과는 의학 연구에 있어 학생 참여를 규정하고 있는지 확인한다.<br>• 졸업성과는 국제보건의료와 관련된 성과에 관심을 갖도록 규정하고 있는지 확인한다. | • 졸업성과<br>• 졸업 후 교육성과 |

## 1.4 사명과 교육성과 수립

### 기본기준

**K.1.4.1 의과대학은 주요 이해관계자를 대학의 사명과 졸업성과의 수립에 참여하도록 하고 있다.**

| *자체평가연구보고서 작성 가이드* |
|---|
| 가이드-1. 사명과 졸업성과 수립에 주요 이해관계자가 참여한다. |

*방문평가단 평가 가이드*

| 기준 | 핵심어 | 가이드 | 근거자료 |
|---|---|---|---|
| K.1.4.1 | • 주요 이해관계자<br>• 대학의 사명<br>• 졸업성과 | • 사명과 졸업성과 수립에 주요 이해관계자가 참여하고 있는지 확인한다. | • 사명<br>• 사명과 졸업성과 수립과정 근거서류 |

**[주]**

- 주요 이해관계자에는 학장, 보직자, 교수, 교육과정 관련 위원회, 학생 대표, 대학 본부, 관련 정부 기관(예: 국가 또는 해당지역 교육 혹은 보건복지 부서) 등이 포함될 수 있다.

### 우수기준

**H.1.4.1 의과대학은 기타 이해관계자를 대학의 사명과 졸업성과의 수립에 참여하도록 하고 있다.**

| *자체평가연구보고서 작성 가이드* |
|---|
| 가이드-1. 사명과 졸업성과 수립에 기타 이해관계자가 참여한다. |

*방문평가단 평가 가이드*

| 기준 | 핵심어 | 가이드 | 근거자료 |
|---|---|---|---|
| H.1.4.1 | • 기타 이해관계자<br>• 대학의 사명<br>• 졸업성과 | • 사명과 졸업성과 수립에 기타 이해관계자가 참여하고 있는지 확인한다. | • 사명<br>• 사명과 졸업성과 수립과정 근거서류 |

**[주]**

- 기타 이해관계자에는 직원 대표, 지역사회와 공공(예: 환자 단체를 포함한 보건의료 전달체계 이용자 등) 대표, 보건의료 당국(예: 지역 보건소 등), 유관 단체(예: 국민건강보험관리공단, 건강보험심사평가원 등), 의학 기관(예: 지역 의사회 등)과 대학원 교육자들을 포함한다.

ASK2019

02

# 교육과정

## 2.1 교육과정

### 기본기준

**K.2.1.1 의과대학은 교육과정의 원리와 원칙에 근거한 전체 교육과정 체계를 가지고 있다.**

| *자체평가연구보고서 작성 가이드* |
|---|
| 가이드-1. 의과대학이 자체적으로 개발한 교육과정의 원리와 원칙이 있다.<br>가이드-2. 의과대학의 전체 교육과정 체계가 있다.<br>가이드-3. 전체 교육과정이 교육과정의 원리와 원칙에 근거하고 있다. |

*방문평가단 평가 가이드*

| 기준 | 핵심어 | 가이드 | 근거자료 |
|---|---|---|---|
| K.2.1.1 | • 교육과정의 원리와 원칙<br>• 전체 교육과정 체계 | • 의과대학이 자체적으로 개발한 교육과정의 원리와 원칙을 확인한다.<br>• 의과대학의 전체 교육과정 체계를 점검한다.<br>• 전체 교육과정이 교육과정의 원리와 원칙에 근거하고 있는지 확인한다. | • 교육과정 개발 관련 자료 |

**[주]**

- 전체 교육과정은 의도한 교육성과, 교육내용/교수요목(syllabus), 프로그램의 경험과 과정을 포함하며, 계획된 교육과 학습 방법, 평가 방법의 구조가 설명되어 있어야 한다. 교육과정에는 학생이 성취할 지식, 술기, 태도를 제시해야 한다.
- 교육과정의 원리와 원칙에는 교육과정의 기본방향/전략, 교육과정 설계원칙, 교육내용 편성원칙, 교수학습법, 교육평가, 교육지원체제 등의 주요 내용을 요약하여 기술한다.
- 교육과정에는 학과, 장기계통, 임상 문제/직무 또는 질병 양상(disease pattern)에 기반을 둘뿐만 아니라 모듈식(modular) 혹은 나선형(spiral) 설계를 포함한다.
- 교육과정은 최근의 학습 원칙에 기반을 둔다.

## 기본기준

### K.2.1.2 의과대학은 학생들이 능동적으로 학습에 참여할 수 있도록 교육과정과 다양한 교수학습방법을 운영하고 있다.

| *자체평가연구보고서 작성 가이드* |
|---|
| 가이드-1. 능동적인 학습 참여가 가능한 교육과정을 가지고 있다.<br>가이드-2. 능동적인 학습 참여를 지원하는 다양한 교수학습방법을 사용하고 있다.<br>가이드-3. 능동적인 학습 참여를 수행한 실적 자료가 있다. |

*방문평가단 평가 가이드*

| 기준 | 핵심어 | 가이드 | 근거자료 |
|---|---|---|---|
| K.2.1.2 | • 능동적인 학습 참여<br>• 다양한 교수학습 방법 | • 능동적인 학습 참여가 가능한 교육과정을 가지고 있는지 확인한다.<br>• 능동적인 학습 참여를 지원하는 다양한 교수학습방법을 사용하고 있는지 확인한다.<br>• 능동적인 학습 참여를 수행한 실적 자료를 확인한다. | • 능동적인 학습 참여 관련 자료<br>• 능동적인 학습 참여 운영실적 |

**[주]**

- 교수학습방법에는 강의, 소규모 교수법, 문제 중심 또는 사례 중심 학습, 팀바탕 학습(team-based learning), 상호협력 학습(peer assisted learning), 실습, 침상 옆 교육(bed-side teaching), 임상 시연, 임상 술기, 실험실 훈련, 지역 사회 내 현장 실습과 웹 기반 수업을 포함한다.

## 기본기준

### K.2.1.3 의과대학은 평등 원칙에 따라 교육과정을 수행하고 있다.

| *자체평가연구보고서 작성 가이드* |
|---|
| 가이드-1. 평등 원칙이 교육과정 운영규정에 반영되어 운영되고 있다. |

*방문평가단 평가 가이드*

| 기준 | 핵심어 | 가이드 | 근거자료 |
|---|---|---|---|
| K.2.1.3 | • 평등 원칙 | • 교육과정 운영규정에 평등 원칙을 제시하고 수행하고 있는지 확인한다. | • 관련 운영규정<br>• 관련 수행자료 |

**[주]**

- 평등 원칙은 성별, 인종, 종교, 성적 취향, 사회 경제적 지위와 상관없이 신체적 능력(장애인 배려 등)을 고려하여 교직원과 학생들을 평등하게 대하는 것을 의미한다.

## 우수기준

### H.2.1.1 의과대학은 학생들이 평생학습을 준비할 수 있도록 교육과정을 운영하고 있다.

| *자체평가연구보고서 작성 가이드* |
|---|
| 가이드-1. 학생들이 평생학습을 준비하는 교육과정을 운영하고 있다.<br>가이드-2. 평생학습 준비에 대한 실적이 있다. |

*방문평가단 평가 가이드*

| 기준 | 핵심어 | 가이드 | 근거자료 |
|---|---|---|---|
| H.2.1.1 | • 평생학습 | • 학생들이 평생학습을 준비하는 교육과정을 운영하고 있는지 확인한다.<br>• 평생학습 준비에 대한 실적이 있는지 확인한다. | • 졸업성과<br>• 평생학습 준비 관련 교육과정과 운영실적 |

**[주]**

- 평생학습은 K.1.1.2 참조.

## 2.2 과학적 방법

### 기본기준

**K.2.2.1 의과대학은 학생들이 분석과 비판적 사고를 포함하는 과학적 방법의 원칙을 학습할 수 있도록 하고 있다.**

| *자체평가연구보고서 작성 가이드* |
|---|
| 가이드-1. 교육과정에 분석과 비판적 사고를 포함한 과학적 방법의 원칙이 제시되어 있다.<br>가이드-2. 과학적 방법을 학습한 실적이 있다. |

*방문평가단 평가 가이드*

| 기준 | 핵심어 | 가이드 | 근거자료 |
|---|---|---|---|
| K.2.2.1 | • 분석과 비판적 사고<br>• 과학적 방법의 원칙 | • 교육과정에 분석과 비판적 사고를 포함한 과학적 방법의 원칙이 제시되어 있는 지 확인한다.<br>• 과학적 방법을 학습한 실적이 있는 지 확인한다. | • 과정에 대한 계획서<br>• 관련 실적 |

### 기본기준

**K.2.2.2 의과대학은 학생들이 의학연구 방법을 학습할 수 있도록 하고 있다.**

| *자체평가연구보고서 작성 가이드* |
|---|
| 가이드-1. 의학연구 방법을 가르치고 있다. |

*방문평가단 평가 가이드*

| 기준 | 핵심어 | 가이드 | 근거자료 |
|---|---|---|---|
| K.2.2.2 | • 의학연구 방법 | • 의학연구 방법을 가르치고 있는지 확인한다. | • 과정에 대한 계획서<br>• 관련 실적 |

## 기본기준

### K.2.2.3 의과대학은 학생들이 근거중심의학을 학습할 수 있도록 하고 있다.

| *자체평가연구보고서 작성 가이드* |
|---|
| 가이드-1. 근거중심의 사고와 의사결정역량을 학습하고 있다. |

*방문평가단 평가 가이드*

| 기준 | 핵심어 | 가이드 | 근거자료 |
|---|---|---|---|
| K.2.2.3 | • 근거중심의학 | • 근거중심의 사고와 의사결정역량을 학습하고 있는지 확인한다. | • 과정에 대한 계획서<br>• 관련 실적 |

**[주]**

- 근거중심의학은 문헌, 임상시험과 증명된 과학적 결과를 기반으로 한다.

## 2.3 기초의학

### 기본기준

**K.2.3.1 의과대학은 인체의 구조와 기능 이해에 필요한 과학적 지식, 개념과 원리를 학습할 수 있도록 기초의학 교육과정을 운영하고 있다.**

| *자체평가연구보고서 작성 가이드* |
|---|
| 가이드-1. 과학적 지식, 개념과 원리를 학습할 수 있는 기초의학 교육과정을 적절히 운영하고 있다. |

*방문평가단 평가 가이드*

| 기준 | 핵심어 | 가이드 | 근거자료 |
|---|---|---|---|
| K.2.3.1 | • 기초의학 교육과정 | • 과학적 지식, 개념과 원리를 학습할 수 있는 기초의학 교육과정을 적절히 운영하고 있는지 확인한다. | • 수업계획서<br>• 교육과정 운영지침 |

**[주]**

- 기초의학 교육내용에는 지역의 요구, 관심과 전통에 따라 면역학, 미생물학(기생충학, 바이러스학, 세균학 을 포함), 병리학, 생리학, 생물물리학, 생화학분자생물학, 세포생물학, 유전학, 약리학, 예방의학, 해부학 등을 포함한다.

### 기본기준

**K.2.3.2 의과대학은 기초의학에서 학습한 지식, 개념 및 원리가 임상의학의 학습에 연계될 수 있도록 기초의학 교육과정을 운영하고 있다.**

| *자체평가연구보고서 작성 가이드* |
|---|
| 가이드-1. 임상의학 학습에 연계된 과학적 지식과 연계된 기초의학 교육과정을 운영하고 있다. |

*방문평가단 평가 가이드*

| 기준 | 핵심어 | 가이드 | 근거자료 |
|---|---|---|---|
| K.2.3.2 | • 임상의학 학습에 연계된 과학적 지식 | • 임상의학 학습에 연계된 과학적 지식과 연계된 기초의학 교육과정을 운영하고 있는지 확인한다. | • 과정 계획서 |

## 우수기준

**H.2.3.1 의과대학은 현재와 미래의 의료 환경 변화에 맞추어 기초의학 교육과정을 수정보완하고 있다.**

| *자체평가연구보고서 작성 가이드* |
|---|
| 가이드-1. 현재와 미래의 의료 환경 변화에 맞추어 기초의학 교육과정을 수정보완하고 있다. |

*방문평가단 평가 가이드*

| 기준 | 핵심어 | 가이드 | 근거자료 |
|---|---|---|---|
| H.2.3.1 | • 의료 환경 변화에 따른 기초의학교육과정 수정보완 | • 현재와 미래의 의료 환경 변화에 맞추어 기초의학 교육과정을 수정보완하고 있는지 확인한다. | • 과정계획서<br>• 관련 실적자료 |

## 2.4 의료인문학

### 기본기준

**K.2.4.1 의과대학은 의료인문학 교육과정을 적절하게 운영하고 있다.**

| *자체평가연구보고서 작성 가이드* |
|---|
| 가이드-1. 의료인문학, 행동과학, 사회과학, 의료윤리, 의료법규 등의 교육과정을 적절하게 운영하고 있다. |

*방문평가단 평가 가이드*

| 기준 | 핵심어 | 가이드 | 근거자료 |
|---|---|---|---|
| K.2.4.1 | • 의료인문학<br>• 행동과학<br>• 사회과학<br>• 의료윤리<br>• 의료법규 | • 의료인문학, 행동과학, 사회과학, 의료윤리, 의료법규 등의 교육과정을 적절하게 운영하고 있는지 확인한다. | • 단계별 교육과정표<br>• 관련 교과목 수업계획서 |

**[주]**

- 행동과학과 사회과학에는 지역의 요구, 관심과 전통에 따라 공중보건의학, 사회의학, 생물통계학, 국제보건의료, 사회역학, 의료사회학, 의료심리학, 의료인류학, 위생학, 지역사회의학을 포함한다.
- 의료윤리는 의사의 행동과 의사 결정에 관련된 가치관, 권리, 책임과 같은 의료 행위에서의 도덕적 문제를 다룬다.
- 의료법규는 약학 기술과 의료 기술(기기, 장비 등)의 생산과 이용에 대한 규정을 포함한 보건의료 서비스 전달 체계와 의료 전문직과 의료행위에 대한 법규와 기타 규정을 다룬다.
- 행동과학, 사회과학, 의료윤리, 의료법규는 건강 문제의 원인, 분포, 결과의 사회경제적, 인구학적, 문화적 결정 요인, 국가 보건의료 체계와 환자의 권리에 관한 지식을 이해하기 위해 필요한 지식, 개념, 방법, 술기와 태도를 포함한다. 이로써 지역사회와 사회의 건강 요구를 분석할 수 있으며, 효과적인 의사소통, 임상 의사결정, 윤리적 진료가 가능하게 한다.

우수기준

**H.2.4.1 의과대학은 현재와 미래의 의료 환경 변화에 맞추어 의료인문학의 내용을 수정보완하고 있다.**

| *자체평가연구보고서 작성 가이드* |
| --- |
| 가이드-1. 현재와 미래의 의료 환경 변화에 맞추어 행동과학, 사회과학, 의료윤리, 의료법규의 내용을 수정보완하고 있다. |

*방문평가단 평가 가이드*

| 기준 | 핵심어 | 가이드 | 근거자료 |
| --- | --- | --- | --- |
| H.2.4.1 | • 의료 환경 변화에 따른 의료인문학 교육과정 수정보완 | • 현재와 미래의 의료 환경 변화에 맞추어 행동과학, 사회과학, 의료윤리, 의료법규의 내용을 수정보완하고 있는지 확인한다. | • 현재와 미래의 의료 환경 변화 분석 자료<br>• 과정에 대한 계획서<br>• 교육관련 위원회의 회의자료 |

## 2.5 임상의학과 술기

### 기본기준

**K.2.5.1 의과대학은 졸업 후 진료가 가능하도록 임상의학과 임상실습 교육과정을 운영하고 있다.**

| *자체평가연구보고서 작성 가이드* |
|---|
| 가이드-1. 졸업 후 일차 진료가 가능한 임상의학과 관련된 교육과정을 가지고 있다.<br>가이드-2. 학생이 습득하여야 하는 기본 술기가 포함된 임상실습지침서가 학생들에게 제공 및 활용되고 있다.<br>가이드-3. 임상실습은 단순 관찰과 같은 수동적인 방법 이외에 의료진의 일부로 실제 진료에 참여하는 등 의사의 업무를 익힐 수 있도록 구성되어 있다.<br>가이드-4. 환자진료 참여를 모니터링하고 피드백하는 교육과정을 운영하고 있다.<br>가이드-5. 임상실습이 다양한 1, 2차 병원에서 이루어지고 있다. |

*방문평가단 평가 가이드*

| 기준 | 핵심어 | 가이드 | 근거자료 |
|---|---|---|---|
| K.2.5.1 | • 졸업 후 진료<br>• 일차진료 | • 졸업 후 일차 진료가 가능한 임상의학과 관련된 교육과정을 가지고 있는지 확인한다.<br>• 학생이 습득하여야 하는 기본 술기가 포함된 임상실습지침서가 학생들에게 제공 및 활용되고 있는지 확인한다.<br>• 임상실습은 단순 관찰과 같은 수동적인 방법 이외에 의료진의 일부로 실제 진료에 참여하는 등 의사의 업무를 익힐 수 있도록 구성되어 있는지 확인한다.<br>• 환자진료 참여를 모니터링하고 피드백하는 교육과정을 운영하고 있는 지 확인한다.<br>• 임상실습이 다양한 1, 2차 병원에서 이루어지고 있는 지 확인한다. | • 관련 수업계획서<br>• 임상실습지침서<br>• 환자진료 참여 피드백 관련 자료 |

**[주]**

- 임상의학 교육내용에는 지역의 요구, 관심과 전통에 따른 임상 각 과목을 포함한다.
- 임상술기에는 병력청취, 신체검사, 의사소통 기술, 수행, 관찰, 응급 진료, 처방, 치료 행위를 포함한다.
- 기본 술기란 활력징후 측정, 정맥 채혈, 혈액도말 검사, 심전도, 정맥 및 근육 주사, 요로 도자, 관장, 환부 소독 및 봉합, 비위관 삽입 등과 같은 일차 진료 수준의 진단 술기, 치료 술기 및 환자안전 관련 술기를 말하며, 이는 대학에 따라서 차이가 있을 수 있다.

**기본기준**

### K.2.5.2 의과대학은 교육과정에 임상실습 전 준비교육을 운영하고 있다.

| *자체평가연구보고서 작성 가이드* |
|---|
| 가이드-1. 임상실습 전 준비교육이 포함된 교육과정이 있다. |

*방문평가단 평가 가이드*

| 기준 | 핵심어 | 가이드 | 근거자료 |
|---|---|---|---|
| K.2.5.2 | • 임상실습 전 준비교육 | • 임상실습 전 준비교육이 포함된 교육과정이 있는지 확인한다.<br>• 환자면담 기법과 기본 술기 교육이 포함되어 있는지 확인한다.<br>• 감염 및 환경 위험 예방과 대처 방법 교육이 포함되어 있는지 확인한다. | • 관련 수업계획서<br>• 수업시간 편성표 |

**[주]**

- 임상실습 전 준비교육은 Introduction to Clinical Medicine(ICM), Fundamentals of Clinical Medicine(FCM) 등을 의미한다. 블록 또는 지속적인 과정이 있으며, 학생 1인당 교육받는 시간이 40시간 이상이어야 한다.

**기본기준**

### K.2.5.3 의과대학은 적절한 임상실습 교육기간을 운영하고 있다.

| *자체평가연구보고서 작성 가이드* |
|---|
| 가이드-1. 임상실습 기간이 적절하다.<br>가이드-2. 학생이 자유롭게 선택할 수 있는 임상실습 과정이 있다. |

*방문평가단 평가 가이드*

| 기준 | 핵심어 | 가이드 | 근거자료 |
|---|---|---|---|
| K.2.5.3 | • 임상실습 교육기간 | • 임상실습 기간이 적절한지 확인한다.<br>• 자유선택 임상실습 과정이 적절한지 확인한다. | • 임상실습지침서<br>• 임상실습 편성표 |

**[주]**

- 임상실습 기간은 주요 임상학과 실습을 포함하여 최소 52주, 주당 36시간 이상이어야 한다.
- 주요 임상학과에는 내과(세부전공분야 포함), 외과(세부전공분야 포함), 산부인과, 소아청소년과, 정신건강의학과, 가정의학과, 응급의학을 포함한다.
- 자유선택 임상실습 기간은 최소 2주 이상이어야 한다.

## 기본기준

### K.2.5.4 의과대학은 환자안전을 강조한 임상실습 교육과정을 운영하고 있다.

| *자체평가연구보고서 작성 가이드* |
|---|
| 가이드-1. 환자안전을 강조한 교육을 하고 있다. |

*방문평가단 평가 가이드*

| 기준 | 핵심어 | 가이드 | 근거자료 |
|---|---|---|---|
| K.2.5.4 | • 환자안전 | • 환자안전을 강조한 교육을 하고 있는지 확인한다. | • 환자안전교육 관련 내용<br>• 임상실습지침서 |

**[주]**

- 환자안전을 강조한 임상실습 교육과정은 학생의 임상실습 활동에서 환자안전에 대해 지도 감독하는 것을 포함한다.

## 우수기준

### H.2.5.1 의과대학은 현재와 미래의 의료 환경 변화를 고려하여 임상의학 교육과정을 수정보완하고 있다.

| *자체평가연구보고서 작성 가이드* |
|---|
| 가이드-1. 의료 환경 변화에 따라 임상의학 교육과정을 수정보완한 실적이 있다. |

*방문평가단 평가 가이드*

| 기준 | 핵심어 | 가이드 | 근거자료 |
|---|---|---|---|
| H.2.5.1 | • 의료 환경 변화에 따른 임상의학 교육과정 수정보완 | • 의료 환경 변화에 따라 임상의학 교육과정을 수정보완한 실적이 있는지 확인한다. | • 현재와 미래의 의료 환경 변화 분석 자료<br>• 관련 자료(교육관련 위원회 회의자료, 교과목 회의자료 등) |

## 우수기준

**H.2.5.2 의과대학은 모든 학생이 환자 돌봄 참여를 포함하여 조기에 임상상황을 접할 수 있도록 하고, 학습 프로그램 단계에 따라 다양한 임상술기를 교육하고 있다.**

| *자체평가연구보고서 작성 가이드* |
|---|
| 가이드-1. 조기임상노출을 위한 교육과정을 운영하고 있다.<br>가이드-2. 환자 돌봄 참여를 포함한 교육과정을 운영하고 있다.<br>가이드-3. 학습 프로그램 단계에 따른 다양한 임상술기를 교육하고 있다. |

*방문평가단 평가 가이드*

| 기준 | 핵심어 | 가이드 | 근거자료 |
|---|---|---|---|
| H.2.5.2 | • 조기임상노출<br>• 환자 돌봄 참여<br>• 다양한 임상술기 교육 | • 조기임상노출을 위한 교육과정을 운영하고 있는지 확인한다.<br>• 환자 돌봄 참여를 포함한 교육과정을 운영하고 있는지 확인한다.<br>• 학습 프로그램 단계에 따른 다양한 임상술기를 교육하고 있는지 확인한다. | • 전체 교육과정<br>• 관련 자료 |

**[주]**

- 조기임상노출은 일차 진료상황에서 일부 이루어지며, 주로 병력청취, 신체진찰과 의사소통을 포함한다.
- 학생의 환자 돌봄 참여는 감독 하에서 검사, 환자치료 일부에 대한 책임을 감당하는 것을 포함하며, 이는 해당 지역사회 시설에서 이루어질 수 있다

## 우수기준

**H.2.5.3 의과대학은 임상실습교육에 참여하는 전임교원 외 의사에게 학생의 지도와 피드백에 관련한 교육과 훈련을 실시하고 있다.**

*자체평가연구보고서 작성 가이드*

가이드-1. 임상실습교육을 담당하는 전임교원 외 의사에 대한 정기적인 교육과 훈련을 실시하고 있다.
가이드-2. 외부 파견교육기관의 경우, 전임교원 외 의사에 대해 임상실습 교육을 실시하거나 교육에 대한 지침을 제공하고 있다.

*방문평가단 평가 가이드*

| 기준 | 핵심어 | 가이드 | 근거자료 |
|---|---|---|---|
| H.2.5.3 | • 교육담당 전임교원 외 의사 교육 | • 임상실습교육을 담당하는 전임교원 외 의사에 대한 정기적인 교육과 훈련을 실시하고 있는지 확인한다.<br>• 외부 파견교육기관의 경우, 전임교원 외 의사에 대해 임상실습 교육을 실시하거나 교육에 대한 지침을 제공하고 있는 지 확인한다. | • 관련 규정<br>• 관련 실적 |

## 2.6 교육과정의 구조, 구성, 기간

### 기본기준

**K.2.6.1 의과대학은 기초의학, 의료인문학, 임상의학 간에 적절한 조화를 이룬 교육과정이 있다.**

| *자체평가연구보고서 작성 가이드* |
|---|
| 가이드-1. 기초의학, 의료인문학, 임상의학 간의 적절한 조화(coordination)를 이루고 있다.<br>가이드-2. 교육과정에는 교육내용, 범위, 순서, 기간과 기타 과정 요소가 명시되어 있다. |

*방문평가단 평가 가이드*

| 기준 | 핵심어 | 가이드 | 근거자료 |
|---|---|---|---|
| K.2.6.1 | • 조화를 이룬 교육과정 | • 기초의학, 의료인문학, 임상의학 간의 적절한 조화(coordination)를 이루고 있는지 확인한다.<br>• 교육과정에 교육내용, 범위, 순서, 기간과 기타 과정 요소가 명시되어 있는지 확인한다. | • 교육과정 계획서<br>• 관련 규정 |

## 기본기준

### K.2.6.2 의과대학은 관련 학문, 학과, 과정이 수평 통합된 교육과정을 운영하고 있다.

| *자체평가연구보고서 작성 가이드* |
|---|
| 가이드-1. 관련 학문, 학과, 과정이 수평 통합(integration)된 교육과정을 운영하고 있다.<br>가이드-2. 통합을 위한 지원제계와 통합교육 책임교수가 지정되어 있다. |

*방문평가단 평가 가이드*

| 기준 | 핵심어 | 가이드 | 근거자료 |
|---|---|---|---|
| K.2.6.2 | • 수평통합 | • 관련 학문, 학과, 과정이 수평 통합(integration)된 교육과정을 운영하고 있는지 확인한다.<br>• 통합을 위한 지원제계와 통합교육 책임교수가 지정되어 있는지 확인한다. | • 교육과정 운영 지침<br>• 기초의학, 임상의학, 의료인문학 교육과정이 연계(수평통합) 되어 있는 교육과정<br>• 조직, 인력, 예산 자료<br>• 통합과정별 임명된 책임교수 명단<br>• 책임교수의 권한과 책임이 명시된 규정 |

**[주]**

- 수평통합의 예는 해부학, 생화학, 생리학과 같은 기초의학 과정 간을 통합하거나 임상의학 과목간의 통합이다.

## 우수기준

### H.2.6.1 의과대학은 임상의학과 기초의학, 의료인문학이 수직 통합된 교육과정을 운영하고 있다.

| *자체평가연구보고서 작성 가이드* |
|---|
| 가이드-1. 수직 통합(integration)된 교육과정을 운영하고 있다. |

*방문평가단 평가 가이드*

| 기준 | 핵심어 | 가이드 | 근거자료 |
|---|---|---|---|
| H.2.6.1 | • 수직통합 | • 수직 통합(integration)된 교육과정을 운영하고 있는지 확인한다. | • 교육과정 운영지침<br>• 기초의학, 임상의학, 의료인문학 교육과정이 연계(수직통합)되어 있는 교육과정<br>• 관련 자료 |

## 우수기준

### H.2.6.2 의과대학은 교육과정에 선택과목을 운영하고 필수과목과 선택과목 간의 균형을 적절하게 유지하고 있다.

| *자체평가연구보고서 작성 가이드* |
|---|
| 가이드-1. 선택과목을 운영하고 있다.<br>가이드-2. 필수과목과 선택과목이 적절히 균형을 유지하고 있다. |

*방문평가단 평가 가이드*

| 기준 | 핵심어 | 가이드 | 근거자료 |
|---|---|---|---|
| H.2.6.2 | • 선택과목<br>• 과목 간의 균형 | • 선택과목을 운영하고 있는지 확인한다.<br>• 필수과목과 선택과목이 적절히 균형을 유지하고 있는지 확인한다. | • 교육과정 운영지침 |

## 2.7 교육과정 관리

### 기본기준

**K.2.7.1 의과대학은 교육과정위원회를 운영하고 있고, 교육과정위원회는 의도한 교육성과를 달성하기 위해 교육과정을 계획하고 실행하기 위한 책임과 권한을 가지고 있다.**

*자체평가연구보고서 작성 가이드*

가이드-1. 교육과정위원회를 운영하고 있다.
가이드-2. 교육과정위원회의 책임과 권한을 명시한 관련 규정이 있다.

*방문평가단 평가 가이드*

| 기준 | 핵심어 | 가이드 | 근거자료 |
|---|---|---|---|
| K.2.7.1 | • 교육과정위원회<br>• 책임과 권한 | • 교육과정위원회를 운영하고 있는지 확인한다.<br>• 교육과정위원회의 책임과 권한을 명시한 관련 규정이 있는지 확인한다. | • 관련 규정<br>• 위원회 회의자료 |

**[주]**

- 교육과정위원회는 대학과 정부의 규정 내에서 특정 학과와 과목간의 이해관계와 무관하게 교육과정을 관리 운영할 수 있는 권한을 가진다. 교육과정위원회는 교수학습방법, 학생평가와 과정평가를 계획하고 실행하기 위해 주어진 자원을 분배한다(K.8.3.2 참조).
- 의도한 교육성과는 K.1.3.3 참조.

### 기본기준

**K.2.7.2 의과대학은 교육과정위원회에 교직원과 학생대표를 참여시키고 있다.**

*자체평가연구보고서 작성 가이드*

가이드-1. 교육과정위원회에 교직원과 학생대표가 참여하고 있다.

*방문평가단 평가 가이드*

| 기준 | 핵심어 | 가이드 | 근거자료 |
|---|---|---|---|
| K.2.7.2 | • 교육과정위원회<br>• 교직원과 학생 참여 | • 교육과정위원회에 교직원과 학생대표가 참여하고 있는지 확인한다. | • 관련 규정<br>• 교직원, 학생 의견수렴, 반영 근거자료 |

## 2.8 의료행위와 보건의료분야의 연계

### 기본기준

**K.2.8.1 의과대학은 졸업 후 교육을 감안한 교육과정을 갖추고 있다.**

| *자체평가연구보고서 작성 가이드* |
|---|
| 가이드-1. 졸업 후 교육 단계에 연계되는 교육과정을 운영하고 있다. |

*방문평가단 평가 가이드*

| 기준 | 핵심어 | 가이드 | 근거자료 |
|---|---|---|---|
| K.2.8.1 | • 졸업 후 교육단계 | • 졸업 후 교육 단계에 연계되는 교육과정을 운영하고 있는지 확인한다. | • 교육과정 |

**[주]**

- 연계는 보건의료 문제를 파악하고 이와 연관되는 교육성과를 의미한다. 적절한 연계를 위해서는 지역, 국가, 세계적 상황을 고려하고 교육과정과 졸업 후 교육단계 및 의료행위가 상호관계를 이루도록 한다. 여기에는 보건의료 분야와의 상호작용, 보건의료 팀 활동에 교육자와 학생의 참여를 포함한다. 활발한 연계는 진로 지도를 위한 근간으로 졸업생들의 잠재적 고용주와의 소통을 의미한다.
- 졸업 후 교육에는 졸업 후 교육(PGME, 면허등록과 직업교육, 전문의과정 교육)과 전문직업성 평생개발/평생교육(CPD/CME)을 포함한다.

## 우수기준

**H.2.8.1 교육과정위원회는 졸업 후 예측되는 의료 환경의 변화와 지역사회의 의견을 고려하여 교육 프로그램을 수정보완하고 있다.**

| *자체평가연구보고서 작성 가이드* |
|---|
| 가이드-1. 졸업 후 예측되는 의료 환경의 변화를 고려하여 교육과정을 수정보완하고 있다.<br>가이드-2. 지역사회의 의견을 고려하여 교육과정을 수정보완하고 있다. |

*방문평가단 평가 가이드*

| 기준 | 핵심어 | 가이드 | 근거자료 |
|---|---|---|---|
| H.2.8.1 | • 의료 환경의 변화<br>• 지역사회 의견<br>• 교육과정의 수정보완 | • 졸업 후 예측되는 의료 환경의 변화를 고려하여 교육과정을 수정보완하고 있는 지 확인한다.<br>• 지역사회의 의견을 고려하여 교육과정을 수정보완하고 있는지 확인한다. | • 교육과정위원회 규정<br>• 교육과정위원회 회의자료 |

ASK2019

# 03 학생평가

## 3.1 평가방법

### 기본기준

**K.3.1.1 의과대학은 학생평가의 원칙, 방법을 규정하고 있다.**

| *자체평가연구보고서 작성 가이드* |
|---|
| 가이드-1. 학생평가의 원칙과 방법이 명시된 규정이 있다.<br>가이드-2. 학생평가의 원칙과 방법과 관련된 규정을 교직원과 학생들이 인지하고 있다. |

*방문평가단 평가 가이드*

| 기준 | 핵심어 | 가이드 | 근거자료 |
|---|---|---|---|
| K.3.1.1 | • 학생평가의 원칙<br>• 학생평가의 방법 | • 학생평가의 원칙, 방법을 명시하고 있다.<br>• 학생평가의 원칙, 방법과 관련된 규정을 교직원과 학생들이 인지하고 있는지 확인한다. | • 평가 관련 규정<br>• 홍보자료<br>• 교직원·학생 면담 |

**[주]**

- 평가방법은 형성평가와 총합평가간의 균형, 시험 횟수에 대한 고려, 시험방법의 균형(필기와 구두시험), 규준 참조 평가와 준거 참조 평가 활용, 개인포트폴리오와 로그북(log-books)의 활용, 특별한 형태의 시험, 예를 들어 객관적 구조화 임상 평가(Objective Structured Clinical Examinations; OSCE) 혹은 짧은 임상수행평가(MiniCEX)의 활용을 포함한다. 또한 평가방법에는 표절방지 시스템도 포함할 수 있다.

## 기본기준

### K.3.1.2　의과대학은 학생평가에 지식, 술기, 태도 영역을 포함하고 있다.

*자체평가연구보고서 작성 가이드*

가이드-1. 학생평가는 성과에 바탕을 둔 지식, 술기, 태도 영역을 포함하고 있다.
가이드-2. 임상실습 교육과정에서 지식, 술기, 태도를 포함한 학생의 수행능력을 평가하고 있다.

*방문평가단 평가 가이드*

| 기준 | 핵심어 | 가이드 | 근거자료 |
|---|---|---|---|
| K.3.1.2 | • 학생평가 영역 | • 교육과정에서 의도한 성과를 확인할 수 있는 지식, 술기, 태도 영역을 포함하고 있는지 확인한다.<br>• 임상실습 교육과정에서 지식, 술기, 태도를 포함한 학생의 수행능력을 평가하고 있는지 확인한다. | • 지식, 술기, 태도 영역이 포함된 평가자료<br>• 임상실습과정에서 학생의 수행능력 평가자료 |

## 기본기준

### K.3.1.3　의과대학은 다양한 평가방법과 형식을 사용하여 학생평가를 하고 있다.

*자체평가연구보고서 작성 가이드*

가이드-1. 학생평가는 다양한 평가방법을 사용하고 있다.

*방문평가단 평가 가이드*

| 기준 | 핵심어 | 가이드 | 근거자료 |
|---|---|---|---|
| K.3.1.3 | • 다양한 평가 방법 | • 다양한 평가방법을 사용하여 학생평가를 하고 있는지 확인한다. | • 학생평가 자료 |

**[주]**

- 평가방법과 형식은 각 대학의 교육과정에 맞게 자체적으로 정하여 실시한다.

## 기본기준

### K.3.1.4 의과대학은 학생평가 결과에 이의를 제기할 수 있는 제도를 운영하고 있다.

*자체평가연구보고서 작성 가이드*

가이드-1. 학생들이 평가결과에 이의를 제기할 수 있는 제도를 운영하고 있다.
가이드-2. 성적, 유급, 졸업 유예와 퇴학 등의 결정에 대한 소명의 기회를 제공하는 제도를 운영하고 있다.

*방문평가단 평가 가이드*

| 기준 | 핵심어 | 가이드 | 근거자료 |
|---|---|---|---|
| K.3.1.4 | • 이의제기 제도<br>• 소명의 기회 제공 | • 학생들이 평가결과에 이의를 제기할 수 있는 규정과 절차가 있다.<br>• 성적, 유급, 졸업 유예와 퇴학 등의 결정에 대한 소명의 기회를 제공하는 관련 규정과 절차가 있다. | • 관련 규정<br>• 관련 위원회 구성과 활동자료<br>• 홍보자료 |

**[주]**

- 의과대학은 학생에게 학점이 확정되기 전 성적, 유급, 졸업유예, 퇴학 등의 결정에 대한 소명의 기회를 보장한다. 이때 이해상충이 있는 교수는 소명 과정에 참여할 수 없다.

## 우수기준

**H.3.1.1 의과대학은 학생평가 방법의 신뢰성과 타당성을 평가하고 새로운 평가방법을 적절하게 활용하며, 외부평가자를 활용하고 있다.**

| *자체평가연구보고서 작성 가이드* |
|---|
| 가이드-1. 학생평가 방법의 신뢰성과 타당성을 평가하고 이를 문서화한다.<br>가이드-2. 개선이 필요한 경우 새로운 평가방법을 도입하여 적용하고 있다.<br>가이드-3. 학생평가 방법을 평가하기 위해 외부평가자를 활용하고 있다. |

*방문평가단 평가 가이드*

| 기준 | 핵심어 | 가이드 | 근거자료 |
|---|---|---|---|
| H.3.1.1 | • 학생평가 방법의 신뢰성과 타당성 평가<br>• 새로운 평가방법 활용<br>• 외부평가자 활용 | • 학생평가 방법의 신뢰성과 타당성을 평가하고 이를 문서화한 것을 확인한다.<br>• 새로운 평가방법을 적절하게 활용하고 있는지 확인한다.<br>• 학생평가 방법을 객관적으로 평가하기 위해 외부평가자를 활용하고 있는지 확인한다. | • 학생평가 방법 평가자료<br>• 새로운 평가방법 적용자료<br>• 외부평가자 활용자료 |

**[주]**

- 평가방법의 신뢰성과 타당성을 평가하고 문서화하는 것은 평가 실무의 적절한 질을 보장하는 과정으로 필요하다.
- 외부평가자의 활용은 평가의 공정성, 질, 투명성을 높일 수 있다.

## 3.2 평가와 학습의 관계

### 기본기준

**K.3.2.1 의과대학은 의도한 교육성과와 교육방법에 적합하게 학생평가를 하고 있다.**

| *자체평가연구보고서 작성 가이드* |
|---|
| 가이드-1. 의도한 교육성과와 교육방법에 적합하게 학생평가를 하고 있다. |

*방문평가단 평가 가이드*

| 기준 | 핵심어 | 가이드 | 근거자료 |
|---|---|---|---|
| K.3.2.1 | • 의도한 교육성과<br>• 교육방법 | • 의도한 교육성과와 교육방법에 적합하게 학생평가를 하고 있는지 확인한다. | • 수업계획서<br>• 평가 관련 실적 |

**[주]**

- 의도한 교육성과는 K.1.3.3 참조.

### 기본기준

**K.3.2.2 의과대학은 의도한 교육성과에 도달할 수 있도록 학생평가를 하고 있다.**

| *자체평가연구보고서 작성 가이드* |
|---|
| 가이드-1. 의도한 교육성과를 학생이 도달할 수 있도록 평가하고 있다.<br>가이드-2. 평가결과를 진급에 활용한다. |

*방문평가단 평가 가이드*

| 기준 | 핵심어 | 가이드 | 근거자료 |
|---|---|---|---|
| K.3.2.2 | • 의도한 교육성과 도달 | • 의도한 교육성과를 학생이 도달할 수 있도록 평가하고 있는지 확인한다.<br>• 평가결과를 진급에 활용하는지 확인한다. | • 평가 관련 자료<br>• 평가결과 활용 자료 |

## 기본기준

### K.3.2.3 의과대학은 학생의 학습을 증진시킬 수 있도록 학생평가를 하고 있다.

| *자체평가연구보고서 작성 가이드* |
|---|
| 가이드-1. 학생의 학습을 증진시킬 수 있도록 학생평가를 하고 있다. |

*방문평가단 평가 가이드*

| 기준 | 핵심어 | 가이드 | 근거자료 |
|---|---|---|---|
| K.3.2.3 | • 학습 증진 | • 학생의 학습을 증진시킬 수 있도록 학생평가를 하고 있는지 확인한다. | • 평가 관련 자료 |

## 기본기준

### K.3.2.4 의과대학은 형성평가와 총괄평가를 균형 있게 실시하고 있다.

| *자체평가연구보고서 작성 가이드* |
|---|
| 가이드-1. 형성평가와 총괄평가를 균형 있게 실시하고 있다. |

*방문평가단 평가 가이드*

| 기준 | 핵심어 | 가이드 | 근거자료 |
|---|---|---|---|
| K.3.2.4 | • 형성평가<br>• 총괄평가 | • 학업향상과 학습에 대한 가이드를 제공하기 위하여 형성평가와 총괄평가를 균형 있게 실시하고 있는지 확인한다. | • 학업향상정도에 대한 지침<br>• 학습지도 실적<br>• 형성평가, 총괄평가 자료 |

## 우수기준

**H.3.2.1 의과대학은 기본지식 습득과 통합학습 모두를 장려하기 위해 시험횟수와 유형을 조정하고 있다.**

| *자체평가연구보고서 작성 가이드* |
|---|
| 가이드-1. 기본지식 습득과 통합학습 모두를 장려하기 위한 합리적인 평가가 되도록 시험횟수와 유형을 조정한다. |

*방문평가단 평가 가이드*

| 기준 | 핵심어 | 가이드 | 근거자료 |
|---|---|---|---|
| H.3.2.1 | • 지식 습득<br>• 통합학습<br>• 시험횟수와 유형 조정 | • 기본지식 습득과 통합학습 모두를 장려하기 위해 교육과정 요소로써 시험횟수와 유형을 사전에 계획하고, 계획에 따라 운영하고 있는지 확인한다.<br>• 운영한 결과를 분석하여 시험횟수와 유형을 조정하는 활동을 하고 있는지 확인한다. | • 수업계획서<br>• 시험분석 결과<br>• 시험횟수와 유형 조정 활동 실적 |

**[주]**

- 기본지식(knowledge based) 습득은 일반적으로 기본의학교육 학습성과(과학적 개념과 원리중심, 진료역량중심, 사람과 사회중심 등)를 포함한 기본의학교육과정에서 필요한 지식습득을 의미한다.
- 시험횟수와 유형 조정은 학습에 부정적인 영향을 최소화하여야 하며, 과중한 교육과정과 과도한 양의 정보를 학생들이 배우도록 하는 요구가 되지 않도록 해야 한다.
- 통합학습(integrated learning) 장려는 개별과목 혹은 주제 분야에 대한 지식의 합리적 평가를 보장하면서 통합평가 시행을 고려하는 것을 포함한다.

## 우수기준

### H.3.2.2 의과대학은 평가결과를 근거로 학생에게 적절한 피드백을 하고 있다.

| *자체평가연구보고서 작성 가이드* |
|---|
| 가이드-1. 학생평가 결과를 근거로 학생에게 적절한 피드백을 제공한다. |

*방문평가단 평가 가이드*

| 기준 | 핵심어 | 가이드 | 근거자료 |
|---|---|---|---|
| H.3.2.2 | • 피드백 | • 평가결과에 대한 피드백을 다양한 방법으로 학생에게 제공하고 있는지 확인한다.<br>• 평가결과에 대하여 적절한 시기 안에 피드백을 제공하고 있는지 확인한다. | • 피드백방법 관련 자료<br>• 교수, 학생 면담 |

ASK2019

# 04 학생

## 4.1 입학정책과 선발

### 기본기준

**K.4.1.1 의과대학은 객관적인 원칙에 근거한 입학정책을 수립하고 시행하고 있다.**

*자체평가연구보고서 작성 가이드*

가이드-1. 학생선발 과정에서 객관성 원칙에 기초한 입학정책이 있다.
가이드-2. 명문화된 입학정책과 학생선발방법을 홍보하고, 학생 선발기준을 공개한다.
가이드-3. 학내외 전문가로 구성된 학생선발 관련 위원회가 전문적이고 지속적으로 운영한다.
가이드-4. 학생선발 관련 위원회가 선발기준과 절차를 검토한다.

*방문평가단 평가 가이드*

| 기준 | 핵심어 | 가이드 | 근거자료 |
|---|---|---|---|
| K.4.1.1 | • 입학정책<br>• 학생선발 관련 위원회 | • 학생선발 과정에서 객관성 원칙에 기초한 입학정책이 있는지 확인한다.<br>• 명문화된 입학정책과 학생선발방법을 홍보하고, 학생 선발기준을 공개하는지 확인한다.<br>• 학내외 전문가로 구성된 학생선발 관련 위원회가 전문적이고 지속적으로 운영되는지 확인한다.<br>• 학생선발 관련 위원회가 선발기준과 절차를 검토하는지 확인한다. | • 입학관련 규정<br>• 입학정책 관련자료<br>• 입학제도에 대한 학내외 검토자료<br>• 학생선발 관련 위원회 규정과 위원회 활동 실적<br>• 학생선발 관련 제반 서류<br>• 면접시험에 사용되는 양식 |

**[주]**

- 입학정책은 국가규정을 준수하고, 지역 상황에 적합해야 한다. 의과대학이 입학정책을 직접 관여하지 않을 경우, 의과대학은 입학생 수와 교육역량 간의 균형을 이룰 수 있도록 해야 한다.
- 학생선발 과정에 대한 기술에는 의사가 되려는 동기에 대한 평가를 포함하여 중고등학교 성적, 다른 관련 학업 혹은 교육 경험, 입학시험과 면접과 같은 선발 근거와 방법을 포함한다. 또한, 의료분야의 다양성을 고려하여 다양한 학생을 선발한다.

## 우수기준

**H.4.1.1 의과대학은 대학의 사명, 교육과정과 졸업생이 갖추어야 할 자질 등의 연계를 고려하여 학생선발을 하고 있다.**

*자체평가연구보고서 작성 가이드*

가이드-1. 대학의 사명, 교육과정과 졸업생이 갖추어야 할 자질 등과 연계된 학생선발 과정이 있다.
가이드-2. 인성평가가 적절히 이루어지고 있다.

*방문평가단 평가 가이드*

| 기준 | 핵심어 | 가이드 | 근거자료 |
|---|---|---|---|
| H.4.1.1 | • 학생선발<br>• 인성평가 | • 대학의 사명, 교육과정과 졸업생이 갖추어야 할 자질 등과 연계된 학생선발 과정이 있는지 확인한다.<br>• 전체 면접시간이 적절히 이루어지고 있는지 확인한다. | • 학생선발 관련 위원회 규정 및 회의자료<br>• 입학관련 규정<br>• 인성평가에 사용되는 양식 |

**[주]**

- 학생 1인당 면접시간은 최소 1시간 이상을 권장한다.

## 우수기준

**H.4.1.2 의과대학은 입학정책을 정기적으로 검토하고, 질적 향상을 위한 개선노력을 하고 있다.**

*자체평가연구보고서 작성 가이드*

가이드-1. 지역사회와 사회의 건강 요구에 따라 관련 사회적, 전문적 자료를 바탕으로 한 입학정책이 있고, 이를 정기적으로 검토하고 있다.
가이드-2. 입학 결정에 대해 이의를 제기할 수 있는 시스템을 갖추어 활용하고 있다.

*방문평가단 평가 가이드*

| 기준 | 핵심어 | 가이드 | 근거자료 |
|---|---|---|---|
| H.4.1.2 | • 입학정책의 정기적 검토<br>• 입학결정에 대한 이의제기 시스템 | • 지역사회와 사회의 건강 요구에 따라 관련 사회적, 전문적 자료를 바탕으로 한 입학 정책이 있고, 이를 정기적으로 검토하고 있는지 확인한다.<br>• 입학 결정에 대해 이의를 제기할 수 있는 시스템을 갖추고 있고 활용되고 있는 지 확인한다. | • 입학관련 위원회 규정 및 회의자료<br>• 입학제도에 대한 학내외 평가 및 개선자료<br>• 이의제기 시스템 관련 근거자료(방법, 명시자료 등) |

## 우수기준

### H.4.1.3 의과대학은 장애학생 입학에 대한 정책을 시행하고 있다.

| *자체평가연구보고서 작성 가이드* |
|---|
| 가이드-1. 장애학생 입학에 대한 정책이 있고 이를 시행하고 있다. |

*방문평가단 평가 가이드*

| 기준 | 핵심어 | 가이드 | 근거자료 |
|---|---|---|---|
| H.4.1.3 | • 장애학생 입학정책 | • 장애학생 입학에 대한 정책이 있고, 이를 시행하고 있는지 확인한다. | • 입학관련 규정 |

**[주]**

- 장애학생 입학에 대한 정책과 시행은 국가의 법과 규정을 준수해야 한다.

## 4.2 입학 정원

### 기본기준

**K.4.2.1 의과대학은 특별전형 모집요강에 입학정원과 선발기준을 명시하고 있다.**

| *자체평가연구보고서 작성 가이드* |
| --- |
| 가이드-1. 특별전형에 의한 선발기준이 있다. |

*방문평가단 평가 가이드*

| 기준 | 핵심어 | 가이드 | 근거자료 |
| --- | --- | --- | --- |
| K.4.2.1 | • 특별전형에 의한 입학정원과 선발기준 | • 특별전형에 의한 선발기준이 있는지 확인한다. | • 입학 관련 자료 |

**[주]**

- 특별전형은 지역사회와 사회의 건강 요구, 잠재적 요구, 불우학생, 성별, 인종, 다른 사회적인 요구에 따른 입학생에 대한 고려를 포함한다.

## 4.3 학생상담과 지원

### 기본기준

**K.4.3.1 의과대학은 학생들의 학업상담 제도를 갖추고 있으며 이를 활용하고 있다.**

*자체평가연구보고서 작성 가이드*

가이드-1. 자율적으로 학업상담을 받을 수 있는 상담시스템이 있다.
가이드-2. 전체학생의 학업성취를 관리하고, 학습부진학생을 지도하는 방안이 있다.
가이드-3. 학년별 유급에 대한 문제점을 분석하고 있다.

*방문평가단 평가 가이드*

| 기준 | 핵심어 | 가이드 | 근거자료 |
|---|---|---|---|
| K.4.3.1 | • 학생상담 시스템<br>• 학습부진학생 프로그램 | • 자율적으로 학업상담을 받을 수 있는 상담시스템이 있는지 확인한다.<br>• 전체학생의 학업성취를 관리하고 학습부진학생을 지도하고 있는지 확인한다.<br>• 학년별 유급에 대한 문제점을 분석하거나 개선 방안이 있는지 확인한다. | • 자율적 학업상담체제<br>• 연도별 학생 유급 현황<br>• 학습부진학생 지도 실적<br>• 상담 등 관련 활동실적<br>• 유급률 및 문제점 분석 자료 |

### 기본기준

**K.4.3.2 의과대학은 학생들의 진로지도를 위한 프로그램을 제공하고 있다.**

*자체평가연구보고서 작성 가이드*

가이드-1. 학생들의 진로지도를 위한 프로그램과 학생 진로지도 실적이 있다.
가이드-2. 학생들의 진로지도를 위한 프로그램을 학생들에게 홍보하고 있다.

*방문평가단 평가 가이드*

| 기준 | 핵심어 | 가이드 | 근거자료 |
|---|---|---|---|
| K.4.3.2 | • 학생 진로지도 프로그램과 실적<br>• 진로지도 프로그램 홍보 | • 학생들의 진로지도를 위한 프로그램과 실적이 있는지 확인한다.<br>• 학생들의 진로지도를 위한 프로그램을 학생들에게 홍보하고 있는지 확인한다. | • 진로지도 프로그램과 실적 (상담일지 포함)<br>• 진로지도 프로그램 홍보 자료 |

**기본기준**

**K.4.3.3 의과대학은 학생의 사회적, 재정적, 개인적 요구를 다루는 학생지원 프로그램을 제공하고 있다.**

*자체평가연구보고서 작성 가이드*

가이드-1. 학생의 사회적, 재정적, 개인적 요구를 상담하기 위해 운영 또는 개발 중인 프로그램이 있다.
가이드-2. 전문적·통합적인 학생지도를 하고 있다.

*방문평가단 평가 가이드*

| 기준 | 핵심어 | 가이드 | 근거자료 |
|---|---|---|---|
| K.4.3.3 | • 학생지원 프로그램 | • 학생의 사회적, 재정적, 개인적 요구를 상담하기 위해 운영 또는 개발 중인 프로그램이 있는지 확인한다.<br>• 전문적·통합적인 학생지도를 하고 있는지 확인한다. | • 학생지원 프로그램 관련 자료<br>• 학생 생활지도 실적(상담일지 포함)<br>• 학생지도 관련자료 |

**[주]**

- 사회적, 경제적, 개인적 요구를 다룬다는 것은 사회적이고 개인적인 문제와 사건들, 건강 문제, 재정문제와 관련된 지원을 의미하며, 보건 진료소에 대한 접근, 예방접종 프로그램과 건강/장애 보험뿐만 아니라 생활보조금, 학자금 대출과 같은 형태의 재정적 지원을 포함한다.

## 기본기준

### K.4.3.4 의과대학은 학생지원을 위한 인적, 물적 자원을 할당하고 있다.

*자체평가연구보고서 작성 가이드*

가이드-1. 장학제도의 운영을 위한 장학규정이 있고, 장학생 선발기준 및 절차가 다양하고, 엄정한 기준과 합리적인 절차에 따라 선발하고 있다.
가이드-2. 학자금 지원노력으로 대학과 연계된 학자금 융자체제가 있다.
가이드-3. 감염 및 환경위험에 노출된 경우, 관리 및 치료대책과 필요한 재정이 확보되어 있다.
가이드-4. 학생지원을 위한 행정담당자가 배정되어 있다.

*방문평가단 평가 가이드*

| 기준 | 핵심어 | 가이드 | 근거자료 |
|---|---|---|---|
| K.4.3.4 | • 장학규정<br>• 대학과 연계된 학자금 융자체계<br>• 감염 및 환경위험 노출 | • 장학제도의 운영을 위한 장학규정이 있고, 장학생 선발 기준 및 절차가 다양하고, 엄정한 기준과 합리적인 절차에 따라 선발하고 있는지 확인한다.<br>• 학자금 지원노력으로 대학과 연계된 학자금 융자체제가 있는지 확인한다.<br>• 감염 및 환경위험에 노출된 경우, 관리 및 치료대책과 필요한 재정이 확보되어 있는지 확인한다.<br>• 학생지원을 위한 행정담당자가 배정되어 있는지 확인한다. | • 학생지원을 위한 인적, 물적 자원 자료<br>• 교내·외 장학회 운영 및 장학생 선발관련 규정<br>• 장학금 지급 결정과 관련된 회의록<br>• 장학금 지급내역<br>• 학자금 융자체제<br>• 감염관련 지침 및 예산편성표<br>• 재정 집행 실적 |

## 기본기준

### K.4.3.5 의과대학은 학생들의 주거현황을 파악하고 있으며, 기숙사를 적절하게 운영하고 있다.

*자체평가연구보고서 작성 가이드*

가이드-1. 학생 주거 및 기숙사 입실현황을 분석하고 있다.
가이드-2. 기숙사 운영규정이 있고, 규정에 따라 운영하고 있다.
가이드-3. 학생 만족도조사를 실시하여 이를 바탕으로 개선하고 있다.

*방문평가단 평가 가이드*

| 기준 | 핵심어 | 가이드 | 근거자료 |
|---|---|---|---|
| K.4.3.5 | • 학생 주거현황<br>• 기숙사 | • 학생 주거 및 기숙사 입실현황, 기숙사 수요대비 공급비율을 분석하고 있는지 확인한다.<br>• 기본 및 편의시설을 위생적으로 관리하고 있는지 확인한다.<br>• 기숙사 운영규정을 확인하고, 규정에 따라 운영하고 있는지 확인한다(타지방 학생 우선 배정과 전용기숙사가 없을 경우 의학과 학생 배려 포함).<br>• 학생 만족도조사를 실시하여 이를 바탕으로 개선하고 있는지 확인한다. | • 학생 주거현황 분석자료<br>• 기숙사 운영규정<br>• 기숙사 시설(현장 확인)<br>• 관리 자료<br>• 학생 만족도조사 자료<br>• 학생 면담(현장 확인) |

## 기본기준

### K.4.3.6 의과대학은 학생상담과 지원에 관련한 비밀을 보장하고 있다.

*자체평가연구보고서 작성 가이드*

가이드-1. 학생상담과 지원에 관련한 비밀을 보장하는 제도가 있다.
가이드-2. 정서적, 신체적 스트레스에 대해 학생들이 쉽게 적응할 수 있도록 도와주는 대학의 개인 상담체제가 있다.
가이드-3. MMPI, 문장완성검사, 성격검사 등을 시행하고 있으며, 학생지도와 상담에 이용하고 있다.
가이드-4. 부당행위에 대한 예방과 보고체계, 피해학생 구제방안이 있다.

*방문평가단 평가 가이드*

| 기준 | 핵심어 | 가이드 | 근거자료 |
|---|---|---|---|
| K.4.3.6 | • 개인 상담체제<br>• 부당행위 관련 시스템 | • 학생상담과 지원에 관련한 비밀을 보장하는 제도가 있는지 확인한다.<br>• 정서적, 신체적 스트레스에 대해 학생들이 쉽게 적응할 수 있도록 도와주는 대학의 개인 상담체제가 있는지 확인한다.<br>• MMPI, 문장완성검사, 성격검사 등을 시행하고 있으며, 학생지도와 상담에 이용하고 있는지 확인한다.<br>• 부당행위에 대한 예방과 보고체계, 피해학생 구제방안이 있는지 확인한다. | • 학생상담과 지원에 대한 규정<br>• 개인 상담체제와 실적<br>• 심리상태 검사 실적자료와 상담 활용실적<br>• 부당한 행위에 대한 예방교육실적 자료<br>• 부당한 행위에 대한 홍보자료 및 실적<br>• 부당한 행위 사례 및 처리자료<br>• 학생, 교수, 교직원 면담(현장 확인) |

## 우수기준

**H.4.3.1 의과대학은 학생의 학업 진척도를 모니터링하고 진로지도 및 계획을 포함한 학업상담을 시행하고 있다.**

| *자체평가연구보고서 작성 가이드* |
|---|
| 가이드-1. 학생의 학업 진척도 모니터링을 바탕으로 한 학업상담이 이루어지고 있다.<br>가이드-2. 학생의 진로지도와 자문 등의 지도를 책임지고 수행하는 신뢰할 수 있는 조언자(멘토)가 있고, 조언자 양성프로그램이 있다. |

*방문평가단 평가 가이드*

| 기준 | 핵심어 | 가이드 | 근거자료 |
|---|---|---|---|
| H.4.3.1 | • 학업 진척도 모니터링<br>• 진로지도 및 학업상담 | • 학생의 학업 진척도 모니터링을 바탕으로 한 학업상담이 이루어지고 있는지 확인한다.<br>• 학생의 진로지도와 자문 등의 지도를 책임지고 수행하는 신뢰할 수 있는 조언자(멘토)가 있고, 조언자 양성프로그램이 있는지 확인한다. | • 학업진척도 모니터링 관련 자료<br>• 진로지도와 계획을 포함한 학업상담 관련 자료<br>• 조언자(멘토)와 학생 면담(현장 확인)<br>• 조언자 양성프로그램 운영 실적 |

## 우수기준

**H.4.3.2 의과대학은 입학, 의학과 진입, 임상실습 진입 시기에 건강검진을 실시하고, 임상실습 전 성인 예방접종을 시행하고 있다.**

| *자체평가연구보고서 작성 가이드* |
|---|
| 가이드-1. 입학, 의학과 진입 및 실습 진입 시 건강검진을 시행하고, 임상실습 전에 우리나라 성인 예방접종 지침에 따른 예방접종이 이루어지고 있다. |

*방문평가단 평가 가이드*

| 기준 | 핵심어 | 가이드 | 근거자료 |
|---|---|---|---|
| H.4.3.2 | • 건강검진<br>• 성인 예방접종 | • 입학, 의학과 진입 및 실습 진입 시 건강검진을 시행하고, 임상실습 전에 우리나라 성인 예방접종 지침에 따른 예방접종이 시행되는지를 확인한다. | • 건강검진 자료<br>• 성인 예방접종 실적 |

**[주]**

- 임상실습을 위한 건강검진과 성인 예방접종은 대학 또는 병원에서 지원한다.

## 우수기준

**H.4.3.3 학생이 정신과 또는 심리치료를 받을 수 있는 외부 의료기관과의 연계체계를 갖추고 있고, 의과대학 내에는 전임상담원이 활동하는 상담실이 있다.**

| *자체평가연구보고서 작성 가이드* |
|---|
| 가이드-1. 필요시 정신과 치료 또는 심리상담을 받을 수 있는 외부 의료전문가 이용체계가 있다.<br>가이드-2. 학생상담실이 있고 전임상담원이 근무하고 있다. |

*방문평가단 평가 가이드*

| 기준 | 핵심어 | 가이드 | 근거자료 |
|---|---|---|---|
| H.4.3.3 | • 정신과 치료 또는 심리상담 | • 필요시 정신과 치료 또는 심리상담을 받을 수 있는 외부 의료전문가 이용체계가 있는지 확인한다.<br>• 학생상담실과 전임상담원이 있고, 상담 실적이 있는지 확인한다. | • 외부 의료전문가 위촉관련 근거자료<br>• 전임상담원과 체제에 대한 규정<br>• 상담실적<br>• 학생 면담(현장 확인) |

**[주]**

- 전임상담원이 활동한다는 것은 상담을 전담하는 전문인력이 있다는 의미이다. 의과대학의 학생담당 부서 직원, 학생담당 부학장 등이 지도하는 경우에는 전문적 운영으로 해석하지 않는다. 의과대학이 아닌 본교에 상담센터가 있는 경우에는 지리적으로 학생들의 접근이 용이하며, 의과대학 학생을 위한 별도의 전문상담체제가 갖추어져 있고, 실적이 있어야 한다.

## 4.4 학생 대표

### 기본기준

**K.4.4.1 의과대학은 사명, 교육과정 설계, 관리, 평가와 그 외 학생과 관련된 문제에 대한 학생대표의 적절한 참여를 보장하고 있다.**

| *자체평가연구보고서 작성 가이드* |
|---|
| 가이드-1. 사명, 교육과정 설계, 관리, 평가와 기타 학생관련 문제들에 대한 학생대표의 적절한 참여를 보장하는 정책을 수립하고 시행하고 있다. |

*방문평가단 평가 가이드*

| 기준 | 핵심어 | 가이드 | 근거자료 |
|---|---|---|---|
| K.4.4.1 | • 학생대표의 참여 보장 | • 사명, 교육과정 설계, 관리, 평가와 기타 학생관련 문제들에 대한 학생대표의 적절한 참여를 보장하는 정책을 수립하고 시행하고 있는지 확인한다. | • 교육과정 관련 위원회 규정 및 회의자료<br>• 학생관련 위원회 규정 및 회의자료 |

**[주]**

- 학생 대표의 참여에는 학생관련 위원회와 교육관련 위원회를 포함한다.

기본기준

**K.4.4.2 의과대학은 학생활동과 학생의 자치활동을 장려하고 있다.**

*자체평가연구보고서 작성 가이드*

가이드-1. 학생자치조직이 있다.
가이드-2. 학생활동에 대한 지도와 지원이 있다.
가이드-3. 학술 및 연구활동, 사회봉사 등에 학생들이 참여하고 있다.

*방문평가단 평가 가이드*

| 기준 | 핵심어 | 가이드 | 근거자료 |
|---|---|---|---|
| K.4.4.2 | • 학생자치활동 | • 학생자치조직이 있는지 확인한다.<br>• 학생활동에 대한 지도와 지원이 있는지 확인한다.<br>• 학술 및 연구 활동, 사회봉사 등에 학생들이 참여하고 있는지 확인한다. | • 학생활동과 학생관련 단체 활동 자료<br>• 학생자치 조직표<br>• 학생면담(현장 확인)<br>• 학생의 학술, 연구, 사회봉사, 동아리 현황 관련 자료<br>• 지원실적 및 자료 |

**[주]**

- 학생활동의 활성화에는 학생단체에 대한 기술적, 재정적 지원 제공을 포함한다.

ASK2019

# 05 교수

## 5.1 채용과 선발정책

### 기본기준

**K.5.1.1 의과대학은 세계의학교육연합회가 권고하는 기초의학 분야별로 적절한 수의 교수를 확보하고 있다.**

| *자체평가연구보고서 작성 가이드* |
| --- |
| 가이드-1. 기초의학 전임교수를 분야별로 적절하게 확보하고 있다. |

*방문평가단 평가 가이드*

| 기준 | 핵심어 | 가이드 | 근거자료 |
| --- | --- | --- | --- |
| K.5.1.1 | • 기초의학<br>• 전임교수의 수<br>• 분야별 적절성 | • 기초의학 전임교수가 분야별로 적절하게 확보되어 있는지 확인한다. | • 기초의학 전임교수 명단<br>• 인사기록(급여대장)<br>• 교육활동 자료(수업계획서 등) |

**[주]**

- 기초의학 분야는 K.2.3.1 참조.
- 전임교수는 교육부에 등록된 교수를 말한다. 다만 총장발령으로 대학에서 급여를 받고 있는 초빙교수, 기금교수, 연구교수 등의 경우로서 전임교수와 동일한 교육, 연구, 봉사 활동을 하고 있으며, 재임용이나 승진시 교육, 연구, 봉사 업적을 적용받는 경우에는 포함할 수 있다.
- 기초의학 전임교수의 수는 총 25명 이상이어야 한다.
- 분야는 교실 혹은 과목을 의미하는 것이 아니라 교육내용을 의미한다.

## 기본기준

### K.5.1.2 의과대학은 의학교육 담당기구가 있고 의학교육을 담당하는 전임교수가 있다.

| *자체평가연구보고서 작성 가이드* |
|---|
| 가이드-1. 의학교육 담당기구(교실, 실, 학과, 센터)가 개설되어 있다.<br>가이드-2. 의학교육을 담당하는 전임교수가 있다. |

*방문평가단 평가 가이드*

| 기준 | 핵심어 | 가이드 | 근거자료 |
|---|---|---|---|
| K.5.1.2 | • 의학교육 담당 기구<br>• 의학교육 전임교수 | • 의학교육 담당 기구(교실, 실, 학과, 센터)가 개설되어 있다.<br>• 의학교육을 담당하는 전임교수가 있는지 확인한다. | • 인사기록(급여대장)<br>• 업무규정 |

[주]

- 의학교육 전임교수는 의학교육 담당 기구(교실, 실, 학과, 센터)에 소속된 의과대학 교수를 말한다. 단, 의학교육 전임교수가 타 교실에 겸임하는 경우, 80%이상을 의학교육 업무에 할애해야 한다.

## 기본기준

### K.5.1.3 의과대학은 의료인문학 분야에 전임 교수 또는 전담 교수를 적절하게 확보하고 있다.

| *자체평가연구보고서 작성 가이드* |
|---|
| 가이드-1. 의료인문학 전임교수 혹은 의료인문학 전담교수를 적절히 확보하고 있다. |

*방문평가단 평가 가이드*

| 기준 | 핵심어 | 가이드 | 근거자료 |
|---|---|---|---|
| K.5.1.3 | • 의료인문학 전임 교수, 전담교수<br>• 교육활동 내용 | • 의료인문학 전임교수나 전담교수가 있는지 확인한다.<br>• 전담교수는 의료인문학 분야 교육과정의 설계, 실행, 및 평가에 적극적으로 참여하고 있는지 확인한다. | • 인사기록(급여대장)<br>• 업무규정<br>• 교육활동 자료(수업계획서 등) |

**[주]**

- 의료인문학 전임교수는 해당분야의 전공자(어문학, 사학, 철학, 윤리학, 사회학, 법학, 경영학, 인류학, 심리학, 예술 등)로 의과대학 교수인 경우 행정적으로 의료인문학 담당기구(교실, 실, 학과, 센터)에 소속되어 있는 자를 의미하며, 타 교실에 겸임하는 경우, 80%이상을 의료인문학 업무에 할애해야 한다.
- 의료인문학 전담교수는 의과대학 교수이면서 의료인문학 교육과정의 설계, 진행, 평가에 활발히 참여하는 자를 말한다.
- 의과대학에는 의료인문학 전임교수가 1인 이상이거나 전담교수가 3명 이상이 있어야 한다.

기본기준

**K.5.1.4 의과대학은 각 임상의학 전공과목별로 적절한 수의 전임교수를 확보하고 있다.**

*자체평가연구보고서 작성 가이드*

가이드-1. 임상의학 전공과목이 적절하게 개설되어 있다.
가이드-1. 임상의학 전공과목별로 적절한 수의 전임교수를 확보하고 있다.

*방문평가단 평가 가이드*

| 기준 | 핵심어 | 가이드 | 근거자료 |
|---|---|---|---|
| K.5.1.4 | • 임상의학 전공과목의 적절성<br>• 전임교수의 수 | • 임상의학 전공과목이 적절하게 개설되어 있는지 확인한다.<br>• 임상의학 전공과목별로 적절한 수의 전임교수를 확보하고 있는지 확인한다. | • 인사기록(급여대장)<br>• 교육활동 자료(수업계획서 등) |

**[주]**

- 임상의학 전공과는 총 20개 이상이어야 한다.
- 임상의학 전임교수는 전공과별로 1인 이상, 총 85명 이상이어야 한다.

기본기준

**K.5.1.5 의과대학은 교수를 채용할 때 연구, 교육, 임상분야 업적에 대한 기준이 있고, 이를 시행하고 있다.**

*자체평가연구보고서 작성 가이드*

가이드-1. 전공에 따른 업적에 대한 뚜렷한 기준을 가진 교수채용 정책을 수립하고 있고, 이에 따라 교수를 채용하고 있다.

*방문평가단 평가 가이드*

| 기준 | 핵심어 | 가이드 | 근거자료 |
|---|---|---|---|
| K.5.1.5 | • 교수채용 정책<br>• 교수채용 기준 | • 명문화된 교수채용 기준이 존재하고, 이에 근거하여 교수채용이 이루어지고 있는지 확인한다. | • 임용규정 및 채용공고 관련 자료<br>• 관련 규정 |

**[주]**

- 업적은 공식적인 자격증, 전문적 경험, 연구 결과, 교육관련 수상들과 동료인정으로 평가한다.

## 기본기준

**K.5.1.6 의과대학은 기초의학, 의학교육학, 의료인문학, 임상의학 교수의 책무를 구체화하는 채용 정책을 수립하여 시행하고 있다.**

| *자체평가연구보고서 작성 가이드* |
|---|
| 가이드-1. 교수채용 정책에 기초의학, 의학교육학, 의료인문학, 임상의학 교수의 책무가 구체화되어 있다.<br>가이드-2. 구체적 책무에 따라 명문화된 교수채용 기준이 있고, 이에 따라 교수를 채용하고 있다. |

*방문평가단 평가 가이드*

| 기준 | 핵심어 | 가이드 | 근거자료 |
|---|---|---|---|
| K.5.1.6 | • 교수채용과 선발 정책<br>• 구체적 책무 | • 전공분야(기초, 임상, 의학교육학, 의료인문학)에 따라 교육, 임상, 연구 등의 업적 기준이 교수채용 목적에 부합하고 있는지 확인한다. | • 임용 및 채용공고 관련 자료<br>• 관련 규정<br>• 선발기준 적용사례 |

## 우수기준

**H.5.1.1 의과대학은 교육, 연구, 봉사기능 간의 균형을 이루어 교수를 채용하는 정책을 수립하여 시행하고 있다.**

| *자체평가연구보고서 작성 가이드* |
|---|
| 가이드-1. 교육, 연구, 봉사기능 간의 균형을 이루는 교수를 채용하는 정책을 수립하여 시행하고 있다. |

*방문평가단 평가 가이드*

| 기준 | 핵심어 | 가이드 | 근거자료 |
|---|---|---|---|
| H.5.1.1 | • 교수채용 정책<br>• 교육, 연구, 봉사 | • 교육, 연구, 봉사업적을 감안한 교수를 채용하는 정책을 수립하여 시행하고 있는지 확인한다. | • 채용공고 관련 자료<br>• 관련 규정 |

## 5.2 교수활동과 개발 정책

### 기본기준

**K.5.2.1 의과대학은 교수가 교육, 연구, 봉사활동 간의 균형을 이룰 수 있도록 정책을 수립하여 시행하고 있다.**

*자체평가연구보고서 작성 가이드*

가이드-1. 업적평가나 승진규정을 통해 교육, 연구, 봉사활동 간의 균형을 이루는 활동을 교수에게 권장하며 이와 관련된 정책을 수립하고 시행하고 있다.
가이드-2. 전임교수의 연구와 진료 이외의 학회활동 등 사회봉사활동을 보장하고 있다.

*방문평가단 평가 가이드*

| 기준 | 핵심어 | 가이드 | 근거자료 |
|---|---|---|---|
| K.5.2.1 | • 교수활동과 개발 정책<br>• 사회봉사활동에 대한 보장 | • 업적평가나 승진규정을 통해 교육, 연구, 봉사활동 간의 균형을 이루는 활동을 교수에게 권장하는지 확인한다.<br>• 교수복무규정과 출장기록 등을 통해 학회활동 등 사회봉사활동을 보장하는지 확인한다. | • 업적평가, 승진 규정<br>• 학회활동 등 사회봉사활동 실적<br>• 관련 교수복무규정<br>• 출장기록 등<br>• 교수 면담 |

**기본기준**

## K.5.2.2 의과대학은 교수업적평가 제도를 수립하여 시행하고 있다.

*자체평가연구보고서 작성 가이드*

가이드-1. 교수업적평가 제도를 시행하고, 이를 승진 등에 반영하고 있다.
가이드-2. 교수업적평가 제도에는 전임교수의 의학교육 연수나 교육관련 교수개발 프로그램에 참여해야 하는 연간 교육시간 등이 제도적으로 의무화되어 있다.
가이드-3. 전임교수가 의학교육 연수나 교육관련 교수개발 프로그램에 적절하게 참석하고 있다.

*방문평가단 평가 가이드*

| 기준 | 핵심어 | 가이드 | 근거자료 |
|---|---|---|---|
| K.5.2.2 | • 교수업적평가<br>• 의학교육 연수 의무화<br>• 교육관련 교수개발 프로그램의 적절성 | • 교수업적평가 제도를 시행하고, 이를 승진 등에 반영하고 있는지 확인한다.<br>• 전임교수의 의학교육 연수나 교육관련 교수개발 프로그램 참여가 제도적으로 의무화되어 있는지 규정집을 확인한다.<br>• 전임교수가 의학교육 연수나 교육관련 교수개발 프로그램에 적절하게 참석하였는지 현황을 확인한다. | • 교수업적평가 제도 시행자료<br>• 규정집<br>• 교수업적평가 제도 및 현황 (평가기준, 실제 평가자료 등)<br>• 의학교육 연수/교수개발 프로그램 교육 시행 자료와 출석현황 |

**[주]**

- 대학은 승진, 급여 혹은 수당 등의 보상을 통해 교육과 연구활동 업적을 인정한다.
- 의학교육 연수나 교육관련 교수개발 프로그램에 전임교수의 50% 이상이 연간 3시간 이상 참석해야 한다(온라인 교육은 인정하지 않음).

**기본기준**

**K.5.2.3 의과대학은 신임교수를 위한 의학교육 연수과정에 신임교수가 의무적으로 참여하도록 하고 있다.**

*자체평가연구보고서 작성 가이드*

가이드-1. 신임교수를 위한 의학교육 연수과정을 의무적으로 시행하고 있다.
가이드-2. 신임교수를 위한 적절한 교육이 시행되고 있다.

*방문평가단 평가 가이드*

| 기준 | 핵심어 | 가이드 | 근거자료 |
|---|---|---|---|
| K.5.2.3 | • 신임교수 의학교육 연수 의무화<br>• 신임교수 의학교육 연수내용의 적절성 | • 신임교수를 위한 의학교육 연수과정이 의무적으로 시행되고 있는지 확인한다.<br>• 신임교수가 적절한 교육을 받았는지 확인한다. | • 관련 규정<br>• 의학교육 관련 신임교수 워크숍 관련 자료와 시간(책자, 프로그램 참석 신임교수 명단 등) |

**[주]**

- 신임교수는 임용 후 1년 이내에 신임교수를 위한 의학교육 연수과정을 15시간 이상 이수해야 한다.

**기본기준**

**K.5.2.4 의과대학은 교수업적평가 기준에 학생교육이나 학술활동 이외의 학회활동이나 공공 목적의 사회봉사활동에 대한 평가기준을 가지고 있다.**

*자체평가연구보고서 작성 가이드*

가이드-1. 교수업적평가 기준에 사회봉사활동 관련 기준을 가지고 있다.
가이드-2. 교수승진, 승급과 재임용에 봉사업적이 반영되어 있다.

*방문평가단 평가 가이드*

| 기준 | 핵심어 | 가이드 | 근거자료 |
|---|---|---|---|
| K.5.2.4 | • 봉사관련 교수업적 | • 교수업적평가 기준에 사회봉사활동 관련 기준이 마련되어 있는지 확인한다.<br>• 교수 승진, 승급과 재임용에 봉사업적이 반영되어 있는지 확인한다. | • 교수업적평가 기준 시행자료<br>• 규정 확인<br>• 교수 면담 |

**기본기준**

**K.5.2.5 의과대학은 교수 모두가 전체 교육과정을 숙지할 수 있는 정책이 있고 이와 관련된 교수활동을 지원하고 있다.**

*자체평가연구보고서 작성 가이드*

가이드-1. 전체 교육과정을 교수 모두가 숙지할 수 있도록 관련 정책을 수립하여 시행하고 있다.
가이드-2. 전체 교육과정에 대한 교수들의 이해를 돕는 활동을 지원하고 있다.

*방문평가단 평가 가이드*

| 기준 | 핵심어 | 가이드 | 근거자료 |
|---|---|---|---|
| K.5.2.5 | • 전체 교육과정<br>• 교수활동 지원 | • 전체 교수를 대상으로 전체 교육과정을 설명하는 시간이 있는지 확인한다,<br>• 전체 교육과정을 이해하고자 하는 교수들의 노력을 지원하고 있는지 확인한다. | • 교수활동 근거자료(전체 교수워크숍, 책임교수워크숍, 교과목 회의 등)와 출석현황<br>• 교수 면담 |

**[주]**

- 전체 교육과정에 대한 숙지는 전반적인 교육과정과 타 과정/교과목 분야의 교수법/학습법에 대한 내용을 포함한다.

**기본기준**

**K.5.2.6 의과대학은 교육을 위한 연수와 교수개발에 교수가 참여할 수 있도록 지원하는 정책을 수립하여 시행하고 있다.**

*자체평가연구보고서 작성 가이드*

가이드-1. 교수의 교육역량 강화를 지원하는 정책을 수립하여 시행하고 있다.
가이드-2. 교수의 전문역량 강화를 지원하는 정책을 수립하여 시행하고 있다.

*방문평가단 평가 가이드*

| 기준 | 핵심어 | 가이드 | 근거자료 |
|---|---|---|---|
| K.5.2.6 | • 교수개발과 지원 정책 | • 교수의 교육역량 강화를 지원하는 정책을 수립하여 시행하고 있는지 확인한다.<br>• 교수의 전문역량 강화를 지원하는 정책을 수립하여 시행하고 있는지 확인한다. | • 교육연수와 개발관련 자료<br>• 재정지원 현황<br>• 교수면담 |

**[주]**

- 재정지원은 교수 1인당 연평균 2백만 원 이상이어야 한다.

## 우수기준

### H.5.2.1 의과대학은 교수 승진정책을 수립하여 시행하고 있다.

*자체평가연구보고서 작성 가이드*

가이드-1. 직급별로 세분화된 교수업적평가 제도가 있고, 이에 따라 교수 승진정책을 시행하고 있다.
가이드-2. 기능별로 세분화된 교수업적평가 제도가 있고, 이에 따라 교수 승진정책을 시행하고 있다.
가이드-3. 교육활동에 대한 인센티브 제도가 있다.

*방문평가단 평가 가이드*

| 기준 | 핵심어 | 가이드 | 근거자료 |
|---|---|---|---|
| H.5.2.1 | • 교수 승진정책<br>• 교육업적<br>• 교육 인센티브 | • 직급별로 세분화된 교수업적평가 제도가 있고, 이에 따른 교수 승진정책을 시행하고 있는지 확인한다.<br>• 기능별로 세분화된 교수업적평가 제도가 있고, 이에 따른 교수 승진정책을 시행하고 있는지 확인한다.<br>• 교육활동에 대한 인센티브 제도가 있는지 확인한다. | • 승진규정<br>• 관련 교수업적평가 규정<br>• 교육 인센티브 관련 규정 |

ASK2019

# 06 교육 자원

## 6.1 시설

### 기본기준

**K.6.1.1 의과대학은 학생 교육을 위한 교육 기본시설을 적절하게 갖추고 있다.**

*자체평가연구보고서 작성 가이드*

가이드-1. 교육과정을 고려할 때, 강의실의 시설과 기자재가 적절하다.
가이드-2. 교육과정을 고려할 때, 실험 실습실의 시설과 기자재가 적절하다.
가이드-3. 교육과정을 고려할 때, 각 시설별 멀티미디어 시설, 조명, 냉난방, 방음, 환기 및 청결상태가 적절하다.

*방문평가단 평가 가이드*

| 기준 | 핵심어 | 가이드 | 근거자료 |
|---|---|---|---|
| K.6.1.1 | • 교육 기본시설 | • 교육과정을 고려할 때, 강의실의 시설과 기자재가 적절한지 확인한다.<br>• 교육과정을 고려할 때, 실험 실습실의 시설과 기자재가 적절한지 확인한다.<br>• 교육과정을 고려할 때, 각 시설별 멀티미디어 시설, 조명, 냉난방, 방음, 환기 및 청결상태가 적절한지 확인한다. | • 현장 확인<br>• 실험 실습실에 놓여있는 기자재 보유 목록<br>• 실험실습기자재 사용 일지 |

**[주]**

- 교육 기본시설에는 강의실, 실험실습실, 임상수기실습실을 포함한다.

## 기본기준

### K.6.1.2 의과대학은 학생 교육을 위한 교육 지원시설을 적절하게 갖추고 있다.

*자체평가연구보고서 작성 가이드*

가이드-1. 교육과정 운영에 필요한 다양한 교육 지원시설이 있다.
가이드-2. 시설별 멀티미디어 시설, 조명, 냉난방, 방음, 환기 및 청결상태 등을 적절히 유지한다.

*방문평가단 평가 가이드*

| 기준 | 핵심어 | 가이드 | 근거자료 |
|---|---|---|---|
| K.6.1.2 | • 교육 지원시설 | • 교육과정 운영에 필요한 다양한 교육 지원시설이 있는지 확인한다.<br>• 시설별 멀티미디어 시설, 조명, 냉난방, 방음, 환기 및 청결상태 등이 양호한지 확인한다. | • 현장 확인<br>• 기자재 보유 목록 |

**[주]**

- 교육 지원시설에는 그룹지도실(소그룹토의실), 연구실험실, 도서관, 정보 기술 시설, 자율학습실을 포함한다.

기본기준

### K.6.1.3 의과대학은 학생 복지시설 및 편의시설을 적절하게 갖추고 있다.

*자체평가연구보고서 작성 가이드*

가이드-1. 학생 복지시설은 학생 수에 대비하여 적절하다.
가이드-2. 학생 편의시설이 적절히 확보되어 있고, 이용 가능 시간대가 적절하다.

*방문평가단 평가 가이드*

| 기준 | 핵심어 | 가이드 | 근거자료 |
|---|---|---|---|
| K.6.1.3 | • 학생 복지시설<br>• 편의시설<br>• 접근성<br>• 편리성 | • 학생회방, 동아리방, 남·여 휴게실은 학생 수에 대비하여 충분한지 확인한다.<br>• 운동시설, 식당, 매점, 자동판매기, 개인용 사물함 등 편의시설이 충분한지 확인한다.<br>• 각종 편의시설의 이용 가능시간대는 적절한지 확인한다. | • 현장 확인<br>• 학생면담 |

**[주]**

- 학생 복지시설은 학생회방, 동아리방, 남·여 휴게실 등을 포함한다.
- 학생 편의시설은 운동시설, 식당, 매점, 자동판매기, 개인 사물함 등을 포함한다.

기본기준

**K.6.1.4 의과대학은 학생의 교육과 복지를 위한 시설을 관리하는 인력이 있고 그에 대해 적절한 예산이 배정되어 있다.**

*자체평가연구보고서 작성 가이드*

가이드-1. 시설·설비의 관리·유지·운영을 위한 인력과 체계가 적절하다.
가이드-2. 시설·설비의 관리·유지·운영을 위한 예산이 적절하다.
가이드-3. 정기적으로 시설·설비에 대한 만족도조사를 하고 있다.
가이드-4. 학생의 개선요구에 따른 개선실적이 있다.

*방문평가단 평가 가이드*

| 기준 | 핵심어 | 가이드 | 근거자료 |
|---|---|---|---|
| K.6.1.4 | • 교육과 복지를 위한 시설<br>• 예산<br>• 인력 | • 시설·설비의 관리·유지·운영을 위한 전담 인력과 운영 규정이 있는지 확인한다.<br>• 시설·설비의 관리·유지·운영을 위한 예산이 적절한지 확인한다.<br>• 정기적으로 시설·설비에 대한 만족도조사를 하고 있는지 확인한다.<br>• 학생의 개선요구에 따른 개선실적이 있는지 확인한다. | • 관련 규정<br>• 현장 확인<br>• 학생면담<br>• 시설·설비의 관리·유지·운영 예산집행 내역<br>• 교육 및 복지시설의 관리 인력 면담<br>• 만족도조사와 개선실적 |

## 기본기준

### K.6.1.5 의과대학의 학생 1인당 연간 등록금 대비 학생교육 관련 직접비용이 적절하다.

| *자체평가연구보고서 작성 가이드* |
|---|
| 가이드-1. 학생교육 관련 직접비용이 적절하다. |

*방문평가단 평가 가이드*

| 기준 | 핵심어 | 가이드 | 근거자료 |
|---|---|---|---|
| K.6.1.5 | • 학생교육 직접 비용 | • 학생교육 관련 직접비용이 적절한지 확인한다.<br>• 전체 등록금 대비 학생교육에 지출한 내역을 확인한다. | • 전체 등록금 대비 학생 교육에 관련한 예결산 내역서 |

**[주]**

- 학생 교육관련 직접비용은 기초의학 실험실습비, 표본제작, CPX와 OSCE 관련 비용, 교재비, 학생 교육용 컴퓨터프로그램, 표준화환자 인건비, 시험문항 개발비, 임상술기실습실 소모품, PBL과 TBL 운영비, 임상실습 관련 비용 등을 포함한다.
- 예산의 적절성은 학생 1인당 등록금 대비 학생교육 관련 직접비용 비율, 정기적인 수업환경 관련 만족도 조사 결과, 학생의 개선 요구에 따른 개선실적 등을 근거로 한다.
- 적절한 학생교육 관련 직접비용은 학생 1인당 연간 등록금 대비 5%이상이다.

## 기본기준

### K.6.1.6 의과대학은 개인 교수실이 적절히 확보되어 있고, 적절한 행정지원 체계를 갖추고 있다.

*자체평가연구보고서 작성 가이드*

가이드-1. 전임교수 이상의 개인 교수실 확보율이 적절하다.
가이드-2. 개인 교수실 실내 설비(채광, 환기, 냉난방, LAN 등)가 양호하다.
가이드-3. 기초의학 교실(과)별 행정지원인력이 적절하다.
가이드-4. 임상의학 교실(과)별 행정지원인력이 적절하다.

*방문평가단 평가 가이드*

| 기준 | 핵심어 | 가이드 | 근거자료 |
|---|---|---|---|
| K.6.1.6 | • 개인 교수실<br>• 행정지원인력 | • 전임교수 이상의 개인 교수실 확보율이 적절한지 확인한다(진료실 겸용 제외).<br>• 개인 교수실 실내 설비가 양호한지 확인한다(채광, 환기, 냉난방, 유무선 LAN 등).<br>• 기초의학 교실(과)별 적절한 행정지원인력이 있는지 확인한다.<br>• 임상의학 교실(과)별 적절한 행정지원인력이 있는지 확인한다. | • 현장 확인<br>• 개인 교수실 확보 현황표<br>• 교수 면담<br>• 행정지원인력 명단 |

**[주]**

- 전임교수 이상의 개인 교수실 확보율은 80% 이상이어야 한다.
- 행정지원인력은 각 교실별 1명 이상이어야 한다.

## 기본기준

### K.6.1.7 의과대학은 교수들의 연구를 위한 적절한 공간과 시설 설비를 갖추고 있다.

| *자체평가연구보고서 작성 가이드* |
|---|
| 가이드-1. 적절한 수준의 연구기자재가 있고, 관리상태가 양호하다.<br>가이드-2. 과별 연구실과 공동연구실의 기자재의 종류, 보유현황 및 관리실태 자료가 있다. |

*방문평가단 평가 가이드*

| 기준 | 핵심어 | 가이드 | 근거자료 |
|---|---|---|---|
| K.6.1.7 | • 연구를 위한 공간<br>• 시설 설비 | • 적절한 수준의 연구기자재가 있고, 관리상태가 양호한지 확인한다.<br>• 과별 연구실과 공동연구실의 기자재의 종류, 보유현황 및 관리실태 자료가 있는지 확인한다. | • 현장 확인<br>• 교수면담<br>• 교수 연구시설 현황표<br>• 과별 연구실과 공동 연구실의 기자재의 종류, 보유현황 및 관리실태 |

## 기본기준

### K.6.1.8 의과대학은 교직원, 학생, 환자에게 안전한 학습 환경을 보장하고 있다.

| *자체평가연구보고서 작성 가이드* |
|---|
| 가이드-1. 안전관련 시설이 적절하게 관리되고 있다.<br>가이드-2. 안전관련 시설에 대한 목록과 점검한 자료가 있다.<br>가이드-3. 안전교육을 시행한 자료가 있다. |

*방문평가단 평가 가이드*

| 기준 | 핵심어 | 가이드 | 근거자료 |
|---|---|---|---|
| K.6.1.8 | • 안전한 환경 | • 안전관련 시설이 적절하게 관리되고 있는지 확인한다.<br>• 안전관련 시설에 대한 목록과 점검한 자료가 있는지 확인한다.<br>• 안전교육을 시행한 자료가 있는지 확인한다. | • 현장 확인<br>• 안전교육 관련 자료 |

**[주]**

- 안전한 학습 환경은 방사선, 유해물질과 시료와 유기체로부터의 보호와 필수정보를 제공하고, 실험실 안전규정과 안전장비를 포함한다.

## 우수기준

**H.6.1.1 의과대학은 교육활동의 변화에 맞추어 시설의 변경 또는 확장 등 학습 환경을 개선하고 있다.**

| *자체평가연구보고서 작성 가이드* |
| --- |
| 가이드-1. 교육활동의 변화에 맞추어 시설의 변경 또는 확장 등 학습 환경을 개선한 실적이 있다. |

*방문평가단 평가 가이드*

| 기준 | 핵심어 | 가이드 | 근거자료 |
| --- | --- | --- | --- |
| H.6.1.1 | • 학습 환경 개선 | • 교육 활동의 개발에 맞추어 시설의 변경 또는 확장 등 학생들을 위한 학습 환경을 개선한 실적이 있는지 확인한다. | • 현장 확인<br>• 학생 면담<br>• 개선 실적 |

## 6.2 임상실습 자원

### 기본기준

**K.6.2.1 의과대학은 학생들이 적절한 임상경험을 할 수 있도록 다양한 질환의 환자와 충분한 수의 환자를 확보하고 있다.**

| *자체평가연구보고서 작성 가이드* |
|---|
| 가이드-1. 학생들이 필수환자군을 경험하기에 충분한 환자를 확보하고 있다. |

*방문평가단 평가 가이드*

| 기준 | 핵심어 | 가이드 | 근거자료 |
|---|---|---|---|
| K.6.2.1 | • 임상실습 자원<br>• 필수환자군 | • 학생들이 필수환자군을 경험하기에 충분한 환자를 확보하고 있는지 확인한다. | • 병원 연간보고서<br>• 임상실습 평가자료<br>• 필수환자군 경험을 확인할 수 있는 자료 |

## 기본기준

**K.6.2.2 의과대학은 학생들이 적절한 임상경험을 할 수 있도록 충분한 임상실습시설을 확보하고 있다.**

| *자체평가연구보고서 작성 가이드* |
|---|
| 가이드-1. 교육병원의 규모가 임상실습에 적절하다.<br>가이드-2. 교육병원 내 학생교육시설이 적절히 확보되어 있다.<br>가이드-3. 교육병원 내 학생전용공간이 적절히 확보되어 있다. |

*방문평가단 평가 가이드*

| 기준 | 핵심어 | 가이드 | 근거자료 |
|---|---|---|---|
| K.6.2.2 | • 임상실습시설 | • 교육병원의 규모가 임상실습에 적절한지 확인한다.<br>• 교육병원 내 학생교육 시설이 적절히 확보되어 있는지 확인한다(예: 예진실 등).<br>• 교육병원 내 학생전용공간이 적절히 확보되어 있는지 확인한다. | • 현장 확인<br>• 학생 면담<br>• 병원 내 각종 학생교육시설 목록<br>• 병원 내 학생전용공간 배치도 |

**[주]**

- 임상실습시설에는 진료시설과 모든 주요 임상과의 순환을 원활하게 할 수 있는 임상실습이 가능한 병원(1차, 2차, 3차 병원의 적절한 배합), 외래 진료, 의원, 보건소, 기타 지역사회 보건진료 시설뿐만 아니라 임상술기실습실을 포함하며, 500병상 이상이어야 한다.
- 교육병원 내 학생전용공간은 학생 20명당 최소 1개 이상이어야 한다.

## 기본기준

### K.6.2.3 의과대학은 학생들이 적절한 임상경험을 할 수 있도록 학생 진료행위에 대한 관리체계가 있다.

| *자체평가연구보고서 작성 가이드* |
| --- |
| 가이드-1. 과별로 임상실습 책임교수가 임명되어 있다.<br>가이드-2. 학생교육을 담당하는 교육전공의(Resident as Teacher)가 있고, 실제로 학생교육을 하고 있다.<br>가이드-3. 학생들이 임상경험을 할 수 있도록 학생의 진료를 감독하고 있다.<br>가이드-4. 학생의 진료에 대해 피드백을 하고 있다. |

*방문평가단 평가 가이드*

| 기준 | 핵심어 | 가이드 | 근거자료 |
| --- | --- | --- | --- |
| K.6.2.3 | • 임상실습 체계 | • 과별로 임상실습 책임교수가 임명되어 있는지 확인한다.<br>• 학생교육을 담당하는 교육전공의(Resident as Teacher)가 있고, 실제로 학생교육을 하고 있는지 확인한다.<br>• 학생들이 임상경험을 할 수 있도록 학생의 진료를 감독하고 있는지 확인한다.<br>• 학생의 진료에 대해 피드백을 하고 있는지 확인한다. | • 학생 면담<br>• 교육전공의 면담<br>• 임상실습지침서<br>• 임상실습 책임교수 임명 공문 및 명단<br>• 임상실습 교육 담당 전공의 임명 공문 및 명단<br>• 임상실습 관련 자료(피드백 자료 등) |

**[주]**

- 임상실습시설에 대한 평가에는 시설, 설비, 환자의 수와 종류 그리고 보건진료(health practices), 감독, 행정측면에서 임상실습 프로그램의 질과 적절성을 포함한다.

## 우수기준

**H.6.2.1 의과대학은 병원을 이용하는 사람들에게 불편을 주지 않으면서, 임상실습을 하는 데 필요한 시설을 평가, 조정, 개선하고 있다.**

| *자체평가연구보고서 작성 가이드* |
|---|
| 가이드-1. 교육병원 내 임상실습 시설이 문제가 없는지 평가하고 있다.<br>가이드-2. 병원을 이용하는 사람들에게 불편을 주지 않으면서, 임상실습시설을 개선한 실적이 있다. |

*방문평가단 평가 가이드*

| 기준 | 핵심어 | 가이드 | 근거자료 |
|---|---|---|---|
| H.6.2.1 | • 임상실습 시설 평가, 조정, 개선 | • 교육병원 내 임상실습 시설이 문제가 없는지 평가하고 있는지 확인한다.<br>• 병원을 이용하는 사람들에게 불편을 주지 않으면서, 임상실습시설을 개선한 실적이 있는지 확인한다. | • 개선 실적 |

## 6.3 정보기술

### 기본기준

**K.6.3.1 의과대학은 교육활동에 정보통신기술을 사용하고 있으며, 구성원이 전자교육매체를 활용할 수 있도록 지원하고 있다.**

*자체평가연구보고서 작성 가이드*

가이드-1. 교육과정에서 적절한 정보통신기술이 효과적으로 사용되고 있다.
가이드-2. 의과대학은 학술정보서비스 체제가 구축되어 있다.
가이드-3. 학술도서 및 학술지의 열람 및 출력이 용이하다.
가이드-4. 학술정보서비스 예산이 적절하다.
가이드-5. 학술정보서비스 전문인력(의학전담사서)이 있다.
가이드-6. 학술정보서비스의 접근성이 용이하다.

*방문평가단 평가 가이드*

| 기준 | 핵심어 | 가이드 | 근거자료 |
|---|---|---|---|
| K.6.3.1 | • 정보통신기술<br>• 전자교육매체<br>• 의학도서관<br>• 학술정보서비스 | • 교육과정에서 적절한 정보통신기술이 효과적으로 사용되는지 확인한다.<br>• 의과대학은 학술정보서비스 체제가 구축되어 있는지 확인한다(단, 대학 본부 중앙도서관에 포함된 경우 의과대학 교수 및 학생을 위한 독립적인 학술정보서비스를 제공함).<br>• 학술도서 및 학술지의 열람 및 출력이 용이한지 확인한다.<br>• 학술정보서비스 예산이 적절한지 확인한다.<br>• 학술정보서비스 전문인력(의학전담사서)이 있는지 확인한다.<br>• 학술정보서비스의 접근성이 용이한지 확인한다. | • 현장 확인<br>• 교수 면담<br>• 학생 면담<br>• 관련규정<br>• 의학도서관 학술지원서비스<br>• 학내 정보지원시스템 자료<br>• 학술정보서비스 체계 확인(하드웨어 및 소프트웨어)<br>• 주요 학술지 검색 및 출력 확인<br>• 학술정보서비스 관련 예결산 자료 |

**[주]**

- 정보와 통신기술에 대한 효과적이고 윤리적인 이용은 컴퓨터, 교내나 외부 네트워크, 기타 수단들의 이용에 대한 고려를 포함한다. 여기에는 도서관 자료와 그 기관의 IT 서비스의 협력을 포함한다. 그리고 학습관리시스템을 통하여 모든 교육항목에 공통적으로 접근할 수 있는 것을 포함한다.

  정보와 통신기술은 전문직업성 평생개발(CPD)을 통해 학생들을 근거중심 의학과 평생 학습에 대해 준비시키는데 활용한다.
- 적절한 학술정보서비스 예산은 교수와 학생 1인당 연 50만 원 이상이다.

## 우수기준

**H.6.3.1 의과대학은 교육자와 학생들이 자율학습을 위해 정보통신기술을 사용할 수 있도록 지원하고 있다.**

| *자체평가연구보고서 작성 가이드* |
|---|
| 가이드-1. 자율학습을 위해 정보통신기술을 사용할 수 있다.<br>가이드-2. 학술정보서비스 관련 예결산이 적절하다.<br>가이드-3. 학술정보서비스 관련 전문인력 지원이 적절하다.<br>가이드-4. 이러닝 시스템을 활용하고 있다. |

*방문평가단 평가 가이드*

| 기준 | 핵심어 | 가이드 | 근거자료 |
|---|---|---|---|
| H.6.3.1 | • 정보통신기술 | • 자율학습을 위해 정보통신기술을 사용할 수 있는지 확인한다.<br>• 학술정보서비스 관련 예결산이 적절한지 확인한다.<br>• 학술정보서비스 관련 전문인력 지원이 적절한지 확인한다.<br>• 이러닝 시스템(상호작용 및 학습관리 등)을 활용한 교육을 제공하고 있는지 확인한다. | • 의학도서관 학술정보서비스<br>• 학술정보서비스 관련 예결산 자료<br>• 학내 정보지원시스템 자료<br>• 교수 및 학생 수 현황과 전문인력 현황표<br>• 이러닝 시스템 활용자료<br>• 학생 면담 |

**[주]**

- 적절한 학술정보서비스 관련 예결산은 교수와 학생 1인당 연 150만 원 이상이다.
- 적절한 학술정보서비스 관련 전문인력 지원은 교수와 학생 150명 당 1인 이상이다.

**우수기준**

**H.6.3.2 의과대학은 학생들이 환자의 자료와 관련된 병원정보시스템에 접근할 수 있도록 지원하고 있다.**

*자체평가연구보고서 작성 가이드*

가이드-1. 학생에게 주어진 권한 내에서 OCS, EMR, PACS 시스템을 사용할 수 있다.

*방문평가단 평가 가이드*

| 기준 | 핵심어 | 가이드 | 근거자료 |
|---|---|---|---|
| H.6.3.2 | • 병원정보시스템 접근성 | • 학생에게 주어진 권한 내에서 OCS, EMR, PACS 시스템을 사용할 수 있는지 확인한다. | • 환자정보 접근 관련 근거자료<br>• 학생 면담 |

## 6.4 의학연구와 의과학자 양성

### 기본기준

**K.6.4.1 의과대학은 의학연구역량을 개발하는 정책이 있다.**

| *자체평가연구보고서 작성 가이드* |
|---|
| 가이드-1. 학생의 의학연구역량을 개발하는 정책이 있다 |

*방문평가단 평가 가이드*

| 기준 | 핵심어 | 가이드 | 근거자료 |
|---|---|---|---|
| K.6.4.1 | • 의학연구역량 개발 정책 | • 학생의 의학연구역량을 개발하는 정책이 있다 | • 정책 관련 자료 |

### 기본기준

**K.6.4.2 의과대학은 연구역량을 개발할 수 있는 교육과정을 운영하고 있다.**

| *자체평가연구보고서 작성 가이드* |
|---|
| 가이드-1. 연구역량을 개발할 수 있는 교육과정이 있다. |

*방문평가단 평가 가이드*

| 기준 | 핵심어 | 가이드 | 근거자료 |
|---|---|---|---|
| K.6.4.2 | • 의학연구 교육과정 | • 연구역량을 개발할 수 있는 교육과정이 있는지 확인한다. | • 수업계획서 및 실적 |

## 기본기준

### K.6.4.3 의과대학은 학생의 연구시설 사용에 대하여 규정하고 있다.

*자체평가연구보고서 작성 가이드*

가이드-1. 학생의 의학연구에 적합한 연구 시설이 있다.
가이드-2. 학생이 연구시설을 사용한 실적이 있다.

*방문평가단 평가 가이드*

| 기준 | 핵심어 | 가이드 | 근거자료 |
|---|---|---|---|
| K.6.4.3 | • 학생의 의학연구<br>• 의과학자 양성 | • 학생의 의학연구에 적합한 연구 시설이 있는지 확인한다.<br>• 학생이 연구시설을 사용한 실적이 있는지 확인한다. | • 관련 규정<br>• 연구시설 목록<br>• 연구시설 사용 실적 |

## 우수기준

### H.6.4.1 의과대학은 의학연구와 교육 간의 상호작용이 가능하도록 지원하며, 학생의 의학연구 참여를 장려하고 있다.

*자체평가연구보고서 작성 가이드*

가이드-1. 교육적 요구에 의해 의학연구가 진행된 실적이 있다.
가이드-2. 학생의 의학연구 참여를 장려한 실적이 있다.

*방문평가단 평가 가이드*

| 기준 | 핵심어 | 가이드 | 근거자료 |
|---|---|---|---|
| H.6.4.1 | • 의학연구와 교육 간의 상호작용<br>• 학생 의학연구 참여 | • 교육적 요구에 의해 의학연구가 진행된 실적이 있는지 확인한다.<br>• 학생의 의학연구 참여를 장려한 실적이 있는지 확인한다. | • 관련 실적<br>• 학생 연구실적 |

**[주]**

- 의학연구와 의과학자 양성과정은 기초의학, 의료인문학, 임상의학의 과학적 연구를 포함한다. 의과학자 양성은 첨단 의학지식과 탐구의 학문적 획득을 의미한다. 교육과정에 근거한 의학연구는 의과대학 자체 내 또는 협력관계에 있는 기관들의 연구 활동과 교수진의 과학적 역량과 연구에 의하여 확보된다. 현재 교육에 대한 영향은 과학적 방법(K.2.2.1 참조)과 근거중심의학(K.2.2.3 참조)의 교육을 촉진시킨다.

## 6.5 교육 전문성

### 기본기준

**K.6.5.1 의과대학은 필요한 분야에 대한 교육 전문성을 확보하고 있다.**

| *자체평가연구보고서 작성 가이드* |
|---|
| 가이드-1. 의과대학은 학생, 교수가 요구하는 분야에 대한 교육 전문가들을 확보하고 있다.<br>가이드-2. 교육에 필요한 분야에 충분한 경력을 갖춘 교육 전문가의 의견을 얻는 것이 용이하다.<br>가이드-3. 교육에 필요한 분야에 대한 교육관련 정보에 쉽게 접근할 수 있다. |

*방문평가단 평가 가이드*

| 기준 | 핵심어 | 가이드 | 근거자료 |
|---|---|---|---|
| K.6.5.1 | • 교육 전문성 | • 의과대학은 학생, 교수가 요구하는 분야에 대한 교육 전문가들을 확보하고 있는지 확인한다.<br>• 교육에 필요한 분야에 충분한 경력을 갖춘 교육 전문가를 만나거나 의견을 얻는 것이 용이한지 확인한다.<br>• 교육에 필요한 분야에 대한 교육관련 정보에 쉽게 접근할 수 있는지 확인한다. | • 교수 면담<br>• 교수개발 실적 자료 |

**[주]**

- 교육 전문성(educational expertise)은 의학 교육의 과정, 실행, 문제점을 다루고, 의학교육에 대한 연구 경험이 있는 의사, 교육학자, 사회학자 등을 포함할 수 있다. 또한 교육 전문성은 의과대학의 교육개발부서나 대학 내의 경험과 관심이 있는 교수자에 의해 제공되거나 국내외 다른 기관에서 제공될 수 있다.

## 기본기준

**K.6.5.2 의과대학은 교육과정 개발, 교수법과 평가방법 개발에 대한 교육 전문성 활용에 관한 정책을 수립하고 실행하고 있다.**

*자체평가연구보고서 작성 가이드*

가이드-1. 교육과정을 개발할 때, 교육 전문성을 활용하도록 하는 정책과 실적이 있다.
가이드-2. 교수법을 개발할 때, 교육 전문성을 활용하도록 하는 정책과 실적이 있다.
가이드-3. 평가방법을 개발할 때, 교육 전문성을 활용하도록 하는 정책과 실적이 있다.

*방문평가단 평가 가이드*

| 기준 | 핵심어 | 가이드 | 근거자료 |
|---|---|---|---|
| K.6.5.2 | • 교육 전문성 활용 | • 교육과정을 개발할 때, 교육 전문성을 활용하도록 하는 정책과 실적이 있는지 확인한다.<br>• 교수법을 개발할 때, 교육 전문성을 활용하도록 하는 정책과 실적이 있는지 확인한다.<br>• 평가방법을 개발할 때, 교육 전문성을 활용하도록 하는 정책과 실적이 있는지 확인한다. | • 교육 전문성 관련 규정<br>• 실적 자료 |

## 우수기준

**H.6.5.1 의과대학은 교직원의 역량개발을 위해 교내외의 교육 전문성을 활용한 실적이 있다.**

*자체평가연구보고서 작성 가이드*

가이드-1. 교직원의 역량개발을 위해 교내외 교육 전문가나 교육 전문기구를 활용한 실적이 있다.

*방문평가단 평가 가이드*

| 기준 | 핵심어 | 가이드 | 근거자료 |
|---|---|---|---|
| H.6.5.1 | • 교직원 개발 | • 교직원의 역량개발을 위해 교내외 교육 전문가나 교육 전문기구를 활용한 실적이 있는지 확인한다. | • 교육 전문성 활용을 위한 근거자료<br>• 교직원 면담 |

**[주]**

- 교직원에는 교수와 교육, 연구, 행정, 기술 등에 관련된 직원을 포함한다.

### 우수기준

## H.6.5.2 의과대학은 교육평가와 의학교육학의 연구 분야에서의 전문성을 개발하고 있다.

*자체평가연구보고서 작성 가이드*

가이드-1. 교육평가의 전문성을 개발한 실적이 있다.
가이드-2. 의학교육학 연구 분야의 전문성을 개발한 실적이 있다.

*방문평가단 평가 가이드*

| 기준 | 핵심어 | 가이드 | 근거자료 |
|---|---|---|---|
| H.6.5.2 | • 교육평가 연구<br>• 의학교육학 연구<br>• 전문성 개발 | • 교육평가의 전문성을 개발한 실적이 있는지 확인한다.<br>• 의학교육학 연구 분야의 전문성을 개발한 실적이 있는지 확인한다. | • 관련 실적 |

**[주]**

- 의학교육학 연구에서는 의학교육에 있어 이론적, 실제적, 사회적 문제를 조사한다.

### 우수기준

## H.6.5.3 의과대학은 교수들이 의학교육 연구에 관심을 갖도록 지원하고 있다.

*자체평가연구보고서 작성 가이드*

가이드-1. 교수들이 교육관련 연구에 관심을 갖도록 지원하는 정책이 있다.
가이드-2. 교육관련 논문 또는 보고서 실적이 있다.

*방문평가단 평가 가이드*

| 기준 | 핵심어 | 가이드 | 근거자료 |
|---|---|---|---|
| H.6.5.3 | • 교수<br>• 의학교육 | • 교수들이 교육관련 연구에 관심을 갖도록 지원하는 정책이 있는지 확인한다.<br>• 교육관련 논문 또는 보고서 실적이 있는지 확인한다. | • 지원 관련 규정<br>• 교육관련 논문 또는 보고서 실적 |

## 6.6 교육적 교류

### 기본기준

**K.6.6.1 의과대학은 교직원과 학생이 국내외의 교육 기관과 교류가 이루어질 수 있도록 적절하게 지원하고 있다.**

| *자체평가연구보고서 작성 가이드* |
|---|
| 가이드-1. 교직원과 학생이 국내외의 교육 기관과 교류할 수 있도록 적절하게 지원하고 있다.<br>가이드-2. 교직원과 학생이 국내외의 교육 기관과 활발히 교류한 실적이 있다. |

*방문평가단 평가 가이드*

| 기준 | 핵심어 | 가이드 | 근거자료 |
|---|---|---|---|
| K.6.6.1 | • 교류 | • 교직원과 학생이 국내외의 교육 기관과 교류할 수 있도록 적절하게 지원하고 있는지 확인한다.<br>• 교직원과 학생이 국내외의 교육 기관과 활발히 교류한 실적이 있는지 확인한다. | • 교직원 면담<br>• 학생 면담<br>• 국내외 교류 관련 자료<br>• 관련 규정 |

**[주]**

- 다른 교육 기관은 타 의과대학, 공중보건학, 치의학, 약학, 수의학을 교육하는 학교와 같은 보건의료 교육 시설과 기관을 포함한다.

## 우수기준

### H.6.6.1 의과대학은 국내외 여러 교육기관과의 협력을 도모하고 타 교육기관에서 취득한 학점을 인정하는 정책을 수립하고 실행하고 있다.

*자체평가연구보고서 작성 가이드*

가이드-1. 여러 교육기관과 협력을 도모한 실적이 있다.
가이드-2. 타 교육기관에서 취득한 학점을 인정하는 정책이 있다.
가이드-3. 타 교육기관에서 취득한 학점을 인정한 실적이 있다.

*방문평가단 평가 가이드*

| 기준 | 핵심어 | 가이드 | 근거자료 |
|---|---|---|---|
| H.6.6.1 | • 타 교육기관<br>• 협력 | • 여러 교육기관과 협력을 도모한 실적이 있는지 확인한다.<br>• 타 교육기관에서 취득한 학점을 인정하는 정책이 있는지 확인한다.<br>• 타 교육기관에서 취득한 학점을 인정한 실적이 있는지 확인한다. | • 관련 규정<br>• 관련 서류<br>• 집행실적 |

**[주]**

- 타 교육기관 학점인정에 대한 정책은 다른 교육기관으로부터 취득할 수 있는 학습 프로그램 비율의 제한을 둔다. 타 교육기관 학점인정은 의과대학 간의 활발한 프로그램 협력과 교육적 요소의 상호 인정에 대한 협약을 수립함으로써 활성화할 수 있다. 또한 타 교육기관 학점인정은 투명한 학점 관리 시스템을 사용하고 교과목의 요구조건에 대한 유연성 있는 해석을 통해 활성화할 수 있다.

ASK2019

# 07 교육평가

## 7.1 교육 모니터링과 평가에 대한 체제

### 기본기준

**K.7.1.1 의과대학은 교육의 과정에 대한 정기적인 모니터링을 하고 있다.**

| *자체평가연구보고서 작성 가이드* |
| --- |
| 가이드-1. 입학에서 졸업까지의 교육의 과정(educational process)을 정기적으로 모니터링하고 있다. |

*방문평가단 평가 가이드*

| 기준 | 핵심어 | 가이드 | 근거자료 |
| --- | --- | --- | --- |
| K.7.1.1 | • 교육의 과정<br>• 모니터링 | • 입학에서 졸업까지의 교육의 과정(educational process)을 정기적으로 모니터링하고 있는지 확인한다. | • 모니터링 실적<br>• 관련 위원회 회의록 등 |

**[주]**

- 교육평가(programme evaluation)는 대학과 대학 교육의 과정의 효과와 적절성을 판단하기 위한 체계적 정보수집의 과정이다. 교육평가에는 사명, 교육과정, 학생의 향상 정도와 의도한 교육성과에 관련된 프로그램의 핵심 측면과 교육프로그램의 질을 나타내거나 입증할 목적으로 신뢰할만하고 타당한 자료수집과 분석방법을 사용하는 것을 포함한다.
- 교육 모니터링(programme monitoring)은 교육의 과정(educational process)이 정상적으로 운영되고 있다는 것과 중재가 필요한 모든 영역을 확인할 목적으로 교육과정의 핵심 측면에 관한 자료의 정기적인 수집을 포함한다. 자료의 수집은 학생의 입학, 평가, 졸업과 관련한 행정적인 절차의 일부로 종종 이루어진다.
- 교육의 과정(educational process)은 교육과정을 포함한 학생의 입학, 평가, 졸업 등 의과대학의 교육과 관련된 시스템과 모든 활동을 의미하며 의과대학의 학습 환경과 문화뿐만 아니라 조직과 자원을 포함한다.

## 기본기준

### K.7.1.2 의과대학은 교육평가체제를 수립하여 시행하고 있다.

*자체평가연구보고서 작성 가이드*

가이드-1. 교육평가체제를 가지고 있다.
가이드-2. 교육과정과 교육과정의 주요 구성요소를 평가하고 있다.
가이드-3. 학생의 향상 정도를 평가하고 있다.
가이드-4. 대학에서 의도한 교육성과에 도달하지 못한 주요 관심분야를 평가하고 있다.

*방문평가단 평가 가이드*

| 기준 | 핵심어 | 가이드 | 근거자료 |
|---|---|---|---|
| K.7.1.2 | • 교육평가체제<br>• 교육과정<br>• 교육과정의 주요 구성요소<br>• 학생의 향상정도<br>• 주요 관심분야 | • 교육평가체제를 가지고 있는지 확인한다.<br>• 교육과정과 교육과정의 주요 구성요소를 평가하고 있는지 확인한다.<br>• 학생의 향상 정도를 평가하고 있는지 확인한다.<br>• 대학에서 의도한 교육성과에 도달하지 못한 주요 관심분야를 평가하고 있는지 확인한다. | • 교육평가체제(규정)<br>• 운영실적 |

**[주]**

- 교육평가체제는 교육평가에 대한 규정, 내규, 지침 등이 있고, 이를 실행하는 담당기구(교실, 실, 학과, 센터)가 있는 것을 의미한다.
- 교육과정의 주요 구성요소에는 교육과정 구조, 구성, 기간(2.6 참조), 필수과목과 선택과목의 사용(H.2.6.2 참조) 등을 포함한다.
- 주요 관심분야는 의도한 교육성과의 불충분한 성취를 의미하고, 의도한 교육성과를 달성하기 위한 과정에서 확인된 불충분한 성취, 약점, 문제점 등을 포함하여 교육과정 개선을 위한 계획으로 활용한다.

## 기본기준

### K.7.1.3 의과대학은 교육평가 결과를 교육과정 개선에 활용하고 있다.

*자체평가연구보고서 작성 가이드*

가이드-1. 교육의 과정(educational process)에 대한 평가결과를 교육의 과정 개선에 활용한 실적이 있다.
가이드-2. 교육과정과 교육과정 구성요소에 대한 평가결과를 교육과정 개선에 활용한 실적이 있다.
가이드-3. 학생 향상정도에 대한 평가결과를 교육과정 개선에 활용한 실적이 있다.
가이드-4. 의도한 교육성과에 도달하지 못한 부분에 대한 평가결과를 교육과정 개선에 활용한 실적이 있다.

*방문평가단 평가 가이드*

| 기준 | 핵심어 | 가이드 | 근거자료 |
|---|---|---|---|
| K.7.1.3 | • 교육과정 개선 반영 | • 교육의 과정(educational process)에 대한 평가결과를 교육의 과정에 반영한 실적이 있는지 확인한다.<br>• 교육과정과 교육과정 구성요소에 대한 평가결과를 교육과정에 반영한 실적이 있는지 확인한다.<br>• 학생 향상정도에 대한 평가결과를 교육과정에 반영한 실적이 있는지 확인한다.<br>• 의도한 교육성과에 도달하지 못한 항목에 대한 평가결과를 교육과정에 반영한 실적이 있는지 확인한다. | • 개선실적 관련 자료 |

## 우수기준

**H.7.1.1 의과대학은 교육의 과정 맥락, 교육과정의 특정 구성 요소, 장기성과, 대학의 사회적 책무성을 반영하여 주기적으로 교육평가의 체제를 수립하고 적용하고 있다.**

*자체평가연구보고서 작성 가이드*

가이드-1. 교육평가를 위한 제도(위원회 또는 기구, 규정 등)가 있다.
가이드-2. 교육의 과정 맥락에 맞추어 주기적으로 평가한다.
가이드-3. 교육과정의 특정 구성요소에 맞추어 주기적으로 평가한다.
가이드-4. 장기성과에 맞추어 주기적으로 평가한다.
가이드-5. 대학의 사회적 책무에 맞추어 주기적으로 평가한다.

*방문평가단 평가 가이드*

| 기준 | 핵심어 | 가이드 | 근거자료 |
|---|---|---|---|
| H.7.1.1 | • 교육의 과정 맥락<br>• 교육과정의 특정 구성요소<br>• 장기성과<br>• 대학의 사회적 책무성<br>• 교육의 과정 평가 | • 교육의 과정 맥락에 맞추어 주기적으로 평가하는지 확인한다.<br>• 교육과정의 특정 구성요소에 맞추어 주기적으로 평가하는지 확인한다.<br>• 장기성과에 맞추어 주기적으로 평가하는지 확인한다.<br>• 대학의 사회적 책무에 맞추어 주기적으로 평가하는지 확인한다. | • 관련 위원회 규정<br>• 위원회 활동 자료<br>• 평가실적 |

**[주]**

- 교육의 과정(educational process)맥락에는 의과대학의 학습 환경과 문화뿐만 아니라 조직과 자원을 포함한다.
- 교육과정의 특정 구성요소에는 과정설명, 교수학습법, 임상실습 순환과 평가방법을 포함한다.
- 장기성과는 예를 들어 의사국가시험, 벤치마킹수행, 국제시험, 진로 선택 그리고 졸업 후 수행에서 나온 결과에 의해 측정되며, 전반적인 성과를 통해 프로그램의 획일성에 대한 위험을 피하면서 교육과정 개선을 위한 기초를 제공한다.
- 사회적 책무는 K.1.1.3 참조.

## 7.2 교육자와 학생의 피드백

### 기본기준

**K.7.2.1 의과대학은 교육자와 학생으로부터의 피드백을 체계적으로 조사, 분석하여 대응하고 있다.**

| *자체평가연구보고서 작성 가이드* |
|---|
| 가이드-1. 교육자(교수, 전공의 등)와 학생의 피드백을 체계적으로 조사, 분석한다.<br>가이드-2. 개선점, 개선계획 등에 대하여 알린다. |

*방문평가단 평가 가이드*

| 기준 | 핵심어 | 가이드 | 근거자료 |
|---|---|---|---|
| K.7.2.1 | • 교육자와 학생으로부터의 피드백 | • 교육자와 학생의 피드백을 체계적으로 조사, 분석하여 대응하는지 확인한다. | • 교육자와 학생의 피드백 분석자료 및 통보 실적 |

### 우수기준

**H.7.2.1 의과대학은 피드백 결과를 교육 프로그램 개발에 활용하고 있다.**

| *자체평가연구보고서 작성 가이드* |
|---|
| 가이드-1. 피드백 결과를 바탕으로 교육 프로그램 개발에 활용한다. |

*방문평가단 평가 가이드*

| 기준 | 핵심어 | 가이드 | 근거자료 |
|---|---|---|---|
| H.7.2.1 | • 피드백 결과의 활용 | • 피드백 결과를 바탕으로 교육 프로그램 개발에 활용하는지 확인한다. | • 관련 실적 |

## 7.3 학생과 졸업생의 수행 능력

### 기본기준

**K.7.3.1 의과대학은 학생과 졸업생 코호트에서 사명, 의도한 교육성과, 교육과정, 투입한 자원에 관련된 수행능력을 분석하고 있다.**

| *자체평가연구보고서 작성 가이드* |
|---|
| 가이드-1. 학생과 졸업생 코호트를 관리하는 제도가 있다.<br>가이드-2. 학생과 졸업생 코호트에서 사명, 의도한 교육성과, 교육과정, 투입한 자원과 관련된 수행능력을 평가한다. |

*방문평가단 평가 가이드*

| 기준 | 핵심어 | 가이드 | 근거자료 |
|---|---|---|---|
| K.7.3.1 | • 학생과 졸업생 코호트<br>• 수행능력 | • 학생과 졸업생 코호트를 관리하는 제도가 있는지 확인한다.<br>• 학생과 졸업생 코호트에서 사명, 의도한 교육성과, 교육과정, 투입한 자원과 관련한 수행능력을 평가하는 지표와 방법을 가지고 있고, 그 실적이 있다. | • 학생과 졸업생 코호트 관리체제<br>• 학생과 졸업생 수행능력 분석자료 |

**[주]**

- 학생 코호트의 수행능력 측정과 평가에는 선택적인 구성요소를 포함한, 특별 관심분야에 학생들이 쏟은 시간뿐만 아니라 실제 학습기간, 시험점수, 합격과 낙제 비율, 성공과 중도탈락 비율과 이유, 그리고 과정의 상황에 관한 학생의 보고서에 대한 정보를 포함한다. 또한 유급생에 대한 인터뷰와 제적생의 제적직전 인터뷰도 포함한다.
- 졸업생 코호트의 수행능력 측정은 의사국가고시 결과, 진로 선택, 졸업 후 역량에 대한 정보를 포함하고, 획일화된 교육과정의 위험성을 회피할 수 있는 교육과정개편을 위한 기초자료를 제공한다.

**우수기준**

**H.7.3.1 의과대학은 학생의 수행능력 내용을 분석하여 학생선발, 교육과정 기획, 학생상담 관련 위원회에 피드백을 하고 있다.**

*자체평가연구보고서 작성 가이드*

가이드-1. 학생 수행능력 내용을 분석하여 학생선발, 교육과정 기획, 학생상담 관련 위원회에 피드백을 하는 제도가 있다.
가이드-2. 학생선발, 교육과정 기획, 학생상담 관련 위원회 등은 학생의 수행능력을 분석한 피드백 자료를 활용하여 개선한다.

*방문평가단 평가 가이드*

| 기준 | 핵심어 | 가이드 | 근거자료 |
|---|---|---|---|
| H.7.3.1 | • 관련 위원회에 피드백 | • 학생 수행능력 내용을 분석하여 학생선발, 교육과정 기획, 학생상담 관련 위원회에 피드백을 하는 제도가 있는지 확인한다.<br>• 학생선발, 교육과정 기획, 학생상담 관련 위원회 등은 학생의 수행능력을 분석한 피드백 자료를 활용하여 개선한 실적이 있다. | • 위원회 활동자료<br>• 피드백자료<br>• 피드백 자료 활용 및 개선 실적 |

## 7.4 이해관계자의 참여

### 기본기준

**K.7.4.1 의과대학은 교육 모니터링과 교육평가 활동에 주요 이해관계자를 포함하고 있다.**

| *자체평가연구보고서 작성 가이드* |
| --- |
| 가이드-1. 교육 모니터링과 교육평가 활동에 주요 이해관계자를 참여시킨다. |

*방문평가단 평가 가이드*

| 기준 | 핵심어 | 가이드 | 근거자료 |
| --- | --- | --- | --- |
| K.7.4.1 | • 주요 이해관계자 | • 교육 모니터링과 교육평가 활동에 주요이해관계자를 참여시킨 실적을 확인한다. | • 주요 이해관계자 명단<br>• 주요 이해관계자 참여 실적<br>• 주요 이해관계자 면담 |

**[주]**

- 주요 이해관계자는 K.1.4.1 참조.

ASK2019

# 08 대학운영체계와 행정

## 8.1 대학운영체계

### 기본기준

**K.8.1.1 의과대학은 교육, 교수, 학생의 업무가 분장되어 있으며 별도의 보직자가 임명되어 있다.**

| *자체평가연구보고서 작성 가이드* |
|---|
| 가이드-1. 대학 행정업무가 구분되어 있다.<br>가이드-2. 교육, 학생, 교수 영역의 보직자가 독립적으로 임명되어 있다. |

*방문평가단 평가 가이드*

| 기준 | 핵심어 | 가이드 | 근거자료 |
|---|---|---|---|
| K.8.1.1 | • 행정<br>• 보직자 | • 대학 행정업무가 구분되어 있는지 확인한다.<br>• 교육, 학생, 교수 영역의 보직자가 독립적으로 임명되어 있는지 확인한다. | • 대학 행정업무 체계도<br>• 행정업무의 고유임무에 대한 규정<br>• 분야별 활동실적 보고서, 회의록 또는 보직수당 지급 내역 |

**[주]**

- 대학운영체계는 의과대학을 관리하는 지배구조와 집행을 의미한다. 대학운영체계는 주로 정책결정, 일반기관과 프로그램정책 수립하는 과정, 그리고 정책실행 제어와 관련되어 있다. 기관과 프로그램의 정책은 일반적으로 의과대학의 사명, 교육과정, 입학정책, 직원채용과 선발정책, 의료행위, 보건의료분야와 외부 관계자들의 상호작용과 연계에 관한 결정을 포함한다.
- 대학운영에 필요한 보직자의 행정업무는 교육, 학생, 교수영역을 반드시 포함하여 구분되어야 하며, 각 분야별 규정과 실적이 포함되어야 한다.

## 기본기준

### K.8.1.2 의과대학은 학장과 보직자가 직무관련 전문성을 확보할 수 있는 체제와 실적이 있다.

| *자체평가연구보고서 작성 가이드* |
|---|
| 가이드-1. 보직 해당분야의 전문성 확보를 위한 제도와 실적이 있다. |

*방문평가단 평가 가이드*

| 기준 | 핵심어 | 가이드 | 근거자료 |
|---|---|---|---|
| K.8.1.2 | • 학장<br>• 보직자<br>• 전문성 | • 보직 해당분야의 전문성 확보를 위한 제도와 실적이 있는지 확인한다. | • 관련 규정<br>• 전문성 개발 실적<br>• 교수 면담 |

## 기본기준

### K.8.1.3 의과대학은 대학운영을 위한 적절한 정책결정 구조와 절차를 갖추고 있다.

| *자체평가연구보고서 작성 가이드* |
|---|
| 가이드-1. 대학운영을 위한 분명한 정책결정 구조와 절차가 있다.<br>가이드-2. 교육, 학생지도, 인사위원회가 구성되어 있고 정기적으로 개최되고 있다. |

*방문평가단 평가 가이드*

| 기준 | 핵심어 | 가이드 | 근거자료 |
|---|---|---|---|
| K.8.1.3 | • 대학운영<br>• 정책결정 | • 대학운영을 위한 분명한 정책결정 구조와 절차가 있는지 확인한다.<br>• 교육, 학생지도, 인사위원회가 구성되어 있고 적절하게 개최되었는지 확인한다. | • 실적 보고서<br>• 관련 규정<br>• 조직 기구도<br>• 위원회 운영규정과 회의록 |

**[주]**

- 대학운영 정책결정을 위한 각종 회의 및 위원회는 규정이 명시되어 있어야 한다. 인사위원회의 경우 선출직이 1/3 이상을 차지하며, 기타 위원회의 경우 당연직 위원이 1/4 이내로 구성되어야 한다. 각 위원회는 정기적으로 개최된 실적이 있어야 한다.

기본기준

**K.8.1.4 의과대학은 지리적으로 떨어져 있는 교육병원마다 학생교육과 연구를 총괄하는 보직자가 있고 교육과 연구를 지원하는 행정체계를 갖추고 있다.**

*자체평가연구보고서 작성 가이드*

가이드-1. 지리적으로 떨어진 교육병원마다 학생실습을 총괄하는 보직자가 있다.
가이드-2. 교육병원에 학생들의 교육과 연구를 지원하는 행정구조가 있다.
가이드-3. 정기적인 회의와 학장과 보직자 방문실적이 있다.

*방문평가단 평가 가이드*

| 기준 | 핵심어 | 가이드 | 근거자료 |
|---|---|---|---|
| K.8.1.4 | • 교육병원<br>• 보직자<br>• 행정구조 | • 지리적으로 떨어진 교육병원마다 학생실습을 총괄하는 보직자가 있는지 확인한다.<br>• 교육병원마다 학생들의 교육과 연구를 지원하는 행정구조가 있는지 확인한다.<br>• 정기적인 회의와 학장과 보직자 방문실적이 있는지 확인한다. | • 교육병원별 교육과 연구지원 행정구조 및 업무분장<br>• 학생 출입현황 자료<br>• 학장단 방문 근거자료<br>• 회의자료 |

**[주]**

- 지리적으로 떨어진 교육병원에 연간 2회 이상의 학장과 보직자 방문실적이 있어야 한다.

## 우수기준

**H.8.1.1 의과대학은 대학운영을 위한 위원회에 주요 이해관계자, 기타 이해관계자의 대표를 포함하고 있다.**

| *자체평가연구보고서 작성 가이드* |
|---|
| 가이드-1. 위원회에 주요 이해관계자를 포함한다.<br>가이드-2. 위원회에 기타 이해관계자를 포함한다. |

*방문평가단 평가 가이드*

| 기준 | 핵심어 | 가이드 | 근거자료 |
|---|---|---|---|
| H.8.1.1 | • 대학운영체계<br>• 주요 이해관계자<br>• 기타 이해관계자 | • 대학운영을 위한 위원회에 교육과 연구지원 직원을 포함하고 있는지 확인한다.<br>• 대학운영을 위한 위원회에 학생을 포함하고 있는지 확인한다.<br>• 대학운영을 위한 위원회에 기타 이해관계자를 포함하고 있는지 확인한다. | • 위원회 관련 규정<br>• 위원회 회의자료 |

**[주]**

- 위원회는 권한과 책임이 있어야 하며, 교육과정위원회가 포함되어야 한다(K.2.7.1 참조).
- 주요 이해관계자는 K.1.4.1 참조.
- 기타 이해관계자는 H.1.4.1 참조.

## 우수기준

### H.8.1.2 의과대학은 대학운영의 전문성을 확보하고 의사결정과정에서 투명성을 보장하고 있다.

| *자체평가연구보고서 작성 가이드* |
|---|
| 가이드-1. 대학운영체계에서 교육, 학생, 교수, 연구분야를 포함한 적절한 분야의 보직자와 활동실적이 있다.<br>가이드-2. 대학운영과 의사결정과정에서 투명성을 보장하고 있다. |

*방문평가단 평가 가이드*

| 기준 | 핵심어 | 가이드 | 근거자료 |
|---|---|---|---|
| H.8.1.2 | • 대학운영의 전문성<br>• 의사결정과정의 투명성 | • 대학운영체계에서 적절한 분야의 보직자와 활동 실적이 있는지 확인한다.<br>• 대학운영과 의사결정과정에서 투명성을 보장하고 있는지 확인한다. | • 운영규정<br>• 행정업무의 고유임무에 대한 규정<br>• 분야별 활동실적 보고서 또는 회의록(보직수당 지급내역) |

**[주]**

- 투명성은 소식지, 웹 정보 혹은 회의록 공개를 통해 확보한다.
- 대학운영체계에는 교육, 학생, 교수, 연구분야를 포함한 최소 7개 분야에 보직자가 있어야 한다.

## 8.2 학장과 보직자

### 기본기준

**K.8.2.1 의과대학은 교육의 전반적인 관리에 관한 학장과 보직자의 책임을 명시하고 있다.**

| *자체평가연구보고서 작성 가이드* |
|---|
| 가이드-1. 의학교육의 전반적인 관리에 관한 의과대학 학장과 보직자의 책임을 명시하고 있다.<br>가이드-2. 대학관련 규정에 학장의 업무와 예산집행에 관한 사항이 명기되어 있다.<br>가이드-3. 학장이 대학의 인사관련 위원회 및 기구를 적절하게 운영하고 있다. |

*방문평가단 평가 가이드*

| 기준 | 핵심어 | 가이드 | 근거자료 |
|---|---|---|---|
| K.8.2.1 | • 학장<br>• 보직자<br>• 책임 | • 의학교육의 전반적인 관리를 위해 의과대학 학장과 보직자의 책임을 명시하고 있는지 확인한다.<br>• 대학관련 규정에 학장의 업무와 예산집행에 관한 사항이 명기되어 있는지 확인한다.<br>• 학장이 대학의 인사관련 위원회 및 기구를 적절하게 운영하고 있는지 확인한다. | • 관련 규정<br>• 실적 보고서<br>• 대학의 전결규정 |

**[주]**

- 학장과 보직자는 교육, 연구, 서비스에 있어 학사적인 문제에 관한 의사결정에 책임이 있는 대학운영체계와 관리구조 내의 보직과 책임자를 의미하며, 여기에는 학장, 부학장, 학과장, 교무처장, 부서장, 과정 리더, 연구소장, 센터장, 또한 상임위원회의 위원장이 포함된다(예: 학생선발위원회, 교육과정기획위원회, 학생상담위원회).
- 학장의 권한과 책임, 인사권에 대한 규정이 명시되어야 하며, 관련 활동자료가 포함되어야 한다.

## 우수기준

### H.8.2.1 의과대학은 학장 선임규정이 있고 선임된 학장과 보직자를 주기적으로 평가하고 있다.

*자체평가연구보고서 작성 가이드*

가이드-1. 교육, 학생, 교수, 연구 분야를 포함한 리더십 개발 경력을 학장 선임대상자 자격요건으로 규정하고 있다.

가이드-2. 사명과 의도한 교육성과의 성취와 관련, 학장과 보직자를 정기적으로 평가할 수 있는 체계와 실적이 있다.

*방문평가단 평가 가이드*

| 기준 | 핵심어 | 가이드 | 근거자료 |
|---|---|---|---|
| H.8.2.1 | • 학장 자격요건<br>• 학장과 보직자 평가 | • 교육, 학생, 교수, 연구 분야를 포함한 리더십 개발 경력을 학장 선임대상자 자격요건으로 규정하고 있는지 확인한다.<br>• 사명과 의도한 교육성과의 성취와 관련, 학장과 보직자를 정기적으로 평가할 수 있는 체계와 실적이 있는지 확인한다. | • 관련 규정<br>• 관련 경력<br>• 평가 실적 |

## 8.3 교육예산과 자원 할당

### 기본기준

**K.8.3.1 의과대학은 교육예산을 포함한 교육관련 재정에 관한 책임과 권한을 명시하고 있다.**

*자체평가연구보고서 작성 가이드*

가이드-1. 확보된 교육예산을 포함한 교육관련 재정에 대한 분명한 책임과 권한을 가지고 있다.
가이드-2. 학장은 실험실습비와 경상비를 전결하고 있다.
가이드-3. 학장이 자율적으로 집행할 수 있는 정책적 재원이 있다.
가이드-4. 예산은 독립적으로 편성되어 있으며, 예산편성에 대하여 구성원의 의견을 수렴하는 과정과 예산심의관련 회의가 있다.

*방문평가단 평가 가이드*

| 기준 | 핵심어 | 가이드 | 근거자료 |
|---|---|---|---|
| K.8.3.1 | • 교육예산<br>• 교육관련 재정<br>• 책임과 권한 | • 확보된 교육예산을 포함한 교육관련 재정에 대한 분명한 책임과 권한을 가지고 있는지 확인한다.<br>• 학장은 실험실습비와 경상비를 전결하고 있는지 확인한다.<br>• 학장이 자율적으로 집행할 수 있는 정책적 재원이 있는지 확인한다.<br>• 예산편성에 대하여 구성원의 의견을 수렴하는 과정과 예산심의관련 회의, 독립예산이 편성되어 있는지 확인한다. | • 관련 규정<br>• 실적 보고서<br>• 대학 전결규정 |

**[주]**

- 교육예산은 각 기관과 국가의 예산 관행에 따르며, 의과대학은 기획된 투명한 예산안에 따라 집행한다.
- 교육관련 재정(의학과에 배정된 학생실험실습비, 교육과정 및 교육 관련 개발과 운영비, 교수연수지원비, 교육관련 세미나 개최비, 학생봉사활동지원비 등)확보현황을 기술하고, 예산의 구성 시에 의견수렴에 대한 규정이 명시되어야 한다. 또한, 의견수렴 자료를 제시하고 교육관련 재정확충을 위한 노력이 포함되어야 한다.

기본기준

**K.8.3.2 의과대학은 교육과정의 실행을 위해 필요한 자원을 확보하고 교육적 요구에 필요한 자원을 할당하고 있다.**

*자체평가연구보고서 작성 가이드*

가이드-1. 교육과정의 실행을 위해 필요한 자원을 확보하고, 교육적 요구에 필요한 자원을 할당하고 있다.
가이드-2. 교육재정이 등록금 인상에 비례하여 증가하고 있다.
가이드-3. 교육관련 재정을 확충하기 위한 노력이 있다.
가이드-4. 교육과정과 관련된 위원회 예산이 적절하다.

*방문평가단 평가 가이드*

| 기준 | 핵심어 | 가이드 | 근거자료 |
|---|---|---|---|
| K.8.3.2 | • 교육관련 자원 할당 | • 교육과정의 실행을 위해 필요한 자원을 확보하고, 교육적 요구에 필요한 자원을 할당하고 있는지 확인한다.<br>• 교육재정이 등록금 인상에 비례하여 증가하고 있는지 확인한다.<br>• 교육관련 재정을 확충하기 위한 노력이 있는지 확인한다.<br>• 교육과정과 관련된 위원회 예산이 적절한지 확인한다. | • 최근 6년간 대학의 교육관련 재정과 등록금 현황<br>• 관련 활동 자료<br>• 교육과정 관련 위원회의 규정, 예산, 집행 실적 |

**[주]**

- 자원 할당은 의과대학의 자율성을 전제로 한다(K.1.2.1 참조).
- 교육예산과 자원 할당에 학생지원과 학생관련 단체를 고려한다(K.4.3.4.과 4.4 참조).
- 적절한 교육과정과 관련된 위원회 예산은 연간 3천만 원 이상이다.

## 8.4 행정직원과 관리

### 기본기준

**K.8.4.1 의과대학은 행정업무가 구분되어 있고, 적정 수의 행정업무 담당직원을 확보하고 있다.**

| *자체평가연구보고서 작성 가이드* |
|---|
| 가이드-1. 행정업무가 교육, 교수, 학생, 연구, 졸업 후 교육, 입학, 재정 등으로 구분되어 있다.<br>가이드-2. 적정수의 행정업무 담당직원을 확보하고 있으며, 업무가 적절히 분장되어 있다. |

*방문평가단 평가 가이드*

| 기준 | 핵심어 | 가이드 | 근거자료 |
|---|---|---|---|
| K.8.4.1 | • 행정업무<br>• 담당직원 | • 행정업무가 교육, 교수, 학생, 연구, 졸업 후 교육, 입학, 재정 등으로 구분되어 있는지 확인한다.<br>• 적정수의 행정업무 담당 직원을 확보하고 있으며, 업무가 적절히 분장되어 있는지 확인한다. | • 관련 규정<br>• 행정 체계도<br>• 구체적 활동 실적 |

**[주]**

- 행정직원은 5명 이상이어야 하며, 행정조교는 인정하지 않는다.

우수기준

### H.8.4.1 의과대학은 행정관리의 질 향상을 위한 자체 프로그램을 수립하고 실행하고 있다.

| *자체평가연구보고서 작성 가이드* |
|---|
| 가이드-1. 행정관리의 질 향상을 위한 자체 프로그램을 수립하고 실행하고 있다.<br>가이드-2. 행정직원은 해당 업무에 대한 경력이나 전문성 개발을 위해 노력하고 있다. |

*방문평가단 평가 가이드*

| 기준 | 핵심어 | 가이드 | 근거자료 |
|---|---|---|---|
| H.8.4.1 | • 행정관리<br>• 질 향상<br>• 전문성 개발 | • 행정관리의 질 향상을 위한 자체 프로그램을 수립하고 실행하고 있는지 확인한다.<br>• 행정직원은 해당 업무에 대한 경력이나 전문성 개발을 위한 노력이 있는지 확인한다. | • 관련 규정<br>• 행정업무의 질 향상 실적<br>• 행정직원의 전문성 개발 실적 |

## 8.5 보건의료분야와의 상호작용

### 기본기준

**K.8.5.1 의과대학은 보건의료분야 및 보건의료 관련분야와 건설적인 상호작용을 하고 있다.**

| *자체평가연구보고서 작성 가이드* |
|---|
| 가이드-1. 보건의료분야 및 보건의료 관련분야와 건설적인 상호작용을 하고 있다. |

*방문평가단 평가 가이드*

| 기준 | 핵심어 | 가이드 | 근거자료 |
|---|---|---|---|
| K.8.5.1 | • 보건의료분야<br>• 보건의료 관련분야<br>• 상호작용 | • 보건의료분야 및 보건의료 관련분야와 건설적인 상호작용을 하고 있는지 확인한다. | • 관련 규정<br>• 관련 실적 |

**[주]**

- 건설적인 상호작용은 정보의 교환, 협력과 새로운 조직계획을 설정하고 수립하는 것을 내포한다. 이는 사회에서 요구되는 자질을 갖춘 의사들의 공급을 촉진한다.
- 보건의료분야는 공공 혹은 민간 보건의료서비스 전달체계와 의학연구기관을 포함한다.
- 보건의료 관련분야는 사회적 이슈, 지역 기관에 따라 건강증진과 질병예방을 위한 영향력을 가진 기관, 규제기구를 포함한다(예: 환경, 영양, 사회적 책무성을 가진다).

## 우수기준

### H.8.5.1 의과대학은 교직원과 학생의 참여를 포함하여 보건의료분야 관계자와 협력을 공식화하고 있다.

| *자체평가연구보고서 작성 가이드* |
|---|
| 가이드-1. 교직원과 학생의 참여를 포함하여 보건의료분야의 관계자와의 협력을 공식화하고 있다. |

*방문평가단 평가 가이드*

| 기준 | 핵심어 | 가이드 | 근거자료 |
|---|---|---|---|
| H.8.5.1 | • 교직원<br>• 학생<br>• 참여<br>• 보건의료분야<br>• 건설적인 상호작용 | • 교직원과 학생의 참여를 포함하여 보건의료 분야의 관계자와의 협력을 공식화하고 있는지 확인한다. | • 관련 규정<br>• 관련 실적 |

**[주]**

- 협력체계를 갖춘다는 것은 협력의 내용과 형식을 기술한 공식적인 협약에 들어감을 의미하며, 합동계약, 협력위원회와 합동프로젝트의 수립을 의미한다.

ASK2019

# 09 지속적 개선

## 9.0 지속적 개선

### 기본기준

**K.9.0.1 의과대학은 대학 발전계획이 있고, 대학 발전기금을 운용하며, 동문 또는 지역사회의 참여를 유도하는 노력을 하고 있다.**

*자체평가연구보고서 작성 가이드*

가이드-1. 대학 발전계획에 대학본부 또는 학교법인의 지원과 발전계획의 진행이 적절하다.
가이드-2. 대학 발전을 위한 동문 또는 지역사회 참여를 유도하기 위해 노력하고 있다.
가이드-3. 대학 발전기금의 규모와 운용이 적절하다.

*방문평가단 평가 가이드*

| 기준 | 핵심어 | 가이드 | 근거자료 |
|---|---|---|---|
| K.9.0.1 | • 발전계획<br>• 발전기금<br>• 동문 또는 지역사회 참여 | • 대학 발전계획에 대학본부 또는 학교법인의 지원과 발전계획의 진행이 적절한지 확인한다.<br>• 대학 발전을 위한 동문 또는 지역사회 참여를 유도하기 위해 노력하고 있는지 확인한다.<br>• 대학 발전기금의 규모(질적, 양적)와 운용(모금에서 정산까지)이 적절한지 확인한다. | • 발전계획서<br>• 발전계획 진행상황 자료<br>• 발전기금 관련 자료<br>• 관련 위원회 활동 자료<br>• 관련 규정 |

**[주]**

- 대학의 경쟁력 제고를 위한 실현가능한 중장기 발전계획을 가지고 있어야 하며, 대학본부의 지원과 이를 위한 위원회의 구성이 명문화되고 실제 실적을 포함해야 한다.

## 기본기준

**K.9.0.2 의과대학은 지속적인 질 관리와 개선을 위해 자체평가업무를 수행하는 상설기구를 갖추고 있고, 관련예산을 편성하여 적절하게 운영하고 있다.**

| *자체평가연구보고서 작성 가이드* |
| --- |
| 가이드-1. 자체평가업무를 수행하는 상설기구를 갖추고 적절한 예산을 확보하고 있다.<br>가이드-2. 자체평가업무를 수행하는 상설기구의 운영과 실적이 적절하다. |

*방문평가단 평가 가이드*

| 기준 | 핵심어 | 가이드 | 근거자료 |
| --- | --- | --- | --- |
| K.9.0.2 | • 지속적인 질 관리와 개선(CQI, Continuous Quality Improvement)<br>• 상설기구<br>• 운영 예산 | • 자체평가업무를 수행하는 상설기구를 갖추고 적절한 예산을 확보하고 있는지 확인한다.<br>• 자체평가업무를 수행하는 상설기구의 운영과 실적이 적절한지 확인한다. | • 관련 규정<br>• 관련 예산<br>• 자체평가 활동 자료 |

## 기본기준

**K.9.0.3 의과대학은 평가인증 결과를 대학운영에 반영하여 지속적으로 개선한 실적이 있다.**

| *자체평가연구보고서 작성 가이드* |
| --- |
| 가이드-1. 평가인증 결과를 대학운영에 적절히 반영하고 있다.<br>가이드-2. 지속적으로 개선한 실적이 있다. |

*방문평가단 평가 가이드*

| 기준 | 핵심어 | 가이드 | 근거자료 |
| --- | --- | --- | --- |
| K.9.0.3 | • 평가인증 결과<br>• 지속적 개선 | • 평가인증 결과를 대학운영에 적절히 반영하였는지 확인한다.<br>• 지속적으로 개선한 실적이 있는지 파악한다. | • 개선 계획서<br>• 이전 평가인증 미비점 개선 실적 대비표 |

## 우수기준

**H.9.0.1 의과대학은 대학 발전관련 전향적 연구와 분석, 지역사회 평가결과와 의학교육 관련 문헌에 근간하여 대학운영을 지속적으로 개선하고 있다.**

*자체평가연구보고서 작성 가이드*

가이드-1. 전향적인 연구와 분석을 통해 대학운영을 지속적으로 개선하고 있다.
가이드-2. 지역사회 평가를 검토하여 대학운영을 지속적으로 개선하고 하고 있다.
가이드-3. 의학교육 문헌을 검토하여 대학운영을 지속적으로 개선하고 있다.

*방문평가단 평가 가이드*

| 기준 | 핵심어 | 가이드 | 근거자료 |
|---|---|---|---|
| H.9.0.1 | • 전향적 연구와 분석<br>• 지역사회 평가 결과<br>• 의학교육 관련 문헌 | • 전향적인 연구와 분석을 통해 대학운영을 지속적으로 개선하고 하고 있는지 확인한다.<br>• 지역사회 평가를 결과를 검토하여 대학운영을 지속적으로 개선하고 하고 있는지 확인한다.<br>• 의학교육 문헌을 검토하여 대학운영을 지속적으로 개선하고 있는지 확인한다. | • 개선계획 관련 자료<br>• 전향적 연구 분석 자료<br>• 지역사회 평가<br>• 개선에 활용한 의학교육 관련 문헌 분석자료 |

**[주]**

- 전향적 연구는 국가별 모범사례에 대한 자료와 증거의 수집과 생성을 위한 연구를 포함한다.
- 지역사회 평가는 의과대학에 대한 지역사회(전국적 평가 포함)의 의료인(의료기관) 또는 비의료인(비의료기관)의 평가를 의미한다.

## 우수기준

**H.9.0.2 의과대학은 대학운영과 관련하여 과거에 도출된 개선사항을 현재와 미래 전망에 따라 대학의 정책과 실행에 반영되도록 보장하고 있다.**

| *자체평가연구보고서 작성 가이드* |
|---|
| 가이드-1. 개선을 위해 과거의 경험을 바탕으로 분석을 시행하고 있다.<br>가이드-2. 현재의 활동과 미래의 전망에 따라 분석결과를 재조명하고 있다.<br>가이드-3. 재조명된 결과를 대학의 정책과 실행에 반영하고 있다. |

*방문평가단 평가 가이드*

| 기준 | 핵심어 | 가이드 | 근거자료 |
|---|---|---|---|
| H.9.0.2 | • 과거의 경험 분석<br>• 현재의 활동 검토<br>• 미래의 전망 조명<br>• 정책과 실행에 반영 | • 개선을 위해 과거의 경험을 바탕으로 분석하고 있는지 확인한다.<br>• 현재의 활동과 미래의 전망에 따라 분석결과를 재조명하고 있는지 확인한다.<br>• 재조명된 결과를 대학의 정책과 실행에 반영하고 있는지 확인한다. | • 발전계획 관련 자료<br>• 이전 평가인증(1주기~최근) 미비점 개선 실적 대비표 |

## 우수기준

**H.9.0.3 의과대학은 지속적 개선 시에 지역사회의 과학, 사회경제, 문화 등의 발전에 맞추어 사명과 졸업성과를 수정하고 있다.**

| *자체평가연구보고서 작성 가이드* |
|---|
| 가이드-1. 대학이 속한 지역사회의 과학, 사회경제, 문화 등을 파악하고 있다.<br>가이드-2. 지역사회의 여러 분야의 발전을 토대로 사명과 졸업성과를 검토하고 있다(평가부문 1.1 참조). |

*방문평가단 평가 가이드*

| 기준 | 핵심어 | 가이드 | 근거자료 |
|---|---|---|---|
| H.9.0.3 | • 지역사회의 변화<br>• 사명과 졸업성과 | • 대학이 속한 지역사회의 과학, 사회경제, 문화 등의 변화를 파악하고 있는지 확인한다.<br>• 지역사회의 여러 분야의 발전을 토대로 사명과 졸업성과를 검토하고 있는지 확인한다. | • 관련 자료<br>• 이전 평가인증(1주기~최근) 미비점 개선 실적 대비표 |

## 우수기준

### H.9.0.4 의과대학은 졸업생이 처하게 될 환경변화에 따라 지속적으로 졸업성과를 수정하고 있다.

*자체평가연구보고서 작성 가이드*

가이드-1. 졸업생의 환경을 분석하여 졸업성과를 수정하고 수정한 사항을 개선에 반영하고 있다(평가부문 1.3 참조).
가이드-2. 졸업생의 졸업 후에 이루어질 임상진료를 고려하여 졸업성과를 검토하고 있다.

*방문평가단 평가 가이드*

| 기준 | 핵심어 | 가이드 | 근거자료 |
|---|---|---|---|
| H.9.0.4 | • 졸업 후 환경변화 분석<br>• 졸업성과 수정 | • 졸업생의 환경을 고려하여 졸업성과를 수정하고 수정한 사항을 개선에 반영하고 있는지 확인한다.<br>• 졸업생의 졸업 후에 이루어질 임상진료를 고려하여 졸업성과를 검토하고 있는지 확인한다. | • 관련 자료<br>• 이전 평가인증(1주기~최근) 미비점 개선 실적 대비표 |

**[주]**

- 졸업성과의 수정에는 졸업 후의 환경변화에 맞게 술기, 공공 보건의료교육, 환자진료 참여 등을 포함한다.

## 우수기준

### H.9.0.5 의과대학은 교육과정, 교육방법의 적절성과 이들의 관련성을 보장하기 위해 지속적으로 교육과정과 교육방법을 수정하고 있다.

*자체평가연구보고서 작성 가이드*

가이드-1. 교육과정과 교육방법에 대한 분석이 적절히 이루어지고 있다(평가부문 2.1 참조).
가이드-2. 교육과정과 교육방법을 적절히 수정하고 있다.

*방문평가단 평가 가이드*

| 기준 | 핵심어 | 가이드 | 근거자료 |
|---|---|---|---|
| H.9.0.5 | • 교육과정 분석 및 수정<br>• 교육방법 분석 및 수정 | • 교육과정과 교육방법에 대한 분석이 적절하게 이루어졌는지 확인한다.<br>• 교육과정과 교육방법을 적절히 수정하고 있는지 확인한다. | • 관련 자료<br>• 이전 평가인증(1주기~최근) 미비점 개선 실적 대비표 |

## 우수기준

### H.9.0.6 의과대학은 교육환경의 변화를 적절하게 반영하여 지속적으로 교육과정을 개선하고 있다.

*자체평가연구보고서 작성 가이드*

가이드-1. 교육환경의 변화에 대한 분석을 통해 교육과정을 개선하고 있다(평가부문 2.2~2.6 참조).
가이드-2. 교육과정의 개선에 현실에 맞지 않는 지식, 개념, 교육방법 등은 폐기하고 있다.
가이드-3. 교육과정의 개선에 새로운 관련 지식, 개념, 교육방법을 포함하고 있다.

*방문평가단 평가 가이드*

| 기준 | 핵심어 | 가이드 | 근거자료 |
|---|---|---|---|
| H.9.0.6 | • 교육 환경의 변화<br>• 교육과정 개선 | • 교육환경의 변화에 대한 분석을 통해 교육과정을 개선하고 있는지 확인한다.<br>• 교육과정의 개선에 현실에 맞지 않는 지식, 개념, 교육방법 등은 폐기하고 있는지 확인한다.<br>• 교육과정의 개선에 새로운 관련 지식, 개념, 교육방법을 포함하고 있는지 확인한다. | • 관련 자료<br>• 이전 평가인증(1주기~최근) 미비점 개선 실적 대비표 |

## 우수기준

### H.9.0.7 의과대학은 의도한 교육성과와 교육방법의 변화에 따라 지속적으로 평가원칙과 방법을 수정하고 있다.

*자체평가연구보고서 작성 가이드*

가이드-1. 의도한 교육성과와 교육방법의 변화에 따라 지속적으로 평가원칙과 방법을 수정하고 있다(평가부문 3.1과 3.2 참조).

*방문평가단 평가 가이드*

| 기준 | 핵심어 | 가이드 | 근거자료 |
|---|---|---|---|
| H.9.0.7 | • 평가원칙과 방법의 수정 | • 의도한 교육성과와 교육방법의 변화에 따라 지속적으로 평가원칙과 방법을 수정하고 있는지 확인한다. | • 관련 자료<br>• 이전 평가인증(1주기~최근) 미비점 개선 실적 대비표 |

**[주]**

- 의도한 교육성과는 K.1.3.3 참조.

우수기준

**H.9.0.8 의과대학은 사회적 요구의 변화에 따라 지속적으로 교수채용 방식과 교수개발 정책을 수정하고 있다.**

*자체평가연구보고서 작성 가이드*

가이드-1. 변화하는 사회적 요구에 따라 교수채용 방식과 교수개발 정책을 수정하고 있다(평가부문 5.1, 5.2 참조).

*방문평가단 평가 가이드*

| 기준 | 핵심어 | 가이드 | 근거자료 |
|---|---|---|---|
| H.9.0.8 | • 교수채용 방식의 수정<br>• 교수개발 정책의 수정 | • 변화하는 사회적 요구에 따라 교수채용 방식을 수정하고 있는지 확인한다.<br>• 변화하는 사회적 요구에 따라 교수개발 정책을 수정하고 있는지 확인한다. | • 관련 자료<br>• 이전 평가인증(1주기~최근) 미비점 개선 실적 대비표 |

우수기준

**H.9.0.9 의과대학은 변화하는 사회적 요구를 바탕으로 교육자원을 지속적으로 조정하고 있다.**

*자체평가연구보고서 작성 가이드*

가이드-1. 변화하는 사회적 요구에 따라 특별 전형에 의한 입학생 수, 교육과 연구 지원과 관련된 직원의 수와 경력, 교육프로그램 등과 같은 교육자원을 지속적으로 조정하고 있다(평가부문 6.1~6.3 참조)

*방문평가단 평가 가이드*

| 기준 | 핵심어 | 가이드 | 근거자료 |
|---|---|---|---|
| H.9.0.9 | • 교육자원의 조정 | • 변화하는 사회적 요구에 따라 특별 전형에 의한 입학생 수, 교육과 연구 지원과 관련된 직원의 수와 경력, 교육프로그램 등과 같은 교육자원을 지속적으로 조정하고 있는지 확인한다. | • 관련 자료<br>• 이전 평가인증(1주기~최근) 미비점 개선 실적 대비표 |

## 우수기준

### H.9.0.10 의과대학은 전체 프로그램 모니터링과 평가과정을 지속적으로 개선하고 있다.

*자체평가연구보고서 작성 가이드*

가이드-1. 전체 프로그램 모니터링을 통해 평가과정을 지속적으로 개선하고 있다(평가부문 7.1~7.3 참조).

*방문평가단 평가 가이드*

| 기준 | 핵심어 | 가이드 | 근거자료 |
|---|---|---|---|
| H.9.0.10 | • 평가과정 개선 | • 전체 프로그램 모니터링을 통해 평가과정이 지속적으로 개선되고 있는지 확인한다. | • 관련 자료<br>• 이전 평가인증(1주기~최근) 미비점 개선 실적 대비표 |

## 우수기준

### H.9.0.11 의과대학은 의료 환경의 변화와 이해관계자의 요구에 따라 대학운영체계를 지속적으로 개선하고 있다.

*자체평가연구보고서 작성 가이드*

가이드-1. 지속적으로 변하는 의료 환경과 의과대학의 요구를 개선방안에 반영하고 있다(평가부문 8.1~8.5 참조).

가이드-2. 이해관계자의 의견을 반영하여 대학운영체계를 지속적으로 개선하고 있다.

*방문평가단 평가 가이드*

| 기준 | 핵심어 | 가이드 | 근거자료 |
|---|---|---|---|
| H.9.0.11 | • 이해관계 조절<br>• 적절한 관리 방식 | • 지속적으로 변하는 의료 환경과 의과대학의 요구를 개선방안에 반영하고 있는지 확인한다.<br>• 이해관계자의 의견을 반영하여 대학운영체계를 지속적으로 개선하고 있는지 확인한다. | • 관련 자료<br>• 이전 평가인증(1주기~최근) 미비점 개선 실적 대비표 |

# 한국의학교육평가원 평가인증기준의 변천과 발전방향

저 자 / 박 원 균 외

발행일 / 2019년 10월 발행

발행인 / 김 영 창

발행처 / (재)한국의학교육평가원

(03129)서울특별시 종로구 대학로1길 10 연지드림빌 203호

Tel. 02.795.1591 Fax. 02.795.1592
